# The impact of sedative and vasopressor agents on cerebrovascular reactivity in severe traumatic brain injury

**DOI:** 10.1186/s40635-023-00524-4

**Published:** 2023-08-05

**Authors:** Logan Froese, Emma Hammarlund, Cecilia A. I. Åkerlund, Jonathan Tjerkaski, Erik Hong, Caroline Lindblad, David W. Nelson, Eric P. Thelin, Frederick A. Zeiler

**Affiliations:** 1 Biomedical Engineering, Faculty of Engineering, University of Manitoba, Winnipeg, Canada; 2 Department of Clinical Neuroscience, Karolinska Institutet, Stockholm, Sweden; 3 Department of Neurology, Karolinska University Hospital, Stockholm, Sweden; 4 Department of Perioperative Medicine and Intensive Care, Karolinska University Hospital, Stockholm, Sweden; 5 Section of Perioperative Medicine and Intensive Care, Department of Physiology and Pharmacology, Karolinska Institutet, Stockholm, Sweden; 6 Department of Neurosurgery, Uppsala University Hospital, Uppsala, Sweden; 7 Section of Neurosurgery, Department of Surgery, Rady Faculty of Health Sciences, University of Manitoba, Winnipeg, MB Canada; 8 Department of Human Anatomy and Cell Science, Rady Faculty of Health Sciences, University of Manitoba, Winnipeg, Canada; 9 Centre On Aging, University of Manitoba, Winnipeg, Canada; 10 Division of Anaesthesia, Department of Medicine, Addenbrooke’s Hospital, University of Cambridge, Cambridge, UK; 11 Department of Medical Sciences, Uppsala University, Uppsala, Sweden

**Keywords:** Autoregulation, Cerebrovascular reactivity, Sedative drugs, Vasopressors

## Abstract

**Background:**

The aim of this study is to evaluate the impact of commonly administered sedatives (Propofol, Alfentanil, Fentanyl, and Midazolam) and vasopressor (Dobutamine, Ephedrine, Noradrenaline and Vasopressin) agents on cerebrovascular reactivity in moderate/severe TBI patients. Cerebrovascular reactivity, as a surrogate for cerebral autoregulation was assessed using the long pressure reactivity index (LPRx). We evaluated the data in two phases, first we assessed the minute-by-minute data relationships between different dosing amounts of continuous infusion agents and physiological variables using boxplots, multiple linear regression and ANOVA. Next, we assessed the relationship between continuous/bolus infusion agents and physiological variables, assessing pre-/post- dose of medication change in physiology using a Wilcoxon signed-ranked test. Finally, we evaluated sub-groups of data for each individual dose change per medication, focusing on key physiological thresholds and demographics.

**Results:**

Of the 475 patients with an average stay of 10 days resulting in over 3000 days of recorded information 367 (77.3%) were male with a median Glasgow coma score of 7 (4–9). The results of this retrospective observational study confirmed that the infusion of most administered agents do not impact cerebrovascular reactivity, which is confirmed by the multiple linear regression components having *p* value > 0.05. Incremental dose changes or bolus doses in these medications in general do not lead to significant changes in cerebrovascular reactivity (confirm by Wilcoxon signed-ranked *p* value > 0.05 for nearly all assessed relationships). Within the sub-group analysis that separated the data based on LPRx pre-dose, a significance between pre-/post-drug change in LPRx was seen, however this may be more of a result from patient state than drug impact.

**Conclusions:**

Overall, this study indicates that commonly administered agents with incremental dosing changes have no clinically significant influence on cerebrovascular reactivity in TBI (nor do they impair cerebrovascular reactivity). Though further investigation in a larger and more diverse TBI patient population is required.

**Supplementary Information:**

The online version contains supplementary material available at 10.1186/s40635-023-00524-4.

## Background

Guideline-based care for moderate/severe traumatic brain injury (TBI) patients use various pharmacological agents as the cornerstones of treatment. Sedation agents are employed to manage intracranial pressure (ICP), facilitate patient management and suppress cerebral metabolic demand [1–3]. Vasopressor agents are utilized to maintain cerebral perfusion pressure (CPP) targets, commonly at approximately 60–70 mmHg [3]. Despite these agents being utilized in care, a refined understanding of the momentary cerebral responses to these agents is limited, with only a small number of studies assessing their impact in TBI [4, 5].

The pressure reactivity index (PRx; correlation between slow-wave of ICP and mean arterial pressure (MAP)) is the most common measure of cerebrovascular reactivity in TBI [6–8], with multiple studies linking impaired cerebrovascular reactivity and poor patient outcome [9–14]. Furthermore, current therapies guided by the Brain Trauma Foundation (BTF), which includes the use of sedative and vasopressor agents, have demonstrated a limited impact on cerebrovascular reactivity [12, 15–17]. Thus, cerebrovascular reactivity monitoring, and its derived metrics, may offer a route to improve patient outcome and may play an important role in cerebral physiologic dysfunction [16, 18].

Currently within the literature there are only a few studies that have assessed the impact of guideline-based therapeutics on cerebrovascular reactivity, with most studies using large time aggregate data, which may miss high-frequency physiological associations [5, 12, 15, 16, 19, 20]. Moreover, the Collaborative European NeuroTrauma Effectiveness Research in TBI (CENTER-TBI) study documented that therapeutic intensity levels (TIL) had little connection to impaired cerebrovascular reactivity, further questioning the role of these agents [16]. Finally, two studies on small populations of critically ill patients found that incremental changes in sedative and vasopressor agents failed to provoke significant responses in cerebrovascular reactivity [4, 5]. Although, again besides from these last two studies, previous work conducted used aggregates of physiology and medication administration data which lacked high temporal resolution (relying on daily treatment measures) [21, 22].

Thus, to both improve therapeutic care and improve the management of CPP and ICP, it is imperative that analyses clearly document the temporal impacts of current therapeutics on cerebrovascular response. As such, the goal of this study was to assess the influence that some commonly utilized sedative and vasopressor agents have on PRx, with a secondary interest in the impact that such agents have on cerebral physiology (primarily ICP). All of this will be accomplished using archived high-frequency physiology data and treatment information from the Department of Neurosurgery, Karolinska University Hospital.

## Materials and methods

### Study design

Patients with moderate to severe TBI (diagnosed as GCS ≤ 8 and > 15 years old) admitted to the neurointensive care unit at Karolinska University Hospital, Stockholm, Sweden, a level one trauma center, between January 1, 2006 to December 31, 2019 were included in this study. These patients had invasive ICP monitoring and archived high-frequency physiology (ICP and arterial blood pressure; ABP) and were retrospectively analyzed (*N* = 475) in this observational study. Patients received treatment according to local guidelines in general concordance to that of the Brain Trauma Foundation (BTF) [3, 23, 24], and is described in detail elsewhere [25]. Note all patients were mechanically ventilated and PaCO_2_ targets were used, where normal to mild hyperventilation (defined here as PaCO_2_ 4.5–5 kPa) was commonly applied as one of several measures to manage increased ICP. Heads are commonly elevated 30 degrees. CPP is calculated with the arterial pressure dome placed at the mid cerebral level.

### IRB ethics

Study was approved by the Swedish Ethical Review Authority (#2020-05227) on November 17, 2020 and adheres to the Helsinki Declaration of 1975.

### Data collection

For this ongoing prospective TBI database, all patient demographics, injury and treatment information were either manually collected by a medical professional or automatically recorded using Clinisoft (Centricity Critical Care, CCC, General Electric Company, Boston). The drug infusion rates and physiological variables were recorded with Clinisoft, which included the timestamped pharmacological and physiological data. In this study, continuous infusions as well as bolus doses of the sedatives propofol, midazolam, morphine, fentanyl and alfentanil and the vasopressor agents dobutamine, noradrenaline, vasopressin, and ephedrine, and the physiological variables (ABP and ICP) were analyzed.

Arterial blood pressure (ABP) was obtained through either radial or femoral arterial lines connected to pressure transducers (Baxter Healthcare Corp. CardioVascular Group, Irvine, CA, or similar devices). ICP was acquired via an intra-parenchymal strain gauge probe (Codman ICP MicroSensor; Codman & Shurtleff Inc., Raynham, MA), raumedic catheter Neurovent-P (Raumedic AG, Münchberg, Germany), parenchymal fiber optic pressure sensor (Camino ICP Monitor, Integra Life Sciences, Plainsboro, NJ, United States; https://www.integralife.com/) or using external ventricular drains (Medtronic, Minneapolis, MN). Again, both ABP and ICP data were directly linked to a Clinisoft database.

### Signal processing

The following signal processing occurred using similar methodology, covered in other publications by our group and the senior author [4, 12, 18, 19, 26]. Data collected were stored in the database as the median for each time period, ranging from 0.5 to 5 min, generating unevenly sampled time-series data. To transform the data to an evenly sampled time series (at 1 min), we imputed the data over a 20-min window using locally weighted estimated scatterplot smoothing (LOWESS), thus generating an imputed minute-by-minute median time-series value for ICP and mean arterial blood pressure (MAP). CPP was then calculated as MAP-ICP.

Cerebrovascular reactivity was assessed through low-frequency PRx (LPRx) which was derived via the moving correlation coefficient of multiple consecutive minute-by-minute samples of ICP and MAP, to give a LPRx value updated every minute [27–29]. The LPRx was found for 10, 15, 20, 30 and 60 consecutive samples (10–60 min of time) and labeled as: LPRx_10, LPRx_15, LPRx_20, LPRx_30, and LPRx_60; in line with previous literature on LPRx in TBI [27–29]. LPRx values range from -1 to 1, with higher values indicating increasingly impaired cerebrovascular reactivity, indicated thresholds for impaired reactivity range from 0, 0.25 and 0.35. [27–29]

### Statistical analysis

All statistical analysis was performed using R statistical computing software. (R Foundation for Statistical Computing (2020), Vienna, Austria, http://www.R-project.org/). This manuscript performed an exploration into the relationships between various treatment agents and cerebrovascular reactivity as well as generic blood pressure measures ICP, CPP and MAP. To accomplish this, the continuous infusion minute-by-minute data were compared to various physiological variables. Also, the mean physiological variable before and after an agent infusion change (both bolus and continuous) were compared, assessing how the change in dosing impacts the overall variable response.

### Minute-by-minute continuous infusion evaluation

The minute-by-minute continuous infusion information was time linked with the pharmacological information from which various comparisons could be performed. Not all agents had continuous infusions thus only midazolam, morphine, propofol, dobutamine, vasopressin and adrenaline were assessed for this minute-by-minute analysis (see Additional file 1: Appendix C and O for more details about infusion timings).

#### LOESS infusion changes

From this data locally estimated scatterplot smoothing (LOESS) plots were used to visually compare the impact of different dosages of the infusion agents on given physiological measures: MAP, ICP, CPP, LPRx_10, LPRx_15, LPRx_20, LPRx_30 and LPRx_60. To derive the LOESS plots, the continuous agents’ infusion rates were paired with their respective minute-by-minute timestamped physiology, thus for every minute there was the physiology and indicated infusion rate. This data was then grouped for all desired continuous infusion agents over the entire data set to give one LOESS plot per agent per physiology. Dosing amounts were adjusted to a standard amount per kilogram (kg) of the patient.

#### Evaluation using multiple linear modeling

Given that most patients had multiple continuous infusions given at the same time, including different types of sedative and pressors, a multivariant linear regression analysis was performed on this data [30–32]. The general methodology for multiple linear regression modeling was done with the minute-by-minute drug infusion and physiological information. From past literature it is known that the physiological variables are inherently linear, thus a first-order difference was applied to all the physiological variables (ICP, CPP, LPRx_10 and LPRx_60) to give the first-order differenced variable response [7, 33–35]. A multiple linear regression model was created for just the time when vasopressor agents were given, just when sedative agents were given and all the time when both sedative and vasopressor agents were given. For more information about linear regression analysis, we refer the interested reader to the following literature [30–32]. It should be noted that vasopressin and morphine were excluded for some of this analysis, given vasopressin had a limited amount of recorded data as compared to the other agents and morphine is not used in a sedative or pressor medication strategy.

#### Injury severity

Using the same data for the multiple infusion evaluation, this data was further sub-divided based on the patient’s Marshall CT score. Again, a multiple linear regression model was created for just the vasopressor agents, just the sedative agents and both the sedative and vasopressor agents [30–32].

Finally on the minute-by-minute data a one-way analysis of variance (ANOVA) test was performed, over all the main physiological variables and Marshall CT score. A Bonferroni adjustment was applied to this data given the generic nature of the test and the number of patients.

#### Pre-/post-drug change evaluation

For both the continuously infused agents and the bolus doses, all points in time where any drug agent was changed (increase, decrease or a bolus dose given) was marked. From this indexed data, date, time, infusion rate, ICP, ABP, CPP and LPRx data could be found. Then the physiology data both pre-/post-infusion rate change was used to find the grand mean value or % time over key thresholds. A 30-min window pre-/post-dose with a 15 min delay was used (thus each change was assessed over 1.25 h of data) and this allowed all agents to reach full onset response, which was taken from our previous work [4]. Any time window that had less than 50% of the data was discarded from the study. Note, the bolus and continuous infusions were in different groups for all analyses, to allow for the comparison of difference between continuous and bolus drug impacts.

The analyzed physiological variables thresholds were: % time LPRx > 0, % time LPRx > 0.25, % time LPRx > 0.35, % time ICP > 20 mmHg, % time ICP > 22 mmHg, % time CPP > 70 mmHg, and % time CPP < 60 mmHg [3, 36–38]. These thresholds are defined from previously described literature, with the time above these limits found to be associated with worse outcome at 6 to 12 months post-injury [3, 36–38].

With the pre-/post-drug change window data (the grand mean or % time over a key threshold) a Wilcoxon signed-ranked test was performed for this data for all agents separated into the agent dose change (increase, decrease or a bolus dose was given). Finally, a Bonferroni p value adjustment was used to account for multiple comparisons. The full list of agents assessed in this study was propofol, alfentanil, fentanyl, midazolam, morphine, dobutamine, ephedrine, noradrenaline and vasopressin which were assessed for the three outlined methods.

#### Evaluation of categories

The pre-/post- drug change window data were then further categorized into sub-groups based on physiology in the pre-dose window. Evaluation of the sub-groups included:

- Pre-time window over 50% time ICP > 20 mmHg.
- Pre-time window over 50% time ICP < 20 mmHg.
- Pre-time window over 50% time LPRx_10 > 0.
- Pre-time window over 50% time LPRx_10 < 0.
- Pre-time window over 50% time LPRx_10 > 0.35.
- Pre-time window over 50% time LPRx_10 < 0.35.
- Continuous infusion going from “Off to On” agent (and vice versa i.e., On to Off)—With the continuous infusion rate data, we could identify if the agent was switched on (from 0 to a positive number) or off (a positive number to 0).
- Assessing the high/medium/low infusion rates of continuous infusion agents (for more detail see Additional file 1: Appendix J)—The infusion rates were stratified by high, medium or low as described in Additional file 1: Appendix J. This allowed for further comparison of physiological response to the studied vasopressors and sedative pharmacologic agents.

For all comparisons, again a Wilcoxon signed-ranked test was performed between the pre- and post-physiological variable windows with Bonferroni correction to adjust for multiple comparisons.

## Results

### Patient characteristics

Table 1 provides the core patient characteristics for all the patients. The median age was 52 years (interquartile range; IQR: 34–62.5 years), with 367 (77.3%) being males with most patients having 10 days of recording resulting in over 3350 days of recording. The continuous infusion rates of these agents ranged from 0.001–7.5 mg/kg/h for propofol, 0.01–0.55 mg/kg/h for morphine, 0.001–0.8 mg/kg/h for midazolam, 0.001–19 μg/kg/min for dobutamine, 0.03–0.48 μg/kg/min for noradrenaline, and 0.07–1.1 Infusion Units/kg/h for vasopressin. Of the 475 patients, 49(10.3%) underwent a decompressive craniectomy and 88(18.5%) did not require hematoma evacuation surgery. These TBI demographics are in keeping with normal TBI cohorts.

**Table 1:** Demographic information

Variable	Median (interquartile range)/ number (%)
Number of patients	475
Male sex	367 (77.3%)
Age	52 (34–62.5)
GCS Eye score	1 (1–2)
GCS Motor score	4 (2–5)
GCS Verbal score	1 (1–2)
GCS	7 (4–9)
Pupil reactivity	
* Both reactive*	353 (74.3%)
* One reactive*	66 (13.9%)
* None reactive*	98 (20.6%)
Hypoxic	145 (30.5%)
Hypertension	156 (32.8%)
Marshall CT classification	5 (1–5)
V	257 (54.1%)
IV	0 (0%)
III	15 (3.2%)
II	78 (16.4%)
I	125 (26.3%)
Subarachnoid hematoma	387 (81.5%)
Epidural hematoma	72 (15.2%)
Craniotomy	289 (60.8%)
Decompressive craniectomy primary	40 (8.42%)
Decompressive craniectomy secondary	9 (1.89%)
External ventricular drain ICP monitoring	249 (52.4%)
GOS (~ 12 months)	3 (3–4)
5	68 (14.3%)
4	167 (35.2%)
3	145 (30.5%)
2	6 (1.3%)
1	89 (18.7%)
ICU length of stay (days)	10 (4.5–15.8)

GCS, Glasgow Coma Scale; GOS, Glasgow Outcome Scale; ICP, intracranial pressure; ICU, intensive care unit; hypoxic and hypertensive events are marked if any patient has had them during patient care

### Minute-by-minute continuous infusion evaluation

#### LOESS response

**Fig. 1:**
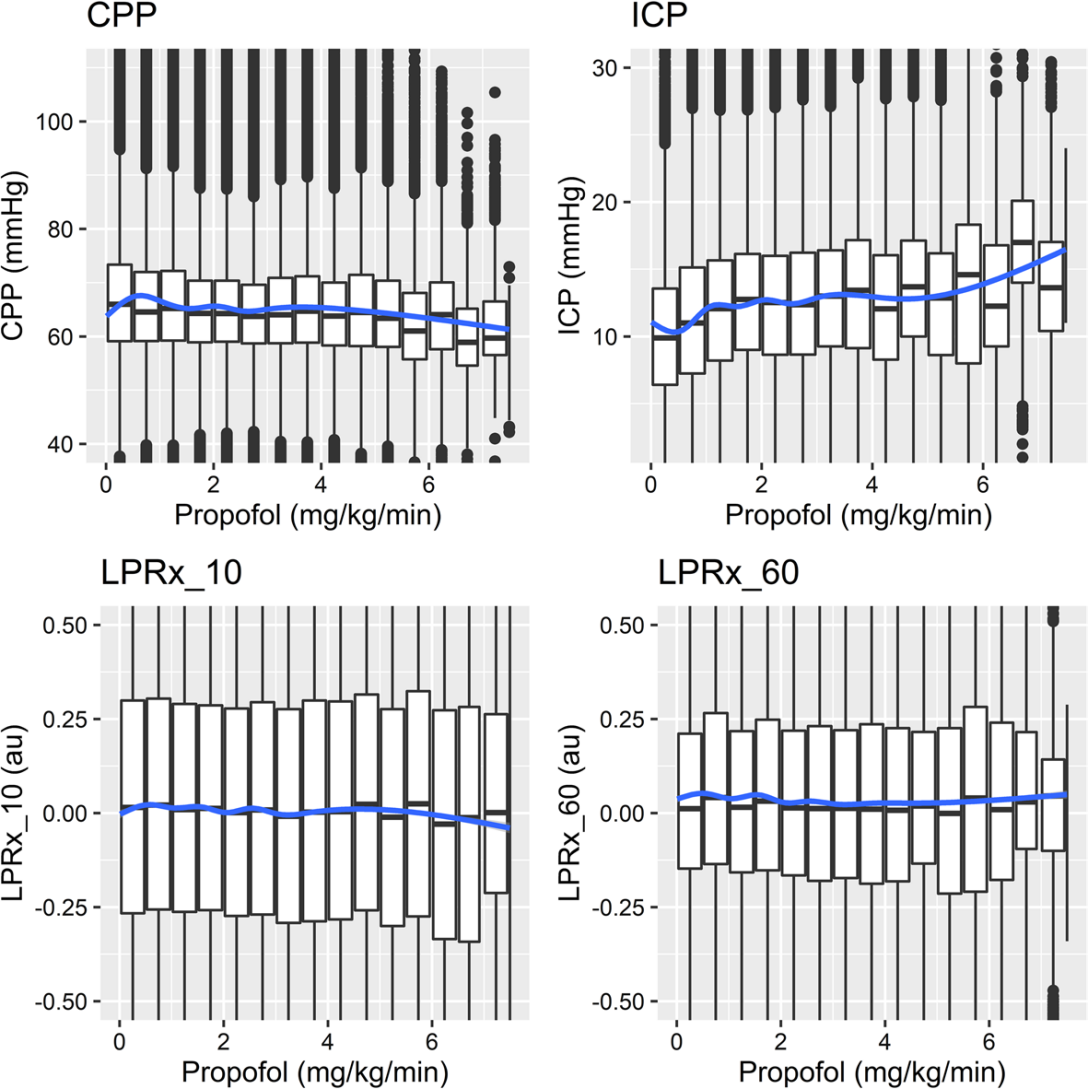
LOESS plots—propofol. Figure of the LOESS plots of different dose amounts of propofol and their associations to different physiological variables (ie minute-by- minute data paired with continuous infusion rate). Au, arbitrary units; CPP, cerebral perfusion pressure; hr, hour; ICP, intracranial pressure; kg, kilogram; LPRx_10, pressure reactivity over 10 min; LPRx_60, pressure reactivity over 60 min; mg, milligram; mmHg, millimeter of mercury; ug, microgram

**Fig. 2:**
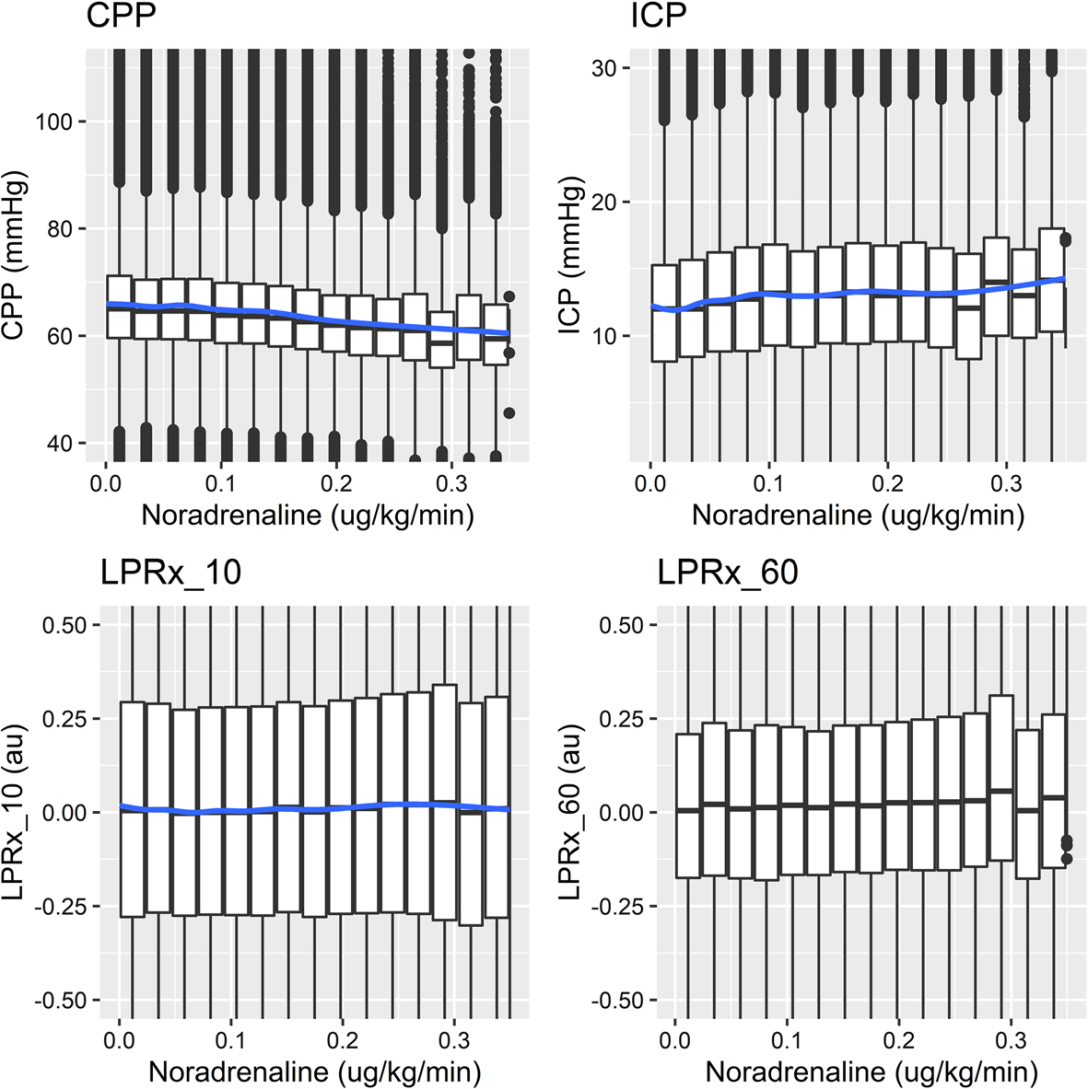
LOESS plot—noradrenaline. Figure of the LOESS plots of different dose amounts of noradrenaline and their associations to different physiological variables (i.e. minute-by-minute data paired with continuous infusion rate). Au, arbitrary units; CPP, cerebral perfusion pressure; h, hour; ICP, intracranial pressure; kg, kilogram; LPRx_10, pressure reactivity over 10 min; LPRx_60, pressure reactivity over 60 min; mg, milligram; mmHg, millimeter of mercury; μg, microgram

Figures 1 and 2 show the LOESS plot of propofol and noradrenaline, other data for this analysis can be found in Additional file 1: Appendix A and B. For the data there was a limited relation between the continuous infused agents and LPRx. Although morphine, propofol and midazolam LOESS curves can be seen related to changes in CPP and ICP at higher doses.

#### Multiple linear regression evaluation

Using the minute-by-minute data, various multiple linear regression models were created. As demonstrated in Additional file 1: Appendix C, the various agents used had a limited impact on ICP, CPP, LPRx_10 or LPRx_60. Notably, no p values reached significance with any infusion agent given nor did any of them have a high mean correlation (<|0.5|).

#### Injury severity

Using the minute-by-minute data sub-divided for Marshal CT score, various multiple linear regression models were created. As demonstrated in Additional file 1: Appendix D, the various agents used had a limited impact on ICP, CPP, LPRx_10 or LPRx_60 and was similar to the full data assessment for all CT scores.

The one-way ANOVA test did have ICP being modulated by Marshall CT score, however with the application of p value adjustment this significance went away. All other variables demonstrated a non-significant relationship between Marshal CT score and the fluctuations in variables (see Additional file 1: Appendix E).

### Pre/post drug change evaluation

#### Overall dose response

**Fig. 3:**
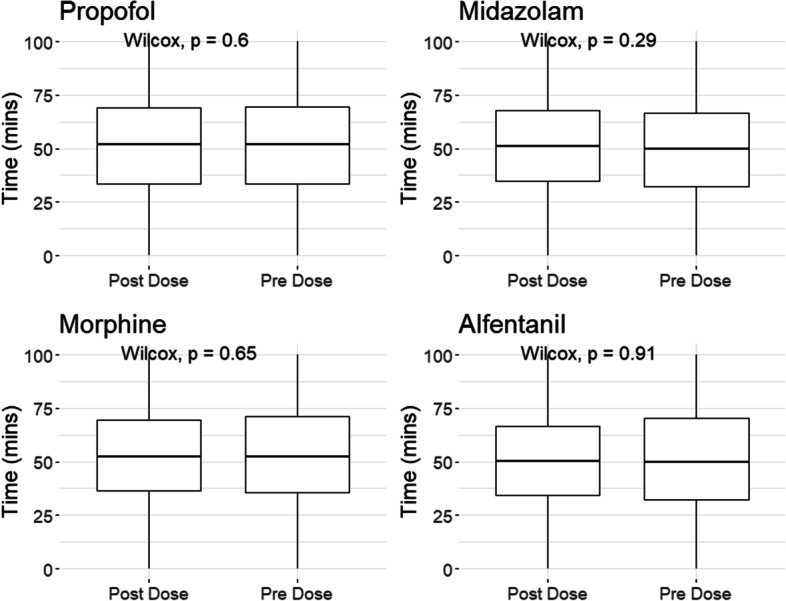
Box plots for bolus doses—% time LPRx_10 > 0. Figure of the boxplots of different bolus agents (and only bolus infusions), with the Wilcoxon signed-ranked value between the pre–post dose. Au, arbitrary units; LPRx_10, pressure reactivity over 10 min; min, minutes

Table 2, Fig. 3 and Additional file 1: Appendix F show the bolus and continuous infusion agents for the full monitoring time. Though some agents demonstrated a significant *p*-value between the pre- and post-windows, the overall data had limited differences. Bolus noradrenaline had a slight decrease in % time of LPRx over key thresholds (previously indicated impaired/intact autoregulation thresholds), however given the small difference in these results and the fact that the median value was 0, the true impact of any of the investigated drugs on LPRx is likely negligible. Finally, noradrenaline and morphine did appear to be significantly associated with ICP, MAP, and CPP shown in Additional file 1: Appendix F.

**Table 2:** Continuous infusion response

Name	Doses	Mean dose change	% time LPRx_10 > 0			
			Pre-dose	Post-dose	*p* value	Adj *p* value
Dobutamine	1519	Decrease	50 (30–68.4)	51.1 (33.3–68.2)	0.295	1
Dobutamine	1222	Increase	50 (30.8–68.1)	50 (30.8–69.2)	0.462	1
Midazolam	5383	Decrease	50 (31.2–67.9)	50 (31.6–67.6)	0.569	1
Midazolam	5178	Increase	50 (31.2–69)	50 (31.8–67.7)	0.131	1
Morphine	8189	Decrease	50.5 (32.1–68)	50 (33.3–67.9)	0.889	1
Morphine	7931	Increase	50 (31–69.2)	50 (32.1–67.9)	0.362	1
Noradrenaline	53,311	Decrease	50 (32–68)	50 (31–67.9)	0.297	1
Noradrenaline	49,928	Increase	50 (31–68)	50 (31–67.9)	0.00673	0.182
Propofol	16,559	Decrease	50 (32–68)	50 (32.6–68.2)	0.375	1
Propofol	14,898	Increase	50 (32–69)	50 (32–67.9)	0.27	1
Vasopressin	78	Decrease	53.7 (51.7–58.1)	53.3 (51.2–54.7)	0.132	1
Vasopressin	66	Increase	53.5 (42–58.9)	52 (45.3–54.2)	0.372	1

The table demonstrates the median and interquartile range of the pre/pose dose windows as well as the Wilcoxon signed-ranked test between these windows with a p value adjust using the Bonferroni analysis. The “change” indicates if the continuous infusion was increase/decrease dose. Note that although noradrenaline showed significance, given that the median value pre–post dose is 0 there is limited evidence that this is a true significant change. LPRx_10, pressure reactivity over 10 min

#### Subcategorization of the DATA

All of the subcategorization of the data can be found in Additional file 1: Appendix G–N. In general, incremental dose changes or bolus doses demonstrated little influence on LPRx or ICP, regardless of group adjustment. However, when separating the doses based on LPRx_10 pre-dose data, all agents had slightly significant *p* values in the LPRx relationships. Though this may have more to do with patient state rather than the drugs which is confirmed by the fact that for both increases and decreases in continuous agents the result in LPRx remains the same. Moreover, given that when LPRx was indicated to be impaired pre-time window (as LPRx_10 > 0 or 0.35) the dose appeared to reduce LPRx, but when LPRx was indicated to be intact (as LPRx_10 < 0 or 0.35) there was an increase in LPRx.

## Discussion

From this temporally resolved dataset prospectively collected at the Karolinska University Hospital, an exploration into the relationships between various treatment agents and cerebrovascular reactivity was performed. The evaluation involved comparing physiologic responses to both continuous and bolus drug infusions, and thus some important aspects can be highlighted.

First, cerebrovascular reactivity, as measured through LPRx metrics, was not significantly impacted by the various medication agents used. These findings are corroborated by publications out of Leuven and Winnipeg for traditional, high-resolution PRx determination [4, 5]. Furthermore, this work is the first study to evaluate the cerebrovascular reactivity response to a large number of treatment agents. As these agents are employed in guideline-based treatment of moderate/severe TBI, their lack of impact on cerebrovascular reactivity carries significance to future analyses [3]. This work supports that small incremental and bolus dosing of recommended treatment agents, may not need to be accounted for in future studies on cerebrovascular reactivity and individualized physiologic targets derived from cerebrovascular reactivity [4]. Moreover, specifically high doses of noradrenaline were not associated with high LPRx values, which helps validate that in general TBI conditions under current CPP and ICP therapies do not significantly degrade cerebrovascular reactivity. Though it must be acknowledged, that more in-depth work assessing the full physiological impact is required.

Second, the variation in vasopressor drug dosing appears to mediate CPP within desired threshold targets (Fig. 2 and Additional file 1: Appendix A demonstrates stable CPP values despite changes in doses). Targeting CPP is an important aspect of future studies looking to mediate cerebrovascular reactivity through optimal CPP targeting. Many collaborative groups in Europe and Canada focus on PRx targeting through CPP [5, 14, 39, 40], and this was the subject of a phase II randomized control trial [41].

Moreover, the plateau wave of ICP vs increases in doses of vasopressin bears highlighting, as seen in Additional file 1: Appendix A. Vasopressin is used to spare noradrenaline at high doses in septic patients, thus this plateau wave in ICP, despite increases in vasopressin doses, might indicate that there is a threshold of ICP management through vasopressin. This fact may have to do with the ability of vasopressin to impact aquaporin-4 which affects the drug carrying implications for ICP control, given aquaporin-4 has a role in the blood brain barrier and water homeostasis [42–45]. However, this requires further exploration in larger multi-center data sets as it is unclear given the limited numbers in this data set.

Third, cerebrovascular reactivity appears to remain relatively unaffected by sedative agents and changes in dosing. In particular, changes in all sedative agents failed to elicit significant alterations in any LPRx or time spent with LPRx above indicated thresholds during the full monitoring time. Although morphine, propofol and midazolam LOESS curves can be seen related to changes in CPP and ICP at higher doses, this may have to do with a limited number of samples in these regions, or refractory intracranial hypertension (Fig. 1 and Additional file 1: Appendix A). Past literature on propofol and cerebral blood flow in TBI patients has noted that this agent has a limited impact on response [46–51]. Moreover work assessing midazolam found a non-significant response in ICP, cerebral blood flow or partial oxygenation [47, 52, 53]. Thus, given the significant number of doses of midazolam and propofol these results bolster that which has already been documented in past literature, noting the limited influence of sedative agents on cerebrovasculature [21, 22].

Next, though the subcategorization based on LPRx pre-dose did indicate some significance in the resulting LPRx, based on the uniformity of change (whether the drug infusion was increased or decreased in pre-dose cerebrovascular reactivity state), the resulting significance appears to have more to do with patient state then the agent itself. All other categorizations of the individual groups failed to have any significant outliers in terms of physiological response to medication agent.

Moreover, the sub-group breakdown of the data based on the Marshall CT score, found similar results across all injury severity. Thus, highlights that impaired cerebrovascular reactivity occurs in a variation of injury severity, and that current treatment regimens still have limited impact.

All this work highlights two major insights for the future of TBI care. First given that cerebrovascular reactivity has a limited impact from currently used guideline-based pharmacological regimens, means that the interest in mediation of cerebrovascular reactivity through other approaches should be explored. Methods like the optimal CPP, individualized ICP and the optimal depth of sedation focus on using physiological (not pharmacological) mediation to attain optimal cerebrovascular reactivity [28, 54–58]. This focus on pathophysiological mediation may overcome the limitations in current treatments and lead to more personalized targeted treatment.

Next, as LPRx seems to be unassociated with larger time aggregated pharmacological dosing regimens means that current practices in the intensive care unit may be inadequate to effectively target patient state. Most physiological responses are highly volatile with significant variation in response over hours and minutes. Thus, though this work has documented that current regimens of the agents do not significantly impact physiological response, there are always outliers and more momentary effects that are not accounted for. Any future acute TBI care study should have a focus on smaller aggregation of time then the common hourly measures.

### Limitations

Despite the interesting results described above, there are some limitations which deserve highlighting. First, this study is based only on a single-center observational patient cohort taken from Sweden and given the nature of TBI, there is high heterogeneity not accounted for in this study. Thus, the more individualized injury patterns that directly impact cerebral physiological response were not accounted for in this manuscript apart from overall Marshal CT score injury polychotomization. Second, we focused on basic statistics and descriptive analysis for data, the full physiological impact of these agents would be more complete through an individualized moment by moment assessment. Third the drugs investigated were often administered at the same time, and thus the physiologic changes may be suppressed by changes in other infusions. Moreover, given the nature of retrospective observational studies it is impossible to truly determine if the change in drug concentration was a result in a change in cerebral physiology or vice versa. This is confirmed by the high doses of noradrenaline and propofol having confounding associations with ICP and CPP. Finally, given the relatively low resolution of the LPRx and limited overall data collection, this is the first work to compare LPRx to medication.

### Future directions

Future investigation would benefit from time-series analytical methodologies, using multi-variate vector autoregressive integrative moving average (VARIMA) models, impulse response function analysis, and Granger causality testing both pre- and post-agent. Such complex work is the focus of ongoing efforts of both the Winnipeg Acute TBI laboratories and the Karolinska Institutet [5, 14, 39, 40]. In addition, despite the lack of significant results, future sub-group analysis to better determine outliers and physiological differences within patients would be beneficial. To what extent age and sex impact cerebral assessment is still unclear with work indicating that older age results in decreased ICP values [59, 60].

## Conclusions

The results of the analysis confirmed that, overall, the continuous infusion or bolus doses of sedative (propofol, alfentanil, fentanyl, morphine and midazolam) and vasopressor (dobutamine, ephedrine, noradrenaline and vasopressin) agents do not impact or impair cerebrovascular reactivity. However, vasopressor agents do appear to maintain CPP and may be useful to target optimal CPP values. Overall, this study indicates that commonly administered sedative and vasopressor agents do not have a clinically significant influence on cerebrovascular reactivity in TBI. These results are still limited, requiring further temporal investigation.

### Supplementary Information

**Additional file 1: Appendix A.** LOESS Curves – CPP/ICP/LPRx_10/LPRx_60.** Appendix B.** LOESS Curves – MAP/LPRx_15/LPRx_20/LPRx_30.** Appendix C1.** Multiple lnear model for all data.** Appendix C2.** Multiple linear model for sedatives data.** Appendix C3.** Multiple linear model for vasopressor data.** Appendix D.** Multiple linear model for segment Marshall CT score data.** Appendix D1.** Multiple linear model for Marshall CT data = 1.** Appendix D2.** Multiple linear model for Marshall CT data = 2.** Appendix D3.** Multiple linear model for Marshall CT data = 3.** Appendix D5.** Linear model for Marshall CT data = 5.** Appendix E.** One-Way ANOVA of physiology and Marshall CT score.** Appendix F.** Infusions of all data.** Appendix G.** Pre-time window over 50% time ICP > 20 mmHg.** Appendix H.** Pre-time window over 50% time ICP < 20 mmHg.** Appendix I.** Pre-time window over 50% time L-PRx_10 > 0.** Appendix J.** Pre-time window over 50% time L-PRx_10 < 0.** Appendix K.** Pre-time window over 50% time L-PRx_10 > 0.35.** Appendix L.** Pre-time window over 50% time L-PRx_10 < 0.35.** Appendix M.** Continuous infusion going from nothing to agent (and vice versa ie, On to Off).** Appendix N.** Assessing the High/Medium/Low of different infusion agent.** Appendix O.** Histogram distributions of continuous infusion agents.

## Data Availability

All data generated or analyzed during this study are included in this published article [and its additional information files].

## Abbreviations

ABP: Arterial blood pressure
CPP: Cerebral perfusion pressure
GCS: Glasgow Coma Scale
ICP: Intracranial blood pressure
ICU: Intensive care unit
LOESS: Locally estimated scatter plot smoothing
LOWESS: Locally weighted estimate scatter plot smoothing
LPRx: Long pressure reactivity index
MAP: Mean arterial blood pressure
TBI: Traumatic brain injury
VARMA: Vector autoregressive moving average filter
