# Supplementary material for: The impact of sedative and vasopressor agents on cerebrovascular reactivity in severe traumatic brain injury
Source: Intensive Care Med Exp. 2023 Aug 5;11:54. doi: 10.1186/s40635-023-00524-4 (PMC10403459; doi:10.1186/s40635-023-00524-4)

Table of Contents

Contents

[Appendix A. LOESS Curves – CPP/ICP/LPRx_10/LPRx_60 4](#_Toc130384448)

[Appendix B. LOESS Curves – MAP/LPRx_15/LPRx_20/LPRx_30 6](#_Toc130384451)

[Appendix C. Multiple Linear Model for All Data 10](#_Toc130384452)

[Appendix C1. Multiple Linear Model for All Data 10](#_Toc130384453)

[Appendix C2. Multiple Linear Model for Sedatives Data 11](#_Toc130384454)

[Appendix C3. Multiple Linear Model for Vasopressor Data 12](#_Toc130384455)

[Appendix D. Multiple Linear Model for Segment Marshall CT Score Data 13](#_Toc130384456)

[Appendix D1. Multiple Linear Model for Marshall CT Data = 1 13](#_Toc130384457)

[Appendix D2. Multiple Linear Model for Marshall CT Data = 2 16](#_Toc130384458)

[Appendix D3. Multiple Linear Model for Marshall CT Data = 3 18](#_Toc130384459)

[Appendix D5. Linear Model for Marshall CT Data = 5 19](#_Toc130384460)

[Appendix E. One-Way ANOVA of Physiology and Marshall CT Score 21](#_Toc130384461)

[Appendix F. Infusions of All Data 23](#_Toc130384462)

[Appendix G. Pre-time window over 50% time ICP > 20mmHg 32](#_Toc130384463)

[Appendix H. Pre-time window over 50% time ICP < 20 mmHg 40](#_Toc130384464)

[Appendix I. Pre-time window over 50% time L-PRx_10 > 0 49](#_Toc130384465)

[Appendix J. Pre-time window over 50% time L-PRx_10 < 0 58](#_Toc130384466)

[Appendix K. Pre-time window over 50% time L-PRx_10 > 0.35 67](#_Toc130384467)

[Appendix L. Pre-time window over 50% time L-PRx_10 < 0.35 75](#_Toc130384468)

[Appendix M. Continuous infusion going from nothing to agent (and vice versa ie, On to Off) 84](#_Toc130384469)

[Appendix N. Assessing the High/Medium/Low of different infusion agent 89](#_Toc130384470)

[Appendix O. Histogram Distributions of Continuous Infusion Agents 96](#_Toc130384471)

The Appendices F-N compares the pre-post dose change for various indicated physiologies. The time window is taken as the value average over a 30 minute window, with the post-window taken 15 minutes after the infusion change. Each comparison has the p value for a Wilcox Signed ranked test and the Bonferroni adjusted p value. Table F is the table of all the data, and G-N are all different subdivisions of the original cohort.

# Appendix A. LOESS Curves – CPP/ICP/LPRx_10/LPRx_60

#
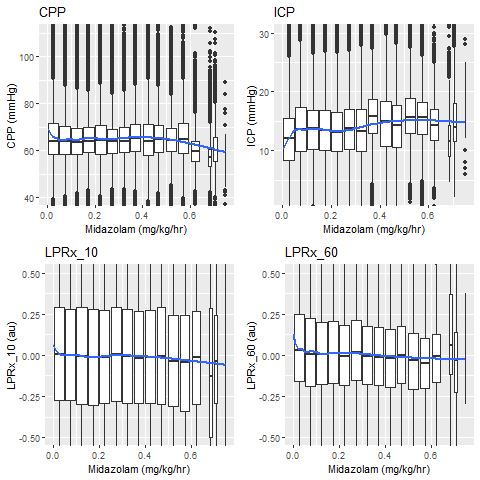

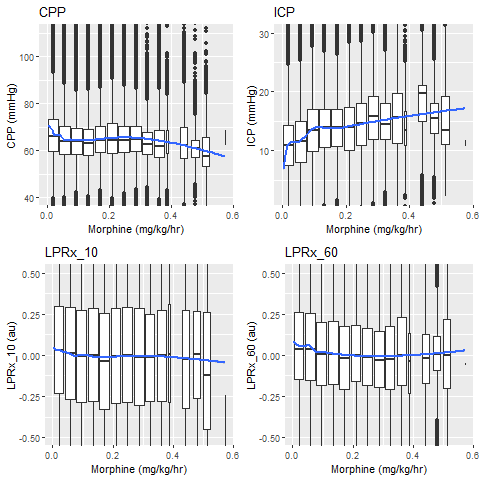


#
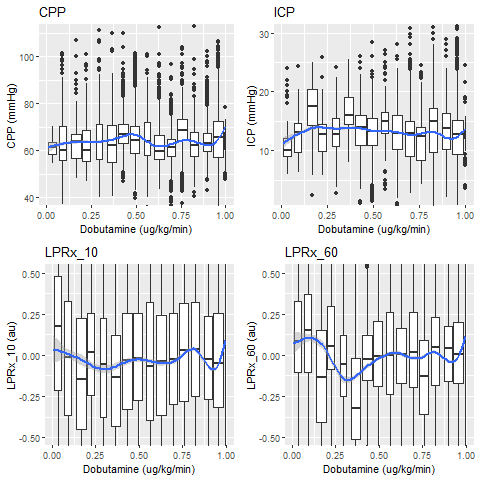

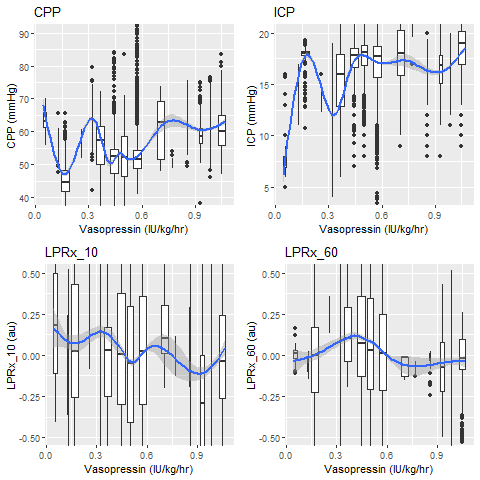


*Figure of the LOESS plots of different dose amounts and different physiological responses, note for these there are limited doses thus the result will not be accurate to the global response. Au, arbitrary units; CPP, cerebral prefusion pressure; hr, hour; ICP, intracranial pressure; IU, infusion units; kg, kilogram; LPRx_10, pressure reactivity over 10 minutes; LPRx_60, pressure reactivity over 60 minutes; mg, milligram; min, minutes; ml, millilitres; mmHg, millimeter of mercury; ug, micrograms;*

# Appendix B. LOESS Curves – MAP/LPRx_15/LPRx_20/LPRx_30


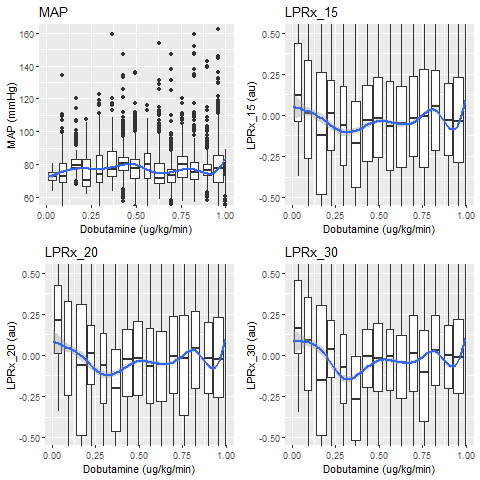


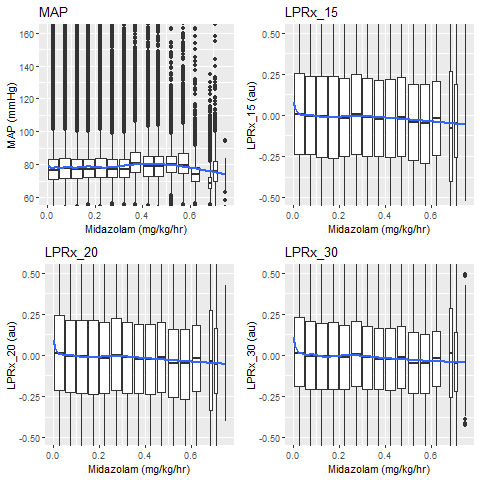

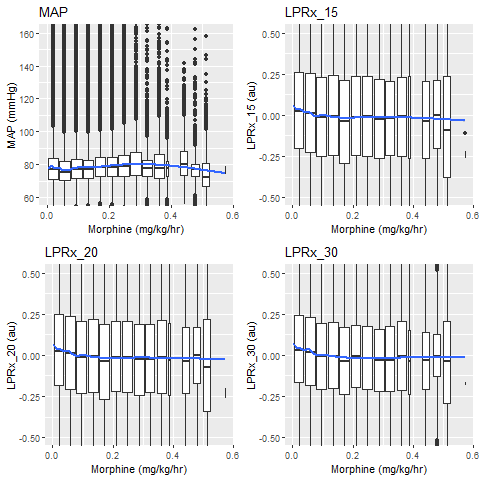

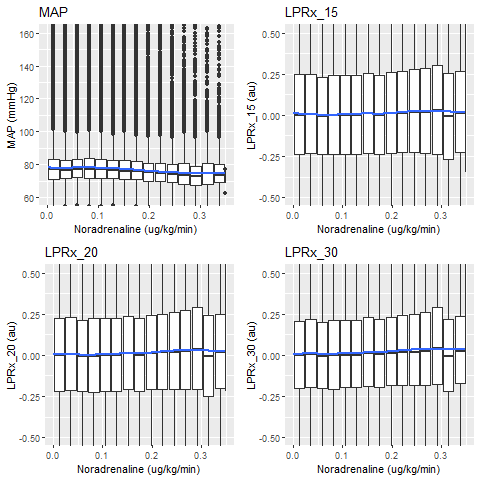

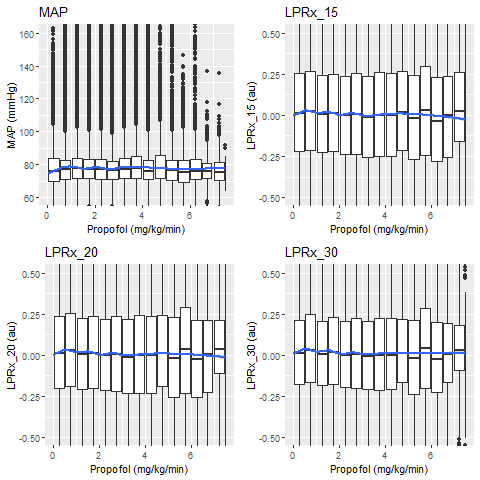

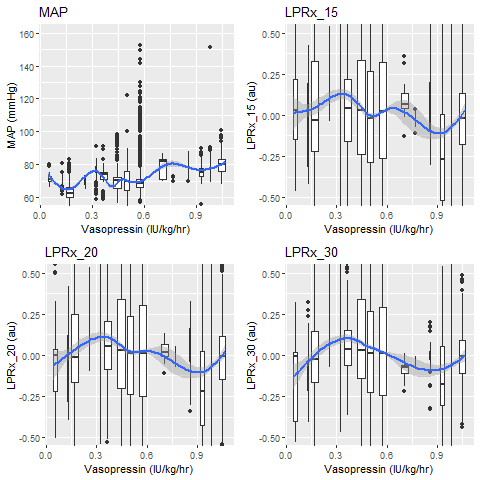


*Figure of the LOESS plots of different dose amounts and different physiological responses, note for these there are limited doses thus the result will not be accurate to the global response. Au, arbitrary units; MAP, mean arterial blood pressure; hr, hour; IU, infusion units; kilogram; LPRx_15, pressure reactivity over 15 minutes; LPRx_20, pressure reactivity over 20 minutes; LPRx_30, pressure reactivity over 30 minutes; mg, milligram; min, minutes; ml, millilitres; mmHg, millimeter of mercury; ug, micrograms;*

# Appendix C. Multiple Linear Model for All Data

All data information used for a multiple linear regression model. For all models physiological variables are the dependent variable and drugs are the independent linear components. The general concept is that independent linear components are used to predict the response variable (physiological variable) through a basic linear regression value between the components. Components that have a low p value and a correlation mean < |0.5| demonstrate a strong linear relationship.

## Appendix C1. Multiple Linear Model for All Data

The linear model for all the data, including times where multiple sedative and vasopressor agents are given at the same time from 431 patients resulting in 3073 days of recording.

| **Physiological Variables** | **Drug** | **Correlation Mean (Standard Deviation)** | **P Value** | **T Value** |
| --- | --- | --- | --- | --- |
| CPP | noradrenaline | 0.000284 (0.000255) | 0.265 | 1.11 |
|  | dobutamine | 0.00813 (0.0852) | 0.924 | 0.0954 |
|  | vasopressin | -0.0456 (0.93) | 0.961 | -0.0491 |
|  | propofol | -0.0144 (0.0129) | 0.265 | -1.12 |
|  | midazolam | -8.02e-05 (0.00402) | 0.984 | -0.02 |
| ICP | noradrenaline | -3.07e-05 (4.69e-05) | 0.512 | -0.656 |
|  | dobutamine | -0.00405 (0.0157) | 0.796 | -0.259 |
|  | vasopressin | -0.018 (0.171) | 0.916 | -0.105 |
|  | propofol | 0.00465 (0.00237) | 0.0495 | 1.96 |
|  | midazolam | 0.000786 (0.000738) | 0.287 | 1.06 |
| LPRx_10 | noradrenaline | 3.39e-06 (4.07e-06) | 0.404 | 0.834 |
|  | dobutamine | -0.000418 (0.00136) | 0.758 | -0.308 |
|  | vasopressin | -0.00253 (0.0148) | 0.865 | -0.17 |
|  | propofol | -7.64e-05 (0.000205) | 0.71 | -0.372 |
|  | midazolam | 6.33e-05 (6.4e-05) | 0.323 | 0.989 |
| LPRx_60 | noradrenaline | 2.19e-07 (1.03e-06) | 0.831 | 0.213 |
|  | dobutamine | 3.61e-05 (0.000344) | 0.916 | 0.105 |
|  | vasopressin | -0.000969 (0.00374) | 0.796 | -0.259 |
|  | propofol | 8.6e-05 (5.21e-05) | 0.0987 | 1.65 |
|  | midazolam | 1.32e-05 (1.62e-05) | 0.414 | 0.818 |

*The table demonstrates the mean and standard deviation of a linear regression model between the drugs and the given physiological variable. Note the close to zero correlation coefficient and the non-significant p values. CPP, cerebral prefusion pressure; ICP, intracranial pressure; LPRx_10, pressure reactivity over 10 minutes; LPRx_60, pressure reactivity over 60 minutes.*

## Appendix C2. Multiple Linear Model for Sedatives Data

The linear model for just sedative data, including only times where just sedative agents are given at the same time from 46 patients resulting in 121 days of recording.

| **Physiological Variables** | **Drug** | **Correlation Mean (Standard Deviation)** | **P Value** | **T Value** |
| --- | --- | --- | --- | --- |
| CPP | propofol | 0.0141 (0.124) | 0.91 | 0.113 |
|  | midazolam | -0.00373 (0.0524) | 0.943 | -0.0712 |
| ICP | propofol | -0.00186 (0.0217) | 0.932 | -0.0858 |
|  | midazolam | 0.00101 (0.00913) | 0.912 | 0.111 |
| LPRx_10 | propofol | 0.000104 (0.00255) | 0.967 | 0.041 |
|  | midazolam | 8.62e-05 (0.00107) | 0.936 | 0.0804 |
| LPRx_60 | propofol | -0.000417 (0.000655) | 0.524 | -0.637 |
|  | midazolam | 0.000217 (0.000276) | 0.431 | 0.788 |

*The table demonstrates the mean and standard deviation of a linear regression model between the drugs and the given physiological variable. Note the close to zero correlation coefficient and the non-significant p values. CPP, cerebral prefusion pressure; ICP, intracranial pressure; LPRx_10, pressure reactivity over 10 minutes; LPRx_60, pressure reactivity over 60 minutes.*

## Appendix C3. Multiple Linear Model for Vasopressor Data

The linear model for just vasopressor data, including only times where just vasopressor agents are given at the same time from 42 patients resulting in 113 days of recording.

| **Physiological Variables** | **Drug** | **Correlation Mean (Standard Deviation)** | **P Value** | **T Value** |
| --- | --- | --- | --- | --- |
| CPP | noradrenaline | 0.00366 (0.00702) | 0.602 | 0.522 |
|  | dobutamine | -0.0976 (0.709) | 0.891 | -0.138 |
| ICP | noradrenaline | 0.00011 (0.00107) | 0.919 | 0.102 |
|  | dobutamine | -0.0169 (0.109) | 0.876 | -0.156 |
| LPRx_10 | noradrenaline | 3.76e-05 (0.000118) | 0.751 | 0.318 |
|  | dobutamine | 0.00122 (0.012) | 0.919 | 0.102 |
| LPRx_60 | noradrenaline | -3.45e-05 (2.9e-05) | 0.234 | -1.19 |
|  | dobutamine | 0.000199 (0.00291) | 0.945 | 0.0684 |

*The table demonstrates the mean and standard deviation of a linear regression model between the drugs and the given physiological variable. Note the close to zero correlation coefficient and the non-significant p values. CPP, cerebral prefusion pressure; ICP, intracranial pressure; LPRx_10, pressure reactivity over 10 minutes; LPRx_60, pressure reactivity over 60 minutes.*

# Appendix D. Multiple Linear Model for Segment Marshall CT Score Data

The segmented for Marshall CT score data. For all models, physiological variables is the dependent variable and drugs are the independent linear components. The general concept is that independent linear components are used to predict the response variable (physiological variable) through a basic linear regression value between the components. Components that have a low p value and a correlation mean < |0.5| demonstrate a strong linear relationship.

## Appendix D1. Multiple Linear Model for Marshall CT Data = 1

The linear model for Marshal CT of 1 data, including times where multiple sedative and vasopressor agents are given at the same time from 117 patients resulting in 793 days of recording.

| **Physiological Variables** | **Drug** | **Correlation Mean (Standard Deviation)** | **P Value** | **T Value** |
| --- | --- | --- | --- | --- |
| CPP | noradrenaline | 0.00119 (0.00111) | 0.284 | 1.07 |
|  | dobutamine | 0.0562 (0.324) | 0.862 | 0.173 |
|  | vasopressin | -0.0205 (0.0275) | 0.456 | -0.745 |
|  | propofol | -0.0023 (0.00823) | 0.78 | -0.279 |
|  | midazolam | -0.000175 (0.000209) | 0.403 | -0.835 |
| ICP | noradrenaline | -0.0161 (0.0613) | 0.792 | -0.263 |
|  | dobutamine | 0.00508 (0.0052) | 0.328 | 0.979 |
|  | vasopressin | 0.00194 (0.00156) | 0.212 | 1.25 |
|  | propofol | 0.00194 (0.00156) | 0.212 | 1.25 |
|  | midazolam | 1.47e-06 (1.75e-05) | 0.933 | 0.084 |
| LPRx_10 | noradrenaline | -0.00158 (0.00512) | 0.758 | -0.308 |
|  | dobutamine | -0.000542 (0.000434) | 0.212 | -1.25 |
|  | vasopressin | 0.000121 (0.00013) | 0.35 | 0.934 |
|  | propofol | 0.000121 (0.00013) | 0.35 | 0.934 |
|  | midazolam | 5.67e-06 (4.4e-06) | 0.197 | 1.29 |
| LPRx_60 | noradrenaline | -0.000112 (0.00129) | 0.931 | -0.0867 |
|  | dobutamine | 2.24e-05 (0.000109) | 0.837 | 0.205 |
|  | vasopressin | 4.31e-05 (3.27e-05) | 0.188 | 1.32 |
|  | propofol | 4.31e-05 (3.27e-05) | 0.188 | 1.32 |
|  | midazolam | 0.00119 (0.00111) | 0.284 | 1.07 |

*The table demonstrates the mean and standard deviation of a linear regression model between the drugs and the given physiological variable. Note the close to zero correlation coefficient and the non-significant p values. CPP, cerebral prefusion pressure; ICP, intracranial pressure; LPRx_10, pressure reactivity over 10 minutes; LPRx_60, pressure reactivity over 60 minutes.*

The linear model for just sedative data and Marshall CT Score of 1, including only times where just sedative agents are given at the same time from 11 patients resulting in 38 days of recording.

| **Physiological Variables** | **Drug** | **Correlation Mean (Standard Deviation)** | **P Value** | **T Value** |
| --- | --- | --- | --- | --- |
| CPP | propofol | -0.0465 (0.186) | 0.803 | -0.25 |
|  | midazolam | 0.0112 (0.142) | 0.937 | 0.0787 |
| ICP | propofol | -0.0159 (0.0493) | 0.746 | -0.323 |
|  | midazolam | -0.00819 (0.0375) | 0.827 | -0.218 |
| LPRx_10 | propofol | 0.00228 (0.00625) | 0.716 | 0.364 |
|  | midazolam | 0.000955 (0.00476) | 0.841 | 0.201 |
| LPRx_60 | propofol | -0.000311 (0.00161) | 0.847 | -0.193 |
|  | midazolam | 0.000886 (0.00125) | 0.478 | 0.709 |

*The table demonstrates the mean and standard deviation of a linear regression model between the drugs and the given physiological variable. Note the close to zero correlation coefficient and the non-significant p values. CPP, cerebral prefusion pressure; ICP, intracranial pressure; LPRx_10, pressure reactivity over 10 minutes; LPRx_60, pressure reactivity over 60 minutes.*

The linear model for just vasopressor data and Marshall CT score of 1, including only times where just vasopressor agents are given at the same time from 10 patients resulting in 43 days of recording.

| **Physiological Variables** | **Drug** | **Correlation Mean (Standard Deviation)** | **P Value** | **T Value** |
| --- | --- | --- | --- | --- |
| CPP | noradrenaline | 0.00438 (0.0139) | 0.752 | 0.315 |
|  | dobutamine | -0.0721 (1.52) | 0.962 | -0.0474 |
| ICP | noradrenaline | 0.000774 (0.00224) | 0.73 | 0.346 |
|  | dobutamine | 0.0557 (0.245) | 0.82 | 0.227 |
| LPRx_10 | noradrenaline | 5.07e-05 (0.00022) | 0.818 | 0.231 |
|  | dobutamine | 0.0061 (0.0241) | 0.8 | 0.253 |
| LPRx_60 | noradrenaline | -3.17e-05 (5.72e-05) | 0.58 | -0.554 |
|  | dobutamine | 8.04e-05 (0.00624) | 0.99 | 0.0129 |

*The table demonstrates the mean and standard deviation of a linear regression model between the drugs and the given physiological variable. Note the close to zero correlation coefficient and the non-significant p values. CPP, cerebral prefusion pressure; ICP, intracranial pressure; LPRx_10, pressure reactivity over 10 minutes; LPRx_60, pressure reactivity over 60 minutes.*

## Appendix D2. Multiple Linear Model for Marshall CT Data = 2

The linear model for Marshal CT of 2 data, including times where multiple sedative and vasopressor agents are given at the same time from 70 patients resulting in 627 days of recording.

| **Physiological Variables** | **Drug** | **Correlation Mean (Standard Deviation)** | **P Value** | **T Value** |
| --- | --- | --- | --- | --- |
| CPP | noradrenaline | 0.000238 (0.000388) | 0.54 | 0.612 |
|  | dobutamine | -0.00966 (0.117) | 0.934 | -0.0826 |
|  | vasopressin | -0.0776 (1.6) | 0.961 | -0.0486 |
|  | propofol | -0.015 (0.0181) | 0.409 | -0.826 |
|  | midazolam | 0.000906 (0.00568) | 0.873 | 0.159 |
| ICP | noradrenaline | -1.95e-05 (7.48e-05) | 0.795 | -0.26 |
|  | dobutamine | -0.00165 (0.0225) | 0.942 | -0.0731 |
|  | vasopressin | 0.0296 (0.308) | 0.923 | 0.096 |
|  | propofol | 0.00708 (0.00349) | 0.0428 | 2.03 |
|  | midazolam | 0.000737 (0.0011) | 0.501 | 0.673 |
| LPRx_10 | noradrenaline | 6.36e-06 (6.22e-06) | 0.307 | 1.02 |
|  | dobutamine | -0.000293 (0.00187) | 0.875 | -0.157 |
|  | vasopressin | -0.000222 (0.0256) | 0.993 | -0.00867 |
|  | propofol | 7.7e-05 (0.00029) | 0.791 | 0.265 |
|  | midazolam | 3.98e-05 (9.1e-05) | 0.662 | 0.437 |
| LPRx_60 | noradrenaline | -1.6e-06 (1.56e-06) | 0.305 | -1.03 |
|  | dobutamine | 0.000153 (0.000469) | 0.744 | 0.327 |
|  | vasopressin | -0.00188 (0.00641) | 0.77 | -0.293 |
|  | propofol | 0.000167 (7.3e-05) | 0.0222 | 2.29 |
|  | midazolam | 1.02e-05 (2.29e-05) | 0.655 | 0.446 |

*The table demonstrates the mean and standard deviation of a linear regression model between the drugs and the given physiological variable. Note the close to zero correlation coefficient and the non-significant p values. CPP, cerebral prefusion pressure; ICP, intracranial pressure; LPRx_10, pressure reactivity over 10 minutes; LPRx_60, pressure reactivity over 60 minutes.*

The linear model for just sedative data and Marshall CT Score of 2, including only times where just sedative agents are given at the same time from 2 patients resulting in 12 days of recording.

| **Physiological Variables** | **Drug** | **Correlation Mean (Standard Deviation)** | **P Value** | **T Value** |
| --- | --- | --- | --- | --- |
| CPP | propofol | 0.0504 (0.215) | 0.815 | 0.234 |
|  | midazolam | -0.00501 (0.0703) | 0.943 | -0.0712 |
| ICP | propofol | -0.00344 (0.0245) | 0.888 | -0.14 |
|  | midazolam | 0.000613 (0.008) | 0.939 | 0.0766 |
| LPRx_10 | propofol | -0.00107 (0.00381) | 0.779 | -0.281 |
|  | midazolam | 0.000132 (0.00125) | 0.916 | 0.106 |
| LPRx_60 | propofol | -0.00049 (0.000988) | 0.62 | -0.495 |
|  | midazolam | 0.000196 (0.000323) | 0.544 | 0.606 |

*The table demonstrates the mean and standard deviation of a linear regression model between the drugs and the given physiological variable. Note the close to zero correlation coefficient and the non-significant p values. CPP, cerebral prefusion pressure; ICP, intracranial pressure; LPRx_10, pressure reactivity over 10 minutes; LPRx_60, pressure reactivity over 60 minutes.*

## Appendix D3. Multiple Linear Model for Marshall CT Data = 3

The linear model for Marshal CT of 3 data, including times where multiple sedative and vasopressor agents are given at the same time from 15 patients resulting in 118 days of recording.

| **Physiological Variables** | **Drug** | **Correlation Mean (Standard Deviation)** | **P Value** | **T Value** |
| --- | --- | --- | --- | --- |
| CPP | noradrenaline | 0.00323 (0.00497) | 0.516 | 0.65 |
|  | dobutamine | -0.0645 (1.22) | 0.958 | -0.053 |
|  | vasopressin | -0.0942 (2.52) | 0.97 | -0.0373 |
|  | propofol | -0.0296 (0.107) | 0.783 | -0.276 |
|  | midazolam | 9.37e-05 (0.0609) | 0.999 | 0.00154 |
| ICP | noradrenaline | 0.000135 (0.00075) | 0.857 | 0.18 |
|  | dobutamine | 0.00919 (0.184) | 0.96 | 0.05 |
|  | vasopressin | -0.0793 (0.381) | 0.835 | -0.208 |
|  | propofol | -0.00509 (0.0162) | 0.753 | -0.314 |
|  | midazolam | 0.00118 (0.0092) | 0.898 | 0.128 |
| LPRx_10 | noradrenaline | -7.49e-07 (7.73e-05) | 0.992 | -0.00969 |
|  | dobutamine | 0.00875 (0.019) | 0.644 | 0.462 |
|  | vasopressin | 0.00154 (0.0393) | 0.969 | 0.0393 |
|  | propofol | -0.000318 (0.00167) | 0.849 | -0.191 |
|  | midazolam | 0.000478 (0.000949) | 0.615 | 0.503 |
| LPRx_60 | noradrenaline | 8.49e-06 (2e-05) | 0.671 | 0.425 |
|  | dobutamine | -0.000478 (0.0049) | 0.922 | -0.0976 |
|  | vasopressin | -0.000338 (0.0101) | 0.973 | -0.0334 |
|  | propofol | -3.73e-05 (0.000433) | 0.931 | -0.0862 |
|  | midazolam | 6.87e-05 (0.000245) | 0.779 | 0.28 |

*The table demonstrates the mean and standard deviation of a linear regression model between the drugs and the given physiological variable. Note the close to zero correlation coefficient and the non-significant p values. CPP, cerebral prefusion pressure; ICP, intracranial pressure; LPRx_10, pressure reactivity over 10 minutes; LPRx_60, pressure reactivity over 60 minutes.*

## Appendix D5. Linear Model for Marshall CT Data = 5

The linear model for Marshal CT of 5 data, including times where multiple sedative and vasopressor agents are given at the same time from 229 patients resulting in 1534 days of recording.

| **Physiological Variables** | **Drug** | **Correlation Mean (Standard Deviation)** | **P Value** | **T Value** |
| --- | --- | --- | --- | --- |
| CPP | noradrenaline | 0.000206 (0.000354) | 0.56 | 0.583 |
|  | dobutamine | 0.0225 (0.138) | 0.87 | 0.163 |
|  | vasopressin | -0.0296 (1.28) | 0.982 | -0.0231 |
|  | propofol | -0.0106 (0.026) | 0.683 | -0.408 |
|  | midazolam | 0.00031 (0.00843) | 0.971 | 0.0368 |
| ICP | noradrenaline | -2.04e-05 (6.36e-05) | 0.748 | -0.321 |
|  | dobutamine | -0.00485 (0.0247) | 0.844 | -0.196 |
|  | vasopressin | -0.0383 (0.23) | 0.868 | -0.166 |
|  | propofol | 0.000519 (0.00468) | 0.912 | 0.111 |
|  | midazolam | -0.000362 (0.00151) | 0.811 | -0.239 |
| LPRx_10 | noradrenaline | 1.18e-06 (5.67e-06) | 0.835 | 0.208 |
|  | dobutamine | -0.000304 (0.0022) | 0.891 | -0.138 |
|  | vasopressin | -0.00493 (0.0205) | 0.81 | -0.24 |
|  | propofol | 9.44e-05 (0.000417) | 0.821 | 0.226 |
|  | midazolam | 3.92e-05 (0.000135) | 0.772 | 0.29 |
| LPRx_60 | noradrenaline | 1.29e-06 (1.44e-06) | 0.371 | 0.895 |
|  | dobutamine | -6.23e-05 (0.000561) | 0.911 | -0.111 |
|  | vasopressin | -0.000646 (0.00521) | 0.901 | -0.124 |
|  | propofol | -2.71e-05 (0.000107) | 0.799 | -0.254 |
|  | midazolam | -1.47e-05 (3.43e-05) | 0.667 | -0.43 |

*The table demonstrates the mean and standard deviation of a linear regression model between the drugs and the given physiological variable. Note the close to zero correlation coefficient and the non-significant p values. CPP, cerebral prefusion pressure; ICP, intracranial pressure; LPRx_10, pressure reactivity over 10 minutes; LPRx_60, pressure reactivity over 60 minutes.*

The linear model for just sedative data and Marshall CT Score of 5, including only times where just sedative agents are given at the same time from 33 patients resulting in 70 days of recording.

| **Physiological Variables** | **Drug** | **Correlation Mean (Standard Deviation)** | **P Value** | **T Value** |
| --- | --- | --- | --- | --- |
| CPP | propofol | -0.0325 (0.418) | 0.938 | -0.0778 |
|  | midazolam | -0.0171 (0.336) | 0.959 | -0.0508 |
| ICP | propofol | 0.00927 (0.111) | 0.934 | 0.0834 |
|  | midazolam | 0.00316 (0.0895) | 0.972 | 0.0354 |
| LPRx_10 | propofol | 0.00463 (0.0098) | 0.637 | 0.472 |
|  | midazolam | -0.00229 (0.00789) | 0.772 | -0.29 |
| LPRx_60 | propofol | 0.000538 (0.0025) | 0.83 | 0.215 |
|  | midazolam | -0.000885 (0.00201) | 0.66 | -0.44 |

*The table demonstrates the mean and standard deviation of a linear regression model between the drugs and the given physiological variable. Note the close to zero correlation coefficient and the non-significant p values. CPP, cerebral prefusion pressure; ICP, intracranial pressure; LPRx_10, pressure reactivity over 10 minutes; LPRx_60, pressure reactivity over 60 minutes.*

The linear model for just vasopressor data and Marshall CT score of 5, including only times where just vasopressor agents are given at the same time from 42 patients resulting in 69 days of recording.

| **Physiological Variables** | **Drug** | **Correlation Mean (Standard Deviation)** | **P Value** | **T Value** |
| --- | --- | --- | --- | --- |
| CPP | noradrenaline | 0.00352 (0.00872) | 0.686 | 0.404 |
|  | dobutamine | -0.0977 (0.831) | 0.906 | -0.118 |
| ICP | noradrenaline | -0.000107 (0.00126) | 0.932 | -0.0851 |
|  | dobutamine | -0.0299 (0.12) | 0.804 | -0.249 |
| LPRx_10 | noradrenaline | 5.45e-05 (0.00015) | 0.717 | 0.363 |
|  | dobutamine | -0.000709 (0.0143) | 0.961 | -0.0495 |
| LPRx_60 | noradrenaline | -3.31e-05 (3.56e-05) | 0.353 | -0.93 |
|  | dobutamine | 0.000196 (0.00338) | 0.954 | 0.0582 |

*The table demonstrates the mean and standard deviation of a linear regression model between the drugs and the given physiological variable. Note the close to zero correlation coefficient and the non-significant p values. CPP, cerebral prefusion pressure; ICP, intracranial pressure; LPRx_10, pressure reactivity over 10 minutes; LPRx_60, pressure reactivity over 60 minutes.*

# Appendix E. One-Way ANOVA of Physiology and Marshall CT Score

The table is the one-way ANOVA test between different variables and Marshall CT Scores over the whole populations.

| **Variable** | **Marshall CT Score (Mean and Standard Deviation / Number of Patients)** | | | | **P Value** | **P Value Adjusted** |
| --- | --- | --- | --- | --- | --- | --- |
|  | 1 | 2 | 3 | 5 |  |  |
| ICP (mmHg) | 10.3 (3.9) | 12.6 (4.86) | 13.4 (4.26) | 12.3 (4.91) | 0.000176 | 0.0703 |
| CPP (mmHg) | 70.6 (6.61) | 68.4 (9.43) | 65.4 (6.68) | 69.4 (7.45) | 0.0299 | 1 |
| LPRx_10 (au) | 0.0241 (0.064) | 0.0334 (0.0761) | 0.043 (0.0919) | 0.0347 (0.0728) | 0.518 | 1 |
| LPRx_60 (au) | 0.0565 (0.089) | 0.0578 (0.0882) | 0.0805 (0.14) | 0.0503 (0.103) | 0.785 | 1 |
| Age | 44.7 (17.7) | 47.8 (18.1) | 43.6 (18.8) | 51.5 (16.5) | 0.00481 | 1 |
| GCS Eye | 1.59 (1.03) | 2.03 (1.23) | 1.77 (1.09) | 1.61 (0.971) | 0.0824 | 1 |
| GCS Motor | 3.37 (1.76) | 3.85 (1.72) | 3.77 (1.64) | 3.52 (1.76) | 0.333 | 1 |
| GCS Verbal | 1.64 (1.11) | 1.93 (1.25) | 1.77 (1.36) | 1.66 (1.07) | 0.441 | 1 |
| GCS | 6.7 (3.38) | 7.92 (3.57) | 7.67 (3.54) | 7.03 (3.33) | 0.118 | 1 |
| Sex (male) | 96 (71.6%) | 67 (75.3%) | 12 (46.2%) | 190 (70.9%) | 0.12 | 1 |
| Hypoxia | 35 (26.1%) | 18 (20.2%) | 2 (7.69%) | 42 (15.7%) | 0.134 | 1 |
| Hypotension | 7 (5.22%) | 5 (5.62%) | 0 (0%) | 4 (1.49%) | - | - |
| SAH | 113 (84.3%) | 77 (86.5%) | 15 (57.7%) | 180 (67.2%) | - | - |
| EDH | 12 (8.96%) | 10 (11.2%) | 1 (3.85%) | 47 (17.5%) | 0.0896 | 1 |
| GOS | 3.42 (1.17) | 3.24 (1.31) | 3.47 (1.51) | 3.16 (1.3) | 0.247 | 1 |
| ICU LoS | 10.7 (5.69) | 12.1 (6.56) | 10.3 (5.97) | 9.72 (6.24) | 0.0482 | 1 |
| **Pupils** |  |  |  |  |  |  |
| Both Reactive | 97 (72.4%) | 54 (60.7%) | 11 (42.3%) | 168 (62.7%) | 0.0314 | 1 |
| One Reactive | 12 (8.96%) | 5 (5.62%) | 1 (3.85%) | 27 (10.1%) |  |  |
| None Reactive | 12 (8.96%) | 13 (14.6%) | 1 (3.85%) | 51 (19%) |  |  |

*The table demonstrates the mean and standard deviation of the variables and the Marshal CT score. One way ANOVA was preformed for these variables. Note in this patient population no patient had a Marshal CT score of 4. ANOVA, analysis of variance; au, arbitrary units; CPP, cerebral prefusion pressure; EDH, epidural hematoma; GCS, Glasgow coma score; GOS, Glasgow outcome score (~12 months); ICP, intracranial pressure; ICU, intensive care unit; LoS, length of stay; LPRx_10, pressure reactivity over 10 minutes; LPRx_60, pressure reactivity over 60 minutes; mmHg, millimeters of mercury; SDH, subdural hematoma.*

# Appendix F. Infusions of All Data

The table contains all infusions given within this cohort, separated into the continuous infusion doses then the bolus doses.

| **Continuous Intravenous** | | | | | | | | | | | | | | |
| --- | --- | --- | --- | --- | --- | --- | --- | --- | --- | --- | --- | --- | --- | --- |
| **Name** | **Doses** | **Mean Dose Change** | **Mean MAP** | | | | **Mean CPP** | | | | **% time CPP>60** | | | |
|  |  |  | **Pre Dose** | **Post Dose** | **P value** | **Adj P Value** | **Pre Dose** | **Post Dose** | **P value** | **Adj P Value** | **Pre Dose** | **Post Dose** | **P value** | **Adj P Value** |
| Dobutamine | 1519 | Decrease | 76.7 (71-83.9) | 76 (70.8-82.8) | 0.0629 | 1 | 64.4 (58.5-70.4) | 63.2 (58.8-69.2) | 0.0376 | 1 | 14.3 (0-73) | 17.4 (0-72.6) | 0.136 | 1 |
| Dobutamine | 1222 | Increase | 73.4 (68.1-79.8) | 72.8 (68.2-79.5) | 0.398 | 1 | 60 (54.2-66.3) | 59.7 (54.3-65.4) | 0.588 | 1 | 59.1 (10.4-100) | 60.7 (7.69-100) | 0.866 | 1 |
| Midazolam | 5383 | Decrease | 78.4 (73.2-84.5) | 78.2 (73.1-84.4) | 0.326 | 1 | 65.2 (59.8-71.2) | 64.9 (59.8-71) | 0.574 | 1 | 9.09 (0-56) | 10.3 (0-57.1) | 0.695 | 1 |
| Midazolam | 5178 | Increase | 78.8 (73.5-85.1) | 78.5 (73.2-84.7) | 0.0272 | 0.733 | 64.5 (59.1-71) | 64.5 (59.2-70.7) | 0.615 | 1 | 12.5 (0-63.6) | 11.1 (0-63) | 0.282 | 1 |
| Morphine | 8189 | Decrease | 77.8 (72.5-84.2) | 77.5 (72.2-83.8) | 0.0978 | 1 | 65.5 (59.8-71.7) | 65.2 (59.6-71.5) | 0.107 | 1 | 7.78 (0-55.6) | 9.68 (0-58.6) | 0.221 | 1 |
| Morphine | 7931 | Increase | 78.2 (72.7-84.9) | 77.5 (72.1-83.9) | <0.0001 | <0.0001 | 65.5 (59.7-72) | 64.9 (59.3-71.2) | 0.000165 | 0.00447 | 8.49 (0-58.3) | 10.3 (0-63) | 0.147 | 1 |
| Noradrenaline | 53311 | Decrease | 79 (73.5-85.4) | 78.4 (73.1-84.7) | <0.0001 | <0.0001 | 66.2 (60.5-72.7) | 65.6 (60.3-71.9) | <0.0001 | <0.0001 | 7.14 (0-46.4) | 7.41 (0-50) | 0.00946 | 0.256 |
| Noradrenaline | 49928 | Increase | 76.5 (71.1-82.9) | 76.5 (71.2-82.9) | 0.157 | 1 | 63.2 (57.9-69.4) | 63.6 (58.3-69.8) | <0.0001 | <0.0001 | 25.6 (0-77.8) | 18.5 (0-75) | <0.0001 | <0.0001 |
| Propofol | 16559 | Decrease | 77.7 (72.1-84.2) | 77.4 (72-83.9) | 0.0485 | 1 | 65.3 (59.6-71.8) | 65 (59.4-71.6) | 0.0177 | 0.478 | 10 (0-58.3) | 10.7 (0-60.7) | 0.177 | 1 |
| Propofol | 14898 | Increase | 78.7 (73-85.5) | 77.6 (72.1-84.1) | <0.0001 | <0.0001 | 65.9 (60-72.6) | 65.1 (59.6-71.7) | <0.0001 | <0.0001 | 8 (0-53.8) | 10.3 (0-58.1) | 0.000857 | 0.0231 |
| Vasopressin | 78 | Decrease | 81.3 (73.7-84.1) | 80.1 (71.9-82.7) | 0.0872 | 1 | 57.5 (55.2-59.8) | 58.7 (55.6-59.6) | 0.936 | 1 | 66 (58.9-78.6) | 60.8 (55.3-96.8) | 0.884 | 1 |
| Vasopressin | 66 | Increase | 75 (70.6-82.7) | 77.1 (70.7-82.4) | 0.639 | 1 | 55.3 (51-57.5) | 56.4 (51.3-59) | 0.217 | 1 | 91.5 (66-100) | 76.3 (60.8-100) | 0.352 | 1 |
| **Name** | **Mean Dose Change** | **% time CPP>70** | | | | **Mean ICP** | | | | **% time ICP>20** | | | |  |
|  |  | **Pre Dose** | **Post Dose** | **P value** | **Adj P Value** | **Pre Dose** | **Post Dose** | **P value** | **Adj P Value** | **Pre Dose** | **Post Dose** | **P value** | **Adj P Value** |  |
| Dobutamine | Decrease | 4.44 (0-46.2) | 3.33 (0-33.3) | 0.00066 | 0.0178 | 13.6 (9.66-16.7) | 13.4 (9.93-16.3) | 0.323 | 1 | 0 (0-4.35) | 0 (0-2) | <0.0001 | 0.00178 |  |
| Dobutamine | Increase | 0 (0-19.2) | 0 (0-11.5) | 0.0737 | 1 | 14.4 (10.7-17.5) | 14.3 (10.7-17) | 0.409 | 1 | 0 (0-10.3) | 0 (0-5.67) | 0.0019 | 0.0513 |  |
| Midazolam | Decrease | 7.14 (0-55.6) | 6.9 (0-56) | 0.273 | 1 | 13.5 (10-16.7) | 13.4 (9.87-16.5) | 0.293 | 1 | 0 (0-4.92) | 0 (0-3.85) | 0.207 | 1 |  |
| Midazolam | Increase | 6.67 (0-53.6) | 4.55 (0-51.9) | 0.0606 | 1 | 14.4 (10.8-17.7) | 14.2 (10.6-17.3) | 0.00651 | 0.176 | 0 (0-10.8) | 0 (0-7.14) | <0.0001 | <0.0001 |  |
| Morphine | Decrease | 8.33 (0-60.7) | 7.41 (0-60) | 0.0697 | 1 | 12.4 (8.79-15.9) | 12.4 (8.83-15.8) | 0.899 | 1 | 0 (0-3.45) | 0 (0-3.12) | 0.37 | 1 |  |
| Morphine | Increase | 9.32 (0-62.5) | 6.9 (0-57.1) | <0.0001 | <0.0001 | 12.9 (9.16-16.5) | 12.7 (8.89-16.2) | 0.00537 | 0.145 | 0 (0-4.35) | 0 (0-3.57) | <0.0001 | <0.0001 |  |
| Noradrenaline | Decrease | 13 (0-69.7) | 8.33 (0-64.3) | <0.0001 | <0.0001 | 12.9 (9.34-16.3) | 12.9 (9.37-16.1) | 0.0153 | 0.413 | 0 (0-4) | 0 (0-3.45) | <0.0001 | <0.0001 |  |
| Noradrenaline | Increase | 4 (0-38.5) | 3.7 (0-42.9) | 0.00219 | 0.059 | 13.5 (9.87-16.9) | 13.2 (9.45-16.5) | <0.0001 | <0.0001 | 0 (0-7.14) | 0 (0-3.7) | <0.0001 | <0.0001 |  |
| Propofol | Decrease | 7.69 (0-60.7) | 7.14 (0-60) | 0.00286 | 0.0773 | 12.4 (8.64-16) | 12.5 (8.64-16.1) | 0.772 | 1 | 0 (0-3.7) | 0 (0-3.57) | 0.214 | 1 |  |
| Propofol | Increase | 11.1 (0-67.9) | 7.41 (0-60.7) | <0.0001 | <0.0001 | 12.9 (9.02-16.6) | 12.6 (8.73-16.2) | <0.0001 | 0.000196 | 0 (0-6.9) | 0 (0-3.57) | <0.0001 | <0.0001 |  |
| Vasopressin | Decrease | 6.14 (0-6.81) | 5.53 (0-6.74) | 0.0125 | 0.337 | 15.2 (13.9-17.1) | 14 (12.8-16.9) | 0.137 | 1 | 7.22 (0-11.5) | 6.07 (0-8.87) | 0.343 | 1 |  |
| Vasopressin | Increase | 0 (0-6.74) | 0 (0-6.74) | 0.694 | 1 | 16.6 (15.1-18.8) | 16.9 (13.9-18.7) | 0.698 | 1 | 8.6 (0-12.9) | 7.22 (0.806-13.7) | 0.934 | 1 |  |
| **Name** | **Mean Dose Change** | **% time ICP>22** | | | | **Mean LPRx_10** | | | | **% time LPRx_10 > 0** | | | |  |
|  |  | **Pre Dose** | **Post Dose** | **P value** | **Adj P Value** | **Pre Dose** | **Post Dose** | **P value** | **Adj P Value** | **Pre Dose** | **Post Dose** | **P value** | **Adj P Value** |  |
| Dobutamine | Decrease | 0 (0-0) | 0 (0-0) | 0.000161 | 0.00436 | 0.0088 (-0.157-0.166) | 0.0207 (-0.139-0.156) | 0.47 | 1 | 50 (30-68.4) | 51.1 (33.3-68.2) | 0.295 | 1 |  |
| Dobutamine | Increase | 0 (0-0) | 0 (0-0) | 0.0204 | 0.55 | 0.00125 (-0.165-0.152) | 0.0141 (-0.151-0.16) | 0.291 | 1 | 50 (30.8-68.1) | 50 (30.8-69.2) | 0.462 | 1 |  |
| Midazolam | Decrease | 0 (0-0) | 0 (0-0) | 0.0855 | 1 | 0.00808 (-0.157-0.156) | 0.00547 (-0.156-0.155) | 0.616 | 1 | 50 (31.2-67.9) | 50 (31.6-67.6) | 0.569 | 1 |  |
| Midazolam | Increase | 0 (0-3.45) | 0 (0-0) | <0.0001 | <0.0001 | 0.0122 (-0.15-0.163) | 0.00256 (-0.159-0.15) | 0.0294 | 0.795 | 50 (31.2-69) | 50 (31.8-67.7) | 0.131 | 1 |  |
| Morphine | Decrease | 0 (0-0) | 0 (0-0) | 0.437 | 1 | 0.00862 (-0.145-0.156) | 0.00809 (-0.141-0.153) | 0.669 | 1 | 50.5 (32.1-68) | 50 (33.3-67.9) | 0.889 | 1 |  |
| Morphine | Increase | 0 (0-0) | 0 (0-0) | <0.0001 | <0.0001 | 0.00701 (-0.15-0.162) | 0.00302 (-0.153-0.155) | 0.168 | 1 | 50 (31-69.2) | 50 (32.1-67.9) | 0.362 | 1 |  |
| Noradrenaline | Decrease | 0 (0-0) | 0 (0-0) | <0.0001 | <0.0001 | 0.0057 (-0.152-0.155) | 0.005 (-0.154-0.153) | 0.109 | 1 | 50 (32-68) | 50 (31-67.9) | 0.297 | 1 |  |
| Noradrenaline | Increase | 0 (0-0) | 0 (0-0) | <0.0001 | <0.0001 | 0.0062 (-0.154-0.158) | 0.00152 (-0.158-0.154) | 0.00108 | 0.0292 | 50 (31-68) | 50 (31-67.9) | 0.00673 | 0.182 |  |
| Propofol | Decrease | 0 (0-0) | 0 (0-0) | 0.0338 | 0.913 | 0.00546 (-0.15-0.157) | 0.0081 (-0.146-0.158) | 0.27 | 1 | 50 (32-68) | 50 (32.6-68.2) | 0.375 | 1 |  |
| Propofol | Increase | 0 (0-0) | 0 (0-0) | <0.0001 | <0.0001 | 0.00733 (-0.147-0.158) | 0.00373 (-0.154-0.155) | 0.0954 | 1 | 50 (32-69) | 50 (32-67.9) | 0.27 | 1 |  |
| Vasopressin | Decrease | 1.07 (0-1.7) | 0.902 (0-1.3) | 0.443 | 1 | 0.0326 (0.0108-0.117) | 0.0188 (0.00906-0.0533) | 0.0112 | 0.301 | 53.7 (51.7-58.1) | 53.3 (51.2-54.7) | 0.132 | 1 |  |
| Vasopressin | Increase | 1.3 (0-2.32) | 1.19 (0-2.95) | 0.874 | 1 | 0.0269 (-0.0487-0.0583) | 0.0188 (-0.01-0.0401) | 0.658 | 1 | 53.5 (42-58.9) | 52 (45.3-54.2) | 0.372 | 1 |  |
| **Name** | **Mean Dose Change** | **% time LPRx_10 > 0.25** | | | | **% time LPRx_10 > 0.35** | | | | **Mean LPRx_15** | | | |  |
|  |  | **Pre Dose** | **Post Dose** | **P value** | **Adj P Value** | **Pre Dose** | **Post Dose** | **P value** | **Adj P Value** | **Pre Dose** | **Post Dose** | **P value** | **Adj P Value** |  |
| Dobutamine | Decrease | 25.9 (8.89-44.2) | 26.9 (10-42.9) | 0.472 | 1 | 16.7 (3.7-34.6) | 18.5 (3.7-34.5) | 0.625 | 1 | -0.00158 (-0.16-0.157) | 0.000162 (-0.155-0.153) | 0.791 | 1 |  |
| Dobutamine | Increase | 25 (10.1-40.7) | 26.1 (11.1-42.3) | 0.149 | 1 | 17.2 (3.7-32) | 17.2 (4-33.3) | 0.38 | 1 | -0.00389 (-0.162-0.142) | 0.00381 (-0.158-0.154) | 0.691 | 1 |  |
| Midazolam | Decrease | 25 (8.7-42.9) | 25 (8-42.3) | 0.426 | 1 | 16.7 (3.7-33.3) | 16.2 (3.57-33.3) | 0.326 | 1 | 0.00168 (-0.141-0.139) | -0.00181 (-0.148-0.138) | 0.406 | 1 |  |
| Midazolam | Increase | 25 (8.7-42.9) | 25 (8-41.9) | 0.133 | 1 | 16.7 (3.57-33.3) | 15.8 (3.57-33.3) | 0.142 | 1 | 0.0103 (-0.142-0.144) | -0.00238 (-0.152-0.135) | 0.0157 | 0.425 |  |
| Morphine | Decrease | 25 (10-41.9) | 25.8 (10.3-42.3) | 0.798 | 1 | 16.7 (3.7-32.4) | 16.7 (3.7-33.3) | 0.783 | 1 | 0.00652 (-0.136-0.144) | 0.0061 (-0.134-0.148) | 0.448 | 1 |  |
| Morphine | Increase | 25 (8-42.9) | 25 (9.3-42.3) | 0.612 | 1 | 15.4 (3.45-33.3) | 16.1 (3.57-32.5) | 0.226 | 1 | 0.00737 (-0.14-0.15) | 0.00327 (-0.14-0.145) | 0.195 | 1 |  |
| Noradrenaline | Decrease | 25 (8.7-42.3) | 25 (8.7-42.2) | 0.476 | 1 | 16 (3.57-33.3) | 16.1 (3.57-32.3) | 0.375 | 1 | 0.000257 (-0.147-0.143) | -0.000999 (-0.149-0.137) | 0.0278 | 0.751 |  |
| Noradrenaline | Increase | 24.4 (8.33-42.3) | 24.1 (9.3-42.3) | 0.928 | 1 | 16 (3.57-33.3) | 16 (3.57-33.3) | 0.849 | 1 | 0.002 (-0.146-0.145) | -0.00634 (-0.154-0.133) | <0.0001 | <0.0001 |  |
| Propofol | Decrease | 25 (10-42.9) | 25 (10.3-42.9) | 0.287 | 1 | 16.2 (3.7-33.3) | 16.7 (3.7-33.3) | 0.152 | 1 | 0.00722 (-0.142-0.15) | 0.0093 (-0.136-0.154) | 0.0643 | 1 |  |
| Propofol | Increase | 25 (9.68-42.9) | 25 (10-42.3) | 0.771 | 1 | 16.1 (3.57-33.3) | 16.7 (3.7-33.3) | 0.508 | 1 | 0.00743 (-0.14-0.153) | 0.00509 (-0.142-0.147) | 0.292 | 1 |  |
| Vasopressin | Decrease | 30.5 (28.3-36.9) | 29.6 (27.8-30) | 0.00298 | 0.0806 | 23.4 (21.1-32.3) | 22.3 (19.4-23) | 0.00232 | 0.0627 | 0.0375 (0.017-0.0995) | 0.0248 (0.017-0.0446) | 0.0743 | 1 |  |
| Vasopressin | Increase | 29.8 (14.3-32.5) | 28.3 (16.6-30.6) | 0.614 | 1 | 23.2 (10.8-26.6) | 21.1 (11.3-24.1) | 0.504 | 1 | 0.0251 (-0.0214-0.0687) | 0.0171 (-0.0528-0.0489) | 0.439 | 1 |  |
| **Name** | **Mean Dose Change** | **% time LPRx_15 > 0** | | | | **% time LPRx_15 > 0.25** | | | | **% time LPRx_15 > 0.35** | | | |  |
|  |  | **Pre Dose** | **Post Dose** | **P value** | **Adj P Value** | **Pre Dose** | **Post Dose** | **P value** | **Adj P Value** | **Pre Dose** | **Post Dose** | **P value** | **Adj P Value** |  |
| Dobutamine | Decrease | 50 (27.1-70.4) | 50 (28-70) | 0.743 | 1 | 20.7 (3.57-40.7) | 20.8 (3.64-41.4) | 0.607 | 1 | 11.1 (0-29.6) | 11.5 (0-30) | 0.483 | 1 |  |
| Dobutamine | Increase | 50 (26.9-70.4) | 48.8 (25.9-71.4) | 0.879 | 1 | 19.2 (3.7-38.5) | 21.4 (3.74-40.7) | 0.289 | 1 | 9.34 (0-28) | 11.5 (0-27.6) | 0.233 | 1 |  |
| Midazolam | Decrease | 50 (29.6-69.2) | 50 (28-69) | 0.508 | 1 | 19.2 (3.45-38.5) | 18.4 (3.23-37.9) | 0.147 | 1 | 9.09 (0-27.3) | 8.7 (0-26.9) | 0.416 | 1 |  |
| Midazolam | Increase | 50 (28.6-70.4) | 50 (28.1-68) | 0.187 | 1 | 19.2 (3.23-39.1) | 18.5 (3.23-37.9) | 0.211 | 1 | 9.09 (0-28) | 8.33 (0-26.9) | 0.361 | 1 |  |
| Morphine | Decrease | 50 (30-69.6) | 50.5 (30.8-69.7) | 0.605 | 1 | 20 (3.57-38.5) | 20 (3.57-39.3) | 0.328 | 1 | 10.3 (0-28) | 10.4 (0-28) | 0.35 | 1 |  |
| Morphine | Increase | 50.4 (28.7-72) | 50 (30-69.2) | 0.336 | 1 | 19.2 (2.17-39.3) | 20 (3.57-39.3) | 0.17 | 1 | 9.09 (0-27.8) | 10.4 (0-27.6) | 0.149 | 1 |  |
| Noradrenaline | Decrease | 50 (28.6-69.6) | 50 (28.6-69) | 0.0828 | 1 | 18.5 (3.45-38.6) | 18.5 (3.33-37.9) | 0.042 | 1 | 8.7 (0-27.6) | 8.7 (0-26.9) | 0.0736 | 1 |  |
| Noradrenaline | Increase | 50 (28.6-70.3) | 48.6 (28-68.3) | <0.0001 | <0.0001 | 18.5 (3.33-39.1) | 18.2 (3.33-37.9) | 0.00835 | 0.226 | 8.33 (0-27.6) | 8.33 (0-26.9) | 0.0312 | 0.842 |  |
| Propofol | Decrease | 50 (30-70.5) | 51.7 (30.8-70.8) | 0.0973 | 1 | 20 (3.7-40) | 20.7 (3.7-40.7) | 0.0506 | 1 | 10.3 (0-28.6) | 10.7 (0-29.6) | 0.0776 | 1 |  |
| Propofol | Increase | 50.6 (29.6-70.8) | 50 (30-70) | 0.698 | 1 | 19.2 (3.57-40) | 20 (3.7-40) | 0.153 | 1 | 10 (0-28.6) | 10.3 (0-28.6) | 0.197 | 1 |  |
| Vasopressin | Decrease | 54.8 (51.9-59.2) | 53.4 (52.1-57.3) | 0.266 | 1 | 27.6 (25.1-33.1) | 26.7 (23.5-30.1) | 0.0468 | 1 | 18.9 (16.5-22.3) | 17.8 (15.6-19.7) | 0.0338 | 0.912 |  |
| Vasopressin | Increase | 54.8 (44.9-60) | 52.3 (44.9-54.8) | 0.143 | 1 | 26.7 (5.75-30.3) | 25.1 (9.25-29.5) | 0.594 | 1 | 17.8 (0-22) | 16.5 (4-21.7) | 0.565 | 1 |  |
| **Name** | **Mean Dose Change** | **Mean LPRx_20** | | | | **% time LPRx_20 > 0** | | | | **% time LPRx_20 > 0.25** | | | |  |
|  |  | **Pre Dose** | **Post Dose** | **P value** | **Adj P Value** | **Pre Dose** | **Post Dose** | **P value** | **Adj P Value** | **Pre Dose** | **Post Dose** | **P value** | **Adj P Value** |  |
| Dobutamine | Decrease | -0.00365 (-0.164-0.15) | -0.00115 (-0.169-0.145) | 0.575 | 1 | 50 (23.4-73.1) | 50 (23.9-71.9) | 0.585 | 1 | 16 (0-40.7) | 16.3 (0-39.5) | 0.628 | 1 |  |
| Dobutamine | Increase | 0.000537 (-0.154-0.14) | -0.00412 (-0.174-0.145) | 0.546 | 1 | 50 (23.1-73.1) | 50 (23.4-72) | 0.673 | 1 | 15.1 (0-38.5) | 16.7 (0-38.5) | 0.894 | 1 |  |
| Midazolam | Decrease | 7.38e-05 (-0.135-0.13) | -0.00564 (-0.139-0.127) | 0.401 | 1 | 50 (26.7-71.4) | 48.4 (25.9-71.4) | 0.273 | 1 | 13.8 (0-35.7) | 13.3 (0-35.2) | 0.339 | 1 |  |
| Midazolam | Increase | 0.00773 (-0.135-0.14) | -0.000616 (-0.143-0.129) | 0.0318 | 0.86 | 50 (25.9-73.3) | 50 (26.1-70.8) | 0.0822 | 1 | 14.3 (0-36.4) | 13.8 (0-35.4) | 0.326 | 1 |  |
| Morphine | Decrease | 0.00447 (-0.13-0.139) | 0.00583 (-0.126-0.143) | 0.468 | 1 | 50 (28.3-72.4) | 51.3 (29-72.4) | 0.503 | 1 | 15.4 (0-36.5) | 15.6 (0-37.9) | 0.814 | 1 |  |
| Morphine | Increase | 0.00696 (-0.136-0.148) | 0.00598 (-0.135-0.141) | 0.341 | 1 | 51.7 (26.9-75) | 51.6 (28.6-71.4) | 0.204 | 1 | 14.3 (0-37.5) | 15.4 (0-37.5) | 0.199 | 1 |  |
| Noradrenaline | Decrease | -0.00198 (-0.144-0.135) | -0.00428 (-0.144-0.128) | 0.0127 | 0.343 | 50 (25.8-71.4) | 50 (25.9-70.4) | 0.0353 | 0.954 | 13.8 (0-36) | 13.5 (0-35.7) | 0.000486 | 0.0131 |  |
| Noradrenaline | Increase | 0.000703 (-0.14-0.137) | -0.0102 (-0.151-0.123) | <0.0001 | <0.0001 | 50 (26.1-72) | 48.3 (25-70) | <0.0001 | <0.0001 | 13.8 (0-36.4) | 13.3 (0-34.6) | <0.0001 | <0.0001 |  |
| Propofol | Decrease | 0.00724 (-0.136-0.147) | 0.0112 (-0.127-0.154) | 0.0129 | 0.348 | 50.5 (28-73.9) | 51.7 (29.2-73.9) | 0.0167 | 0.45 | 16.3 (0-38.5) | 16.7 (0-40) | 0.0648 | 1 |  |
| Propofol | Increase | 0.00842 (-0.136-0.15) | 0.00819 (-0.13-0.149) | 0.62 | 1 | 50 (26.9-74.1) | 51.7 (28.7-73.1) | 0.356 | 1 | 14.8 (0-38.5) | 16.1 (0-38.6) | 0.055 | 1 |  |
| Vasopressin | Decrease | 0.0389 (0.021-0.0835) | 0.0278 (-0.0129-0.0517) | 0.235 | 1 | 53.8 (51.6-58.3) | 52.6 (51.6-55.1) | 0.341 | 1 | 25.3 (22.4-30.2) | 24.1 (22.4-26.9) | 0.219 | 1 |  |
| Vasopressin | Increase | 0.0278 (-0.0487-0.0686) | 0.0236 (-0.048-0.0487) | 0.512 | 1 | 53.8 (37.9-58.3) | 52.6 (37.5-57.9) | 0.449 | 1 | 24.1 (1.61-27.7) | 22.4 (4.61-26.9) | 0.471 | 1 |  |
| **Name** | **Mean Dose Change** | **% time LPRx_20 > 0.35** | | | | **Mean LPRx_30** | | | | **% time LPRx_30 > 0** | | | |  |
|  |  | **Pre Dose** | **Post Dose** | **P value** | **Adj P Value** | **Pre Dose** | **Post Dose** | **P value** | **Adj P Value** | **Pre Dose** | **Post Dose** | **P value** | **Adj P Value** |  |
| Dobutamine | Decrease | 4.65 (0-27.3) | 6.67 (0-27.4) | 0.957 | 1 | -0.0159 (-0.167-0.164) | -0.0186 (-0.186-0.143) | 0.0947 | 1 | 48 (17-79.4) | 46.2 (16.7-75.9) | 0.243 | 1 |  |
| Dobutamine | Increase | 3.85 (0-24.9) | 6.67 (0-26.2) | 0.354 | 1 | -0.00383 (-0.155-0.15) | -0.0232 (-0.175-0.135) | 0.0336 | 0.908 | 50 (19.2-79.3) | 44.9 (16.7-76) | 0.0281 | 0.758 |  |
| Midazolam | Decrease | 3.7 (0-22.2) | 3.57 (0-21.7) | 0.483 | 1 | -0.00134 (-0.137-0.13) | -0.00132 (-0.14-0.125) | 0.635 | 1 | 50 (20.7-76) | 48.3 (20.7-75.9) | 0.654 | 1 |  |
| Midazolam | Increase | 3.7 (0-23.1) | 3.57 (0-22.2) | 0.583 | 1 | 0.00368 (-0.132-0.142) | -0.00126 (-0.136-0.129) | 0.15 | 1 | 50 (20-79.1) | 49.9 (20.7-76) | 0.208 | 1 |  |
| Morphine | Decrease | 4 (0-24.1) | 4.55 (0-24.4) | 0.403 | 1 | 0.01 (-0.126-0.148) | 0.00743 (-0.125-0.149) | 0.735 | 1 | 51.9 (24.1-77.8) | 51.6 (23.1-78.7) | 0.855 | 1 |  |
| Morphine | Increase | 3.7 (0-23.3) | 4.17 (0-24.1) | 0.00608 | 0.164 | 0.0109 (-0.134-0.152) | 0.00624 (-0.126-0.149) | 0.745 | 1 | 52 (21.4-80.8) | 50.8 (23.1-77.8) | 0.399 | 1 |  |
| Noradrenaline | Decrease | 3.7 (0-23.3) | 3.57 (0-23.1) | 0.000371 | 0.01 | -0.00256 (-0.141-0.134) | -0.00631 (-0.141-0.126) | 0.00987 | 0.266 | 50 (20.7-76.7) | 48.3 (20-75.8) | 0.000549 | 0.0148 |  |
| Noradrenaline | Increase | 3.7 (0-24) | 3.57 (0-22.2) | <0.0001 | 0.00095 | 0.000215 (-0.135-0.138) | -0.00912 (-0.141-0.12) | <0.0001 | <0.0001 | 50 (20.8-77.8) | 48 (19.5-74.1) | <0.0001 | <0.0001 |  |
| Propofol | Decrease | 4.35 (0-25.9) | 6.67 (0-26.9) | 0.0832 | 1 | 0.00977 (-0.13-0.158) | 0.0177 (-0.117-0.169) | <0.0001 | 0.000407 | 51.9 (23.3-79.8) | 53.6 (25-81.5) | <0.0001 | 0.00259 |  |
| Propofol | Increase | 4 (0-25.9) | 5.81 (0-26.7) | 0.0539 | 1 | 0.00968 (-0.13-0.155) | 0.0168 (-0.116-0.162) | <0.0001 | 0.0015 | 51.7 (23.1-80.4) | 53.6 (25-80.8) | 0.000356 | 0.0096 |  |
| Vasopressin | Decrease | 16.3 (13.2-20.9) | 15.2 (9.76-18) | 0.106 | 1 | 0.0326 (-0.0058-0.0915) | 0.0267 (0.021-0.0722) | 0.928 | 1 | 53.1 (40.6-61.2) | 52.2 (51.5-59.1) | 0.827 | 1 |  |
| Vasopressin | Increase | 15.2 (0-18.8) | 14.1 (0.806-17.6) | 0.308 | 1 | 0.0267 (-0.0777-0.0504) | 0.0267 (-0.076-0.0525) | 0.816 | 1 | 51.6 (31.3-60.1) | 51.6 (28.4-55.5) | 0.583 | 1 |  |
| **Name** | **Mean Dose Change** | **% time LPRx_30 > 0.25** | | | | **% time LPRx_30 > 0.35** | | | | **Mean LPRx_60** | | | |  |
|  |  | **Pre Dose** | **Post Dose** | **P value** | **Adj P Value** | **Pre Dose** | **Post Dose** | **P value** | **Adj P Value** | **Pre Dose** | **Post Dose** | **P value** | **Adj P Value** |  |
| Dobutamine | Decrease | 9.09 (0-38.8) | 8 (0-34.9) | 0.132 | 1 | 0 (0-24) | 0 (0-20) | 0.273 | 1 | 0.00232 (-0.171-0.185) | -0.00491 (-0.2-0.173) | 0.273 | 1 |  |
| Dobutamine | Increase | 10 (0-39) | 7.45 (0-36) | 0.0826 | 1 | 0 (0-21.7) | 0 (0-22.1) | 0.704 | 1 | 0.0148 (-0.155-0.199) | -0.0176 (-0.176-0.156) | 0.0036 | 0.0972 |  |
| Midazolam | Decrease | 3.85 (0-31.7) | 4 (0-31.1) | 0.762 | 1 | 0 (0-16) | 0 (0-16) | 0.848 | 1 | 0.00628 (-0.135-0.16) | 0.0111 (-0.121-0.153) | 0.116 | 1 |  |
| Midazolam | Increase | 4.04 (0-33.3) | 4.35 (0-32) | 0.661 | 1 | 0 (0-17.4) | 0 (0-16.7) | 0.601 | 1 | 0.00623 (-0.133-0.165) | 0.0101 (-0.122-0.158) | 0.311 | 1 |  |
| Morphine | Decrease | 7.69 (0-35.1) | 7.69 (0-35.9) | 0.788 | 1 | 0 (0-20) | 0 (0-20.7) | 0.42 | 1 | 0.0196 (-0.133-0.187) | 0.0214 (-0.118-0.182) | 0.324 | 1 |  |
| Morphine | Increase | 7.14 (0-34.6) | 7.69 (0-37) | 0.173 | 1 | 0 (0-18.6) | 0 (0-21.2) | 0.0137 | 0.369 | 0.0197 (-0.135-0.188) | 0.0203 (-0.12-0.186) | 0.423 | 1 |  |
| Noradrenaline | Decrease | 5.88 (0-32.7) | 3.7 (0-32) | <0.0001 | <0.0001 | 0 (0-17.4) | 0 (0-16.7) | <0.0001 | <0.0001 | 0.0059 (-0.138-0.16) | 0.00253 (-0.136-0.152) | 0.12 | 1 |  |
| Noradrenaline | Increase | 4.76 (0-33.3) | 3.7 (0-31) | <0.0001 | <0.0001 | 0 (0-17.9) | 0 (0-16) | <0.0001 | <0.0001 | 0.0074 (-0.133-0.162) | 0.00477 (-0.13-0.155) | 0.162 | 1 |  |
| Propofol | Decrease | 8.33 (0-37.9) | 8.7 (0-40) | 0.165 | 1 | 0 (0-22.2) | 0 (0-24) | 0.159 | 1 | 0.0227 (-0.124-0.191) | 0.0304 (-0.107-0.205) | <0.0001 | 0.000171 |  |
| Propofol | Increase | 7.41 (0-36.7) | 8.7 (0-40) | 0.00413 | 0.112 | 0 (0-21.1) | 0 (0-24) | 0.00767 | 0.207 | 0.0225 (-0.123-0.191) | 0.0304 (-0.107-0.204) | 0.000153 | 0.00414 |  |
| Vasopressin | Decrease | 20.8 (17.3-27.4) | 20.6 (11.5-24) | 0.586 | 1 | 13.3 (11.6-17.1) | 12.1 (0-14.8) | 0.102 | 1 | 0.0102 (-0.0194-0.0749) | 0.0215 (0.00414-0.0398) | 0.54 | 1 |  |
| Vasopressin | Increase | 20.6 (0-21.9) | 19.7 (0-20.6) | 0.47 | 1 | 12.4 (0-13.9) | 11.9 (0-12.9) | 0.357 | 1 | 0.01 (-0.0814-0.0215) | 0.01 (-0.102-0.038) | 0.722 | 1 |  |
| **Name** | **Mean Dose Change** | **% time LPRx_60 > 0** | | | | **% time LPRx_60 > 0.25** | | | | **% time LPRx_60 > 0.35** | | | |  |
|  |  | **Pre Dose** | **Post Dose** | **P value** | **Adj P Value** | **Pre Dose** | **Post Dose** | **P value** | **Adj P Value** | **Pre Dose** | **Post Dose** | **P value** | **Adj P Value** |  |
| Dobutamine | Decrease | 50 (4.45-96.3) | 47.7 (0-100) | 0.54 | 1 | 0 (0-37.6) | 0 (0-34.3) | 0.0753 | 1 | 0 (0-17.3) | 0 (0-12.2) | 0.24 | 1 |  |
| Dobutamine | Increase | 56 (10.3-100) | 43.5 (0-96.1) | 0.000707 | 0.0191 | 0 (0-42.9) | 0 (0-28.6) | 0.000719 | 0.0194 | 0 (0-16) | 0 (0-7.14) | 0.00784 | 0.212 |  |
| Midazolam | Decrease | 51.3 (7.14-96) | 53.6 (7.69-96.4) | 0.176 | 1 | 0 (0-29.6) | 0 (0-28) | 0.283 | 1 | 0 (0-8) | 0 (0-7.18) | 0.0812 | 1 |  |
| Midazolam | Increase | 51.9 (4.55-96.7) | 52.9 (7.14-96.7) | 0.293 | 1 | 0 (0-31.1) | 0 (0-28.6) | 0.249 | 1 | 0 (0-10.7) | 0 (0-6.88) | 0.0518 | 1 |  |
| Morphine | Decrease | 55.6 (8.7-100) | 56.7 (10.7-100) | 0.196 | 1 | 0 (0-38.7) | 0 (0-36) | 0.174 | 1 | 0 (0-17.9) | 0 (0-14.3) | 0.0288 | 0.779 |  |
| Morphine | Increase | 56 (7.41-100) | 56 (7.41-100) | 0.675 | 1 | 0 (0-37) | 0 (0-36) | 0.37 | 1 | 0 (0-14.8) | 0 (0-13.8) | 0.14 | 1 |  |
| Noradrenaline | Decrease | 51.5 (7.14-95.8) | 50 (3.45-96.6) | 0.00696 | 0.188 | 0 (0-31) | 0 (0-26.9) | <0.0001 | <0.0001 | 0 (0-10.3) | 0 (0-4.35) | <0.0001 | <0.0001 |  |
| Noradrenaline | Increase | 51.9 (7.41-96.3) | 51.7 (3.45-97.8) | 0.00721 | 0.195 | 0 (0-30.8) | 0 (0-26.9) | <0.0001 | <0.0001 | 0 (0-10) | 0 (0-3.85) | <0.0001 | <0.0001 |  |
| Propofol | Decrease | 57.1 (10.7-100) | 60.7 (12-100) | <0.0001 | 0.000543 | 0 (0-39.3) | 0 (0-41.7) | 0.914 | 1 | 0 (0-16.7) | 0 (0-17.2) | 0.815 | 1 |  |
| Propofol | Increase | 57.1 (11.1-100) | 60.7 (11.5-100) | 0.00021 | 0.00566 | 0 (0-38.5) | 0 (0-40.7) | 0.469 | 1 | 0 (0-16.3) | 0 (0-16.1) | 0.362 | 1 |  |
| Vasopressin | Decrease | 50.2 (39.6-61.9) | 52.4 (49.9-69.7) | 0.563 | 1 | 14.8 (0-16.1) | 14.5 (0-17.9) | 0.808 | 1 | 9.67 (0-9.94) | 9.66 (0-10.8) | 0.552 | 1 |  |
| Vasopressin | Increase | 50 (3.36-51.5) | 49.9 (3.25-54) | 0.817 | 1 | 14.5 (0-15.2) | 14.5 (0-16.4) | 0.867 | 1 | 9.66 (0-9.94) | 6.44 (0-9.94) | 0.554 | 1 |  |
| **Bolus** | | | | | | | | | | | | | |  |
| **Name** | **Doses** | **Mean MAP** | | | | **Mean CPP** | | | | **% time CPP>60** | | | |  |
|  |  | **Pre Dose** | **Post Dose** | **P value** | **Adj P Value** | **Pre Dose** | **Post Dose** | **P value** | **Adj P Value** | **Pre Dose** | **Post Dose** | **P value** | **Adj P Value** |  |
| Ephedrine | 43 | 71.4 (63.6-81) | 77.8 (67.3-86.7) | 0.137 | 1 | 57.4 (54.1-72.4) | 62.3 (55.7-74.9) | 0.183 | 1 | 70.4 (6.79-100) | 46.2 (4.26-100) | 0.749 | 1 |  |
| Fentanyl | 214 | 81.2 (75.5-89.3) | 80.2 (72.5-87.9) | 0.125 | 1 | 69.3 (61.6-77.9) | 68 (61.4-76.1) | 0.455 | 1 | 6.9 (0-36.8) | 11.1 (0-37.5) | 0.32 | 1 |  |
| Midazolam | 1097 | 79.4 (75-85.6) | 78.9 (74.2-85.1) | 0.0779 | 1 | 63.7 (58.8-69.7) | 63.6 (58.3-68.9) | 0.36 | 1 | 20 (0-66.7) | 20 (0-72.7) | 0.762 | 1 |  |
| Morphine | 4592 | 81.6 (75.3-90.5) | 80.3 (74-88.9) | <0.0001 | <0.0001 | 68.8 (61.9-78.9) | 67.2 (60.8-76.2) | <0.0001 | <0.0001 | 2.56 (0-34.6) | 4.49 (0-46.4) | <0.0001 | <0.0001 |  |
| Propofol | 4783 | 80.3 (74.8-86.7) | 79.2 (73.7-85.7) | <0.0001 | <0.0001 | 66 (60.3-73.1) | 65 (59.6-71.8) | <0.0001 | 0.000675 | 8 (0-50) | 12 (0-57.4) | 0.000289 | 0.00781 |  |
| Rapifen | 392 | 78.4 (72.6-85.7) | 77.3 (71.7-83.5) | 0.0903 | 1 | 66.6 (60.5-74.1) | 65.5 (60.6-73.5) | 0.417 | 1 | 11.7 (0-46.2) | 14.8 (0-53.4) | 0.761 | 1 |  |
| **Name** | **% time CPP>70** | | | | **Mean ICP** | | | | **% time ICP>20** | | | |  |  |
|  | **Pre Dose** | **Post Dose** | **P value** | **Adj P Value** | **Pre Dose** | **Post Dose** | **P value** | **Adj P Value** | **Pre Dose** | **Post Dose** | **P value** | **Adj P Value** |  |  |
| Ephedrine | 0 (0-46.4) | 7.41 (0-69.9) | 0.223 | 1 | 11.5 (5.64-16) | 10.7 (6.35-16.1) | 0.704 | 1 | 0 (0-3.01) | 0 (0-4.26) | 0.865 | 1 |  |  |
| Fentanyl | 34.6 (8.14-95.5) | 32 (3.64-82.1) | 0.0719 | 1 | 12.3 (7.55-15.7) | 11.1 (7.69-15.1) | 0.147 | 1 | 0 (0-10.4) | 0 (0-6.84) | 0.542 | 1 |  |  |
| Midazolam | 6.12 (0-43.5) | 3.7 (0-37) | 0.0676 | 1 | 16.5 (12.7-19.3) | 16 (12.4-19.3) | 0.155 | 1 | 4.17 (0-29.2) | 3.7 (0-27) | 0.127 | 1 |  |  |
| Morphine | 32 (2.22-96.2) | 23.3 (0-88.3) | <0.0001 | <0.0001 | 12.5 (8.73-16.5) | 12.7 (8.87-16.7) | 0.14 | 1 | 0 (0-7.14) | 0 (0-7.14) | 0.924 | 1 |  |  |
| Propofol | 13.8 (0-70.4) | 8.33 (0-63.5) | <0.0001 | <0.0001 | 14.2 (10.1-18) | 14 (9.76-17.9) | 0.132 | 1 | 0 (0-14.3) | 0 (0-12.5) | 0.000607 | 0.0164 |  |  |
| Alfentanil | 17.6 (3.06-72) | 13.3 (0-72) | 0.428 | 1 | 11.6 (7.81-15) | 10.9 (7.32-14.1) | 0.154 | 1 | 0 (0-4.21) | 0 (0-3.33) | 0.0112 | 0.303 |  |  |
| **Name** | **% time ICP>22** | | | | **Mean LPRx_10** | | | | **% time LPRx_10 > 0** | | | |  |  |
|  | **Pre Dose** | **Post Dose** | **P value** | **Adj P Value** | **Pre Dose** | **Post Dose** | **P value** | **Adj P Value** | **Pre Dose** | **Post Dose** | **P value** | **Adj P Value** |  |  |
| Ephedrine | 0 (0-0.122) | 0 (0-0.114) | 0.428 | 1 | 0.0132 (-0.0743-0.113) | -0.0197 (-0.239-0.0868) | 0.0432 | 1 | 53.3 (40.3-66) | 42.9 (28.3-55.2) | 0.0445 | 1 |  |  |
| Fentanyl | 0 (0-3.7) | 0 (0-3.57) | 0.496 | 1 | 0.01 (-0.0982-0.136) | -0.0032 (-0.165-0.144) | 0.544 | 1 | 53.6 (36-66.3) | 54.5 (30.8-70.2) | 0.867 | 1 |  |  |
| Midazolam | 0 (0-8.33) | 0 (0-7.69) | 0.259 | 1 | 0.00334 (-0.136-0.14) | 0.0102 (-0.127-0.146) | 0.375 | 1 | 50 (32.1-66.7) | 51.2 (34.6-68) | 0.292 | 1 |  |  |
| Morphine | 0 (0-0.757) | 0 (0-1.23) | 0.454 | 1 | 0.0245 (-0.107-0.176) | 0.0245 (-0.107-0.163) | 0.411 | 1 | 52.5 (35.7-71.1) | 52.3 (36.6-69.6) | 0.651 | 1 |  |  |
| Propofol | 0 (0-4.3) | 0 (0-3.85) | 0.000417 | 0.0113 | 0.0193 (-0.127-0.165) | 0.0175 (-0.133-0.16) | 0.506 | 1 | 52 (33.3-69.6) | 51.9 (33.3-69) | 0.6 | 1 |  |  |
| Alfentanil | 0 (0-2.89) | 0 (0-0) | 0.0224 | 0.605 | 0.0118 (-0.128-0.153) | 0.00404 (-0.141-0.146) | 0.852 | 1 | 50 (32.1-70.4) | 50.2 (34.2-66.7) | 0.914 | 1 |  |  |
| **Name** | **% time LPRx_10 > 0.25** | | | | **% time LPRx_10 > 0.35** | | | | **Mean LPRx_15** | | | |  |  |
|  | **Pre Dose** | **Post Dose** | **P value** | **Adj P Value** | **Pre Dose** | **Post Dose** | **P value** | **Adj P Value** | **Pre Dose** | **Post Dose** | **P value** | **Adj P Value** |  |  |
| Ephedrine | 32.5 (12.6-42.4) | 21.7 (6.17-33.9) | 0.181 | 1 | 26.2 (4.15-32.7) | 15.4 (3.61-25.7) | 0.133 | 1 | 0.0208 (-0.108-0.121) | -0.048 (-0.242-0.0468) | 0.0295 | 0.795 |  |  |
| Fentanyl | 26.7 (11.2-40.7) | 25 (10.2-42.9) | 0.952 | 1 | 18.4 (4.19-29.4) | 15.6 (3.48-30) | 0.689 | 1 | 0.0155 (-0.0933-0.134) | -0.0208 (-0.139-0.158) | 0.196 | 1 |  |  |
| Midazolam | 25 (8.33-40.6) | 26.3 (11.1-41.7) | 0.128 | 1 | 15.4 (3.7-30.8) | 17.4 (4-32.1) | 0.0375 | 1 | 0.00445 (-0.137-0.135) | 0.00814 (-0.111-0.136) | 0.288 | 1 |  |  |
| Morphine | 27.1 (11.5-44) | 27.6 (12.5-42.9) | 0.389 | 1 | 18.2 (4.17-33.3) | 19 (6.25-33.3) | 0.311 | 1 | 0.0252 (-0.101-0.169) | 0.0261 (-0.107-0.16) | 0.3 | 1 |  |  |
| Propofol | 25 (10.3-42.9) | 26.1 (11.1-42.3) | 0.206 | 1 | 16 (3.7-32.1) | 17.4 (4.12-33.3) | 0.0506 | 1 | 0.0155 (-0.115-0.162) | 0.0202 (-0.122-0.16) | 0.842 | 1 |  |  |
| Alfentanil | 25.5 (10.6-41.8) | 27.1 (11.4-41.5) | 0.733 | 1 | 15.1 (3.7-31.4) | 19.2 (5.94-31.1) | 0.139 | 1 | 0.00912 (-0.122-0.149) | 0.0128 (-0.115-0.139) | 0.92 | 1 |  |  |
| **Name** | **% time LPRx_15 > 0** | | | | **% time LPRx_15 > 0.25** | | | | **% time LPRx_15 > 0.35** | | | |  |  |
|  | **Pre Dose** | **Post Dose** | **P value** | **Adj P Value** | **Pre Dose** | **Post Dose** | **P value** | **Adj P Value** | **Pre Dose** | **Post Dose** | **P value** | **Adj P Value** |  |  |
| Ephedrine | 53.3 (32.9-64.4) | 45.7 (25-52.1) | 0.0644 | 1 | 30.5 (4.3-38) | 20.8 (5.79-35.8) | 0.533 | 1 | 17.9 (1.79-27.1) | 17.4 (0-23.1) | 0.424 | 1 |  |  |
| Fentanyl | 53.8 (33.3-70.4) | 50 (29.3-70) | 0.482 | 1 | 20.8 (4-34.6) | 18.5 (3.6-40) | 0.998 | 1 | 11.1 (0-23.3) | 10 (0-26.2) | 0.595 | 1 |  |  |
| Midazolam | 50 (29.6-69.2) | 51.7 (33.3-69.2) | 0.207 | 1 | 19 (3.57-36.4) | 22.2 (6.9-39.3) | 0.0114 | 0.309 | 8.33 (0-25.9) | 12 (0-26.9) | 0.025 | 0.675 |  |  |
| Morphine | 53.6 (33.3-73.1) | 53.6 (34.3-72.4) | 0.68 | 1 | 23.1 (4.76-41.9) | 23.1 (6.67-41.7) | 0.68 | 1 | 12.5 (0-29.7) | 13.6 (0-30) | 0.443 | 1 |  |  |
| Propofol | 52 (32-72.4) | 52.5 (33.3-71.9) | 0.917 | 1 | 20.7 (3.7-40.7) | 22.2 (4.21-41.7) | 0.0232 | 0.627 | 10.7 (0-28.6) | 11.5 (0-29.5) | 0.0337 | 0.909 |  |  |
| Alfentanil | 50.8 (32-72.1) | 51.9 (32.1-71) | 0.809 | 1 | 18.3 (3.57-37) | 22.8 (3.67-39.3) | 0.357 | 1 | 8.33 (0-26.9) | 13.2 (0-26.9) | 0.118 | 1 |  |  |
| **Name** | **Mean LPRx_20** | | | | **% time LPRx_20 > 0** | | | | **% time LPRx_20 > 0.25** | | | |  |  |
|  | **Pre Dose** | **Post Dose** | **P value** | **Adj P Value** | **Pre Dose** | **Post Dose** | **P value** | **Adj P Value** | **Pre Dose** | **Post Dose** | **P value** | **Adj P Value** |  |  |
| Ephedrine | 0.0288 (-0.0823-0.145) | -0.0181 (-0.217-0.0419) | 0.0288 | 0.778 | 51.7 (35.6-67.3) | 44.4 (23.6-56.3) | 0.0824 | 1 | 29.8 (7.06-35.7) | 22.2 (1.14-29.6) | 0.207 | 1 |  |  |
| Fentanyl | 0.0257 (-0.103-0.121) | -0.0114 (-0.139-0.15) | 0.226 | 1 | 53.6 (30-70.4) | 49.9 (30.8-67.9) | 0.64 | 1 | 19.5 (0-33.1) | 13.3 (0-33.3) | 0.513 | 1 |  |  |
| Midazolam | 0.00979 (-0.131-0.147) | 0.0166 (-0.105-0.134) | 0.298 | 1 | 50 (28-72.4) | 51.9 (32-70.8) | 0.377 | 1 | 15.4 (0-36.4) | 18.5 (0-38.3) | 0.119 | 1 |  |  |
| Morphine | 0.0291 (-0.105-0.17) | 0.0235 (-0.11-0.166) | 0.325 | 1 | 53.8 (32-76.9) | 53.6 (31-76) | 0.291 | 1 | 20 (0-41.3) | 20.4 (0-41.4) | 0.697 | 1 |  |  |
| Propofol | 0.0219 (-0.114-0.162) | 0.0248 (-0.107-0.163) | 0.246 | 1 | 53.3 (29.6-76) | 53.8 (31.8-75.9) | 0.297 | 1 | 17.2 (0-40) | 18.5 (0-40.7) | 0.038 | 1 |  |  |
| Alfentanil | 0.00764 (-0.113-0.151) | 0.019 (-0.11-0.148) | 0.854 | 1 | 51.9 (29.6-76) | 53.2 (31.9-75) | 0.658 | 1 | 13.8 (0-38.5) | 20 (0-37.9) | 0.179 | 1 |  |  |
| **Name** | **% time LPRx_20 > 0.35** | | | | **Mean LPRx_30** | | | | **% time LPRx_30 > 0** | | | |  |  |
|  | **Pre Dose** | **Post Dose** | **P value** | **Adj P Value** | **Pre Dose** | **Post Dose** | **P value** | **Adj P Value** | **Pre Dose** | **Post Dose** | **P value** | **Adj P Value** |  |  |
| Ephedrine | 12.2 (0-26.7) | 13 (0-21.6) | 0.49 | 1 | 0.0452 (-0.0873-0.136) | 0.00398 (-0.139-0.067) | 0.355 | 1 | 46.7 (23.8-70) | 52.5 (30.2-63.7) | 0.73 | 1 |  |  |
| Fentanyl | 7.41 (0-22.1) | 6.9 (0-25) | 0.938 | 1 | 0.00362 (-0.0919-0.139) | 0.00297 (-0.119-0.15) | 0.779 | 1 | 50 (26.2-75) | 50 (27.1-73.2) | 0.467 | 1 |  |  |
| Midazolam | 4 (0-24.1) | 7.41 (0-25) | 0.13 | 1 | 0.0103 (-0.134-0.152) | 0.0156 (-0.102-0.15) | 0.296 | 1 | 53.8 (23.3-80) | 52.2 (25.8-78.6) | 0.964 | 1 |  |  |
| Morphine | 7.69 (0-28) | 8.33 (0-27.9) | 0.59 | 1 | 0.0289 (-0.113-0.182) | 0.0314 (-0.11-0.173) | 0.943 | 1 | 55.6 (26.9-84) | 55.9 (26.9-83.3) | 0.95 | 1 |  |  |
| Propofol | 6.52 (0-25.9) | 7.41 (0-27.6) | 0.0549 | 1 | 0.0243 (-0.113-0.168) | 0.035 (-0.0953-0.179) | 0.00358 | 0.0967 | 54.5 (26.3-82.6) | 56.7 (28.6-84.6) | 0.00648 | 0.175 |  |  |
| Alfentanil | 4 (0-25.9) | 7.69 (0-24.1) | 0.211 | 1 | 0.025 (-0.103-0.159) | 0.0256 (-0.0976-0.168) | 0.949 | 1 | 51.9 (27.6-80) | 55.6 (27.7-82.6) | 0.508 | 1 |  |  |
| **Name** | **% time LPRx_30 > 0.25** | | | | **% time LPRx_30 > 0.35** | | | | **Mean LPRx_60** | | | |  |  |
|  | **Pre Dose** | **Post Dose** | **P value** | **Adj P Value** | **Pre Dose** | **Post Dose** | **P value** | **Adj P Value** | **Pre Dose** | **Post Dose** | **P value** | **Adj P Value** |  |  |
| Ephedrine | 20 (0-32.7) | 15.4 (0-29.6) | 0.763 | 1 | 4.17 (0-22.9) | 3.57 (0-22.7) | 0.787 | 1 | 0.0684 (-0.141-0.24) | 0.0412 (-0.0608-0.199) | 0.863 | 1 |  |  |
| Fentanyl | 10 (0-37.8) | 14.6 (0-39.3) | 0.245 | 1 | 0 (0-21.7) | 0 (0-24.9) | 0.627 | 1 | 0.0455 (-0.124-0.248) | 0.018 (-0.135-0.229) | 0.542 | 1 |  |  |
| Midazolam | 10 (0-37) | 11.9 (0-38.5) | 0.624 | 1 | 0 (0-20.2) | 0 (0-21.7) | 0.171 | 1 | 0.0372 (-0.147-0.211) | 0.0319 (-0.107-0.205) | 0.611 | 1 |  |  |
| Morphine | 13.8 (0-41.7) | 13.8 (0-40.7) | 0.541 | 1 | 2.22 (0-25) | 0 (0-25) | 0.887 | 1 | 0.0329 (-0.127-0.215) | 0.0378 (-0.129-0.213) | 0.883 | 1 |  |  |
| Propofol | 10.7 (0-39.3) | 11.5 (0-41.4) | 0.403 | 1 | 0 (0-22.2) | 0 (0-24.1) | 0.48 | 1 | 0.0381 (-0.118-0.211) | 0.0464 (-0.0952-0.221) | 0.00855 | 0.231 |  |  |
| Alfentanil | 12.5 (0-39.3) | 12.7 (0-34.6) | 0.53 | 1 | 0 (0-21.4) | 0 (0-18.5) | 0.455 | 1 | 0.0396 (-0.102-0.176) | 0.0452 (-0.0864-0.2) | 0.691 | 1 |  |  |
| **Name** | **% time LPRx_60 > 0** | | | | **% time LPRx_60 > 0.25** | | | | **% time LPRx_60 > 0.35** | | | |  |  |
|  | **Pre Dose** | **Post Dose** | **P value** | **Adj P Value** | **Pre Dose** | **Post Dose** | **P value** | **Adj P Value** | **Pre Dose** | **Post Dose** | **P value** | **Adj P Value** |  |  |
| Ephedrine | 55 (21-88.8) | 52.6 (24-95) | 0.886 | 1 | 22.7 (0-50) | 3.33 (0-49) | 0.537 | 1 | 11.5 (0-31.6) | 0 (0-34.8) | 0.554 | 1 |  |  |
| Fentanyl | 63.6 (7.41-100) | 59.3 (0-100) | 0.541 | 1 | 3.57 (0-51.9) | 0 (0-50) | 0.157 | 1 | 0 (0-21.7) | 0 (0-29.1) | 0.419 | 1 |  |  |
| Midazolam | 62.5 (8-100) | 57.1 (10.7-100) | 0.621 | 1 | 0 (0-44) | 0 (0-42.2) | 0.569 | 1 | 0 (0-23.7) | 0 (0-17.2) | 0.15 | 1 |  |  |
| Morphine | 58.6 (12.8-100) | 59.3 (10.8-100) | 0.276 | 1 | 0 (0-44.8) | 0 (0-43.3) | 0.0775 | 1 | 0 (0-23.8) | 0 (0-19.8) | 0.00909 | 0.245 |  |  |
| Propofol | 60.7 (14.3-100) | 66.7 (16.7-100) | 0.000652 | 0.0176 | 0 (0-43.5) | 0 (0-45) | 0.512 | 1 | 0 (0-20) | 0 (0-17.3) | 0.0435 | 1 |  |  |
| Alfentanil | 60 (18.1-97.5) | 63.8 (17.4-100) | 0.412 | 1 | 0 (0-38.5) | 0 (0-36) | 0.312 | 1 | 0 (0-23.2) | 0 (0-11.7) | 0.0574 | 1 |  |  |

*The table demonstrates the median and interquartile range of the pre/pose dose windows as well as the Wilcox signed ranked test between these windows with p values adjusted using the Bonferroni analysis. The “change” indicates if the continuous infusion was increase/decrease or was a bolus dose. CPP, cerebral prefusion pressure; ICP, intracranial pressure; LPRx_10, pressure reactivity over 10 minutes; LPRx_15, pressure reactivity over 15 minutes; LPRx_20, pressure reactivity over 20 minutes; LPRx_30, pressure reactivity over 30 minutes; LPRx_60, pressure reactivity over 60 minutes;*

# Appendix G. Pre-time window over 50% time ICP > 20mmHg

The table contains all infusions given with the ICP pre-time window 50% > 20 mmHg, separated into the continuous infusion doses then the bolus doses.

| **Continuous Intravenous** | | | | | | | | | | | | | | |
| --- | --- | --- | --- | --- | --- | --- | --- | --- | --- | --- | --- | --- | --- | --- |
| **Name** | **Doses** | **Mean Dose Change** | **Mean MAP** | | | | **Mean CPP** | | | | **% time CPP>60** | | | |
|  |  |  | **Pre Dose** | **Post Dose** | **P value** | **Adj P Value** | **Pre Dose** | **Post Dose** | **P value** | **Adj P Value** | **Pre Dose** | **Post Dose** | **P value** | **Adj P Value** |
| Dobutamine | 108 | Decrease | 83.5 (75.6-88.9) | 82 (77.4-87.1) | 0.252 | 1 | 60.7 (53.7-65.9) | 61.3 (58-65.8) | 0.394 | 1 | 54.3 (16.6-95.5) | 31.1 (8.31-80.2) | 0.0884 | 1 |
| Dobutamine | 99 | Increase | 78.6 (72.8-84.7) | 78.8 (72.1-83.7) | 0.637 | 1 | 57 (52.2-62.9) | 57.4 (52.1-62.3) | 0.949 | 1 | 86 (53.8-100) | 82.2 (39.9-100) | 0.898 | 1 |
| Midazolam | 531 | Increase | 83 (77.8-89.5) | 83.1 (78.1-89.4) | 0.67 | 1 | 60.7 (55.4-67.2) | 62.5 (57.2-68.5) | 0.00719 | 0.194 | 48.1 (3.7-92.6) | 31 (0-84) | 0.0129 | 0.348 |
| Midazolam | 397 | Decrease | 83.9 (77.6-90.7) | 84 (77.8-89.6) | 0.423 | 1 | 61 (55.5-68.2) | 62.9 (57-68.7) | 0.108 | 1 | 40 (3.7-95.7) | 30.8 (3.33-90.9) | 0.0729 | 1 |
| Morphine | 584 | Increase | 83 (77.6-89.2) | 81.7 (76-87.8) | 0.00409 | 0.11 | 60.6 (55-66.6) | 61.6 (56.1-67) | 0.272 | 1 | 50 (3.85-92.4) | 40.4 (3.51-93.2) | 0.483 | 1 |
| Morphine | 483 | Decrease | 83 (77.5-90.4) | 81.5 (76.1-88.6) | 0.0174 | 0.469 | 60.4 (54.4-67.2) | 60.9 (55.2-67.4) | 0.398 | 1 | 55.2 (5.83-94.3) | 48.1 (3.57-96.2) | 0.559 | 1 |
| Noradrenaline | 3785 | Increase | 81.6 (76-88.6) | 81.4 (75.9-88.4) | 0.694 | 1 | 59.4 (54-65.7) | 60.8 (55.5-67.6) | <0.0001 | <0.0001 | 60.7 (12-96.2) | 46.2 (3.85-96.2) | <0.0001 | <0.0001 |
| Noradrenaline | 3416 | Decrease | 83.6 (78-90.7) | 82.6 (77.2-89.6) | <0.0001 | 0.000108 | 61.1 (55.4-67.8) | 62.1 (56.8-68.9) | <0.0001 | 0.000194 | 42.9 (3.85-92.3) | 32.5 (0-88.9) | <0.0001 | 0.00115 |
| Propofol | 934 | Increase | 83.4 (78.3-90.1) | 82.1 (77.1-88.4) | 0.00047 | 0.0127 | 60.7 (55.9-67.4) | 61.9 (56.6-67.7) | 0.0685 | 1 | 45.1 (4-87.8) | 34.5 (3.33-88) | 0.122 | 1 |
| Propofol | 907 | Decrease | 83.2 (78.5-90.7) | 83.1 (77.7-89.1) | 0.0798 | 1 | 60.4 (56-67.4) | 62.2 (56.7-68.3) | 0.00518 | 0.14 | 48.1 (6.78-90.9) | 33.3 (3.23-89.3) | 0.018 | 0.487 |
| Vasopressin | 8 | Increase | 71.1 (70.7-72.1) | 72.2 (71.5-72.9) | 0.195 | 1 | 51 (50.2-52.3) | 53 (51.6-55.6) | 0.083 | 1 | 100 (100-100) | 100 (88.9-100) | 0.24 | 1 |
| **Name** | **Mean Dose Change** | **% time CPP>70** | | | | **Mean ICP** | | | | **% time ICP>20** | | | |  |
|  |  | **Pre Dose** | **Post Dose** | **P value** | **Adj P Value** | **Pre Dose** | **Post Dose** | **P value** | **Adj P Value** | **Pre Dose** | **Post Dose** | **P value** | **Adj P Value** |  |
| Dobutamine | Decrease | 2.44 (0-11.6) | 0 (0-12.4) | 0.162 | 1 | 21.7 (20.8-24.4) | 20.1 (18.3-23.5) | <0.0001 | 0.000423 | 98.9 (82.3-100) | 70.7 (3.7-100) | <0.0001 | <0.0001 |  |
| Dobutamine | Increase | 0 (0-7.75) | 0 (0-8.05) | 0.975 | 1 | 21.6 (20.7-22.9) | 21.5 (19-23.9) | 0.0907 | 1 | 92.6 (72.9-100) | 92.9 (24.6-100) | 0.0505 | 1 |  |
| Midazolam | Increase | 3.45 (0-24.6) | 3.45 (0-29.6) | 0.598 | 1 | 21.8 (20.7-23.6) | 20.6 (18.6-22.6) | <0.0001 | <0.0001 | 90.9 (74.7-100) | 76 (18.3-100) | <0.0001 | <0.0001 |  |
| Midazolam | Decrease | 3.23 (0-27.6) | 3.57 (0-30.4) | 0.52 | 1 | 21.6 (20.5-23.8) | 20.8 (18.9-22.8) | <0.0001 | <0.0001 | 91.7 (72.7-100) | 82.4 (21.4-100) | <0.0001 | <0.0001 |  |
| Morphine | Increase | 2.57 (0-20) | 0 (0-20) | 0.929 | 1 | 21.9 (20.8-23.6) | 20.5 (18.4-22.6) | <0.0001 | <0.0001 | 90 (73.1-100) | 70.5 (11.7-100) | <0.0001 | <0.0001 |  |
| Morphine | Decrease | 1.98 (0-19.2) | 0 (0-20.2) | 0.997 | 1 | 22 (20.8-24.1) | 20.7 (18.7-23.4) | <0.0001 | <0.0001 | 92.3 (75.9-100) | 76 (15.6-100) | <0.0001 | <0.0001 |  |
| Noradrenaline | Increase | 0 (0-15.4) | 0 (0-25) | 0.0721 | 1 | 21.4 (20.6-23.2) | 20.6 (18.4-22.4) | <0.0001 | <0.0001 | 88.9 (71.4-100) | 74.1 (14.8-100) | <0.0001 | <0.0001 |  |
| Noradrenaline | Decrease | 3.57 (0-27.6) | 3.33 (0-32.1) | 0.964 | 1 | 21.6 (20.7-23.5) | 20.6 (18.4-22.5) | <0.0001 | <0.0001 | 92 (73.7-100) | 77.8 (16.1-100) | <0.0001 | <0.0001 |  |
| Propofol | Increase | 3.45 (0-23.1) | 2.27 (0-27.6) | 0.862 | 1 | 21.7 (20.7-23.3) | 20.1 (17.8-22.4) | <0.0001 | <0.0001 | 88 (71.4-100) | 56.1 (9.09-96.4) | <0.0001 | <0.0001 |  |
| Propofol | Decrease | 0 (0-24) | 3.33 (0-30.2) | 0.14 | 1 | 21.6 (20.6-23.6) | 20.3 (17.9-22.8) | <0.0001 | <0.0001 | 88.9 (70.6-100) | 65.5 (10.7-100) | <0.0001 | <0.0001 |  |
| Vasopressin | Increase | 0 (0-0) | 0 (0-0) | 1 | NaN | 20.4 (20.1-20.7) | 19.7 (18.6-20.3) | 0.161 | 1 | 60.6 (58.6-69.2) | 39.8 (25.8-60.5) | 0.0401 | 0.923 |  |
| **Name** | **Mean Dose Change** | **% time ICP>22** | | | | **Mean LPRx_10** | | | | **% time LPRx_10 > 0** | | | |  |
|  |  | **Pre Dose** | **Post Dose** | **P value** | **Adj P Value** | **Pre Dose** | **Post Dose** | **P value** | **Adj P Value** | **Pre Dose** | **Post Dose** | **P value** | **Adj P Value** |  |
| Dobutamine | Decrease | 44.2 (22.2-100) | 14.3 (0-93.3) | <0.0001 | 0.00178 | -0.0598 (-0.193-0.133) | 0.0254 (-0.115-0.12) | 0.177 | 1 | 40.7 (26.5-65.4) | 52 (37-66.8) | 0.0294 | 0.793 |  |
| Dobutamine | Increase | 39.3 (17.4-92.2) | 29.2 (0-96.1) | 0.0857 | 1 | -0.038 (-0.164-0.126) | -0.00948 (-0.114-0.122) | 0.671 | 1 | 43.2 (23.1-65.5) | 46.4 (33.5-63) | 0.32 | 1 |  |
| Midazolam | Increase | 38.9 (14.6-84.6) | 15.4 (0-70.6) | <0.0001 | <0.0001 | 0.0157 (-0.147-0.167) | -0.00259 (-0.159-0.137) | 0.181 | 1 | 50 (32.2-69) | 50 (31.1-66.7) | 0.335 | 1 |  |
| Midazolam | Decrease | 37 (11.5-85.7) | 16.7 (0-80) | <0.0001 | 0.000365 | 0.00666 (-0.166-0.163) | -0.00475 (-0.171-0.139) | 0.46 | 1 | 51.7 (33.3-66.7) | 46.7 (30.4-66.7) | 0.208 | 1 |  |
| Morphine | Increase | 41.5 (15.9-82.9) | 14.8 (0-69) | <0.0001 | <0.0001 | 0.0264 (-0.125-0.181) | 0.0112 (-0.16-0.162) | 0.112 | 1 | 52.2 (35.7-70.1) | 51.4 (32.5-69) | 0.271 | 1 |  |
| Morphine | Decrease | 43.5 (14.8-89) | 17.2 (0-83.3) | <0.0001 | <0.0001 | 0.035 (-0.112-0.206) | 0.0195 (-0.152-0.178) | 0.0783 | 1 | 52.4 (36.4-73.1) | 50 (32.1-70.2) | 0.106 | 1 |  |
| Noradrenaline | Increase | 32.1 (11.5-73.3) | 11.1 (0-64) | <0.0001 | <0.0001 | 0.019 (-0.132-0.172) | 0.00939 (-0.151-0.169) | 0.0133 | 0.36 | 50 (33.3-69.2) | 50 (32-68.2) | 0.0631 | 1 |  |
| Noradrenaline | Decrease | 37 (12-81.3) | 11.5 (0-65.5) | <0.0001 | <0.0001 | 0.0141 (-0.139-0.171) | -0.00569 (-0.164-0.167) | 0.00594 | 0.16 | 51.7 (33.3-69.2) | 50 (30.4-68.2) | 0.0259 | 0.7 |  |
| Propofol | Increase | 38.9 (15.5-75) | 10.7 (0-62.4) | <0.0001 | <0.0001 | 0.0377 (-0.129-0.18) | 0.0105 (-0.152-0.177) | 0.0624 | 1 | 53.6 (33.5-71.4) | 50 (32-70.4) | 0.117 | 1 |  |
| Propofol | Decrease | 37.5 (14.3-80) | 14.3 (0-72.7) | <0.0001 | <0.0001 | 0.018 (-0.147-0.18) | -0.00238 (-0.161-0.158) | 0.072 | 1 | 51.9 (32.1-70.4) | 50 (31.8-69.6) | 0.126 | 1 |  |
| Vasopressin | Increase | 15 (12.3-16.3) | 8.06 (5.7-12.1) | 0.155 | 1 | -0.275 (-0.398--0.144) | 0.0451 (-0.189-0.0635) | 0.195 | 1 | 27.4 (13.7-44.7) | 48.2 (21.8-62.1) | 0.462 | 1 |  |
| **Name** | **Mean Dose Change** | **% time LPRx_10 > 0.25** | | | | **% time LPRx_10 > 0.35** | | | | **Mean LPRx_15** | | | |  |
|  |  | **Pre Dose** | **Post Dose** | **P value** | **Adj P Value** | **Pre Dose** | **Post Dose** | **P value** | **Adj P Value** | **Pre Dose** | **Post Dose** | **P value** | **Adj P Value** |  |
| Dobutamine | Decrease | 16.1 (4.23-41.9) | 28.1 (15.1-36.2) | 0.103 | 1 | 8.01 (0-28.5) | 16.7 (6.95-28.7) | 0.0661 | 1 | -0.0768 (-0.206-0.0896) | -0.00363 (-0.138-0.103) | 0.138 | 1 |  |
| Dobutamine | Increase | 23.3 (8.12-37.5) | 23.8 (11.8-35.6) | 0.645 | 1 | 15 (1.16-30.1) | 15.6 (4.4-27.6) | 0.669 | 1 | -0.042 (-0.176-0.111) | -0.0449 (-0.146-0.0657) | 0.679 | 1 |  |
| Midazolam | Increase | 26.9 (11.1-45) | 25 (7.69-41.4) | 0.112 | 1 | 18.5 (4-35.7) | 16 (3.7-33.3) | 0.182 | 1 | 0.00248 (-0.15-0.156) | -0.00281 (-0.155-0.121) | 0.499 | 1 |  |
| Midazolam | Decrease | 25 (10.7-43.5) | 25 (7.69-42.5) | 0.326 | 1 | 16.1 (3.7-34.5) | 15.4 (3.45-33.3) | 0.454 | 1 | -0.0147 (-0.158-0.162) | 0.000882 (-0.141-0.119) | 0.81 | 1 |  |
| Morphine | Increase | 28 (13-46.3) | 26.4 (9.15-44.8) | 0.117 | 1 | 18.5 (4.17-36.4) | 17.9 (3.57-34.6) | 0.236 | 1 | 0.0235 (-0.11-0.164) | 0.0109 (-0.152-0.163) | 0.204 | 1 |  |
| Morphine | Decrease | 28.6 (13.3-48.1) | 27.5 (11.1-46.2) | 0.149 | 1 | 20.7 (6.59-37) | 17.9 (3.85-36.3) | 0.2 | 1 | 0.0198 (-0.117-0.179) | 0.0164 (-0.141-0.171) | 0.393 | 1 |  |
| Noradrenaline | Increase | 25 (10.3-44.4) | 25 (9.68-44.4) | 0.511 | 1 | 16.7 (3.7-34.6) | 17.2 (3.57-34.8) | 0.796 | 1 | 0.0081 (-0.132-0.161) | -0.00251 (-0.147-0.15) | 0.00337 | 0.0909 |  |
| Noradrenaline | Decrease | 25 (8.33-44.4) | 24.1 (8.7-44.4) | 0.404 | 1 | 17.1 (3.57-34.6) | 15.4 (3.45-34.5) | 0.368 | 1 | 0.00651 (-0.135-0.166) | -0.000602 (-0.154-0.159) | 0.0593 | 1 |  |
| Propofol | Increase | 26.9 (11.4-46.2) | 26.1 (10.1-44.8) | 0.48 | 1 | 17.6 (3.85-35.4) | 17.4 (4-35.7) | 0.817 | 1 | 0.0291 (-0.118-0.18) | 0.00933 (-0.143-0.17) | 0.136 | 1 |  |
| Propofol | Decrease | 26.9 (10.7-46.4) | 25 (9.68-43.3) | 0.153 | 1 | 18.5 (5.26-36) | 17.2 (3.85-33.3) | 0.0834 | 1 | 0.0111 (-0.135-0.177) | 0.0054 (-0.147-0.156) | 0.165 | 1 |  |
| Vasopressin | Increase | 0 (0-0) | 11.3 (4.84-13.1) | 0.0369 | 0.849 | 0 (0-0) | 8.06 (4.84-9.84) | 0.0367 | 0.845 | -0.196 (-0.296--0.0472) | 0.00629 (-0.124-0.031) | 0.195 | 1 |  |
| **Name** | **Mean Dose Change** | **% time LPRx_15 > 0** | | | | **% time LPRx_15 > 0.25** | | | | **% time LPRx_15 > 0.35** | | | |  |
|  |  | **Pre Dose** | **Post Dose** | **P value** | **Adj P Value** | **Pre Dose** | **Post Dose** | **P value** | **Adj P Value** | **Pre Dose** | **Post Dose** | **P value** | **Adj P Value** |  |
| Dobutamine | Decrease | 42.1 (12-65.6) | 48.3 (30.2-69.8) | 0.105 | 1 | 5.42 (0-24.1) | 19.3 (6.84-35.6) | 0.000604 | 0.0163 | 1.11 (0-20) | 9.09 (0-25.6) | 0.015 | 0.405 |  |
| Dobutamine | Increase | 40 (20.1-68.5) | 44.4 (26.6-65) | 0.767 | 1 | 11.5 (0-37) | 13.3 (2.3-30.2) | 0.958 | 1 | 4.55 (0-26.9) | 8.89 (0-21.4) | 0.965 | 1 |  |
| Midazolam | Increase | 50 (29.2-69.2) | 51.7 (29.2-68) | 0.989 | 1 | 20.7 (3.77-41.5) | 19.2 (3.33-38) | 0.51 | 1 | 11.1 (0-30) | 8 (0-25.9) | 0.341 | 1 |  |
| Midazolam | Decrease | 51.6 (28.9-69.2) | 50 (29.6-67.9) | 0.865 | 1 | 19.2 (3.33-39.1) | 18.5 (0-37) | 0.575 | 1 | 8 (0-28.6) | 7.69 (0-25.9) | 0.79 | 1 |  |
| Morphine | Increase | 53.5 (33.1-70.6) | 52 (29.2-71.4) | 0.594 | 1 | 22.2 (4-43.3) | 21 (3.45-41.7) | 0.414 | 1 | 11.3 (0-30.8) | 10.7 (0-29.6) | 0.454 | 1 |  |
| Morphine | Decrease | 51.4 (33.3-71.4) | 53.6 (30-74.1) | 0.909 | 1 | 23.1 (6.9-44.2) | 22.2 (3.85-44) | 0.654 | 1 | 12.8 (0-33.3) | 11.1 (0-32.2) | 0.671 | 1 |  |
| Noradrenaline | Increase | 50 (29.6-71.9) | 50 (28.6-70) | 0.0701 | 1 | 20 (3.57-41.7) | 18.5 (3.33-39.3) | 0.1 | 1 | 10 (0-30) | 9.38 (0-29.2) | 0.334 | 1 |  |
| Noradrenaline | Decrease | 50 (29.2-72.7) | 50 (26.9-71.4) | 0.245 | 1 | 19.2 (3.45-42.3) | 19.3 (2.86-40.7) | 0.495 | 1 | 10 (0-29.7) | 8 (0-30.8) | 0.269 | 1 |  |
| Propofol | Increase | 53.6 (32.1-74.1) | 51.9 (30.1-72.4) | 0.318 | 1 | 22.2 (3.7-44.4) | 21.4 (4-42.9) | 0.862 | 1 | 11.1 (0-32) | 11.1 (0-31) | 0.72 | 1 |  |
| Propofol | Decrease | 51.7 (31.5-74.1) | 50 (30.8-70.9) | 0.387 | 1 | 21.7 (3.85-42.9) | 20 (3.7-40.7) | 0.301 | 1 | 11.1 (0-33.3) | 10.7 (0-29.7) | 0.263 | 1 |  |
| Vasopressin | Increase | 32.3 (27.8-42.9) | 45 (21.8-57.3) | 0.562 | 1 | 0 (0-0) | 3.23 (0-3.28) | 0.0124 | 0.285 | 0 (0-0) | 0 (0-0) | 0.382 | 1 |  |
| **Name** | **Mean Dose Change** | **Mean LPRx_20** | | | | **% time LPRx_20 > 0** | | | | **% time LPRx_20 > 0.25** | | | |  |
|  |  | **Pre Dose** | **Post Dose** | **P value** | **Adj P Value** | **Pre Dose** | **Post Dose** | **P value** | **Adj P Value** | **Pre Dose** | **Post Dose** | **P value** | **Adj P Value** |  |
| Dobutamine | Decrease | -0.075 (-0.207-0.112) | -0.00881 (-0.167-0.099) | 0.462 | 1 | 41.8 (13.6-73.3) | 43.4 (22.5-65.7) | 0.461 | 1 | 1.85 (0-21) | 13.6 (0-32.8) | 0.0114 | 0.307 |  |
| Dobutamine | Increase | -0.0325 (-0.174-0.132) | -0.0741 (-0.139-0.0387) | 0.449 | 1 | 42.2 (20-69.6) | 42.2 (22.2-63.9) | 0.99 | 1 | 7.41 (0-38.5) | 4.08 (0-28.9) | 0.356 | 1 |  |
| Midazolam | Increase | -0.0026 (-0.147-0.141) | -0.00725 (-0.147-0.13) | 0.857 | 1 | 48.3 (25.9-72.6) | 50 (28.6-70) | 0.786 | 1 | 14.3 (0-37) | 13.3 (0-37) | 0.66 | 1 |  |
| Midazolam | Decrease | -0.0112 (-0.145-0.144) | -0.00784 (-0.122-0.129) | 0.708 | 1 | 50 (25.7-71.4) | 50 (28.6-71.7) | 0.882 | 1 | 12.5 (0-34.8) | 13 (0-33.3) | 0.688 | 1 |  |
| Morphine | Increase | 0.00667 (-0.122-0.167) | 0.014 (-0.14-0.153) | 0.347 | 1 | 52 (30.8-74.3) | 53.5 (25.9-75) | 0.725 | 1 | 16 (0-40) | 15.3 (0-41.4) | 0.523 | 1 |  |
| Morphine | Decrease | 0.0146 (-0.129-0.173) | 0.0172 (-0.137-0.177) | 0.769 | 1 | 50 (30.2-75) | 55.6 (29.1-75.5) | 0.546 | 1 | 18.5 (0-41.7) | 18.2 (0-42) | 0.903 | 1 |  |
| Noradrenaline | Increase | 0.00561 (-0.128-0.151) | -0.0071 (-0.149-0.142) | 0.0087 | 0.235 | 50 (25.9-74.1) | 50 (25.9-72.4) | 0.128 | 1 | 13.8 (0-38.5) | 14.3 (0-37) | 0.588 | 1 |  |
| Noradrenaline | Decrease | 0.00458 (-0.135-0.157) | 0.00419 (-0.139-0.159) | 0.397 | 1 | 50 (25.9-74.1) | 50 (26.9-75) | 0.8 | 1 | 14.3 (0-39.3) | 14.3 (0-38.8) | 0.817 | 1 |  |
| Propofol | Increase | 0.0297 (-0.12-0.178) | 0.0205 (-0.131-0.169) | 0.272 | 1 | 55.6 (29.6-79.1) | 52.1 (29.6-74.3) | 0.118 | 1 | 17.2 (0-41.6) | 17.9 (0-44) | 0.509 | 1 |  |
| Propofol | Decrease | 0.0136 (-0.13-0.162) | 0.00698 (-0.134-0.149) | 0.324 | 1 | 52 (28-75.9) | 51.2 (30.6-73.3) | 0.566 | 1 | 17.2 (0-40) | 16.7 (0-39.3) | 0.584 | 1 |  |
| Vasopressin | Increase | -0.0952 (-0.229--0.0322) | -0.0197 (-0.12-0.0152) | 0.279 | 1 | 29.4 (19.3-45.7) | 52.5 (26.6-59.3) | 0.43 | 1 | 0 (0-0) | 0 (0-0) | 0.382 | 1 |  |
| **Name** | **Mean Dose Change** | **% time LPRx_20 > 0.35** | | | | **Mean LPRx_30** | | | | **% time LPRx_30 > 0** | | | |  |
|  |  | **Pre Dose** | **Post Dose** | **P value** | **Adj P Value** | **Pre Dose** | **Post Dose** | **P value** | **Adj P Value** | **Pre Dose** | **Post Dose** | **P value** | **Adj P Value** |  |
| Dobutamine | Decrease | 0 (0-12.8) | 3.58 (0-25) | 0.116 | 1 | -0.0311 (-0.174-0.139) | -0.0459 (-0.175-0.0607) | 0.56 | 1 | 43.4 (9.23-80.9) | 41.1 (22-61) | 0.65 | 1 |  |
| Dobutamine | Increase | 0 (0-20) | 0 (0-14.6) | 0.555 | 1 | -0.0544 (-0.18-0.0894) | -0.101 (-0.205-0.0432) | 0.12 | 1 | 38.1 (15.6-74.1) | 29.2 (0-62.8) | 0.0614 | 1 |  |
| Midazolam | Increase | 4.17 (0-23.7) | 3.57 (0-23.1) | 0.401 | 1 | 0.0055 (-0.137-0.146) | 7.02e-05 (-0.135-0.133) | 0.799 | 1 | 50 (19.6-78.9) | 51.7 (20.7-74.1) | 0.698 | 1 |  |
| Midazolam | Decrease | 3.85 (0-24) | 3.45 (0-21.7) | 0.424 | 1 | 0.0027 (-0.133-0.149) | -0.000516 (-0.137-0.139) | 0.943 | 1 | 51.1 (24.1-82.8) | 50 (21.4-76.9) | 0.77 | 1 |  |
| Morphine | Increase | 5 (0-26.9) | 6.9 (0-26.4) | 0.972 | 1 | 0.00878 (-0.116-0.178) | 0.0312 (-0.117-0.172) | 0.936 | 1 | 53.6 (22.2-82.1) | 53.7 (24-83.9) | 0.913 | 1 |  |
| Morphine | Decrease | 7.41 (0-29.2) | 8 (0-28.6) | 0.825 | 1 | 0.0102 (-0.106-0.159) | 0.0236 (-0.121-0.186) | 0.731 | 1 | 51.7 (27-81.5) | 55.2 (26.4-83.7) | 0.361 | 1 |  |
| Noradrenaline | Increase | 3.85 (0-25.9) | 3.7 (0-25.9) | 0.615 | 1 | -0.00165 (-0.127-0.146) | -0.00157 (-0.135-0.148) | 0.63 | 1 | 50 (20.7-79.3) | 50 (21.4-78.6) | 0.991 | 1 |  |
| Noradrenaline | Decrease | 4 (0-25.9) | 3.7 (0-26.9) | 0.644 | 1 | 0.00205 (-0.125-0.164) | 0.0118 (-0.128-0.159) | 0.591 | 1 | 50 (22.2-78.6) | 53.6 (23.3-80) | 0.0581 | 1 |  |
| Propofol | Increase | 6.9 (0-28.6) | 7.14 (0-31) | 0.384 | 1 | 0.0225 (-0.106-0.185) | 0.0219 (-0.107-0.186) | 0.909 | 1 | 56 (26.9-85.7) | 56 (27.6-82.1) | 0.633 | 1 |  |
| Propofol | Decrease | 5 (0-28) | 4.65 (0-26.9) | 0.751 | 1 | 0.0115 (-0.11-0.168) | 0.0108 (-0.126-0.175) | 0.958 | 1 | 51.7 (25-80.2) | 53.3 (25-82.8) | 0.647 | 1 |  |
| Vasopressin | Increase | 0 (0-0) | 0 (0-0) | 0.382 | 1 | -0.0441 (-0.0824--0.0271) | -0.0649 (-0.154--0.021) | 0.721 | 1 | 17 (15.5-25.6) | 25.8 (2.42-26.3) | 0.958 | 1 |  |
| **Name** | **Mean Dose Change** | **% time LPRx_30 > 0.25** | | | | **% time LPRx_30 > 0.35** | | | | **Mean LPRx_60** | | | |  |
|  |  | **Pre Dose** | **Post Dose** | **P value** | **Adj P Value** | **Pre Dose** | **Post Dose** | **P value** | **Adj P Value** | **Pre Dose** | **Post Dose** | **P value** | **Adj P Value** |  |
| Dobutamine | Decrease | 0 (0-22.8) | 4.35 (0-30.1) | 0.066 | 1 | 0 (0-11.9) | 0 (0-16.3) | 0.19 | 1 | -0.018 (-0.1-0.138) | -0.0421 (-0.231-0.0843) | 0.149 | 1 |  |
| Dobutamine | Increase | 0 (0-30.8) | 0 (0-14.5) | 0.151 | 1 | 0 (0-12.2) | 0 (0-4.06) | 0.72 | 1 | -0.0761 (-0.18-0.0945) | -0.0708 (-0.256-0.0593) | 0.545 | 1 |  |
| Midazolam | Increase | 5.26 (0-34.5) | 4 (0-36.9) | 0.958 | 1 | 0 (0-17.8) | 0 (0-23.9) | 0.635 | 1 | 0.0062 (-0.12-0.182) | 0.0146 (-0.101-0.172) | 0.961 | 1 |  |
| Midazolam | Decrease | 3.85 (0-32.1) | 6.45 (0-33.3) | 0.902 | 1 | 0 (0-13.3) | 0 (0-17.9) | 0.436 | 1 | 0.00711 (-0.131-0.154) | 0.0248 (-0.0953-0.17) | 0.297 | 1 |  |
| Morphine | Increase | 7.85 (0-39.3) | 10.5 (0-42.9) | 0.651 | 1 | 0 (0-24) | 0 (0-26.9) | 0.666 | 1 | 0.0241 (-0.121-0.208) | 0.0488 (-0.0942-0.222) | 0.245 | 1 |  |
| Morphine | Decrease | 10 (0-38.6) | 13.3 (0-44.8) | 0.258 | 1 | 0 (0-23.1) | 0 (0-27.9) | 0.198 | 1 | 0.0347 (-0.105-0.216) | 0.0456 (-0.1-0.222) | 0.676 | 1 |  |
| Noradrenaline | Increase | 3.7 (0-34.6) | 3.85 (0-34.8) | 0.724 | 1 | 0 (0-20) | 0 (0-19.4) | 0.819 | 1 | 0.00737 (-0.135-0.167) | 0.019 (-0.114-0.181) | 0.00382 | 0.103 |  |
| Noradrenaline | Decrease | 4.76 (0-38.5) | 6.67 (0-38.5) | 0.855 | 1 | 0 (0-22.2) | 0 (0-24) | 0.807 | 1 | 0.0176 (-0.116-0.199) | 0.0232 (-0.11-0.202) | 0.4 | 1 |  |
| Propofol | Increase | 7.14 (0-40.6) | 11.5 (0-46.4) | 0.0976 | 1 | 0 (0-24.8) | 0 (0-29) | 0.111 | 1 | 0.0376 (-0.0941-0.217) | 0.0393 (-0.0972-0.229) | 0.978 | 1 |  |
| Propofol | Decrease | 7.41 (0-39.3) | 8 (0-42.3) | 0.798 | 1 | 0 (0-22.2) | 0 (0-25) | 0.785 | 1 | 0.0234 (-0.111-0.175) | 0.0325 (-0.101-0.223) | 0.159 | 1 |  |
| Vasopressin | Increase | 0 (0-0) | 0 (0-0) | 1 | NaN | 0 (0-0) | 0 (0-0) | 1 | NaN | -0.0331 (-0.088--0.0156) | -0.117 (-0.214--0.104) | 0.0207 | 0.475 |  |
| **Name** | **Mean Dose Change** | **% time LPRx_60 > 0** | | | | **% time LPRx_60 > 0.25** | | | | **% time LPRx_60 > 0.35** | | | |  |
|  |  | **Pre Dose** | **Post Dose** | **P value** | **Adj P Value** | **Pre Dose** | **Post Dose** | **P value** | **Adj P Value** | **Pre Dose** | **Post Dose** | **P value** | **Adj P Value** |  |
| Dobutamine | Decrease | 39 (12-87.5) | 27.1 (0-85.4) | 0.0658 | 1 | 0 (0-21.2) | 0 (0-8.94) | 0.859 | 1 | 0 (0-0) | 0 (0-0) | 0.892 | 1 |  |
| Dobutamine | Increase | 27.3 (0-72.6) | 17.4 (0-80) | 0.181 | 1 | 0 (0-7.22) | 0 (0-0) | 0.55 | 1 | 0 (0-0) | 0 (0-0) | 0.898 | 1 |  |
| Midazolam | Increase | 50 (7.69-100) | 53.8 (10.9-100) | 0.561 | 1 | 0 (0-33.3) | 0 (0-37.3) | 0.537 | 1 | 0 (0-11.8) | 0 (0-10.3) | 0.478 | 1 |  |
| Midazolam | Decrease | 50 (8.33-96.3) | 60.7 (14.8-100) | 0.0681 | 1 | 0 (0-29.6) | 0 (0-27.6) | 0.41 | 1 | 0 (0-7.01) | 0 (0-3.85) | 0.244 | 1 |  |
| Morphine | Increase | 60.7 (11.4-100) | 64.5 (17.1-100) | 0.503 | 1 | 0 (0-42.5) | 0 (0-48.8) | 0.24 | 1 | 0 (0-15) | 0 (0-23.3) | 0.258 | 1 |  |
| Morphine | Decrease | 61.9 (14.3-100) | 64.3 (17.5-100) | 0.675 | 1 | 0 (0-48.6) | 0 (0-46.8) | 0.533 | 1 | 0 (0-20) | 0 (0-25) | 0.723 | 1 |  |
| Noradrenaline | Increase | 52.2 (6.9-96.4) | 57.7 (6.67-100) | 0.0111 | 0.299 | 0 (0-30.8) | 0 (0-31.8) | 0.278 | 1 | 0 (0-10) | 0 (0-4.55) | 0.106 | 1 |  |
| Noradrenaline | Decrease | 57.7 (9.64-100) | 60.5 (6.9-100) | 0.563 | 1 | 0 (0-39.4) | 0 (0-40.7) | 0.458 | 1 | 0 (0-16.7) | 0 (0-13.8) | 0.227 | 1 |  |
| Propofol | Increase | 65.5 (14.3-100) | 64.3 (16-100) | 0.557 | 1 | 0 (0-46.2) | 0 (0-48) | 0.619 | 1 | 0 (0-20.7) | 0 (0-21.4) | 0.973 | 1 |  |
| Propofol | Decrease | 57.1 (12-100) | 64.3 (11.5-100) | 0.124 | 1 | 0 (0-34.6) | 0 (0-42.1) | 0.397 | 1 | 0 (0-13.5) | 0 (0-20) | 0.339 | 1 |  |
| Vasopressin | Increase | 3.28 (2.42-3.36) | 0 (0-0) | 0.0558 | 1 | 0 (0-0) | 0 (0-0) | 0.382 | 1 | 0 (0-0) | 0 (0-0) | 1 | NaN |  |
| **Bolus** | | | | | | | | | | | | | |  |
| **Name** | **Doses** | **Mean MAP** | | | | **Mean CPP** | | | | **% time CPP>60** | | | |  |
|  |  | **Pre Dose** | **Post Dose** | **P value** | **Adj P Value** | **Pre Dose** | **Post Dose** | **P value** | **Adj P Value** | **Pre Dose** | **Post Dose** | **P value** | **Adj P Value** |  |
| Fentanyl | 12 | 90.6 (87.2-90.9) | 83.5 (82.2-86.7) | 0.0139 | 0.376 | 66.7 (64.5-68.8) | 66.9 (64.4-72.8) | 0.583 | 1 | 27 (20.2-32.1) | 12.2 (3.92-31.1) | 0.0989 | 1 |  |
| Midazolam | 193 | 83.8 (78.1-90.9) | 83 (78.2-90.1) | 0.57 | 1 | 61.5 (55.4-66.4) | 62.3 (56.8-68.8) | 0.195 | 1 | 40 (3.7-88.5) | 25 (0-87.9) | 0.16 | 1 |  |
| Morphine | 399 | 85.1 (78.1-92.4) | 82.7 (77.5-90.3) | 0.0126 | 0.34 | 62.7 (56-69.9) | 61.6 (55.8-69.2) | 0.479 | 1 | 32.4 (3.45-89.2) | 36 (0-90.2) | 0.973 | 1 |  |
| Propofol | 545 | 83.8 (78.7-89.8) | 83 (77.8-89.1) | 0.0363 | 0.98 | 62.3 (57-67.7) | 62.5 (56.5-68.9) | 0.622 | 1 | 33.3 (3.7-83.3) | 30.8 (0-84) | 0.629 | 1 |  |
| Alfentanil | 18 | 79.5 (77.1-85.9) | 80.2 (75.2-83.3) | 0.673 | 1 | 58 (55.3-62.6) | 59.7 (57.2-64) | 0.443 | 1 | 76.6 (30.5-95.2) | 68.4 (28.6-88.2) | 0.578 | 1 |  |
| **Name** | **% time CPP>70** | | | | **Mean ICP** | | | | **% time ICP>20** | | | |  |  |
|  | **Pre Dose** | **Post Dose** | **P value** | **Adj P Value** | **Pre Dose** | **Post Dose** | **P value** | **Adj P Value** | **Pre Dose** | **Post Dose** | **P value** | **Adj P Value** |  |  |
| Fentanyl | 32.2 (23.8-50) | 20 (3.85-45.6) | 0.75 | 1 | 21.8 (21.6-23.6) | 15.6 (10.3-16.8) | <0.0001 | 0.00095 | 68.9 (57.7-90.1) | 3.7 (0-12) | <0.0001 | 0.000836 |  |  |
| Midazolam | 2.78 (0-23.3) | 0.631 (0-40) | 0.789 | 1 | 22.2 (21-24) | 21.2 (18.4-23.4) | <0.0001 | 0.000105 | 93.1 (75-100) | 76.9 (13.9-100) | <0.0001 | <0.0001 |  |  |
| Morphine | 4.65 (0-42.9) | 2.38 (0-35.2) | 0.104 | 1 | 22.1 (20.9-24) | 21 (18.7-23.8) | <0.0001 | <0.0001 | 88 (69.5-100) | 74.2 (22.2-100) | <0.0001 | <0.0001 |  |  |
| Propofol | 4 (0-25) | 3.57 (0-37) | 0.822 | 1 | 21.6 (20.7-23.2) | 20.8 (18.9-22.9) | <0.0001 | <0.0001 | 84.6 (66.7-96.3) | 68.8 (22.5-95.1) | <0.0001 | <0.0001 |  |  |
| Alfentanil | 0 (0-18) | 1.67 (0-6.84) | 0.677 | 1 | 21.7 (21-23.1) | 19.7 (16-23.1) | 0.0549 | 1 | 76.3 (64.7-94.3) | 56.5 (8.36-92.9) | 0.162 | 1 |  |  |
| **Name** | **% time ICP>22** | | | | **Mean LPRx_10** | | | | **% time LPRx_10 > 0** | | | |  |  |
|  | **Pre Dose** | **Post Dose** | **P value** | **Adj P Value** | **Pre Dose** | **Post Dose** | **P value** | **Adj P Value** | **Pre Dose** | **Post Dose** | **P value** | **Adj P Value** |  |  |
| Fentanyl | 36.4 (17.9-68.5) | 0 (0-1.92) | <0.0001 | 0.00165 | 0.0996 (0.0318-0.193) | 0.0594 (0.0295-0.0929) | 0.37 | 1 | 61.8 (57.1-65.7) | 56.3 (50.4-60) | 0.0396 | 1 |  |  |
| Midazolam | 50 (18.5-88) | 31 (0-80.8) | 0.000154 | 0.00417 | 0.0338 (-0.0936-0.204) | 0.029 (-0.109-0.144) | 0.168 | 1 | 55.6 (36-75) | 51.7 (37.5-65) | 0.193 | 1 |  |  |
| Morphine | 48.7 (21.4-84.7) | 23.1 (2.48-86.9) | <0.0001 | 0.00013 | 0.0514 (-0.115-0.226) | 0.059 (-0.102-0.188) | 0.724 | 1 | 56 (34.5-75) | 53.8 (36.4-71.2) | 0.764 | 1 |  |  |
| Propofol | 36.7 (17.2-73.1) | 20 (2.5-66.7) | <0.0001 | <0.0001 | 0.032 (-0.127-0.163) | 0.0148 (-0.125-0.161) | 0.565 | 1 | 53.6 (33.3-70.4) | 51.2 (32.1-69.2) | 0.449 | 1 |  |  |
| Alfentanil | 39.6 (17.3-66.7) | 13.9 (0-64) | 0.123 | 1 | 0.0288 (-0.185-0.137) | -0.0101 (-0.0884-0.162) | 0.938 | 1 | 45.4 (27.7-62.9) | 48.6 (35.1-62.9) | 0.849 | 1 |  |  |
| **Name** | **% time LPRx_10 > 0.25** | | | | **% time LPRx_10 > 0.35** | | | | **Mean LPRx_15** | | | |  |  |
|  | **Pre Dose** | **Post Dose** | **P value** | **Adj P Value** | **Pre Dose** | **Post Dose** | **P value** | **Adj P Value** | **Pre Dose** | **Post Dose** | **P value** | **Adj P Value** |  |  |
| Fentanyl | 46 (36.4-47.6) | 35 (31.4-46.2) | 0.434 | 1 | 29.7 (12.5-39.3) | 29.8 (22.9-30.8) | 0.839 | 1 | 0.0854 (0.0612-0.154) | 0.0471 (-0.0693-0.0932) | 0.0526 | 1 |  |  |
| Midazolam | 30.8 (12.5-46.4) | 26.9 (10.7-42.6) | 0.262 | 1 | 19.2 (4.55-39.1) | 17.9 (4.35-34.6) | 0.422 | 1 | 0.0402 (-0.112-0.186) | 0.0208 (-0.105-0.136) | 0.269 | 1 |  |  |
| Morphine | 28.6 (13-50) | 29.6 (14.3-48) | 0.748 | 1 | 19.2 (4-39.2) | 20.7 (7.14-38.3) | 0.524 | 1 | 0.0434 (-0.111-0.212) | 0.0505 (-0.0968-0.19) | 0.973 | 1 |  |  |
| Propofol | 25 (11.1-43.3) | 26.7 (11.1-42.5) | 0.529 | 1 | 15.4 (3.85-32.1) | 17.8 (4.17-33.3) | 0.604 | 1 | 0.0163 (-0.121-0.161) | 0.0265 (-0.119-0.16) | 0.998 | 1 |  |  |
| Alfentanil | 28.2 (10.6-38.9) | 29 (17.7-45.8) | 0.874 | 1 | 14.9 (4.44-28.6) | 15.8 (10.6-40.7) | 0.496 | 1 | 0.0337 (-0.126-0.154) | 0.0385 (-0.0725-0.271) | 0.815 | 1 |  |  |
| **Name** | **% time LPRx_15 > 0** | | | | **% time LPRx_15 > 0.25** | | | | **% time LPRx_15 > 0.35** | | | |  |  |
|  | **Pre Dose** | **Post Dose** | **P value** | **Adj P Value** | **Pre Dose** | **Post Dose** | **P value** | **Adj P Value** | **Pre Dose** | **Post Dose** | **P value** | **Adj P Value** |  |  |
| Fentanyl | 66 (53.6-73.1) | 53.6 (42.9-58.8) | 0.00847 | 0.229 | 34.6 (19-38.9) | 23.3 (15.6-38.5) | 0.707 | 1 | 19.2 (10.7-28.7) | 20 (11.1-30.8) | 0.931 | 1 |  |  |
| Midazolam | 53.8 (31.4-74.1) | 50 (34.8-67.9) | 0.427 | 1 | 21.4 (4.35-48) | 21.9 (4.55-41.7) | 0.799 | 1 | 10.7 (0-37) | 13.3 (0-33.3) | 0.87 | 1 |  |  |
| Morphine | 56.7 (33.3-77.5) | 55.6 (35.7-74) | 0.867 | 1 | 24.3 (3.92-50) | 26.1 (6.86-47.9) | 0.507 | 1 | 13.6 (0-37.3) | 16 (0-36.7) | 0.162 | 1 |  |  |
| Propofol | 54.2 (32.1-73.1) | 52.5 (33.3-72) | 0.674 | 1 | 19.2 (3.45-40.7) | 23.1 (4.17-42.3) | 0.12 | 1 | 9.27 (0-28) | 11.5 (0-28.6) | 0.184 | 1 |  |  |
| Alfentanil | 55.4 (32.2-72.9) | 51 (24.7-84.6) | 0.924 | 1 | 16 (3.39-38.9) | 30.7 (15.8-56.2) | 0.247 | 1 | 7.74 (0-28.7) | 16.9 (0.833-42.1) | 0.411 | 1 |  |  |
| **Name** | **Mean LPRx_20** | | | | **% time LPRx_20 > 0** | | | | **% time LPRx_20 > 0.25** | | | |  |  |
|  | **Pre Dose** | **Post Dose** | **P value** | **Adj P Value** | **Pre Dose** | **Post Dose** | **P value** | **Adj P Value** | **Pre Dose** | **Post Dose** | **P value** | **Adj P Value** |  |  |
| Fentanyl | 0.131 (0.109-0.194) | -0.00215 (-0.0698-0.0299) | 0.000878 | 0.0237 | 66.5 (53.8-81.9) | 46.2 (37.1-60) | 0.00196 | 0.053 | 31.6 (14.3-44.6) | 14.1 (11.5-30.8) | 0.174 | 1 |  |  |
| Midazolam | 0.0341 (-0.105-0.201) | 0.0168 (-0.112-0.129) | 0.272 | 1 | 56 (27.9-76.9) | 52 (32.1-68) | 0.218 | 1 | 17.9 (0-45.8) | 18.5 (0-41.4) | 0.449 | 1 |  |  |
| Morphine | 0.0543 (-0.11-0.215) | 0.0597 (-0.104-0.205) | 0.922 | 1 | 57.1 (33.3-84) | 57.1 (33.3-79.3) | 0.745 | 1 | 20 (0-50) | 23.8 (0-46.4) | 0.382 | 1 |  |  |
| Propofol | 0.0172 (-0.113-0.156) | 0.0258 (-0.0988-0.149) | 0.75 | 1 | 53.8 (31.6-76.2) | 53.8 (33.3-74.4) | 0.937 | 1 | 14.8 (0-38.5) | 20 (0-39.3) | 0.347 | 1 |  |  |
| Alfentanil | 0.0668 (-0.0806-0.11) | 0.00838 (-0.0773-0.278) | 0.938 | 1 | 57.6 (29.1-77.9) | 52.6 (27.5-82.6) | 1 | 1 | 15.2 (0.833-35.7) | 20 (8.69-57.8) | 0.279 | 1 |  |  |
| **Name** | **% time LPRx_20 > 0.35** | | | | **Mean LPRx_30** | | | | **% time LPRx_30 > 0** | | | |  |  |
|  | **Pre Dose** | **Post Dose** | **P value** | **Adj P Value** | **Pre Dose** | **Post Dose** | **P value** | **Adj P Value** | **Pre Dose** | **Post Dose** | **P value** | **Adj P Value** |  |  |
| Fentanyl | 23.1 (7.14-31.7) | 11.4 (5.77-16.8) | 0.172 | 1 | 0.184 (0.00433-0.311) | 0.0516 (-0.108-0.115) | 0.04 | 1 | 97.3 (39.3-100) | 46.5 (32.4-51) | 0.0218 | 0.588 |  |  |
| Midazolam | 6.98 (0-32) | 8.33 (0-30) | 0.576 | 1 | 0.0225 (-0.108-0.211) | -0.00281 (-0.112-0.166) | 0.296 | 1 | 54.3 (22.7-85.7) | 50 (25.8-76) | 0.238 | 1 |  |  |
| Morphine | 7.69 (0-32) | 12 (0-34.1) | 0.308 | 1 | 0.0448 (-0.113-0.24) | 0.0561 (-0.11-0.226) | 0.775 | 1 | 59.3 (28-91.3) | 61.9 (30.4-88.7) | 0.843 | 1 |  |  |
| Propofol | 4 (0-23.9) | 7.41 (0-25.9) | 0.446 | 1 | 0.0137 (-0.112-0.165) | 0.0161 (-0.0986-0.174) | 0.618 | 1 | 51.9 (26.7-83.3) | 55.3 (29-83.3) | 0.541 | 1 |  |  |
| Alfentanil | 5.65 (0-23.8) | 11 (0.862-31.6) | 0.468 | 1 | 0.0429 (-0.0454-0.148) | 0.0331 (-0.075-0.302) | 0.888 | 1 | 52.6 (30.5-73.6) | 52.4 (37-91.1) | 0.751 | 1 |  |  |
| **Name** | **% time LPRx_30 > 0.25** | | | | **% time LPRx_30 > 0.35** | | | | **Mean LPRx_60** | | | |  |  |
|  | **Pre Dose** | **Post Dose** | **P value** | **Adj P Value** | **Pre Dose** | **Post Dose** | **P value** | **Adj P Value** | **Pre Dose** | **Post Dose** | **P value** | **Adj P Value** |  |  |
| Fentanyl | 36.1 (7.14-64.7) | 24.4 (7.5-40) | 0.338 | 1 | 26.8 (2.38-55.6) | 11.3 (2.5-24.4) | 0.338 | 1 | 0.131 (-0.0576-0.316) | 0.311 (0.0166-0.469) | 0.174 | 1 |  |  |
| Midazolam | 10.3 (0-47.4) | 8.7 (0-38.5) | 0.545 | 1 | 0 (0-21.4) | 0 (0-25) | 0.736 | 1 | 0.0538 (-0.165-0.249) | 0.00631 (-0.168-0.188) | 0.241 | 1 |  |  |
| Morphine | 14.3 (0-53.5) | 16.7 (0-50) | 0.855 | 1 | 0 (0-27.4) | 3.45 (0-35.4) | 0.382 | 1 | 0.0765 (-0.127-0.296) | 0.048 (-0.145-0.247) | 0.328 | 1 |  |  |
| Propofol | 9.09 (0-38.5) | 8.33 (0-37.8) | 0.902 | 1 | 0 (0-17.1) | 0 (0-20) | 0.928 | 1 | 0.0319 (-0.126-0.22) | 0.0274 (-0.0942-0.171) | 0.946 | 1 |  |  |
| Alfentanil | 17 (0-35.3) | 19 (1.92-71.4) | 0.422 | 1 | 11.5 (0-20.8) | 12.3 (0-44.4) | 0.685 | 1 | 0.0869 (0.00725-0.229) | 0.211 (0.0744-0.346) | 0.389 | 1 |  |  |
| **Name** | **% time LPRx_60 > 0** | | | | **% time LPRx_60 > 0.25** | | | | **% time LPRx_60 > 0.35** | | | |  |  |
|  | **Pre Dose** | **Post Dose** | **P value** | **Adj P Value** | **Pre Dose** | **Post Dose** | **P value** | **Adj P Value** | **Pre Dose** | **Post Dose** | **P value** | **Adj P Value** |  |  |
| Fentanyl | 72 (11.5-100) | 90 (73.1-100) | 0.261 | 1 | 32.7 (3.85-87) | 71.8 (2.78-100) | 0.466 | 1 | 5.41 (0-43.8) | 46.3 (0-86.7) | 0.177 | 1 |  |  |
| Midazolam | 69.6 (0-100) | 50 (0-100) | 0.349 | 1 | 0 (0-51.7) | 0 (0-35.7) | 0.356 | 1 | 0 (0-27.3) | 0 (0-14.3) | 0.442 | 1 |  |  |
| Morphine | 70.6 (13.9-100) | 62.1 (7.06-100) | 0.518 | 1 | 3.45 (0-66.1) | 0 (0-55.5) | 0.314 | 1 | 0 (0-40.4) | 0 (0-27.5) | 0.298 | 1 |  |  |
| Propofol | 61.5 (12.5-100) | 58.3 (16.3-100) | 0.891 | 1 | 0 (0-44.8) | 0 (0-33.3) | 0.172 | 1 | 0 (0-22.2) | 0 (0-7.69) | 0.0356 | 0.961 |  |  |
| Alfentanil | 80 (45-100) | 100 (69.3-100) | 0.164 | 1 | 23.3 (1.67-42.1) | 29 (1.92-82.4) | 0.597 | 1 | 1.67 (0-30.4) | 0 (0-51.3) | 0.835 | 1 |  |  |

*The table demonstrates the median and interquartile range of the pre/pose dose windows as well as the Wilcox signed ranked test between these windows with p values adjusted using the Bonferroni analysis. The “change” indicates if the continuous infusion was increase/decrease or was a bolus dose. CPP, cerebral prefusion pressure; ICP, intracranial pressure; LPRx_10, pressure reactivity over 10 minutes; LPRx_15, pressure reactivity over 15 minutes; LPRx_20, pressure reactivity over 20 minutes; LPRx_30, pressure reactivity over 30 minutes; LPRx_60, pressure reactivity over 60 minutes;*

# Appendix H. Pre-time window over 50% time ICP < 20 mmHg

The table contains all infusions given with the ICP pre-time window 50% < 20 mmHg, separated into the continuous infusion doses then the bolus doses.

| **Continuous Intravenous** | | | | | | | | | | | | | | |
| --- | --- | --- | --- | --- | --- | --- | --- | --- | --- | --- | --- | --- | --- | --- |
| **Name** | **Doses** | **Mean Dose Change** | **Mean MAP** | | | | **Mean CPP** | | | | **% time CPP>60** | | | |
|  |  |  | **Pre Dose** | **Post Dose** | **P value** | **Adj P Value** | **Pre Dose** | **Post Dose** | **P value** | **Adj P Value** | **Pre Dose** | **Post Dose** | **P value** | **Adj P Value** |
| Dobutamine | 1410 | Decrease | 76.4 (70.8-83.3) | 75.5 (70.6-82.2) | 0.0761 | 1 | 64.7 (58.9-70.6) | 63.4 (58.9-69.4) | 0.0194 | 0.523 | 11.5 (0-66.7) | 16.4 (0-71.8) | 0.0427 | 1 |
| Dobutamine | 1121 | Increase | 72.9 (67.9-79.5) | 72.5 (67.9-78.9) | 0.436 | 1 | 60.1 (54.3-66.7) | 60 (54.5-65.7) | 0.621 | 1 | 52.9 (8.57-100) | 57.1 (7.14-100) | 0.783 | 1 |
| Midazolam | 4978 | Decrease | 78.1 (72.9-84) | 77.8 (72.9-83.9) | 0.413 | 1 | 65.4 (60.1-71.4) | 65 (60-71.2) | 0.271 | 1 | 7.69 (0-51.7) | 8.33 (0-53.4) | 0.386 | 1 |
| Midazolam | 4626 | Increase | 78.4 (73.1-84.5) | 78 (72.8-84) | 0.0305 | 0.825 | 64.9 (59.6-71.3) | 64.7 (59.4-70.9) | 0.148 | 1 | 10.7 (0-57.7) | 10 (0-60) | 0.731 | 1 |
| Morphine | 7695 | Decrease | 77.4 (72.2-83.7) | 77.2 (72-83.5) | 0.265 | 1 | 65.8 (60.2-72) | 65.5 (59.9-71.7) | 0.0637 | 1 | 7.14 (0-51.7) | 7.69 (0-55.6) | 0.146 | 1 |
| Morphine | 7330 | Increase | 77.8 (72.4-84.5) | 77.1 (71.8-83.5) | <0.0001 | <0.0001 | 65.9 (60-72.4) | 65.1 (59.6-71.5) | <0.0001 | 0.000585 | 7.41 (0-53.6) | 7.69 (0-59.3) | 0.0813 | 1 |
| Noradrenaline | 49821 | Decrease | 78.7 (73.3-85) | 78.1 (72.8-84.3) | <0.0001 | <0.0001 | 66.5 (60.9-72.9) | 65.8 (60.5-72.1) | <0.0001 | <0.0001 | 6.67 (0-42.9) | 6.9 (0-46.7) | 0.000135 | 0.00364 |
| Noradrenaline | 46053 | Increase | 76.1 (70.8-82.4) | 76.1 (70.9-82.4) | 0.122 | 1 | 63.4 (58.2-69.7) | 63.8 (58.6-70) | <0.0001 | <0.0001 | 23.1 (0-74.1) | 17.2 (0-72) | <0.0001 | <0.0001 |
| Propofol | 15631 | Decrease | 77.3 (71.8-83.8) | 77.1 (71.8-83.5) | 0.121 | 1 | 65.5 (59.9-72) | 65.2 (59.6-71.8) | 0.00204 | 0.055 | 8 (0-55.2) | 10.3 (0-58.6) | 0.0509 | 1 |
| Propofol | 13936 | Increase | 78.3 (72.7-85) | 77.3 (71.9-83.7) | <0.0001 | <0.0001 | 66.2 (60.4-72.9) | 65.3 (59.8-71.9) | <0.0001 | <0.0001 | 7.41 (0-50) | 8 (0-55.6) | 0.000106 | 0.00286 |
| Vasopressin | 78 | Decrease | 81.3 (73.7-84.1) | 80.1 (71.9-82.7) | 0.0872 | 1 | 57.5 (55.2-59.8) | 58.7 (55.6-59.6) | 0.936 | 1 | 66 (58.9-78.6) | 60.8 (55.3-96.8) | 0.884 | 1 |
| Vasopressin | 58 | Increase | 79.2 (69-84.1) | 80.1 (69.7-82.7) | 0.493 | 1 | 56 (52.4-58.4) | 57 (51.2-59.1) | 0.293 | 1 | 78.2 (62.1-100) | 70.2 (60.8-100) | 0.472 | 1 |
| **Name** | **Mean Dose Change** | **% time CPP>70** | | | | **Mean ICP** | | | | **% time ICP>20** | | | |  |
|  |  | **Pre Dose** | **Post Dose** | **P value** | **Adj P Value** | **Pre Dose** | **Post Dose** | **P value** | **Adj P Value** | **Pre Dose** | **Post Dose** | **P value** | **Adj P Value** |  |
| Dobutamine | Decrease | 5.06 (0-51.8) | 3.45 (0-38.5) | 0.00133 | 0.0359 | 13.1 (9.16-16) | 13.1 (9.39-15.9) | 0.752 | 1 | 0 (0-2.5) | 0 (0-0) | 0.00137 | 0.0369 |  |
| Dobutamine | Increase | 0 (0-20) | 0 (0-12) | 0.0741 | 1 | 13.9 (10.2-16.6) | 13.8 (10.3-16.5) | 0.595 | 1 | 0 (0-4.17) | 0 (0-0) | 0.00219 | 0.0592 |  |
| Midazolam | Decrease | 7.69 (0-58.6) | 7.14 (0-59.3) | 0.189 | 1 | 13.1 (9.77-15.9) | 13 (9.61-15.8) | 0.833 | 1 | 0 (0-2.48) | 0 (0-2.47) | 0.763 | 1 |  |
| Midazolam | Increase | 7.32 (0-57.1) | 6.45 (0-55.5) | 0.0304 | 0.822 | 13.7 (10.3-16.6) | 13.5 (10.1-16.4) | 0.118 | 1 | 0 (0-4.32) | 0 (0-3.57) | 0.000134 | 0.00363 |  |
| Morphine | Decrease | 10.3 (0-63.5) | 8 (0-62.1) | 0.059 | 1 | 12.1 (8.47-15.2) | 12.1 (8.57-15.2) | 0.422 | 1 | 0 (0-0) | 0 (0-0) | 0.31 | 1 |  |
| Morphine | Increase | 11.1 (0-65.5) | 7.14 (0-60.7) | <0.0001 | <0.0001 | 12.4 (8.84-15.6) | 12.2 (8.58-15.5) | 0.0831 | 1 | 0 (0-2.33) | 0 (0-0) | 0.000331 | 0.00895 |  |
| Noradrenaline | Decrease | 14.3 (0-72.4) | 10 (0-66.7) | <0.0001 | <0.0001 | 12.5 (9.07-15.6) | 12.5 (9.12-15.5) | 0.83 | 1 | 0 (0-3.33) | 0 (0-0) | <0.0001 | <0.0001 |  |
| Noradrenaline | Increase | 4.35 (0-40.7) | 3.85 (0-44.4) | 0.000157 | 0.00424 | 13 (9.52-16) | 12.7 (9.13-15.8) | <0.0001 | <0.0001 | 0 (0-3.7) | 0 (0-0) | <0.0001 | <0.0001 |  |
| Propofol | Decrease | 8.33 (0-63) | 7.41 (0-61.5) | 0.000882 | 0.0238 | 12 (8.39-15.4) | 12.1 (8.43-15.5) | 0.0801 | 1 | 0 (0-0.123) | 0 (0-0.408) | 0.118 | 1 |  |
| Propofol | Increase | 13 (0-70) | 7.69 (0-63) | <0.0001 | <0.0001 | 12.4 (8.73-15.8) | 12.2 (8.51-15.6) | 0.00286 | 0.0772 | 0 (0-3.7) | 0 (0-0.373) | <0.0001 | <0.0001 |  |
| Vasopressin | Decrease | 6.14 (0-6.81) | 5.53 (0-6.74) | 0.0125 | 0.337 | 15.2 (13.9-17.1) | 14 (12.8-16.9) | 0.137 | 1 | 7.22 (0-11.5) | 6.07 (0-8.87) | 0.343 | 1 |  |
| Vasopressin | Increase | 2.66 (0-6.81) | 5.53 (0-6.74) | 0.658 | 1 | 15.8 (15-18.2) | 16.7 (13.9-18) | 0.868 | 1 | 7.22 (0-11.5) | 7.22 (0-10.9) | 0.873 | 1 |  |
| **Name** | **Mean Dose Change** | **% time ICP>22** | | | | **Mean LPRx_10** | | | | **% time LPRx_10 > 0** | | | |  |
|  |  | **Pre Dose** | **Post Dose** | **P value** | **Adj P Value** | **Pre Dose** | **Post Dose** | **P value** | **Adj P Value** | **Pre Dose** | **Post Dose** | **P value** | **Adj P Value** |  |
| Dobutamine | Decrease | 0 (0-0) | 0 (0-0) | 0.0173 | 0.467 | 0.0106 (-0.154-0.169) | 0.0201 (-0.142-0.163) | 0.627 | 1 | 50 (30.5-68.8) | 51.1 (32.9-68.4) | 0.57 | 1 |  |
| Dobutamine | Increase | 0 (0-0) | 0 (0-0) | 0.165 | 1 | 0.00536 (-0.165-0.154) | 0.0188 (-0.158-0.164) | 0.312 | 1 | 50 (31.6-68.2) | 50 (30.8-69.2) | 0.618 | 1 |  |
| Midazolam | Decrease | 0 (0-0) | 0 (0-0) | 0.955 | 1 | 0.00797 (-0.156-0.155) | 0.00619 (-0.156-0.155) | 0.769 | 1 | 50 (31-67.9) | 50 (32-67.7) | 0.832 | 1 |  |
| Midazolam | Increase | 0 (0-0) | 0 (0-0) | 0.00419 | 0.113 | 0.0121 (-0.15-0.161) | 0.00302 (-0.159-0.151) | 0.0792 | 1 | 50.5 (31-69) | 50 (31.8-67.7) | 0.238 | 1 |  |
| Morphine | Decrease | 0 (0-0) | 0 (0-0) | 0.0993 | 1 | 0.00622 (-0.147-0.153) | 0.00785 (-0.14-0.152) | 0.384 | 1 | 50 (32.1-67.9) | 50 (33.3-67.9) | 0.599 | 1 |  |
| Morphine | Increase | 0 (0-0) | 0 (0-0) | 0.0021 | 0.0566 | 0.00508 (-0.151-0.16) | 0.00294 (-0.153-0.154) | 0.304 | 1 | 50 (30.8-69.2) | 50 (32.1-67.6) | 0.473 | 1 |  |
| Noradrenaline | Decrease | 0 (0-0) | 0 (0-0) | <0.0001 | <0.0001 | 0.00535 (-0.153-0.154) | 0.00573 (-0.153-0.152) | 0.358 | 1 | 50 (32-67.9) | 50 (31.2-67.9) | 0.644 | 1 |  |
| Noradrenaline | Increase | 0 (0-0) | 0 (0-0) | <0.0001 | <0.0001 | 0.00498 (-0.156-0.156) | 0.000554 (-0.159-0.152) | 0.00768 | 0.207 | 50 (31-68) | 50 (31-67.9) | 0.0229 | 0.619 |  |
| Propofol | Decrease | 0 (0-0) | 0 (0-0) | 0.107 | 1 | 0.00466 (-0.15-0.156) | 0.0084 (-0.145-0.158) | 0.109 | 1 | 50 (32-68) | 50 (33.3-68) | 0.196 | 1 |  |
| Propofol | Increase | 0 (0-0) | 0 (0-0) | <0.0001 | <0.0001 | 0.00596 (-0.149-0.157) | 0.00349 (-0.155-0.153) | 0.225 | 1 | 50 (32-68.4) | 50 (32-67.9) | 0.482 | 1 |  |
| Vasopressin | Decrease | 1.07 (0-1.7) | 0.902 (0-1.3) | 0.443 | 1 | 0.0326 (0.0108-0.117) | 0.0188 (0.00906-0.0533) | 0.0112 | 0.301 | 53.7 (51.7-58.1) | 53.3 (51.2-54.7) | 0.132 | 1 |  |
| Vasopressin | Increase | 1.19 (0-1.7) | 1.07 (0-2.07) | 0.916 | 1 | 0.0326 (0.0108-0.0587) | 0.0188 (0.00108-0.0376) | 0.16 | 1 | 53.7 (50.4-59) | 52.6 (47.7-54.2) | 0.145 | 1 |  |
| **Name** | **Mean Dose Change** | **% time LPRx_10 > 0.25** | | | | **% time LPRx_10 > 0.35** | | | | **Mean LPRx_15** | | | |  |
|  |  | **Pre Dose** | **Post Dose** | **P value** | **Adj P Value** | **Pre Dose** | **Post Dose** | **P value** | **Adj P Value** | **Pre Dose** | **Post Dose** | **P value** | **Adj P Value** |  |
| Dobutamine | Decrease | 26.4 (9.41-44.4) | 26.9 (10-43.5) | 0.706 | 1 | 17.3 (4-34.8) | 18.7 (3.7-34.6) | 0.904 | 1 | 0.00277 (-0.155-0.161) | 0.000788 (-0.155-0.158) | 0.994 | 1 |  |
| Dobutamine | Increase | 25 (10.3-40.8) | 26.9 (10.7-42.9) | 0.172 | 1 | 17.9 (3.7-32.1) | 17.4 (3.85-33.3) | 0.409 | 1 | -0.00224 (-0.158-0.143) | 0.00801 (-0.162-0.16) | 0.55 | 1 |  |
| Midazolam | Decrease | 25 (8.46-42.3) | 25 (8-42.3) | 0.594 | 1 | 16.7 (3.7-33.3) | 16.7 (3.57-33.3) | 0.432 | 1 | 0.00262 (-0.14-0.137) | -0.0019 (-0.148-0.139) | 0.395 | 1 |  |
| Midazolam | Increase | 25 (8-42.9) | 25 (8-41.9) | 0.327 | 1 | 16.2 (3.57-33.3) | 15.4 (3.57-33.3) | 0.312 | 1 | 0.0112 (-0.142-0.143) | -0.00229 (-0.152-0.135) | 0.0244 | 0.657 |  |
| Morphine | Decrease | 25 (9.09-41.4) | 25 (10-42.3) | 0.558 | 1 | 16 (3.7-32.1) | 16.7 (3.57-33.3) | 0.566 | 1 | 0.00573 (-0.137-0.141) | 0.00499 (-0.134-0.147) | 0.338 | 1 |  |
| Morphine | Increase | 25 (7.69-42.3) | 25 (9.3-42.3) | 0.356 | 1 | 15.4 (3.45-33.3) | 16 (3.57-32.1) | 0.12 | 1 | 0.00647 (-0.142-0.149) | 0.00269 (-0.139-0.143) | 0.291 | 1 |  |
| Noradrenaline | Decrease | 24.4 (8.82-42.3) | 25 (8.7-41.9) | 0.602 | 1 | 16 (3.57-32.3) | 16.1 (3.57-32.1) | 0.495 | 1 | -1.87e-05 (-0.148-0.141) | -0.000992 (-0.149-0.136) | 0.0742 | 1 |  |
| Noradrenaline | Increase | 24.1 (8-42.3) | 24.1 (9.09-41.9) | 0.895 | 1 | 16 (3.57-33.3) | 16 (3.57-32.3) | 0.747 | 1 | 0.00127 (-0.147-0.144) | -0.00677 (-0.155-0.132) | <0.0001 | <0.0001 |  |
| Propofol | Decrease | 25 (10-42.8) | 25 (10.3-42.9) | 0.135 | 1 | 16 (3.7-33.3) | 16.7 (3.7-33.3) | 0.053 | 1 | 0.00688 (-0.143-0.149) | 0.00946 (-0.135-0.154) | 0.0248 | 0.669 |  |
| Propofol | Increase | 25 (9.09-42.3) | 25 (10-42.3) | 0.934 | 1 | 16 (3.57-33.3) | 16.7 (3.57-33.3) | 0.433 | 1 | 0.00673 (-0.141-0.151) | 0.00493 (-0.142-0.146) | 0.5 | 1 |  |
| Vasopressin | Decrease | 30.5 (28.3-36.9) | 29.6 (27.8-30) | 0.00298 | 0.0806 | 23.4 (21.1-32.3) | 22.3 (19.4-23) | 0.00232 | 0.0627 | 0.0375 (0.017-0.0995) | 0.0248 (0.017-0.0446) | 0.0743 | 1 |  |
| Vasopressin | Increase | 30.5 (28.3-32.6) | 29.6 (25.1-31.1) | 0.237 | 1 | 23.4 (21.1-27.9) | 22.3 (18.9-24.1) | 0.168 | 1 | 0.0375 (0.017-0.07) | 0.0171 (-0.0467-0.0489) | 0.153 | 1 |  |
| **Name** | **Mean Dose Change** | **% time LPRx_15 > 0** | | | | **% time LPRx_15 > 0.25** | | | | **% time LPRx_15 > 0.35** | | | |  |
|  |  | **Pre Dose** | **Post Dose** | **P value** | **Adj P Value** | **Pre Dose** | **Post Dose** | **P value** | **Adj P Value** | **Pre Dose** | **Post Dose** | **P value** | **Adj P Value** |  |
| Dobutamine | Decrease | 50 (28-70.8) | 50 (28-70.3) | 0.955 | 1 | 21.4 (3.85-41.4) | 21.4 (3.57-41.7) | 0.795 | 1 | 11.5 (0-30) | 11.5 (0-30.8) | 0.838 | 1 |  |
| Dobutamine | Increase | 50 (27.6-70.4) | 50 (25.8-71.4) | 0.92 | 1 | 19.2 (3.85-38.5) | 21.4 (3.85-41.4) | 0.236 | 1 | 9.8 (0-28) | 12 (0-28.6) | 0.197 | 1 |  |
| Midazolam | Decrease | 50 (29.4-69) | 50 (27.6-69) | 0.546 | 1 | 19.2 (3.45-38.5) | 18.2 (3.23-37.9) | 0.188 | 1 | 9.3 (0-26.9) | 8.7 (0-26.9) | 0.456 | 1 |  |
| Midazolam | Increase | 51 (28.6-70.4) | 50 (28-68) | 0.192 | 1 | 19.2 (2.58-38.6) | 18.5 (3.23-37.9) | 0.278 | 1 | 8.33 (0-27.6) | 8.33 (0-26.9) | 0.531 | 1 |  |
| Morphine | Decrease | 50 (30-69.2) | 50 (30.8-69.2) | 0.648 | 1 | 19.8 (3.57-38.5) | 20 (3.57-39.1) | 0.286 | 1 | 10.2 (0-27.6) | 10.3 (0-27.6) | 0.31 | 1 |  |
| Morphine | Increase | 50 (28.6-72) | 50 (30-69.2) | 0.356 | 1 | 18.5 (0-39.1) | 20 (3.57-38.9) | 0.108 | 1 | 8.7 (0-27.6) | 10.4 (0-27.6) | 0.0876 | 1 |  |
| Noradrenaline | Decrease | 50 (28.6-69.2) | 50 (28.6-69) | 0.136 | 1 | 18.5 (3.45-38.5) | 18.5 (3.33-37.9) | 0.0487 | 1 | 8.57 (0-27.3) | 8.7 (0-26.9) | 0.114 | 1 |  |
| Noradrenaline | Increase | 50 (28.6-70) | 48.4 (28-68) | <0.0001 | <0.0001 | 18.5 (3.23-38.5) | 18.2 (3.33-37.5) | 0.026 | 0.702 | 8.33 (0-27.6) | 8.16 (0-26.7) | 0.0523 | 1 |  |
| Propofol | Decrease | 50 (30-70.4) | 51.7 (30.8-70.7) | 0.0577 | 1 | 20 (3.7-40) | 20.7 (3.7-40.7) | 0.024 | 0.647 | 10.3 (0-28.6) | 10.7 (0-29.6) | 0.0365 | 0.985 |  |
| Propofol | Increase | 50 (29.2-70.8) | 50 (30-70) | 0.916 | 1 | 19.2 (3.57-40) | 20 (3.7-39.3) | 0.147 | 1 | 9.68 (0-28.6) | 10.3 (0-28.6) | 0.207 | 1 |  |
| Vasopressin | Decrease | 54.8 (51.9-59.2) | 53.4 (52.1-57.3) | 0.266 | 1 | 27.6 (25.1-33.1) | 26.7 (23.5-30.1) | 0.0468 | 1 | 18.9 (16.5-22.3) | 17.8 (15.6-19.7) | 0.0338 | 0.912 |  |
| Vasopressin | Increase | 54.8 (52.1-61) | 52.3 (48-54.8) | 0.0339 | 0.914 | 27.3 (24.3-30.4) | 26.7 (16.7-30.3) | 0.254 | 1 | 18.9 (16.2-22.2) | 17.2 (9-22.3) | 0.296 | 1 |  |
| **Name** | **Mean Dose Change** | **Mean LPRx_20** | | | | **% time LPRx_20 > 0** | | | | **% time LPRx_20 > 0.25** | | | |  |
|  |  | **Pre Dose** | **Post Dose** | **P value** | **Adj P Value** | **Pre Dose** | **Post Dose** | **P value** | **Adj P Value** | **Pre Dose** | **Post Dose** | **P value** | **Adj P Value** |  |
| Dobutamine | Decrease | 0.002 (-0.161-0.153) | 0.000706 (-0.168-0.154) | 0.48 | 1 | 50 (24.1-73.1) | 50 (24-72) | 0.484 | 1 | 16.7 (0-41.6) | 16.7 (0-40) | 0.292 | 1 |  |
| Dobutamine | Increase | 0.00343 (-0.15-0.142) | 0.00185 (-0.175-0.152) | 0.736 | 1 | 51.4 (24-73.1) | 50 (23.8-73.1) | 0.688 | 1 | 15.4 (0-38.5) | 17.4 (0-39.5) | 0.663 | 1 |  |
| Midazolam | Decrease | 0.000627 (-0.133-0.13) | -0.00562 (-0.139-0.127) | 0.355 | 1 | 50 (26.7-71.4) | 48.3 (25.8-71) | 0.246 | 1 | 13.8 (0-35.7) | 13.3 (0-35.4) | 0.402 | 1 |  |
| Midazolam | Increase | 0.00833 (-0.133-0.14) | -0.000323 (-0.143-0.128) | 0.0304 | 0.822 | 51 (25.9-73.3) | 50 (25.9-71) | 0.0588 | 1 | 13.8 (0-36) | 13.8 (0-34.8) | 0.367 | 1 |  |
| Morphine | Decrease | 0.00417 (-0.13-0.137) | 0.00457 (-0.126-0.141) | 0.443 | 1 | 50.1 (28-72) | 50.2 (29-72) | 0.623 | 1 | 15.4 (0-36) | 15.4 (0-37.5) | 0.83 | 1 |  |
| Morphine | Increase | 0.00732 (-0.138-0.146) | 0.00531 (-0.135-0.139) | 0.428 | 1 | 51.7 (26.7-75) | 50.6 (28.6-71.4) | 0.199 | 1 | 14.3 (0-37) | 15.4 (0-37) | 0.143 | 1 |  |
| Noradrenaline | Decrease | -0.00249 (-0.144-0.133) | -0.005 (-0.145-0.126) | 0.0181 | 0.489 | 50 (25.7-71.4) | 48.9 (25.9-70.4) | 0.0229 | 0.618 | 13.8 (0-35.7) | 13.3 (0-35.5) | 0.000189 | 0.00509 |  |
| Noradrenaline | Increase | 0.000346 (-0.141-0.135) | -0.0103 (-0.151-0.122) | <0.0001 | <0.0001 | 50 (26.2-72) | 48.2 (25-70) | <0.0001 | <0.0001 | 13.8 (0-36) | 13 (0-34.5) | <0.0001 | <0.0001 |  |
| Propofol | Decrease | 0.00703 (-0.137-0.147) | 0.0114 (-0.126-0.154) | 0.0055 | 0.149 | 50 (28-73.7) | 51.9 (29.2-73.9) | 0.00995 | 0.269 | 16.1 (0-38.5) | 16.7 (0-40) | 0.0421 | 1 |  |
| Propofol | Increase | 0.0071 (-0.137-0.148) | 0.00777 (-0.13-0.148) | 0.413 | 1 | 50 (26.7-73.9) | 51.6 (28.6-73) | 0.17 | 1 | 14.8 (0-38.5) | 16 (0-38.3) | 0.0695 | 1 |  |
| Vasopressin | Decrease | 0.0389 (0.021-0.0835) | 0.0278 (-0.0129-0.0517) | 0.235 | 1 | 53.8 (51.6-58.3) | 52.6 (51.6-55.1) | 0.341 | 1 | 25.3 (22.4-30.2) | 24.1 (22.4-26.9) | 0.219 | 1 |  |
| Vasopressin | Increase | 0.0295 (-0.00547-0.0798) | 0.0236 (-0.038-0.0517) | 0.261 | 1 | 53.8 (51.6-58.3) | 52.6 (38.7-54.8) | 0.162 | 1 | 25.3 (15.3-29.6) | 24.1 (15.1-29.1) | 0.343 | 1 |  |
| **Name** | **Mean Dose Change** | **% time LPRx_20 > 0.35** | | | | **Mean LPRx_30** | | | | **% time LPRx_30 > 0** | | | |  |
|  |  | **Pre Dose** | **Post Dose** | **P value** | **Adj P Value** | **Pre Dose** | **Post Dose** | **P value** | **Adj P Value** | **Pre Dose** | **Post Dose** | **P value** | **Adj P Value** |  |
| Dobutamine | Decrease | 6.86 (0-27.7) | 6.9 (0-27.6) | 0.671 | 1 | -0.0137 (-0.166-0.169) | -0.0163 (-0.187-0.15) | 0.12 | 1 | 48.1 (17.9-78.2) | 46.3 (16.7-76.9) | 0.297 | 1 |  |
| Dobutamine | Increase | 4 (0-25) | 7.14 (0-28.6) | 0.234 | 1 | 0.00144 (-0.147-0.156) | -0.0168 (-0.172-0.14) | 0.0734 | 1 | 51.5 (19.2-80) | 46.4 (18.5-76.7) | 0.0707 | 1 |  |
| Midazolam | Decrease | 3.7 (0-22.2) | 3.57 (0-21.7) | 0.614 | 1 | -0.00209 (-0.137-0.127) | -0.00166 (-0.14-0.124) | 0.654 | 1 | 50 (20-76) | 48.3 (20.7-75.9) | 0.71 | 1 |  |
| Midazolam | Increase | 3.64 (0-23.1) | 3.57 (0-22.2) | 0.765 | 1 | 0.00326 (-0.131-0.141) | -0.00163 (-0.137-0.128) | 0.143 | 1 | 50 (20-78.9) | 48.7 (20.2-76.7) | 0.224 | 1 |  |
| Morphine | Decrease | 4 (0-24) | 4.17 (0-24.1) | 0.451 | 1 | 0.01 (-0.127-0.147) | 0.00666 (-0.125-0.145) | 0.831 | 1 | 51.9 (24.1-77.8) | 51.1 (23.1-78.1) | 0.637 | 1 |  |
| Morphine | Increase | 3.7 (0-23.1) | 4.17 (0-24.1) | 0.00473 | 0.128 | 0.0111 (-0.135-0.151) | 0.00533 (-0.127-0.146) | 0.685 | 1 | 51.9 (21.4-80.8) | 50 (23.1-77.8) | 0.304 | 1 |  |
| Noradrenaline | Decrease | 3.7 (0-23.3) | 3.57 (0-22.2) | 0.000309 | 0.00834 | -0.00273 (-0.142-0.132) | -0.00753 (-0.142-0.124) | 0.00417 | 0.113 | 50 (20-76.7) | 48.1 (20-75) | <0.0001 | 0.00099 |  |
| Noradrenaline | Increase | 3.7 (0-23.3) | 3.57 (0-22.2) | <0.0001 | 0.000933 | 0.000475 (-0.136-0.137) | -0.00966 (-0.142-0.118) | <0.0001 | <0.0001 | 50 (20.8-77.8) | 47.8 (19.4-74.1) | <0.0001 | <0.0001 |  |
| Propofol | Decrease | 4.35 (0-25.9) | 6.67 (0-26.9) | 0.0642 | 1 | 0.00977 (-0.131-0.157) | 0.0182 (-0.117-0.169) | <0.0001 | 0.000267 | 51.9 (23.3-79.5) | 53.6 (25-81.5) | 0.000113 | 0.00306 |  |
| Propofol | Increase | 4 (0-25.6) | 5 (0-26.1) | 0.077 | 1 | 0.00839 (-0.131-0.153) | 0.0166 (-0.117-0.161) | <0.0001 | 0.000904 | 51.6 (23.1-80) | 53.6 (25-80.8) | 0.00014 | 0.00379 |  |
| Vasopressin | Decrease | 16.3 (13.2-20.9) | 15.2 (9.76-18) | 0.106 | 1 | 0.0326 (-0.0058-0.0915) | 0.0267 (0.021-0.0722) | 0.928 | 1 | 53.1 (40.6-61.2) | 52.2 (51.5-59.1) | 0.827 | 1 |  |
| Vasopressin | Increase | 15.9 (10-20.9) | 15 (8-18) | 0.201 | 1 | 0.0267 (-0.0675-0.0527) | 0.0267 (-0.0555-0.0534) | 0.791 | 1 | 52.4 (39.2-61.2) | 51.6 (35.9-55.5) | 0.498 | 1 |  |
| **Name** | **Mean Dose Change** | **% time LPRx_30 > 0.25** | | | | **% time LPRx_30 > 0.35** | | | | **Mean LPRx_60** | | | |  |
|  |  | **Pre Dose** | **Post Dose** | **P value** | **Adj P Value** | **Pre Dose** | **Post Dose** | **P value** | **Adj P Value** | **Pre Dose** | **Post Dose** | **P value** | **Adj P Value** |  |
| Dobutamine | Decrease | 10.4 (0-40) | 8.51 (0-35.9) | 0.0486 | 1 | 0 (0-24.9) | 0 (0-21.3) | 0.15 | 1 | 0.00383 (-0.174-0.193) | -0.000707 (-0.198-0.177) | 0.458 | 1 |  |
| Dobutamine | Increase | 11.1 (0-40) | 8.89 (0-37.9) | 0.172 | 1 | 0 (0-22.2) | 0 (0-23.3) | 0.799 | 1 | 0.0261 (-0.152-0.212) | -0.00958 (-0.169-0.163) | 0.00289 | 0.078 |  |
| Midazolam | Decrease | 3.92 (0-31.2) | 3.88 (0-31) | 0.73 | 1 | 0 (0-16.4) | 0 (0-15.8) | 0.995 | 1 | 0.00623 (-0.135-0.16) | 0.0104 (-0.122-0.153) | 0.179 | 1 |  |
| Midazolam | Increase | 4 (0-33.3) | 4.35 (0-30.8) | 0.643 | 1 | 0 (0-17.4) | 0 (0-16.1) | 0.455 | 1 | 0.00623 (-0.134-0.164) | 0.00888 (-0.124-0.156) | 0.354 | 1 |  |
| Morphine | Decrease | 7.69 (0-34.8) | 7.69 (0-35.6) | 0.955 | 1 | 0 (0-20) | 0 (0-20.4) | 0.647 | 1 | 0.0182 (-0.134-0.186) | 0.0207 (-0.12-0.181) | 0.366 | 1 |  |
| Morphine | Increase | 7.14 (0-34.6) | 7.41 (0-36) | 0.231 | 1 | 0 (0-18.5) | 0 (0-20.7) | 0.0166 | 0.448 | 0.0188 (-0.136-0.186) | 0.0176 (-0.122-0.181) | 0.662 | 1 |  |
| Noradrenaline | Decrease | 6.25 (0-32.1) | 3.7 (0-31) | <0.0001 | <0.0001 | 0 (0-17.2) | 0 (0-16) | <0.0001 | <0.0001 | 0.00495 (-0.14-0.158) | 0.000871 (-0.137-0.149) | 0.0714 | 1 |  |
| Noradrenaline | Increase | 6.25 (0-33.3) | 3.7 (0-30.8) | <0.0001 | <0.0001 | 0 (0-17.9) | 0 (0-15.6) | <0.0001 | <0.0001 | 0.0075 (-0.133-0.161) | 0.0036 (-0.131-0.153) | 0.0199 | 0.537 |  |
| Propofol | Decrease | 8.33 (0-37.5) | 8.89 (0-40) | 0.18 | 1 | 0 (0-22.2) | 0 (0-23.8) | 0.142 | 1 | 0.0224 (-0.125-0.192) | 0.0304 (-0.107-0.205) | <0.0001 | 0.000341 |  |
| Propofol | Increase | 7.69 (0-36.2) | 8.42 (0-39.3) | 0.0114 | 0.307 | 0 (0-20.8) | 0 (0-23.3) | 0.0181 | 0.488 | 0.0218 (-0.125-0.188) | 0.0293 (-0.108-0.203) | 0.000109 | 0.00295 |  |
| Vasopressin | Decrease | 20.8 (17.3-27.4) | 20.6 (11.5-24) | 0.586 | 1 | 13.3 (11.6-17.1) | 12.1 (0-14.8) | 0.102 | 1 | 0.0102 (-0.0194-0.0749) | 0.0215 (0.00414-0.0398) | 0.54 | 1 |  |
| Vasopressin | Increase | 20.6 (4.44-23.5) | 20.5 (6.76-23.5) | 0.349 | 1 | 12.4 (0-16.6) | 12.1 (0-14.4) | 0.271 | 1 | 0.0101 (-0.0733-0.0301) | 0.0107 (-0.0159-0.0398) | 0.475 | 1 |  |
| **Name** | **Mean Dose Change** | **% time LPRx_60 > 0** | | | | **% time LPRx_60 > 0.25** | | | | **% time LPRx_60 > 0.35** | | | |  |
|  |  | **Pre Dose** | **Post Dose** | **P value** | **Adj P Value** | **Pre Dose** | **Post Dose** | **P value** | **Adj P Value** | **Pre Dose** | **Post Dose** | **P value** | **Adj P Value** |  |
| Dobutamine | Decrease | 51.7 (3.85-96.4) | 48.3 (0-100) | 0.897 | 1 | 0 (0-39.3) | 0 (0-34.6) | 0.0785 | 1 | 0 (0-18.5) | 0 (0-13.7) | 0.246 | 1 |  |
| Dobutamine | Increase | 57.7 (11.5-100) | 45.2 (0-96.4) | 0.0011 | 0.0296 | 0 (0-45.2) | 0 (0-30.8) | 0.00056 | 0.0151 | 0 (0-17.9) | 0 (0-9.52) | 0.00456 | 0.123 |  |
| Midazolam | Decrease | 51.2 (6.9-96) | 53.5 (7.41-96.3) | 0.355 | 1 | 0 (0-29.6) | 0 (0-28) | 0.357 | 1 | 0 (0-8) | 0 (0-7.41) | 0.133 | 1 |  |
| Midazolam | Increase | 51.9 (4.17-96.6) | 52.2 (7.14-96.6) | 0.406 | 1 | 0 (0-30.8) | 0 (0-26.9) | 0.255 | 1 | 0 (0-10.2) | 0 (0-6.45) | 0.0631 | 1 |  |
| Morphine | Decrease | 55.2 (8-100) | 56 (10.7-100) | 0.21 | 1 | 0 (0-38.5) | 0 (0-35.7) | 0.108 | 1 | 0 (0-17.4) | 0 (0-13.8) | 0.0167 | 0.452 |  |
| Morphine | Increase | 55.6 (7.14-100) | 55.6 (7.41-100) | 0.849 | 1 | 0 (0-36) | 0 (0-34.5) | 0.173 | 1 | 0 (0-14.8) | 0 (0-11.5) | 0.051 | 1 |  |
| Noradrenaline | Decrease | 50.2 (7.14-95.5) | 50 (3.33-96.4) | 0.00316 | 0.0854 | 0 (0-30.8) | 0 (0-26.1) | <0.0001 | <0.0001 | 0 (0-9.94) | 0 (0-4) | <0.0001 | <0.0001 |  |
| Noradrenaline | Increase | 51.9 (7.41-96.2) | 50.6 (3.45-96.7) | 0.000317 | 0.00857 | 0 (0-30.8) | 0 (0-26.7) | <0.0001 | <0.0001 | 0 (0-10.2) | 0 (0-3.85) | <0.0001 | <0.0001 |  |
| Propofol | Decrease | 57.1 (10.7-100) | 60.7 (12-100) | <0.0001 | 0.00123 | 0 (0-40) | 0 (0-41.7) | 0.945 | 1 | 0 (0-16.7) | 0 (0-17.2) | 0.977 | 1 |  |
| Propofol | Increase | 56.7 (10.7-100) | 60 (11.5-100) | 0.000244 | 0.00659 | 0 (0-38.5) | 0 (0-40) | 0.362 | 1 | 0 (0-16) | 0 (0-16) | 0.326 | 1 |  |
| Vasopressin | Decrease | 50.2 (39.6-61.9) | 52.4 (49.9-69.7) | 0.563 | 1 | 14.8 (0-16.1) | 14.5 (0-17.9) | 0.808 | 1 | 9.67 (0-9.94) | 9.66 (0-10.8) | 0.552 | 1 |  |
| Vasopressin | Increase | 50.1 (26.7-53.6) | 50.1 (23.3-54.3) | 0.635 | 1 | 14.7 (0-15.3) | 14.5 (0-16.4) | 0.799 | 1 | 9.66 (0-9.94) | 9.66 (0-10.8) | 0.541 | 1 |  |
| **Bolus** | | | | | | | | | | | | | |  |
| **Name** | **Doses** | **Mean MAP** | | | | **Mean CPP** | | | | **% time CPP>60** | | | |  |
|  |  | **Pre Dose** | **Post Dose** | **P value** | **Adj P Value** | **Pre Dose** | **Post Dose** | **P value** | **Adj P Value** | **Pre Dose** | **Post Dose** | **P value** | **Adj P Value** |  |
| Ephedrine | 43 | 71.4 (63.6-81) | 77.8 (67.3-86.7) | 0.137 | 1 | 57.4 (54.1-72.4) | 62.3 (55.7-74.9) | 0.183 | 1 | 70.4 (6.79-100) | 46.2 (4.26-100) | 0.749 | 1 |  |
| Fentanyl | 202 | 80.3 (75.5-88.1) | 80 (72.4-87.9) | 0.204 | 1 | 69.5 (61.5-78.2) | 68 (61.3-76.1) | 0.379 | 1 | 4.35 (0-37) | 11.1 (0-40.4) | 0.202 | 1 |  |
| Midazolam | 894 | 78.7 (74.1-84.2) | 78.2 (73.5-83.6) | 0.0925 | 1 | 64.2 (59.2-70.1) | 63.7 (58.7-68.9) | 0.0935 | 1 | 15.3 (0-63) | 19.8 (0-66.7) | 0.291 | 1 |  |
| Morphine | 4180 | 81.3 (74.9-90.3) | 80 (73.7-88.7) | <0.0001 | <0.0001 | 69.3 (62.7-79.5) | 67.6 (61.3-76.8) | <0.0001 | <0.0001 | 0 (0-28.9) | 3.7 (0-41.9) | <0.0001 | <0.0001 |  |
| Propofol | 4219 | 79.8 (74.2-86) | 78.6 (73.3-85) | <0.0001 | <0.0001 | 66.5 (60.8-73.7) | 65.3 (60.1-72.4) | <0.0001 | <0.0001 | 6.9 (0-44.8) | 10.5 (0-53.3) | <0.0001 | 0.000849 |  |
| Alfentanil | 374 | 78.2 (72.2-85.6) | 77.2 (71.6-83.5) | 0.116 | 1 | 67.2 (61-74.4) | 65.8 (60.9-73.9) | 0.346 | 1 | 11.1 (0-42.1) | 12.5 (0-48.4) | 0.697 | 1 |  |
| **Name** | **% time CPP>70** | | | | **Mean ICP** | | | | **% time ICP>20** | | | |  |  |
|  | **Pre Dose** | **Post Dose** | **P value** | **Adj P Value** | **Pre Dose** | **Post Dose** | **P value** | **Adj P Value** | **Pre Dose** | **Post Dose** | **P value** | **Adj P Value** |  |  |
| Ephedrine | 0 (0-46.4) | 7.41 (0-69.9) | 0.223 | 1 | 11.5 (5.64-16) | 10.7 (6.35-16.1) | 0.704 | 1 | 0 (0-3.01) | 0 (0-4.26) | 0.865 | 1 |  |  |
| Fentanyl | 34.6 (7.41-96.3) | 32 (3.57-82.1) | 0.0744 | 1 | 12.1 (7.23-14.9) | 10.8 (7.18-14.4) | 0.341 | 1 | 0 (0-7.41) | 0 (0-5.4) | 0.732 | 1 |  |  |
| Midazolam | 7.14 (0-49.7) | 3.85 (0-34.3) | 0.0319 | 0.861 | 15.3 (11.6-17.9) | 15.1 (11.6-18) | 0.968 | 1 | 0.325 (0-10.7) | 0 (0-12) | 0.416 | 1 |  |  |
| Morphine | 38.5 (3.45-97.6) | 27.6 (0-91.7) | <0.0001 | <0.0001 | 11.8 (8.34-15.4) | 12.1 (8.47-15.6) | 0.0112 | 0.302 | 0 (0-3.38) | 0 (0-3.6) | 0.0986 | 1 |  |  |
| Propofol | 16.7 (0-75.9) | 10.8 (0-67.4) | <0.0001 | <0.0001 | 13.3 (9.5-16.7) | 13.2 (9.22-16.7) | 0.713 | 1 | 0 (0-7.41) | 0 (0-6.9) | 0.0729 | 1 |  |  |
| Alfentanil | 18.9 (3.45-75.6) | 14.8 (0-74.8) | 0.409 | 1 | 11.3 (7.63-14.3) | 10.7 (7.16-13.7) | 0.2 | 1 | 0 (0-3.7) | 0 (0-0.357) | 0.0229 | 0.617 |  |  |
| **Name** | **% time ICP>22** | | | | **Mean LPRx_10** | | | | **% time LPRx_10 > 0** | | | |  |  |
|  | **Pre Dose** | **Post Dose** | **P value** | **Adj P Value** | **Pre Dose** | **Post Dose** | **P value** | **Adj P Value** | **Pre Dose** | **Post Dose** | **P value** | **Adj P Value** |  |  |
| Ephedrine | 0 (0-0.122) | 0 (0-0.114) | 0.428 | 1 | 0.0132 (-0.0743-0.113) | -0.0197 (-0.239-0.0868) | 0.0432 | 1 | 53.3 (40.3-66) | 42.9 (28.3-55.2) | 0.0445 | 1 |  |  |
| Fentanyl | 0 (0-3.48) | 0 (0-3.57) | 0.617 | 1 | 0.00737 (-0.107-0.135) | -0.00654 (-0.166-0.144) | 0.624 | 1 | 51.9 (32.1-66.3) | 52.4 (30.8-70.7) | 0.605 | 1 |  |  |
| Midazolam | 0 (0-3.57) | 0 (0-3.7) | 0.0906 | 1 | -0.00281 (-0.148-0.122) | 0.00784 (-0.138-0.142) | 0.138 | 1 | 50 (32-65.4) | 50.9 (33.3-68.4) | 0.1 | 1 |  |  |
| Morphine | 0 (0-0) | 0 (0-0) | 0.000947 | 0.0256 | 0.0243 (-0.106-0.173) | 0.0243 (-0.108-0.161) | 0.468 | 1 | 52.4 (36-70.6) | 52.2 (36.6-69.2) | 0.7 | 1 |  |  |
| Propofol | 0 (0-2.38) | 0 (0-0.606) | 0.297 | 1 | 0.0186 (-0.127-0.166) | 0.0175 (-0.134-0.16) | 0.588 | 1 | 51.9 (33.3-69.6) | 51.9 (33.3-69) | 0.763 | 1 |  |  |
| Alfentanil | 0 (0-0.0174) | 0 (0-0) | 0.0604 | 1 | 0.00914 (-0.125-0.154) | 0.00404 (-0.142-0.145) | 0.844 | 1 | 50 (32.1-70.4) | 50.5 (33.6-66.7) | 0.949 | 1 |  |  |
| **Name** | **% time LPRx_10 > 0.25** | | | | **% time LPRx_10 > 0.35** | | | | **Mean LPRx_15** | | | |  |  |
|  | **Pre Dose** | **Post Dose** | **P value** | **Adj P Value** | **Pre Dose** | **Post Dose** | **P value** | **Adj P Value** | **Pre Dose** | **Post Dose** | **P value** | **Adj P Value** |  |  |
| Ephedrine | 32.5 (12.6-42.4) | 21.7 (6.17-33.9) | 0.181 | 1 | 26.2 (4.15-32.7) | 15.4 (3.61-25.7) | 0.133 | 1 | 0.0208 (-0.108-0.121) | -0.048 (-0.242-0.0468) | 0.0295 | 0.795 |  |  |
| Fentanyl | 25.5 (11.2-40) | 24 (9.32-42.3) | 0.89 | 1 | 18.2 (4.19-27.6) | 13.8 (0-29.6) | 0.517 | 1 | 0.0102 (-0.106-0.12) | -0.0238 (-0.153-0.164) | 0.309 | 1 |  |  |
| Midazolam | 24.1 (8-38.1) | 26.1 (11.1-40.7) | 0.033 | 0.892 | 14.7 (3.48-28.6) | 17.4 (4-31.8) | 0.00914 | 0.247 | 5.09e-05 (-0.14-0.119) | 0.00445 (-0.112-0.134) | 0.108 | 1 |  |  |
| Morphine | 26.9 (11.1-43.5) | 27.6 (12.5-42.9) | 0.429 | 1 | 18.2 (4.17-33.3) | 18.8 (5.76-33.3) | 0.383 | 1 | 0.0242 (-0.1-0.166) | 0.023 (-0.107-0.157) | 0.292 | 1 |  |  |
| Propofol | 25.5 (10-42.9) | 25.9 (11.1-42.3) | 0.266 | 1 | 16 (3.7-32.1) | 17.4 (4.17-33.3) | 0.0577 | 1 | 0.0153 (-0.114-0.162) | 0.0199 (-0.122-0.16) | 0.833 | 1 |  |  |
| Alfentanil | 25 (10.7-42.1) | 26.9 (11.1-41.4) | 0.774 | 1 | 15.1 (3.7-31.8) | 19.2 (5.31-31) | 0.169 | 1 | 0.00858 (-0.121-0.148) | 0.012 (-0.115-0.136) | 0.949 | 1 |  |  |
| **Name** | **% time LPRx_15 > 0** | | | | **% time LPRx_15 > 0.25** | | | | **% time LPRx_15 > 0.35** | | | |  |  |
|  | **Pre Dose** | **Post Dose** | **P value** | **Adj P Value** | **Pre Dose** | **Post Dose** | **P value** | **Adj P Value** | **Pre Dose** | **Post Dose** | **P value** | **Adj P Value** |  |  |
| Ephedrine | 53.3 (32.9-64.4) | 45.7 (25-52.1) | 0.0644 | 1 | 30.5 (4.3-38) | 20.8 (5.79-35.8) | 0.533 | 1 | 17.9 (1.79-27.1) | 17.4 (0-23.1) | 0.424 | 1 |  |  |
| Fentanyl | 52.8 (32.1-70.2) | 50 (27.5-71.6) | 0.803 | 1 | 20.8 (3.88-34.6) | 18.5 (3.57-40) | 0.998 | 1 | 11.1 (0-22.9) | 9.76 (0-26.1) | 0.487 | 1 |  |  |
| Midazolam | 50 (28.6-66.7) | 51.7 (33.3-69.5) | 0.0741 | 1 | 18.5 (2.88-34.6) | 22.1 (6.9-37.8) | 0.00427 | 0.115 | 8 (0-24.1) | 11.5 (0-25) | 0.0126 | 0.341 |  |  |
| Morphine | 53.2 (33.3-73) | 53.3 (33.3-72) | 0.71 | 1 | 23.1 (5.26-40.7) | 23.1 (6.67-41.2) | 0.853 | 1 | 12.5 (0-29.6) | 13.3 (0-29.4) | 0.755 | 1 |  |  |
| Propofol | 52 (32-72.2) | 52.4 (33.3-72) | 0.801 | 1 | 20.7 (3.85-40.7) | 22.1 (4.35-41.7) | 0.0623 | 1 | 10.7 (0-29) | 11.5 (0-29.6) | 0.0749 | 1 |  |  |
| Alfentanil | 50.6 (32-71.9) | 51.9 (32.4-70.7) | 0.793 | 1 | 18.3 (3.57-37) | 22.6 (3.57-38.3) | 0.479 | 1 | 8.51 (0-26.9) | 13.1 (0-26.9) | 0.156 | 1 |  |  |
| **Name** | **Mean LPRx_20** | | | | **% time LPRx_20 > 0** | | | | **% time LPRx_20 > 0.25** | | | |  |  |
|  | **Pre Dose** | **Post Dose** | **P value** | **Adj P Value** | **Pre Dose** | **Post Dose** | **P value** | **Adj P Value** | **Pre Dose** | **Post Dose** | **P value** | **Adj P Value** |  |  |
| Ephedrine | 0.0288 (-0.0823-0.145) | -0.0181 (-0.217-0.0419) | 0.0288 | 0.778 | 51.7 (35.6-67.3) | 44.4 (23.6-56.3) | 0.0824 | 1 | 29.8 (7.06-35.7) | 22.2 (1.14-29.6) | 0.207 | 1 |  |  |
| Fentanyl | 0.0199 (-0.112-0.103) | -0.0141 (-0.157-0.153) | 0.456 | 1 | 52 (29.4-69.7) | 50 (30.8-75.3) | 0.933 | 1 | 19.2 (0-32.4) | 12.2 (0-35.9) | 0.609 | 1 |  |  |
| Midazolam | 0.00619 (-0.133-0.132) | 0.0157 (-0.102-0.134) | 0.105 | 1 | 50 (28-70.8) | 51.9 (32-71.9) | 0.129 | 1 | 14.8 (0-34.6) | 18.5 (0-37.5) | 0.0383 | 1 |  |  |
| Morphine | 0.0283 (-0.105-0.169) | 0.0224 (-0.11-0.162) | 0.292 | 1 | 53.8 (32-76.8) | 53.6 (31-75.9) | 0.31 | 1 | 20 (0-40.7) | 20 (0-40.7) | 0.957 | 1 |  |  |
| Propofol | 0.022 (-0.114-0.163) | 0.0248 (-0.108-0.166) | 0.248 | 1 | 52.7 (29.6-76) | 53.8 (31.3-76) | 0.255 | 1 | 17.2 (0-40.2) | 18.5 (0-40.7) | 0.0537 | 1 |  |  |
| Alfentanil | 0.00643 (-0.113-0.155) | 0.019 (-0.111-0.145) | 0.821 | 1 | 51.9 (29.7-75) | 53.2 (32.1-74.8) | 0.648 | 1 | 13.8 (0-38.5) | 19.2 (0-36) | 0.244 | 1 |  |  |
| **Name** | **% time LPRx_20 > 0.35** | | | | **Mean LPRx_30** | | | | **% time LPRx_30 > 0** | | | |  |  |
|  | **Pre Dose** | **Post Dose** | **P value** | **Adj P Value** | **Pre Dose** | **Post Dose** | **P value** | **Adj P Value** | **Pre Dose** | **Post Dose** | **P value** | **Adj P Value** |  |  |
| Ephedrine | 12.2 (0-26.7) | 13 (0-21.6) | 0.49 | 1 | 0.0452 (-0.0873-0.136) | 0.00398 (-0.139-0.067) | 0.355 | 1 | 46.7 (23.8-70) | 52.5 (30.2-63.7) | 0.73 | 1 |  |  |
| Fentanyl | 7.15 (0-20) | 4.17 (0-25) | 0.892 | 1 | -0.00438 (-0.106-0.13) | -3e-04 (-0.119-0.152) | 0.482 | 1 | 49.7 (22.5-73) | 50 (26.9-74.7) | 0.166 | 1 |  |  |
| Midazolam | 4 (0-22.2) | 7.41 (0-24) | 0.0569 | 1 | 0.00961 (-0.138-0.146) | 0.0199 (-0.101-0.146) | 0.112 | 1 | 53.5 (23.4-79.3) | 53.5 (25.8-79.2) | 0.596 | 1 |  |  |
| Morphine | 7.69 (0-27.5) | 8 (0-27.3) | 0.832 | 1 | 0.0279 (-0.112-0.178) | 0.0297 (-0.11-0.17) | 0.83 | 1 | 55.6 (26.9-83.3) | 55.6 (26.9-82.5) | 0.993 | 1 |  |  |
| Propofol | 6.67 (0-26.1) | 7.41 (0-28) | 0.0755 | 1 | 0.0251 (-0.114-0.168) | 0.0384 (-0.0944-0.18) | 0.00319 | 0.0862 | 55.2 (26.2-82.2) | 57.1 (28.6-84.6) | 0.00672 | 0.181 |  |  |
| Alfentanil | 3.92 (0-25.9) | 7.69 (0-24.1) | 0.262 | 1 | 0.0242 (-0.109-0.161) | 0.0256 (-0.102-0.166) | 0.935 | 1 | 51.9 (27.1-80) | 55.6 (26-82.1) | 0.539 | 1 |  |  |
| **Name** | **% time LPRx_30 > 0.25** | | | | **% time LPRx_30 > 0.35** | | | | **Mean LPRx_60** | | | |  |  |
|  | **Pre Dose** | **Post Dose** | **P value** | **Adj P Value** | **Pre Dose** | **Post Dose** | **P value** | **Adj P Value** | **Pre Dose** | **Post Dose** | **P value** | **Adj P Value** |  |  |
| Ephedrine | 20 (0-32.7) | 15.4 (0-29.6) | 0.763 | 1 | 4.17 (0-22.9) | 3.57 (0-22.7) | 0.787 | 1 | 0.0684 (-0.141-0.24) | 0.0412 (-0.0608-0.199) | 0.863 | 1 |  |  |
| Fentanyl | 7.69 (0-36) | 14 (0-39.1) | 0.173 | 1 | 0 (0-18.6) | 0 (0-25) | 0.482 | 1 | 0.0308 (-0.125-0.241) | 0.015 (-0.146-0.203) | 0.381 | 1 |  |  |
| Midazolam | 9.45 (0-35.7) | 12.7 (0-38.3) | 0.458 | 1 | 0 (0-20) | 0 (0-21.5) | 0.204 | 1 | 0.0342 (-0.145-0.204) | 0.0388 (-0.0943-0.206) | 0.275 | 1 |  |  |
| Morphine | 13.8 (0-40.9) | 13.3 (0-40) | 0.501 | 1 | 2.33 (0-25) | 0 (0-24) | 0.59 | 1 | 0.0306 (-0.127-0.207) | 0.0366 (-0.127-0.209) | 0.644 | 1 |  |  |
| Propofol | 11.1 (0-39.6) | 12 (0-41.9) | 0.35 | 1 | 0 (0-23.3) | 0 (0-24.7) | 0.463 | 1 | 0.0393 (-0.117-0.21) | 0.0483 (-0.0946-0.226) | 0.0045 | 0.121 |  |  |
| Alfentanil | 11.7 (0-39.8) | 11.3 (0-33.3) | 0.411 | 1 | 0 (0-21.3) | 0 (0-17.2) | 0.419 | 1 | 0.0366 (-0.108-0.171) | 0.0354 (-0.0974-0.18) | 0.791 | 1 |  |  |
| **Name** | **% time LPRx_60 > 0** | | | | **% time LPRx_60 > 0.25** | | | | **% time LPRx_60 > 0.35** | | | |  |  |
|  | **Pre Dose** | **Post Dose** | **P value** | **Adj P Value** | **Pre Dose** | **Post Dose** | **P value** | **Adj P Value** | **Pre Dose** | **Post Dose** | **P value** | **Adj P Value** |  |  |
| Ephedrine | 55 (21-88.8) | 52.6 (24-95) | 0.886 | 1 | 22.7 (0-50) | 3.33 (0-49) | 0.537 | 1 | 11.5 (0-31.6) | 0 (0-34.8) | 0.554 | 1 |  |  |
| Fentanyl | 62.8 (5.6-100) | 57.1 (0-100) | 0.37 | 1 | 0 (0-47.7) | 0 (0-45.8) | 0.119 | 1 | 0 (0-21.7) | 0 (0-19) | 0.249 | 1 |  |  |
| Midazolam | 59.8 (9.64-100) | 60 (15-100) | 0.309 | 1 | 0 (0-42.3) | 0 (0-42.7) | 0.84 | 1 | 0 (0-23.1) | 0 (0-17.9) | 0.193 | 1 |  |  |
| Morphine | 57.9 (13-100) | 59.1 (11.1-100) | 0.171 | 1 | 0 (0-42.9) | 0 (0-42.3) | 0.112 | 1 | 0 (0-23.1) | 0 (0-19.2) | 0.0139 | 0.375 |  |  |
| Propofol | 60.5 (14.8-100) | 67.1 (17.2-100) | 0.000416 | 0.0112 | 0 (0-43.5) | 0 (0-46.5) | 0.799 | 1 | 0 (0-20) | 0 (0-18.5) | 0.15 | 1 |  |  |
| Alfentanil | 58.9 (17.9-96.6) | 60.4 (14.4-100) | 0.592 | 1 | 0 (0-37.4) | 0 (0-32.7) | 0.263 | 1 | 0 (0-22.2) | 0 (0-11.3) | 0.0631 | 1 |  |  |

*The table demonstrates the median and interquartile range of the pre/pose dose windows as well as the Wilcox signed ranked test between these windows with p values adjusted using the Bonferroni analysis. The “change” indicates if the continuous infusion was increase/decrease or was a bolus dose. CPP, cerebral prefusion pressure; ICP, intracranial pressure; LPRx_10, pressure reactivity over 10 minutes; LPRx_15, pressure reactivity over 15 minutes; LPRx_20, pressure reactivity over 20 minutes; LPRx_30, pressure reactivity over 30 minutes; LPRx_60, pressure reactivity over 60 minutes;*

# Appendix I. Pre-time window over 50% time L-PRx_10 > 0

The table contains all infusions given with the LPRx_10 pre-time window 50% > 0, separated into the continuous infusion doses then the bolus doses.

| **Continuous Intravenous** | | | | | | | | | | | | | | |
| --- | --- | --- | --- | --- | --- | --- | --- | --- | --- | --- | --- | --- | --- | --- |
| **Name** | **Doses** | **Mean Dose Change** | **Mean MAP** | | | | **Mean CPP** | | | | **% time CPP>60** | | | |
|  |  |  | **Pre Dose** | **Post Dose** | **P value** | **Adj P Value** | **Pre Dose** | **Post Dose** | **P value** | **Adj P Value** | **Pre Dose** | **Post Dose** | **P value** | **Adj P Value** |
| Dobutamine | 744 | Decrease | 76.7 (70.6-82.8) | 75.5 (70.6-81.6) | 0.125 | 1 | 63.8 (58.4-70) | 62.9 (58.2-68.2) | 0.102 | 1 | 14.3 (0-76.3) | 21.4 (0-78.6) | 0.142 | 1 |
| Dobutamine | 587 | Increase | 73.1 (67.9-78.9) | 72.3 (67.9-78.2) | 0.324 | 1 | 59.5 (54.3-65.9) | 59.6 (54.6-64.7) | 0.778 | 1 | 64 (13.8-100) | 66.7 (11.1-100) | 0.957 | 1 |
| Midazolam | 2669 | Decrease | 78.3 (73.1-84.5) | 78.1 (73.3-84.3) | 0.649 | 1 | 64.6 (59.5-71) | 64.7 (59.7-71) | 0.571 | 1 | 10 (0-61.5) | 10.7 (0-57.1) | 0.83 | 1 |
| Midazolam | 2583 | Increase | 78.5 (73.1-85.1) | 78 (72.9-84.4) | 0.0376 | 1 | 64.2 (58.9-70.8) | 64.2 (59.1-70.3) | 0.754 | 1 | 13 (0-69.2) | 12.1 (0-64.3) | 0.434 | 1 |
| Morphine | 4111 | Decrease | 77.7 (72.6-84.2) | 77.3 (72.2-83.7) | 0.0697 | 1 | 65.2 (59.8-71.6) | 65.1 (59.5-71.3) | 0.179 | 1 | 7.41 (0-57.7) | 10.3 (0-60) | 0.0709 | 1 |
| Morphine | 3967 | Increase | 78.1 (72.7-84.9) | 77.3 (71.9-83.9) | <0.0001 | 0.000169 | 65.2 (59.5-71.9) | 64.7 (59-70.9) | 0.00655 | 0.177 | 8.89 (0-60.7) | 10.7 (0-66) | 0.208 | 1 |
| Noradrenaline | 26222 | Decrease | 79 (73.5-85.2) | 78.3 (73-84.3) | <0.0001 | <0.0001 | 65.9 (60.1-72.5) | 65.3 (59.9-71.5) | <0.0001 | <0.0001 | 7.14 (0-51.9) | 8.7 (0-53.6) | <0.0001 | 0.00218 |
| Noradrenaline | 24659 | Increase | 76.4 (71-82.6) | 76.2 (70.9-82.5) | 0.442 | 1 | 62.8 (57.5-69.1) | 63.2 (58.2-69.3) | <0.0001 | <0.0001 | 26.9 (0-81.5) | 21.4 (0-76.9) | <0.0001 | <0.0001 |
| Propofol | 8135 | Decrease | 77.6 (72.1-84.1) | 77.3 (71.9-83.7) | 0.0275 | 0.743 | 65.1 (59.5-71.8) | 64.8 (59.4-71.4) | 0.083 | 1 | 9.09 (0-61.5) | 11.1 (0-61.5) | 0.103 | 1 |
| Propofol | 7359 | Increase | 78.7 (73.1-85.4) | 77.6 (72.1-84) | <0.0001 | <0.0001 | 65.7 (60-72.6) | 65 (59.5-71.5) | <0.0001 | <0.0001 | 7.69 (0-55.3) | 10.7 (0-59.3) | 0.00226 | 0.061 |
| Vasopressin | 60 | Decrease | 82.2 (75.6-84.1) | 80.2 (74.2-82.7) | 0.0566 | 1 | 57.5 (55.5-59.1) | 58.7 (56.4-59.6) | 0.77 | 1 | 66 (58.9-76.3) | 60.8 (55.3-84.5) | 0.831 | 1 |
| Vasopressin | 45 | Increase | 81.3 (71.1-84.1) | 80.1 (70.7-82.7) | 0.283 | 1 | 56.4 (51.5-58.7) | 57.5 (54.5-59.1) | 0.185 | 1 | 70.2 (60.8-100) | 68 (60.8-100) | 0.339 | 1 |
| **Name** | **Mean Dose Change** | **% time CPP>70** | | | | **Mean ICP** | | | | **% time ICP>20** | | | |  |
|  |  | **Pre Dose** | **Post Dose** | **P value** | **Adj P Value** | **Pre Dose** | **Post Dose** | **P value** | **Adj P Value** | **Pre Dose** | **Post Dose** | **P value** | **Adj P Value** |  |
| Dobutamine | Decrease | 3.85 (0-40.1) | 2.13 (0-23.3) | 0.0092 | 0.248 | 13.5 (9.62-16.4) | 13.5 (10-16.2) | 0.674 | 1 | 0 (0-3.85) | 0 (0-0) | 0.00244 | 0.0659 |  |
| Dobutamine | Increase | 0 (0-15.4) | 0 (0-7.85) | 0.114 | 1 | 14.1 (10.5-17.2) | 14 (10.4-17) | 0.46 | 1 | 0 (0-8.89) | 0 (0-3.7) | 0.00358 | 0.0968 |  |
| Midazolam | Decrease | 5.88 (0-55.2) | 6.67 (0-54.8) | 0.869 | 1 | 13.6 (10.2-16.7) | 13.5 (10-16.5) | 0.155 | 1 | 0 (0-4.35) | 0 (0-3.7) | 0.241 | 1 |  |
| Midazolam | Increase | 4.17 (0-52.3) | 4 (0-48.1) | 0.309 | 1 | 14.4 (10.8-17.5) | 14 (10.5-17.1) | 0.00795 | 0.215 | 0 (0-10) | 0 (0-5.02) | <0.0001 | 0.000353 |  |
| Morphine | Decrease | 7.69 (0-61.5) | 7.14 (0-56.6) | 0.18 | 1 | 12.5 (8.87-15.8) | 12.4 (8.89-15.8) | 0.528 | 1 | 0 (0-3.33) | 0 (0-2.48) | 0.638 | 1 |  |
| Morphine | Increase | 7.69 (0-63.1) | 5.88 (0-54.5) | 0.000804 | 0.0217 | 13 (9.25-16.6) | 12.7 (8.91-16.2) | 0.00949 | 0.256 | 0 (0-4.55) | 0 (0-3.45) | <0.0001 | 0.000941 |  |
| Noradrenaline | Decrease | 10.7 (0-69.2) | 7.5 (0-60) | <0.0001 | <0.0001 | 13 (9.43-16.2) | 12.9 (9.43-16) | 0.023 | 0.62 | 0 (0-4) | 0 (0-3.45) | <0.0001 | <0.0001 |  |
| Noradrenaline | Increase | 3.85 (0-34.6) | 3.7 (0-36) | 0.0688 | 1 | 13.5 (9.9-16.8) | 13 (9.34-16.4) | <0.0001 | <0.0001 | 0 (0-7.14) | 0 (0-3.57) | <0.0001 | <0.0001 |  |
| Propofol | Decrease | 7.14 (0-61.5) | 6.9 (0-57.7) | 0.137 | 1 | 12.5 (8.55-16) | 12.4 (8.42-16) | 0.331 | 1 | 0 (0-3.57) | 0 (0-3.45) | 0.11 | 1 |  |
| Propofol | Increase | 10.3 (0-69.2) | 7.41 (0-58.6) | <0.0001 | <0.0001 | 13 (8.98-16.6) | 12.7 (8.61-16.2) | 0.000457 | 0.0123 | 0 (0-6.9) | 0 (0-3.57) | <0.0001 | <0.0001 |  |
| Vasopressin | Decrease | 6.74 (3.23-6.81) | 5.67 (0-6.74) | 0.012 | 0.324 | 15.2 (13.7-16.6) | 13.9 (12.7-16.9) | 0.146 | 1 | 8.04 (0-11.5) | 6.64 (0-8.87) | 0.276 | 1 |  |
| Vasopressin | Increase | 5.82 (0-6.81) | 5.82 (0-6.74) | 0.536 | 1 | 15.2 (13.9-17.7) | 15.2 (12.7-17) | 0.283 | 1 | 8.87 (0-11.5) | 7.22 (0-8.87) | 0.553 | 1 |  |
| **Name** | **Mean Dose Change** | **% time ICP>22** | | | | **Mean LPRx_10** | | | | **% time LPRx_10 > 0** | | | |  |
|  |  | **Pre Dose** | **Post Dose** | **P value** | **Adj P Value** | **Pre Dose** | **Post Dose** | **P value** | **Adj P Value** | **Pre Dose** | **Post Dose** | **P value** | **Adj P Value** |  |
| Dobutamine | Decrease | 0 (0-0) | 0 (0-0) | 0.00512 | 0.138 | 0.167 (0.0788-0.272) | 0.0387 (-0.105-0.185) | <0.0001 | <0.0001 | 68.9 (60-80.8) | 53.8 (37-70.5) | <0.0001 | <0.0001 |  |
| Dobutamine | Increase | 0 (0-2.17) | 0 (0-0) | 0.00286 | 0.0773 | 0.159 (0.0642-0.258) | 0.0387 (-0.0987-0.19) | <0.0001 | <0.0001 | 69.2 (60-81.5) | 55.6 (36.7-74.1) | <0.0001 | <0.0001 |  |
| Midazolam | Decrease | 0 (0-0) | 0 (0-0) | 0.128 | 1 | 0.156 (0.0666-0.274) | 0.0205 (-0.14-0.16) | <0.0001 | <0.0001 | 67.9 (58.6-80.8) | 51.9 (33.3-68) | <0.0001 | <0.0001 |  |
| Midazolam | Increase | 0 (0-2.38) | 0 (0-0) | <0.0001 | 0.000369 | 0.162 (0.0721-0.281) | 0.0214 (-0.135-0.163) | <0.0001 | <0.0001 | 69 (59.3-80.8) | 51.9 (34.5-69) | <0.0001 | <0.0001 |  |
| Morphine | Decrease | 0 (0-0) | 0 (0-0) | 0.538 | 1 | 0.155 (0.0662-0.265) | 0.0213 (-0.123-0.166) | <0.0001 | <0.0001 | 67.9 (58.6-80) | 51.9 (35.3-69.2) | <0.0001 | <0.0001 |  |
| Morphine | Increase | 0 (0-0) | 0 (0-0) | <0.0001 | 0.000191 | 0.16 (0.0666-0.277) | 0.018 (-0.131-0.168) | <0.0001 | <0.0001 | 69.2 (59.3-82.1) | 51.9 (34.6-69) | <0.0001 | <0.0001 |  |
| Noradrenaline | Decrease | 0 (0-0) | 0 (0-0) | <0.0001 | <0.0001 | 0.157 (0.0651-0.273) | 0.0169 (-0.132-0.168) | <0.0001 | <0.0001 | 68.2 (58.8-80) | 51.7 (33.3-69.2) | <0.0001 | <0.0001 |  |
| Noradrenaline | Increase | 0 (0-0.131) | 0 (0-0) | <0.0001 | <0.0001 | 0.159 (0.0649-0.275) | 0.0159 (-0.135-0.166) | <0.0001 | <0.0001 | 68.3 (58.8-80.8) | 51.7 (33.3-69) | <0.0001 | <0.0001 |  |
| Propofol | Decrease | 0 (0-0) | 0 (0-0) | 0.114 | 1 | 0.159 (0.0689-0.273) | 0.0229 (-0.125-0.173) | <0.0001 | <0.0001 | 69 (59.1-80.8) | 52.2 (34.8-70) | <0.0001 | <0.0001 |  |
| Propofol | Increase | 0 (0-0.204) | 0 (0-0) | <0.0001 | <0.0001 | 0.159 (0.068-0.279) | 0.0191 (-0.131-0.165) | <0.0001 | <0.0001 | 69 (59.3-81.5) | 51.9 (34.5-69) | <0.0001 | <0.0001 |  |
| Vasopressin | Decrease | 1.3 (0-1.7) | 1.07 (0-1.3) | 0.301 | 1 | 0.0354 (0.0188-0.207) | 0.0188 (0.00906-0.0316) | <0.0001 | <0.0001 | 54.4 (53.3-64.7) | 53.3 (51.2-54.2) | <0.0001 | 0.000183 |  |
| Vasopressin | Increase | 1.3 (0-1.7) | 1.07 (0-1.7) | 0.47 | 1 | 0.0326 (0.0188-0.0638) | 0.0188 (0.00906-0.0326) | 0.000548 | 0.0148 | 54.4 (53.3-63) | 53.3 (51.2-54.2) | <0.0001 | 0.00135 |  |
| **Name** | **Mean Dose Change** | **% time LPRx_10 > 0.25** | | | | **% time LPRx_10 > 0.35** | | | | **Mean LPRx_15** | | | |  |
|  |  | **Pre Dose** | **Post Dose** | **P value** | **Adj P Value** | **Pre Dose** | **Post Dose** | **P value** | **Adj P Value** | **Pre Dose** | **Post Dose** | **P value** | **Adj P Value** |  |
| Dobutamine | Decrease | 44.4 (32-57.1) | 29 (11.5-45.5) | <0.0001 | <0.0001 | 33.3 (20.8-46.4) | 20.7 (4.85-36.7) | <0.0001 | <0.0001 | 0.154 (0.0612-0.262) | 0.0187 (-0.114-0.175) | <0.0001 | <0.0001 |  |
| Dobutamine | Increase | 40.9 (29.6-55.8) | 29 (14.3-44.4) | <0.0001 | <0.0001 | 31 (19.2-44.4) | 18.8 (5.33-36) | <0.0001 | <0.0001 | 0.142 (0.0597-0.249) | 0.0373 (-0.115-0.17) | <0.0001 | <0.0001 |  |
| Midazolam | Decrease | 41.9 (29.6-56.2) | 26.5 (10.3-42.9) | <0.0001 | <0.0001 | 32 (18.5-46.4) | 17.9 (3.7-33.3) | <0.0001 | <0.0001 | 0.133 (0.0463-0.257) | 0.0129 (-0.133-0.152) | <0.0001 | <0.0001 |  |
| Midazolam | Increase | 42.9 (29.6-57.7) | 26.7 (10.7-43.3) | <0.0001 | <0.0001 | 32 (18.5-48.1) | 17.4 (3.85-34.5) | <0.0001 | <0.0001 | 0.138 (0.0504-0.26) | 0.0192 (-0.132-0.146) | <0.0001 | <0.0001 |  |
| Morphine | Decrease | 41.4 (29-55.7) | 26.9 (11.1-43.5) | <0.0001 | <0.0001 | 30.8 (17.9-45.8) | 18.3 (3.85-33.3) | <0.0001 | <0.0001 | 0.137 (0.0476-0.252) | 0.0219 (-0.118-0.168) | <0.0001 | <0.0001 |  |
| Morphine | Increase | 41.9 (28.6-57.1) | 26.7 (10.7-43.3) | <0.0001 | <0.0001 | 30.8 (17.2-47.1) | 17.9 (3.85-33.3) | <0.0001 | <0.0001 | 0.143 (0.0502-0.267) | 0.0185 (-0.125-0.157) | <0.0001 | <0.0001 |  |
| Noradrenaline | Decrease | 42.3 (29-56.5) | 26.7 (10.7-43.3) | <0.0001 | <0.0001 | 31 (17.9-46.4) | 17.9 (3.85-33.3) | <0.0001 | <0.0001 | 0.139 (0.0447-0.256) | 0.0169 (-0.13-0.153) | <0.0001 | <0.0001 |  |
| Noradrenaline | Increase | 42.3 (28.6-56.7) | 26.1 (10.7-43.3) | <0.0001 | <0.0001 | 31 (18.4-46.7) | 17.9 (3.7-33.3) | <0.0001 | <0.0001 | 0.14 (0.0469-0.259) | 0.0109 (-0.136-0.147) | <0.0001 | <0.0001 |  |
| Propofol | Decrease | 42.9 (29.6-56.7) | 26.7 (11.1-44.4) | <0.0001 | <0.0001 | 32 (18.5-46.7) | 18.5 (4.17-34.8) | <0.0001 | <0.0001 | 0.146 (0.0526-0.266) | 0.023 (-0.115-0.169) | <0.0001 | <0.0001 |  |
| Propofol | Increase | 42.3 (29-57.1) | 26.7 (10.7-43.6) | <0.0001 | <0.0001 | 31 (17.9-47.9) | 17.9 (3.85-34.5) | <0.0001 | <0.0001 | 0.147 (0.0544-0.269) | 0.0174 (-0.122-0.16) | <0.0001 | <0.0001 |  |
| Vasopressin | Decrease | 30.5 (29.6-46.8) | 29.6 (27.8-29.9) | <0.0001 | <0.0001 | 23.4 (22.3-35) | 22.3 (20.5-23) | <0.0001 | <0.0001 | 0.0375 (0.0248-0.121) | 0.0248 (0.017-0.0429) | 0.000424 | 0.0114 |  |
| Vasopressin | Increase | 30.5 (29.6-32.6) | 29.6 (27.8-30.6) | 0.00854 | 0.231 | 23.4 (22.3-28) | 22.3 (20.5-24) | 0.00335 | 0.0904 | 0.0375 (0.0248-0.07) | 0.0171 (0.0109-0.0429) | 0.00418 | 0.113 |  |
| **Name** | **Mean Dose Change** | **% time LPRx_15 > 0** | | | | **% time LPRx_15 > 0.25** | | | | **% time LPRx_15 > 0.35** | | | |  |
|  |  | **Pre Dose** | **Post Dose** | **P value** | **Adj P Value** | **Pre Dose** | **Post Dose** | **P value** | **Adj P Value** | **Pre Dose** | **Post Dose** | **P value** | **Adj P Value** |  |
| Dobutamine | Decrease | 70.4 (57.6-84.8) | 53.3 (32.6-73.4) | <0.0001 | <0.0001 | 40 (24-54.2) | 24 (4.55-43.5) | <0.0001 | <0.0001 | 25.9 (12.9-43.4) | 13.6 (0-33.3) | <0.0001 | <0.0001 |  |
| Dobutamine | Increase | 70.8 (58.3-84.6) | 55.6 (30-74.5) | <0.0001 | <0.0001 | 36.6 (22.2-54.9) | 24.1 (4.88-41.9) | <0.0001 | <0.0001 | 25.8 (9.09-41.7) | 15.6 (0-29.6) | <0.0001 | <0.0001 |  |
| Midazolam | Decrease | 69 (56.4-83.3) | 51.9 (31-71.4) | <0.0001 | <0.0001 | 36.7 (21.4-54.2) | 20.7 (3.7-39.3) | <0.0001 | <0.0001 | 23.8 (9.09-41.7) | 11.1 (0-27.6) | <0.0001 | <0.0001 |  |
| Midazolam | Increase | 69.2 (56.7-85) | 52.4 (32-70.6) | <0.0001 | <0.0001 | 36.7 (21.4-55.6) | 21.4 (3.7-40) | <0.0001 | <0.0001 | 23.3 (8-44) | 10.7 (0-28.6) | <0.0001 | <0.0001 |  |
| Morphine | Decrease | 69.2 (56.4-83.9) | 52.4 (33.3-72) | <0.0001 | <0.0001 | 36.7 (22.2-53.8) | 22.2 (4.04-41.4) | <0.0001 | <0.0001 | 24.1 (10.3-42.3) | 12 (0-29.3) | <0.0001 | <0.0001 |  |
| Morphine | Increase | 70.8 (56.8-86.2) | 52.4 (32.1-71.4) | <0.0001 | <0.0001 | 36.7 (21.7-56.7) | 21.4 (4-40.7) | <0.0001 | <0.0001 | 24 (8.63-43.8) | 11.5 (0-28.6) | <0.0001 | <0.0001 |  |
| Noradrenaline | Decrease | 69.2 (56-83.3) | 51.9 (31-70.4) | <0.0001 | <0.0001 | 37 (21.4-55.2) | 21.1 (3.7-40) | <0.0001 | <0.0001 | 24.1 (9.3-42.9) | 10.8 (0-28.6) | <0.0001 | <0.0001 |  |
| Noradrenaline | Increase | 69.6 (56.5-84) | 51.6 (30.4-70.4) | <0.0001 | <0.0001 | 37 (21.4-55.2) | 20 (3.57-39.3) | <0.0001 | <0.0001 | 24.1 (9.09-43.3) | 10.7 (0-28.6) | <0.0001 | <0.0001 |  |
| Propofol | Decrease | 70.4 (57.1-84.8) | 53.6 (33.3-72.4) | <0.0001 | <0.0001 | 38.5 (23.1-56.6) | 22.6 (4.55-42.9) | <0.0001 | <0.0001 | 25.9 (10.3-44.4) | 12.1 (0-31) | <0.0001 | <0.0001 |  |
| Propofol | Increase | 70.4 (57.7-85.2) | 52.4 (32.1-71.4) | <0.0001 | <0.0001 | 37.9 (22.2-56.7) | 21.4 (4.35-41.4) | <0.0001 | <0.0001 | 25 (10-44.8) | 11.4 (0-30) | <0.0001 | <0.0001 |  |
| Vasopressin | Decrease | 54.8 (53.1-60.3) | 53.4 (52.1-54.8) | 0.00487 | 0.131 | 27.6 (26.7-34.8) | 26.7 (24.9-28.2) | <0.0001 | 0.00168 | 18.9 (17.5-24.5) | 17.8 (16.3-19.7) | 0.000102 | 0.00275 |  |
| Vasopressin | Increase | 54.8 (53.4-60) | 53.4 (52.1-54.8) | 0.00468 | 0.126 | 27.6 (25.1-30.4) | 26.7 (24.9-28.2) | 0.0269 | 0.727 | 18.9 (16.5-22.2) | 17.8 (16.3-19.7) | 0.0271 | 0.732 |  |
| **Name** | **Mean Dose Change** | **Mean LPRx_20** | | | | **% time LPRx_20 > 0** | | | | **% time LPRx_20 > 0.25** | | | |  |
|  |  | **Pre Dose** | **Post Dose** | **P value** | **Adj P Value** | **Pre Dose** | **Post Dose** | **P value** | **Adj P Value** | **Pre Dose** | **Post Dose** | **P value** | **Adj P Value** |  |
| Dobutamine | Decrease | 0.136 (0.0334-0.264) | 0.0196 (-0.13-0.171) | <0.0001 | <0.0001 | 70 (54.9-88) | 51.9 (27.6-73.3) | <0.0001 | <0.0001 | 36 (16-55.7) | 19.2 (0-41.7) | <0.0001 | <0.0001 |  |
| Dobutamine | Increase | 0.13 (0.0327-0.251) | 0.0197 (-0.124-0.163) | <0.0001 | <0.0001 | 71.4 (53.6-89.3) | 53.6 (27.6-76) | <0.0001 | <0.0001 | 33.3 (14.3-54.1) | 20.8 (0-39.8) | <0.0001 | <0.0001 |  |
| Midazolam | Decrease | 0.11 (0.022-0.241) | 0.0101 (-0.127-0.149) | <0.0001 | <0.0001 | 68 (51.9-86.7) | 51.7 (28.6-74.1) | <0.0001 | <0.0001 | 29.6 (12-52) | 15.4 (0-37) | <0.0001 | <0.0001 |  |
| Midazolam | Increase | 0.117 (0.0237-0.254) | 0.0182 (-0.125-0.143) | <0.0001 | <0.0001 | 70.4 (52-88.5) | 53.1 (29.6-73.1) | <0.0001 | <0.0001 | 30.4 (11.5-53.6) | 16.7 (0-37.8) | <0.0001 | <0.0001 |  |
| Morphine | Decrease | 0.119 (0.0269-0.249) | 0.0218 (-0.113-0.16) | <0.0001 | <0.0001 | 69 (53.6-87.5) | 53.6 (31.9-75) | <0.0001 | <0.0001 | 31.2 (14.3-53.6) | 18.5 (0-40) | <0.0001 | <0.0001 |  |
| Morphine | Increase | 0.129 (0.0305-0.258) | 0.0181 (-0.118-0.153) | <0.0001 | <0.0001 | 71.4 (54-90.9) | 53.3 (31-74.1) | <0.0001 | <0.0001 | 31 (13.7-55.6) | 17.9 (0-40) | <0.0001 | <0.0001 |  |
| Noradrenaline | Decrease | 0.118 (0.022-0.248) | 0.0134 (-0.125-0.143) | <0.0001 | <0.0001 | 69 (52-87) | 51.9 (28.6-72.4) | <0.0001 | <0.0001 | 30.8 (13.3-53.6) | 16.3 (0-37.9) | <0.0001 | <0.0001 |  |
| Noradrenaline | Increase | 0.118 (0.0236-0.25) | 0.00585 (-0.134-0.138) | <0.0001 | <0.0001 | 69.2 (52.2-87.1) | 50.9 (27.6-72) | <0.0001 | <0.0001 | 31 (12.5-53.6) | 14.8 (0-36.7) | <0.0001 | <0.0001 |  |
| Propofol | Decrease | 0.131 (0.0301-0.265) | 0.0247 (-0.109-0.167) | <0.0001 | <0.0001 | 70.8 (53.6-88.9) | 53.6 (32-75.9) | <0.0001 | <0.0001 | 33.3 (15.4-56.2) | 19.2 (0-42.3) | <0.0001 | <0.0001 |  |
| Propofol | Increase | 0.13 (0.0318-0.267) | 0.0195 (-0.112-0.164) | <0.0001 | <0.0001 | 70.8 (53.8-89.3) | 53.3 (31-75) | <0.0001 | <0.0001 | 33.3 (14.3-56) | 18.4 (0-40.7) | <0.0001 | <0.0001 |  |
| Vasopressin | Decrease | 0.0389 (0.026-0.0873) | 0.0278 (0.021-0.0517) | 0.00524 | 0.141 | 53.8 (52.4-58.4) | 52.6 (51.6-54.8) | 0.0193 | 0.522 | 25.3 (22.5-30.4) | 24.1 (22.4-26.9) | 0.00235 | 0.0633 |  |
| Vasopressin | Increase | 0.0389 (0.021-0.0686) | 0.0236 (0.017-0.0517) | 0.0563 | 1 | 53.8 (52.6-58.3) | 52.6 (51.6-54.8) | 0.088 | 1 | 25.3 (22.4-30.2) | 24.1 (22.4-26.9) | 0.0841 | 1 |  |
| **Name** | **Mean Dose Change** | **% time LPRx_20 > 0.35** | | | | **Mean LPRx_30** | | | | **% time LPRx_30 > 0** | | | |  |
|  |  | **Pre Dose** | **Post Dose** | **P value** | **Adj P Value** | **Pre Dose** | **Post Dose** | **P value** | **Adj P Value** | **Pre Dose** | **Post Dose** | **P value** | **Adj P Value** |  |
| Dobutamine | Decrease | 23.1 (4.44-43.3) | 7.69 (0-30.8) | <0.0001 | <0.0001 | 0.0969 (-0.0291-0.277) | 0.000228 (-0.153-0.162) | <0.0001 | <0.0001 | 66.7 (42.3-93.4) | 48 (21.3-77.5) | <0.0001 | <0.0001 |  |
| Dobutamine | Increase | 18.8 (3.57-40.7) | 9.09 (0-28) | <0.0001 | <0.0001 | 0.103 (-0.0176-0.252) | 0.00998 (-0.143-0.146) | <0.0001 | <0.0001 | 69.2 (42.6-95.5) | 48.4 (22.4-76.9) | <0.0001 | <0.0001 |  |
| Midazolam | Decrease | 17.2 (0-37.9) | 4.35 (0-24) | <0.0001 | <0.0001 | 0.0759 (-0.0289-0.217) | 0.0157 (-0.115-0.144) | <0.0001 | <0.0001 | 65.4 (42.3-90.3) | 51.7 (25-77.8) | <0.0001 | <0.0001 |  |
| Midazolam | Increase | 17.2 (0-39.3) | 6.67 (0-24.1) | <0.0001 | <0.0001 | 0.076 (-0.028-0.228) | 0.0152 (-0.114-0.145) | <0.0001 | <0.0001 | 66.7 (40.8-92.9) | 51.9 (25-78.6) | <0.0001 | <0.0001 |  |
| Morphine | Decrease | 18.5 (3.45-40) | 7.41 (0-25.9) | <0.0001 | <0.0001 | 0.0887 (-0.0186-0.234) | 0.025 (-0.109-0.167) | <0.0001 | <0.0001 | 66.7 (44.4-91.7) | 54.2 (26.9-81.5) | <0.0001 | <0.0001 |  |
| Morphine | Increase | 17.9 (0-41.2) | 7.14 (0-25.9) | <0.0001 | <0.0001 | 0.0899 (-0.0178-0.243) | 0.0184 (-0.106-0.17) | <0.0001 | <0.0001 | 69.4 (44.8-93.8) | 53.6 (26.7-81.9) | <0.0001 | <0.0001 |  |
| Noradrenaline | Decrease | 17.9 (2.4-39.3) | 4.17 (0-25) | <0.0001 | <0.0001 | 0.0799 (-0.0281-0.226) | 0.0117 (-0.117-0.144) | <0.0001 | <0.0001 | 65.5 (41.4-91.5) | 51.9 (24.4-78.3) | <0.0001 | <0.0001 |  |
| Noradrenaline | Increase | 17.9 (0-40) | 4 (0-24.1) | <0.0001 | <0.0001 | 0.0817 (-0.0253-0.23) | 0.00848 (-0.12-0.137) | <0.0001 | <0.0001 | 66.7 (42.3-92.3) | 50.5 (24-77.1) | <0.0001 | <0.0001 |  |
| Propofol | Decrease | 20 (3.57-42.9) | 7.41 (0-29.2) | <0.0001 | <0.0001 | 0.0977 (-0.0184-0.256) | 0.0304 (-0.0991-0.189) | <0.0001 | <0.0001 | 69 (42.9-93.1) | 56 (28.6-83.3) | <0.0001 | <0.0001 |  |
| Propofol | Increase | 19.2 (3.57-42.9) | 7.14 (0-28.6) | <0.0001 | <0.0001 | 0.0975 (-0.0176-0.254) | 0.0278 (-0.0989-0.177) | <0.0001 | <0.0001 | 69.2 (43.3-93.9) | 55.6 (27.6-82.8) | <0.0001 | <0.0001 |  |
| Vasopressin | Decrease | 16.3 (14.1-20.9) | 14.6 (13.5-18) | 0.000637 | 0.0172 | 0.0357 (0.0245-0.0915) | 0.0267 (0.0245-0.0534) | 0.265 | 1 | 53.1 (51.5-61.2) | 51.6 (51.5-55.5) | 0.282 | 1 |  |
| Vasopressin | Increase | 16.3 (14.1-20.9) | 15.2 (13.9-16.3) | 0.0297 | 0.801 | 0.0357 (0.0245-0.0504) | 0.0267 (0.0245-0.0534) | 0.525 | 1 | 53.1 (51.5-57) | 51.6 (51.5-55.5) | 0.737 | 1 |  |
| **Name** | **Mean Dose Change** | **% time LPRx_30 > 0.25** | | | | **% time LPRx_30 > 0.35** | | | | **Mean LPRx_60** | | | |  |
|  |  | **Pre Dose** | **Post Dose** | **P value** | **Adj P Value** | **Pre Dose** | **Post Dose** | **P value** | **Adj P Value** | **Pre Dose** | **Post Dose** | **P value** | **Adj P Value** |  |
| Dobutamine | Decrease | 25.6 (3.67-59.1) | 11.7 (0-38) | <0.0001 | <0.0001 | 12.5 (0-42.9) | 0 (0-25.2) | <0.0001 | <0.0001 | 0.05 (-0.106-0.226) | 0.0324 (-0.123-0.233) | 0.327 | 1 |  |
| Dobutamine | Increase | 23.1 (2.59-53.3) | 11.5 (0-38.7) | <0.0001 | <0.0001 | 10 (0-36.7) | 0 (0-24.1) | <0.0001 | 0.000152 | 0.0533 (-0.0769-0.24) | 0.0241 (-0.122-0.206) | 0.0206 | 0.556 |  |
| Midazolam | Decrease | 19.4 (0-48) | 8.76 (0-33.3) | <0.0001 | <0.0001 | 3.85 (0-30.8) | 0 (0-19) | <0.0001 | <0.0001 | 0.0337 (-0.0951-0.194) | 0.0325 (-0.0876-0.185) | 0.732 | 1 |  |
| Midazolam | Increase | 19.7 (0-50) | 10 (0-34.7) | <0.0001 | <0.0001 | 4.76 (0-31.3) | 0 (0-21) | <0.0001 | <0.0001 | 0.0378 (-0.0955-0.206) | 0.0367 (-0.083-0.19) | 0.921 | 1 |  |
| Morphine | Decrease | 23.1 (0-50) | 11.5 (0-39.3) | <0.0001 | <0.0001 | 8.11 (0-34.5) | 0 (0-23.7) | <0.0001 | <0.0001 | 0.0515 (-0.0937-0.23) | 0.0495 (-0.0855-0.222) | 0.985 | 1 |  |
| Morphine | Increase | 22.2 (0-52.2) | 11.1 (0-39.3) | <0.0001 | <0.0001 | 6.9 (0-34.8) | 0 (0-24.1) | <0.0001 | <0.0001 | 0.0552 (-0.0881-0.232) | 0.0506 (-0.0856-0.225) | 0.454 | 1 |  |
| Noradrenaline | Decrease | 20.6 (0-50) | 7.69 (0-34.6) | <0.0001 | <0.0001 | 7.14 (0-32.3) | 0 (0-20) | <0.0001 | <0.0001 | 0.0342 (-0.0985-0.205) | 0.0295 (-0.098-0.195) | 0.104 | 1 |  |
| Noradrenaline | Increase | 20.6 (0-50) | 7.41 (0-33.3) | <0.0001 | <0.0001 | 7.14 (0-33.3) | 0 (0-19.4) | <0.0001 | <0.0001 | 0.0372 (-0.0901-0.208) | 0.032 (-0.0929-0.196) | 0.00232 | 0.0627 |  |
| Propofol | Decrease | 24 (0-55.2) | 12 (0-43.3) | <0.0001 | <0.0001 | 10.3 (0-38.7) | 0 (0-26.9) | <0.0001 | <0.0001 | 0.0598 (-0.0791-0.246) | 0.06 (-0.0718-0.249) | 0.225 | 1 |  |
| Propofol | Increase | 23.3 (0-53.9) | 11.8 (0-42.9) | <0.0001 | <0.0001 | 8.33 (0-38.5) | 0 (0-26.9) | <0.0001 | <0.0001 | 0.0623 (-0.0716-0.248) | 0.0601 (-0.0707-0.249) | 0.81 | 1 |  |
| Vasopressin | Decrease | 21.9 (19.7-27.4) | 20.6 (19.7-24) | 0.0825 | 1 | 13.3 (11.9-17.1) | 12.2 (8.92-14.8) | 0.0132 | 0.355 | 0.0214 (0.0102-0.0749) | 0.0215 (0.01-0.0398) | 0.86 | 1 |  |
| Vasopressin | Increase | 21.7 (19.7-24) | 20.5 (19.7-24) | 0.138 | 1 | 13.3 (11.9-17.1) | 12.1 (8-14.8) | 0.0795 | 1 | 0.0102 (-0.00237-0.0398) | 0.0215 (0.01-0.0398) | 0.792 | 1 |  |
| **Name** | **Mean Dose Change** | **% time LPRx_60 > 0** | | | | **% time LPRx_60 > 0.25** | | | | **% time LPRx_60 > 0.35** | | | |  |
|  |  | **Pre Dose** | **Post Dose** | **P value** | **Adj P Value** | **Pre Dose** | **Post Dose** | **P value** | **Adj P Value** | **Pre Dose** | **Post Dose** | **P value** | **Adj P Value** |  |
| Dobutamine | Decrease | 62.5 (15.4-100) | 60.8 (11.4-100) | 0.634 | 1 | 0 (0-48) | 0 (0-50) | 0.463 | 1 | 0 (0-27.1) | 0 (0-28.1) | 0.614 | 1 |  |
| Dobutamine | Increase | 69 (23.1-100) | 57.6 (9.31-100) | 0.0137 | 0.369 | 0 (0-50) | 0 (0-39.3) | 0.0479 | 1 | 0 (0-23) | 0 (0-21.2) | 0.0669 | 1 |  |
| Midazolam | Decrease | 61.5 (15.4-100) | 60.9 (16-100) | 0.698 | 1 | 0 (0-38.7) | 0 (0-36.7) | 0.303 | 1 | 0 (0-18.5) | 0 (0-15.8) | 0.0927 | 1 |  |
| Midazolam | Increase | 63 (13.8-100) | 62.1 (16-100) | 0.799 | 1 | 0 (0-40.1) | 0 (0-38) | 0.371 | 1 | 0 (0-20) | 0 (0-16.1) | 0.074 | 1 |  |
| Morphine | Decrease | 64.3 (18.6-100) | 65.2 (20.7-100) | 0.351 | 1 | 0 (0-50) | 0 (0-45.2) | 0.355 | 1 | 0 (0-27.6) | 0 (0-22.4) | 0.0825 | 1 |  |
| Morphine | Increase | 67.7 (19.3-100) | 66.7 (18.5-100) | 0.574 | 1 | 0 (0-48.3) | 0 (0-46.2) | 0.309 | 1 | 0 (0-25) | 0 (0-22.2) | 0.268 | 1 |  |
| Noradrenaline | Decrease | 60 (17.2-100) | 59.3 (12.5-100) | 0.139 | 1 | 0 (0-41.4) | 0 (0-38.5) | <0.0001 | <0.0001 | 0 (0-20) | 0 (0-14.8) | <0.0001 | <0.0001 |  |
| Noradrenaline | Increase | 61.9 (19.1-100) | 60.7 (12.9-100) | 0.00429 | 0.116 | 0 (0-42.9) | 0 (0-38.5) | <0.0001 | <0.0001 | 0 (0-20.8) | 0 (0-14.3) | <0.0001 | <0.0001 |  |
| Propofol | Decrease | 66.7 (22.2-100) | 67.9 (21.4-100) | 0.114 | 1 | 3.45 (0-51.9) | 0 (0-52.5) | 0.359 | 1 | 0 (0-29.9) | 0 (0-29.6) | 0.406 | 1 |  |
| Propofol | Increase | 68 (23.1-100) | 68 (20.8-100) | 0.84 | 1 | 3.57 (0-52) | 0 (0-53.2) | 0.0422 | 1 | 0 (0-29.9) | 0 (0-28) | 0.0435 | 1 |  |
| Vasopressin | Decrease | 51.5 (50.2-61.9) | 52.4 (49.9-54.3) | 0.654 | 1 | 14.8 (14.5-17.3) | 14.9 (2.34-16.9) | 0.773 | 1 | 9.67 (2.78-9.94) | 9.66 (0-10.8) | 0.846 | 1 |  |
| Vasopressin | Increase | 50.2 (49.9-56) | 51.5 (49.9-54.3) | 0.945 | 1 | 14.8 (7.97-15.2) | 14.5 (7.69-16.4) | 0.964 | 1 | 9.67 (3.38-9.94) | 9.66 (0-10.8) | 0.712 | 1 |  |
| **Bolus** | | | | | | | | | | | | | |  |
| **Name** | **Doses** | **Mean MAP** | | | | **Mean CPP** | | | | **% time CPP>60** | | | |  |
|  |  | **Pre Dose** | **Post Dose** | **P value** | **Adj P Value** | **Pre Dose** | **Post Dose** | **P value** | **Adj P Value** | **Pre Dose** | **Post Dose** | **P value** | **Adj P Value** |  |
| Ephedrine | 28 | 73 (63.6-88.2) | 78 (68.2-88.5) | 0.133 | 1 | 58.8 (53.3-75.6) | 62.4 (56.1-72.9) | 0.106 | 1 | 59.1 (3.08-100) | 44 (8.68-97.6) | 0.94 | 1 |  |
| Fentanyl | 115 | 81.4 (76.6-89.2) | 79.5 (71.9-87.9) | 0.0804 | 1 | 68.9 (61.5-76.9) | 67.9 (61.2-73.9) | 0.514 | 1 | 9.52 (0-43.2) | 11.1 (0-46.7) | 0.327 | 1 |  |
| Midazolam | 530 | 79.3 (74.6-85.8) | 78.5 (73.4-84.2) | 0.0485 | 1 | 63.2 (58.1-70) | 63 (57.6-67.9) | 0.252 | 1 | 22.9 (0-74.5) | 25 (0-76.4) | 0.395 | 1 |  |
| Morphine | 2476 | 82.1 (75.5-91.2) | 80.6 (74.1-89.9) | <0.0001 | 0.000265 | 69.4 (61.9-80) | 67.6 (60.9-77.5) | <0.0001 | 0.000123 | 0 (0-34.7) | 4.17 (0-46.4) | <0.0001 | 0.000225 |  |
| Propofol | 2480 | 80.8 (74.9-87.5) | 79.4 (73.8-86.2) | <0.0001 | <0.0001 | 66.3 (60.4-73.8) | 64.9 (59.9-72.6) | 0.000849 | 0.0229 | 6.34 (0-50) | 10.7 (0-53.8) | 0.00125 | 0.0337 |  |
| Alfentanil | 186 | 78.9 (73.2-85.9) | 77.1 (71.9-82.4) | 0.0575 | 1 | 67.3 (61.2-74.7) | 65.3 (60.6-71.8) | 0.0932 | 1 | 11 (0-41.1) | 14.6 (0-48.3) | 0.265 | 1 |  |
| **Name** | **% time CPP>70** | | | | **Mean ICP** | | | | **% time ICP>20** | | | |  |  |
|  | **Pre Dose** | **Post Dose** | **P value** | **Adj P Value** | **Pre Dose** | **Post Dose** | **P value** | **Adj P Value** | **Pre Dose** | **Post Dose** | **P value** | **Adj P Value** |  |  |
| Ephedrine | 2.5 (0-54.9) | 17.1 (0-49.7) | 0.379 | 1 | 11.5 (5.64-16) | 10.6 (5.78-16) | 0.993 | 1 | 0.122 (0-2.72) | 0 (0-2.01) | 0.398 | 1 |  |  |
| Fentanyl | 34.6 (7.41-84) | 27.6 (0-62.3) | 0.0646 | 1 | 13.9 (10.7-16.3) | 10.9 (7.74-15.6) | 0.0383 | 1 | 0 (0-16.7) | 0 (0-7.28) | 0.253 | 1 |  |  |
| Midazolam | 4.5 (0-49.4) | 3.57 (0-29.9) | 0.0892 | 1 | 16.5 (13.1-19.6) | 16.1 (12.8-19.3) | 0.208 | 1 | 4.17 (0-33.3) | 3.92 (0-26.9) | 0.309 | 1 |  |  |
| Morphine | 38 (2.38-100) | 27.6 (0-92.6) | <0.0001 | 0.000319 | 12.4 (8.62-16.4) | 12.8 (8.76-16.5) | 0.293 | 1 | 0 (0-5.04) | 0 (0-5.4) | 0.943 | 1 |  |  |
| Propofol | 14.6 (0-77.8) | 10.7 (0-67.5) | 0.00108 | 0.0293 | 14.2 (9.99-18) | 13.9 (9.59-17.8) | 0.105 | 1 | 0 (0-13.2) | 0 (0-11.5) | 0.00529 | 0.143 |  |  |
| Alfentanil | 18.5 (3.45-73.4) | 11.8 (0-63.8) | 0.246 | 1 | 11.5 (7.79-14.8) | 10.9 (8.15-14.1) | 0.864 | 1 | 0 (0-3.7) | 0 (0-3.67) | 0.59 | 1 |  |  |
| **Name** | **% time ICP>22** | | | | **Mean LPRx_10** | | | | **% time LPRx_10 > 0** | | | |  |  |
|  | **Pre Dose** | **Post Dose** | **P value** | **Adj P Value** | **Pre Dose** | **Post Dose** | **P value** | **Adj P Value** | **Pre Dose** | **Post Dose** | **P value** | **Adj P Value** |  |  |
| Ephedrine | 0 (0-0.635) | 0 (0-0.114) | 0.467 | 1 | 0.0742 (0.0132-0.198) | -0.0361 (-0.2-0.0114) | <0.0001 | 0.00175 | 60.8 (54-72.9) | 42.4 (29.8-51.5) | <0.0001 | 0.00174 |  |  |
| Fentanyl | 0 (0-7.41) | 0 (0-3.7) | 0.336 | 1 | 0.121 (0.0419-0.207) | 0.0178 (-0.0915-0.142) | <0.0001 | <0.0001 | 65.2 (59.1-76.6) | 55.2 (34.6-65) | <0.0001 | <0.0001 |  |  |
| Midazolam | 0 (0-11.1) | 0 (0-7.62) | 0.106 | 1 | 0.146 (0.051-0.27) | 0.0173 (-0.112-0.158) | <0.0001 | <0.0001 | 67.9 (57.7-80) | 52 (35.8-68.9) | <0.0001 | <0.0001 |  |  |
| Morphine | 0 (0-0.611) | 0 (0-0.757) | 0.592 | 1 | 0.161 (0.062-0.275) | 0.0349 (-0.086-0.181) | <0.0001 | <0.0001 | 69.2 (59.2-81.6) | 53.8 (38.5-72) | <0.0001 | <0.0001 |  |  |
| Propofol | 0 (0-4) | 0 (0-3.7) | 0.0177 | 0.479 | 0.158 (0.0633-0.265) | 0.0283 (-0.115-0.177) | <0.0001 | <0.0001 | 69 (59.3-80.8) | 53.3 (35.9-70.8) | <0.0001 | <0.0001 |  |  |
| Alfentanil | 0 (0-0.195) | 0 (0-0) | 0.564 | 1 | 0.171 (0.07-0.282) | 0.0218 (-0.103-0.173) | <0.0001 | <0.0001 | 71.1 (60.7-81.5) | 51.8 (38.2-69.2) | <0.0001 | <0.0001 |  |  |
| **Name** | **% time LPRx_10 > 0.25** | | | | **% time LPRx_10 > 0.35** | | | | **Mean LPRx_15** | | | |  |  |
|  | **Pre Dose** | **Post Dose** | **P value** | **Adj P Value** | **Pre Dose** | **Post Dose** | **P value** | **Adj P Value** | **Pre Dose** | **Post Dose** | **P value** | **Adj P Value** |  |  |
| Ephedrine | 32.9 (28.1-53.2) | 19.8 (3.81-32.3) | 0.000314 | 0.00848 | 26.4 (19.1-45.5) | 14.3 (2.42-25.7) | 0.000375 | 0.0101 | 0.0859 (0.0208-0.205) | -0.0278 (-0.209-0.0197) | <0.0001 | 0.000803 |  |  |
| Fentanyl | 40 (27.4-47.6) | 26.7 (11.5-42.9) | 0.000138 | 0.00372 | 26.7 (16.7-40.4) | 15.6 (3.51-29.8) | <0.0001 | 0.00243 | 0.112 (0.0612-0.191) | 0.00248 (-0.114-0.101) | <0.0001 | <0.0001 |  |  |
| Midazolam | 40.7 (28.4-56.5) | 27.1 (13.9-42.3) | <0.0001 | <0.0001 | 29.4 (16.8-43.9) | 17.9 (6.9-33) | <0.0001 | <0.0001 | 0.135 (0.0486-0.253) | 0.0182 (-0.107-0.144) | <0.0001 | <0.0001 |  |  |
| Morphine | 41.9 (29.2-56.5) | 29.1 (14.3-44.8) | <0.0001 | <0.0001 | 30.4 (17.9-45.8) | 20 (7.14-34.6) | <0.0001 | <0.0001 | 0.15 (0.05-0.272) | 0.0378 (-0.0876-0.182) | <0.0001 | <0.0001 |  |  |
| Propofol | 41.4 (28-56) | 27.7 (13.3-44.4) | <0.0001 | <0.0001 | 29.7 (16.7-45.8) | 19 (6.88-35.1) | <0.0001 | <0.0001 | 0.149 (0.052-0.26) | 0.0354 (-0.103-0.182) | <0.0001 | <0.0001 |  |  |
| Alfentanil | 42.9 (28.9-59.2) | 28.8 (14.3-42.8) | <0.0001 | <0.0001 | 30.9 (16.8-46.2) | 20 (7.48-33) | <0.0001 | <0.0001 | 0.148 (0.0473-0.266) | 0.0337 (-0.107-0.175) | <0.0001 | <0.0001 |  |  |
| **Name** | **% time LPRx_15 > 0** | | | | **% time LPRx_15 > 0.25** | | | | **% time LPRx_15 > 0.35** | | | |  |  |
|  | **Pre Dose** | **Post Dose** | **P value** | **Adj P Value** | **Pre Dose** | **Post Dose** | **P value** | **Adj P Value** | **Pre Dose** | **Post Dose** | **P value** | **Adj P Value** |  |  |
| Ephedrine | 61.7 (51.6-76) | 48 (25-51.3) | 0.000128 | 0.00344 | 33.3 (29.4-50.7) | 19.2 (3.26-30.6) | 0.00537 | 0.145 | 23.3 (15.2-46.8) | 16.4 (0-23.1) | 0.00385 | 0.104 |  |  |
| Fentanyl | 66.7 (56.6-78.3) | 51.7 (31.9-67.3) | <0.0001 | <0.0001 | 30 (19.6-44.4) | 18.5 (4.55-44) | 0.00125 | 0.0338 | 16 (7.32-33.3) | 11.1 (0-26.7) | 0.00914 | 0.247 |  |  |
| Midazolam | 69 (56.5-84.8) | 52.2 (34.9-69.2) | <0.0001 | <0.0001 | 34.7 (21.9-55.7) | 22.5 (7.69-41.4) | <0.0001 | <0.0001 | 23.1 (10.3-43.5) | 13.6 (0-27.6) | <0.0001 | <0.0001 |  |  |
| Morphine | 70.8 (56.7-86.7) | 55.2 (36.7-74.1) | <0.0001 | <0.0001 | 37.3 (23.7-57.1) | 25.6 (7.69-44.1) | <0.0001 | <0.0001 | 24.5 (11.1-44.4) | 14.8 (0-32.1) | <0.0001 | <0.0001 |  |  |
| Propofol | 70.8 (57.1-84.6) | 55 (34.9-73.3) | <0.0001 | <0.0001 | 37.5 (21.4-55.6) | 24.1 (6.98-43.4) | <0.0001 | <0.0001 | 24 (8.65-43.5) | 13.8 (0-31.8) | <0.0001 | <0.0001 |  |  |
| Alfentanil | 72.4 (57.9-85.1) | 55.3 (34.5-77.9) | <0.0001 | <0.0001 | 35.4 (19.3-58.2) | 24 (8.17-42.9) | <0.0001 | 0.000447 | 22.2 (8.33-46) | 14.3 (0-30.6) | 0.000282 | 0.00763 |  |  |
| **Name** | **Mean LPRx_20** | | | | **% time LPRx_20 > 0** | | | | **% time LPRx_20 > 0.25** | | | |  |  |
|  | **Pre Dose** | **Post Dose** | **P value** | **Adj P Value** | **Pre Dose** | **Post Dose** | **P value** | **Adj P Value** | **Pre Dose** | **Post Dose** | **P value** | **Adj P Value** |  |  |
| Ephedrine | 0.105 (0.0288-0.162) | 0.00213 (-0.186-0.0343) | <0.0001 | 0.00163 | 60.5 (51.7-69.6) | 46 (20.5-51.6) | <0.0001 | 0.00137 | 30.2 (29.2-45.1) | 23 (0-29.6) | 0.00269 | 0.0726 |  |  |
| Fentanyl | 0.0943 (0.0286-0.156) | 0.00506 (-0.0893-0.122) | <0.0001 | 0.000801 | 65.4 (53-81.5) | 51.2 (36-66.3) | <0.0001 | 0.00235 | 27.3 (12-42.9) | 14.3 (1.79-39.9) | 0.0117 | 0.317 |  |  |
| Midazolam | 0.13 (0.0425-0.242) | 0.0194 (-0.112-0.129) | <0.0001 | <0.0001 | 69.6 (54.2-87.5) | 52.4 (32.1-72) | <0.0001 | <0.0001 | 32.1 (15.3-54.1) | 20 (0-37.9) | <0.0001 | <0.0001 |  |  |
| Morphine | 0.142 (0.0378-0.271) | 0.0417 (-0.0905-0.188) | <0.0001 | <0.0001 | 72.4 (54.7-91.5) | 56.2 (34.5-78.7) | <0.0001 | <0.0001 | 33.3 (17.1-57.1) | 22.4 (3.33-43.9) | <0.0001 | <0.0001 |  |  |
| Propofol | 0.133 (0.0376-0.26) | 0.0417 (-0.0963-0.184) | <0.0001 | <0.0001 | 72.1 (54.8-88.9) | 56.4 (34.5-78.6) | <0.0001 | <0.0001 | 33.3 (14.8-55.2) | 21.4 (2.52-42.9) | <0.0001 | <0.0001 |  |  |
| Alfentanil | 0.125 (0.0299-0.252) | 0.0434 (-0.0927-0.152) | <0.0001 | <0.0001 | 71.7 (56.2-86.6) | 55.4 (32.4-78.1) | <0.0001 | <0.0001 | 34 (10.8-53.1) | 22.4 (5.38-41.9) | 0.00165 | 0.0446 |  |  |
| **Name** | **% time LPRx_20 > 0.35** | | | | **Mean LPRx_30** | | | | **% time LPRx_30 > 0** | | | |  |  |
|  | **Pre Dose** | **Post Dose** | **P value** | **Adj P Value** | **Pre Dose** | **Post Dose** | **P value** | **Adj P Value** | **Pre Dose** | **Post Dose** | **P value** | **Adj P Value** |  |  |
| Ephedrine | 21.7 (6.58-33.7) | 20.8 (0-26.4) | 0.0521 | 1 | 0.0654 (-0.0024-0.191) | 0.0321 (-0.137-0.117) | 0.031 | 0.836 | 53.8 (38.8-87) | 52.5 (30.5-60.5) | 0.0942 | 1 |  |  |
| Fentanyl | 12.5 (0-31.8) | 7.69 (0-24.2) | 0.0827 | 1 | 0.0462 (-0.0584-0.2) | 0.0418 (-0.0891-0.213) | 0.561 | 1 | 56.5 (32.2-89.6) | 56.7 (29.6-73.5) | 0.469 | 1 |  |  |
| Midazolam | 18.6 (3.74-38.5) | 8.33 (0-24.8) | <0.0001 | <0.0001 | 0.113 (-0.00455-0.24) | 0.0257 (-0.0974-0.144) | <0.0001 | <0.0001 | 71 (48-93.1) | 55.2 (26.9-78.5) | <0.0001 | <0.0001 |  |  |
| Morphine | 20 (4-42.9) | 10.7 (0-30.8) | <0.0001 | <0.0001 | 0.117 (0.00227-0.273) | 0.0513 (-0.0918-0.193) | <0.0001 | <0.0001 | 71.2 (47.6-96.4) | 58.2 (30.8-86.4) | <0.0001 | <0.0001 |  |  |
| Propofol | 19.2 (3.57-41) | 9.2 (0-30.3) | <0.0001 | <0.0001 | 0.11 (-0.0032-0.257) | 0.0516 (-0.08-0.194) | <0.0001 | <0.0001 | 71.9 (47.8-95.9) | 59.1 (32.5-88) | <0.0001 | <0.0001 |  |  |
| Alfentanil | 17.6 (0.806-43.6) | 10.5 (0-28.3) | 0.00551 | 0.149 | 0.0981 (0.00642-0.24) | 0.0419 (-0.0843-0.195) | 0.00127 | 0.0344 | 65.4 (48-92) | 58.9 (31.7-86.1) | 0.0215 | 0.58 |  |  |
| **Name** | **% time LPRx_30 > 0.25** | | | | **% time LPRx_30 > 0.35** | | | | **Mean LPRx_60** | | | |  |  |
|  | **Pre Dose** | **Post Dose** | **P value** | **Adj P Value** | **Pre Dose** | **Post Dose** | **P value** | **Adj P Value** | **Pre Dose** | **Post Dose** | **P value** | **Adj P Value** |  |  |
| Ephedrine | 29.9 (3.75-48.6) | 23 (7.41-31.2) | 0.365 | 1 | 22.9 (0-30.5) | 10.5 (0-22.7) | 0.496 | 1 | 0.0684 (0.0184-0.115) | 0.0426 (-0.0258-0.243) | 0.787 | 1 |  |  |
| Fentanyl | 14.7 (0-40) | 20.7 (0-50.8) | 0.43 | 1 | 5 (0-29.1) | 3.57 (0-29.1) | 0.804 | 1 | 0.0502 (-0.121-0.232) | 0.038 (-0.0596-0.225) | 0.841 | 1 |  |  |
| Midazolam | 28 (4-53.6) | 14.7 (0-37.9) | <0.0001 | <0.0001 | 10.3 (0-34.7) | 0 (0-21.5) | <0.0001 | 0.00054 | 0.107 (-0.0972-0.241) | 0.0625 (-0.0814-0.217) | 0.125 | 1 |  |  |
| Morphine | 28 (3.85-57.7) | 17.9 (0-44.4) | <0.0001 | <0.0001 | 13.8 (0-40) | 4 (0-28.6) | <0.0001 | <0.0001 | 0.079 (-0.0806-0.274) | 0.0684 (-0.0843-0.248) | 0.0989 | 1 |  |  |
| Propofol | 25 (2.27-55.6) | 16.1 (0-45.2) | <0.0001 | <0.0001 | 10.7 (0-39.1) | 2.27 (0-26.7) | <0.0001 | <0.0001 | 0.0854 (-0.0687-0.266) | 0.0735 (-0.0626-0.26) | 0.514 | 1 |  |  |
| Alfentanil | 24.6 (4.16-51.7) | 15.1 (0-34.3) | 0.0027 | 0.073 | 10.3 (0-34.9) | 0 (0-19.1) | 0.00505 | 0.136 | 0.082 (-0.0548-0.211) | 0.0467 (-0.0926-0.213) | 0.265 | 1 |  |  |
| **Name** | **% time LPRx_60 > 0** | | | | **% time LPRx_60 > 0.25** | | | | **% time LPRx_60 > 0.35** | | | |  |  |
|  | **Pre Dose** | **Post Dose** | **P value** | **Adj P Value** | **Pre Dose** | **Post Dose** | **P value** | **Adj P Value** | **Pre Dose** | **Post Dose** | **P value** | **Adj P Value** |  |  |
| Ephedrine | 55 (41.1-86) | 55.2 (43.4-100) | 0.748 | 1 | 32.2 (0-44.7) | 29.7 (0-61.9) | 0.815 | 1 | 23.5 (0-30.9) | 23 (0-47.8) | 0.939 | 1 |  |  |
| Fentanyl | 69 (9.09-100) | 66.7 (25-100) | 0.917 | 1 | 3.85 (0-45.8) | 0 (0-59) | 0.534 | 1 | 0 (0-29.9) | 0 (0-39.3) | 0.667 | 1 |  |  |
| Midazolam | 70.8 (26.1-100) | 64.1 (25.7-100) | 0.345 | 1 | 14 (0-55) | 6.57 (0-46) | 0.215 | 1 | 0 (0-30.8) | 0 (0-26.1) | 0.11 | 1 |  |  |
| Morphine | 70 (25.9-100) | 66.7 (20.8-100) | 0.376 | 1 | 13 (0-57.7) | 6.74 (0-52.3) | 0.0091 | 0.246 | 0 (0-35.5) | 0 (0-29.1) | 0.00157 | 0.0424 |  |  |
| Propofol | 73.9 (28-100) | 74.1 (26.8-100) | 0.905 | 1 | 7.69 (0-57.1) | 3.45 (0-56.4) | 0.0658 | 1 | 0 (0-33.3) | 0 (0-26.9) | 0.0136 | 0.367 |  |  |
| Alfentanil | 70.2 (36-100) | 64.3 (15.9-100) | 0.745 | 1 | 10.7 (0-48.1) | 0 (0-43.7) | 0.202 | 1 | 0 (0-25.6) | 0 (0-14.2) | 0.138 | 1 |  |  |

*The table demonstrates the median and interquartile range of the pre/pose dose windows as well as the Wilcox signed ranked test between these windows with p values adjusted using the Bonferroni analysis. The “change” indicates if the continuous infusion was increase/decrease or was a bolus dose. CPP, cerebral prefusion pressure; ICP, intracranial pressure; LPRx_10, pressure reactivity over 10 minutes; LPRx_15, pressure reactivity over 15 minutes; LPRx_20, pressure reactivity over 20 minutes; LPRx_30, pressure reactivity over 30 minutes; LPRx_60, pressure reactivity over 60 minutes;*

# Appendix J. Pre-time window over 50% time L-PRx_10 < 0

The table contains all infusions given with the LPRx_10 pre-time window 50% < 0, separated into the continuous infusion doses then the bolus doses.

| **Continuous Intravenous** | | | | | | | | | | | | | | |
| --- | --- | --- | --- | --- | --- | --- | --- | --- | --- | --- | --- | --- | --- | --- |
| **Name** | **Doses** | **Mean Dose Change** | **Mean MAP** | | | | **Mean CPP** | | | | **% time CPP>60** | | | |
|  |  |  | **Pre Dose** | **Post Dose** | **P value** | **Adj P Value** | **Pre Dose** | **Post Dose** | **P value** | **Adj P Value** | **Pre Dose** | **Post Dose** | **P value** | **Adj P Value** |
| Dobutamine | 745 | Decrease | 76.8 (71.4-84.9) | 76.6 (70.8-83.8) | 0.292 | 1 | 64.7 (58.5-70.9) | 63.2 (59.1-69.7) | 0.238 | 1 | 15.4 (0-72.7) | 15.4 (0-65.4) | 0.683 | 1 |
| Dobutamine | 610 | Increase | 73.4 (68.2-81.5) | 73 (68.3-80.9) | 0.734 | 1 | 60.4 (53.9-66.9) | 59.7 (53.9-65.9) | 0.639 | 1 | 55.6 (8.7-100) | 56.3 (4.88-100) | 0.829 | 1 |
| Midazolam | 2547 | Decrease | 78.5 (73.1-84.7) | 78.4 (73-84.5) | 0.449 | 1 | 65.7 (60.1-71.3) | 65 (59.8-71) | 0.246 | 1 | 8.7 (0-51.9) | 9.38 (0-56) | 0.578 | 1 |
| Midazolam | 2444 | Increase | 79.2 (73.8-85.2) | 78.9 (73.5-84.9) | 0.274 | 1 | 64.8 (59.5-71.1) | 64.7 (59.3-71) | 0.621 | 1 | 12 (0-59.3) | 10.7 (0-63) | 0.581 | 1 |
| Morphine | 3881 | Decrease | 77.8 (72.3-84.2) | 77.7 (72.2-83.9) | 0.727 | 1 | 65.8 (60-71.7) | 65.4 (59.8-71.8) | 0.429 | 1 | 8.7 (0-54.5) | 8 (0-57.5) | 0.881 | 1 |
| Morphine | 3747 | Increase | 78.3 (72.7-84.8) | 77.5 (72.3-83.9) | 0.0014 | 0.0379 | 65.7 (59.7-72) | 65 (59.5-71.4) | 0.0125 | 0.337 | 8.33 (0-56.5) | 9.52 (0-60.7) | 0.471 | 1 |
| Noradrenaline | 25728 | Decrease | 79.1 (73.6-85.7) | 78.6 (73.1-85.1) | <0.0001 | <0.0001 | 66.5 (60.9-72.9) | 66 (60.7-72.4) | <0.0001 | <0.0001 | 7.14 (0-42.9) | 6.9 (0-45) | 0.542 | 1 |
| Noradrenaline | 24013 | Increase | 76.6 (71.2-83.3) | 76.8 (71.5-83.5) | 0.00708 | 0.191 | 63.5 (58.2-69.7) | 64.1 (58.5-70.4) | <0.0001 | <0.0001 | 24 (0-73.1) | 15.8 (0-72.4) | <0.0001 | <0.0001 |
| Propofol | 7976 | Decrease | 77.8 (72.1-84.3) | 77.6 (72.1-84.1) | 0.652 | 1 | 65.5 (59.8-71.8) | 65.2 (59.5-71.8) | 0.134 | 1 | 10.3 (0-55.6) | 10.7 (0-60) | 0.7 | 1 |
| Propofol | 7147 | Increase | 78.6 (73-85.4) | 77.7 (72.2-84.3) | <0.0001 | <0.0001 | 66.1 (60.1-72.5) | 65.2 (59.7-71.8) | <0.0001 | 0.000163 | 8.33 (0-52) | 9.68 (0-56.7) | 0.115 | 1 |
| Vasopressin | 20 | Increase | 71.8 (70.1-74.8) | 72.2 (70.6-76.3) | 0.478 | 1 | 52.6 (50.1-55.5) | 53.5 (50.1-57.8) | 0.583 | 1 | 100 (93.1-100) | 100 (75-100) | 0.715 | 1 |
| Vasopressin | 16 | Decrease | 73.9 (68.9-80.6) | 74.6 (67.6-78.9) | 0.642 | 1 | 57.1 (51.4-62) | 56.9 (49.4-65) | 1 | 1 | 81.7 (28.7-100) | 98.4 (15-100) | 0.798 | 1 |
| **Name** | **Mean Dose Change** | **% time CPP>70** | | | | **Mean ICP** | | | | **% time ICP>20** | | | |  |
|  |  | **Pre Dose** | **Post Dose** | **P value** | **Adj P Value** | **Pre Dose** | **Post Dose** | **P value** | **Adj P Value** | **Pre Dose** | **Post Dose** | **P value** | **Adj P Value** |  |
| Dobutamine | Decrease | 6.9 (0-50) | 3.57 (0-41.7) | 0.0302 | 0.815 | 13.8 (9.65-16.8) | 13.4 (9.6-16.4) | 0.381 | 1 | 0 (0-6.25) | 0 (0-3.33) | 0.0172 | 0.465 |  |
| Dobutamine | Increase | 0 (0-20.6) | 0 (0-18.9) | 0.363 | 1 | 14.8 (11-17.7) | 14.6 (11-17.1) | 0.625 | 1 | 0 (0-11.5) | 0 (0-6.63) | 0.122 | 1 |  |
| Midazolam | Decrease | 8 (0-55.1) | 6.98 (0-57.1) | 0.143 | 1 | 13.3 (9.94-16.6) | 13.4 (9.74-16.5) | 0.855 | 1 | 0 (0-5.94) | 0 (0-4.35) | 0.575 | 1 |  |
| Midazolam | Increase | 7.41 (0-54.2) | 6.25 (0-56) | 0.0979 | 1 | 14.4 (10.8-17.8) | 14.3 (10.6-17.6) | 0.241 | 1 | 0 (0-11.5) | 0 (0-8.33) | 0.00161 | 0.0435 |  |
| Morphine | Decrease | 10.3 (0-59.6) | 8 (0-63) | 0.293 | 1 | 12.4 (8.73-15.9) | 12.4 (8.83-15.9) | 0.633 | 1 | 0 (0-3.57) | 0 (0-3.33) | 0.392 | 1 |  |
| Morphine | Increase | 11.1 (0-61.7) | 7.14 (0-59.3) | 0.000582 | 0.0157 | 12.8 (9.1-16.4) | 12.7 (8.86-16.2) | 0.191 | 1 | 0 (0-4.35) | 0 (0-3.57) | 0.00111 | 0.03 |  |
| Noradrenaline | Decrease | 15.4 (0-70.4) | 10.4 (0-67.9) | <0.0001 | <0.0001 | 12.9 (9.27-16.3) | 12.9 (9.33-16.2) | 0.291 | 1 | 0 (0-4) | 0 (0-3.45) | <0.0001 | <0.0001 |  |
| Noradrenaline | Increase | 5 (0-42.9) | 3.85 (0-50) | 0.0256 | 0.692 | 13.6 (9.83-16.9) | 13.3 (9.57-16.6) | <0.0001 | <0.0001 | 0 (0-7.41) | 0 (0-3.85) | <0.0001 | <0.0001 |  |
| Propofol | Decrease | 10 (0-60) | 7.14 (0-62.1) | 0.0112 | 0.301 | 12.3 (8.76-16) | 12.6 (8.91-16.2) | 0.158 | 1 | 0 (0-3.7) | 0 (0-3.57) | 0.89 | 1 |  |
| Propofol | Increase | 13 (0-65.4) | 7.41 (0-61.7) | <0.0001 | <0.0001 | 12.8 (9.1-16.5) | 12.6 (8.93-16.2) | 0.00719 | 0.194 | 0 (0-6.9) | 0 (0-3.57) | <0.0001 | <0.0001 |  |
| Vasopressin | Increase | 0 (0-0) | 0 (0-0) | 0.965 | 1 | 19 (17.5-19.7) | 19.1 (18-19.8) | 0.738 | 1 | 9.17 (0-58.2) | 14.5 (2.42-45.5) | 0.891 | 1 |  |
| Vasopressin | Decrease | 0 (0-6.51) | 0 (0-0) | 0.0916 | 1 | 17.5 (15.3-18.7) | 16.3 (14.6-18.3) | 0.491 | 1 | 0 (0-6.23) | 0 (0-4.5) | 0.845 | 1 |  |
| **Name** | **Mean Dose Change** | **% time ICP>22** | | | | **Mean LPRx_10** | | | | **% time LPRx_10 > 0** | | | |  |
|  |  | **Pre Dose** | **Post Dose** | **P value** | **Adj P Value** | **Pre Dose** | **Post Dose** | **P value** | **Adj P Value** | **Pre Dose** | **Post Dose** | **P value** | **Adj P Value** |  |
| Dobutamine | Decrease | 0 (0-0) | 0 (0-0) | 0.0149 | 0.403 | -0.161 (-0.286--0.0705) | -0.00639 (-0.166-0.135) | <0.0001 | <0.0001 | 29.6 (17.2-40) | 48 (29.2-66.7) | <0.0001 | <0.0001 |  |
| Dobutamine | Increase | 0 (0-0) | 0 (0-0) | 0.76 | 1 | -0.165 (-0.288--0.0698) | -0.0189 (-0.21-0.133) | <0.0001 | <0.0001 | 30.8 (15.4-40) | 46.6 (26.9-65.4) | <0.0001 | <0.0001 |  |
| Midazolam | Decrease | 0 (0-0) | 0 (0-0) | 0.342 | 1 | -0.167 (-0.287--0.0711) | -0.0149 (-0.181-0.148) | <0.0001 | <0.0001 | 29.6 (17.2-40.7) | 48.1 (29.5-66.7) | <0.0001 | <0.0001 |  |
| Midazolam | Increase | 0 (0-3.57) | 0 (0-0) | 0.00141 | 0.038 | -0.159 (-0.295--0.0699) | -0.0248 (-0.184-0.136) | <0.0001 | <0.0001 | 30.4 (16.7-40.7) | 47.7 (28.6-65.5) | <0.0001 | <0.0001 |  |
| Morphine | Decrease | 0 (0-0) | 0 (0-0) | 0.418 | 1 | -0.153 (-0.271--0.066) | -0.00654 (-0.161-0.139) | <0.0001 | <0.0001 | 31 (19.2-40.7) | 49 (30.8-66.7) | <0.0001 | <0.0001 |  |
| Morphine | Increase | 0 (0-0) | 0 (0-0) | 0.000875 | 0.0236 | -0.156 (-0.277--0.0687) | -0.0155 (-0.173-0.144) | <0.0001 | <0.0001 | 30 (17.4-40) | 48.1 (29.6-66.7) | <0.0001 | <0.0001 |  |
| Noradrenaline | Decrease | 0 (0-0) | 0 (0-0) | <0.0001 | <0.0001 | -0.157 (-0.275--0.0653) | -0.0134 (-0.176-0.139) | <0.0001 | <0.0001 | 30.8 (18.5-40.7) | 48.1 (28.9-66.7) | <0.0001 | <0.0001 |  |
| Noradrenaline | Increase | 0 (0-0) | 0 (0-0) | <0.0001 | <0.0001 | -0.161 (-0.278--0.0679) | -0.02 (-0.181-0.139) | <0.0001 | <0.0001 | 30.8 (17.9-40.5) | 47.8 (28.6-65.5) | <0.0001 | <0.0001 |  |
| Propofol | Decrease | 0 (0-0) | 0 (0-0) | 0.144 | 1 | -0.156 (-0.275--0.067) | -0.00983 (-0.168-0.142) | <0.0001 | <0.0001 | 31 (19.2-40.7) | 48.3 (30-66.7) | <0.0001 | <0.0001 |  |
| Propofol | Increase | 0 (0-0) | 0 (0-0) | <0.0001 | <0.0001 | -0.154 (-0.274--0.0651) | -0.0116 (-0.176-0.145) | <0.0001 | <0.0001 | 31 (18.5-40.7) | 48.1 (29.6-66.7) | <0.0001 | <0.0001 |  |
| Vasopressin | Increase | 3.28 (0-11) | 6.45 (0-10.5) | 0.593 | 1 | -0.285 (-0.436--0.082) | 0.0208 (-0.12-0.0635) | 0.000228 | 0.00615 | 25.4 (11.3-39.3) | 45.5 (39.8-61.3) | 0.000121 | 0.00326 |  |
| Vasopressin | Decrease | 0 (0-2.42) | 0 (0-0.806) | 0.71 | 1 | -0.105 (-0.247-0.000898) | 0.0522 (-0.13-0.179) | 0.0426 | 1 | 35.9 (24.7-42.1) | 57.4 (34.8-71.5) | 0.0088 | 0.238 |  |
| **Name** | **Mean Dose Change** | **% time LPRx_10 > 0.25** | | | | **% time LPRx_10 > 0.35** | | | | **Mean LPRx_15** | | | |  |
|  |  | **Pre Dose** | **Post Dose** | **P value** | **Adj P Value** | **Pre Dose** | **Post Dose** | **P value** | **Adj P Value** | **Pre Dose** | **Post Dose** | **P value** | **Adj P Value** |  |
| Dobutamine | Decrease | 10 (0-20) | 25 (7.41-40.7) | <0.0001 | <0.0001 | 4.17 (0-12.9) | 15.9 (3.23-32.6) | <0.0001 | <0.0001 | -0.161 (-0.284--0.0637) | -0.0277 (-0.182-0.126) | <0.0001 | <0.0001 |  |
| Dobutamine | Increase | 11.2 (0-21.7) | 24 (8.53-40.3) | <0.0001 | <0.0001 | 4.35 (0-15.4) | 16 (3.45-32) | <0.0001 | <0.0001 | -0.158 (-0.292--0.0549) | -0.0271 (-0.209-0.121) | <0.0001 | <0.0001 |  |
| Midazolam | Decrease | 10 (0-20) | 23.1 (7.23-41.4) | <0.0001 | <0.0001 | 4 (0-14.3) | 14.8 (3.23-32.1) | <0.0001 | <0.0001 | -0.148 (-0.27--0.0566) | -0.0188 (-0.164-0.12) | <0.0001 | <0.0001 |  |
| Midazolam | Increase | 10 (0-20) | 22.2 (7.14-40.7) | <0.0001 | <0.0001 | 3.85 (0-14.3) | 14.3 (2.29-31) | <0.0001 | <0.0001 | -0.147 (-0.27--0.0517) | -0.0243 (-0.168-0.122) | <0.0001 | <0.0001 |  |
| Morphine | Decrease | 10.3 (0-20.7) | 23.3 (7.69-40.7) | <0.0001 | <0.0001 | 4 (0-14.3) | 14.8 (3.45-32) | <0.0001 | <0.0001 | -0.14 (-0.255--0.0527) | -0.0101 (-0.15-0.126) | <0.0001 | <0.0001 |  |
| Morphine | Increase | 9.09 (0-20) | 23.1 (7.69-40.7) | <0.0001 | <0.0001 | 3.85 (0-13.8) | 14.3 (3.45-32) | <0.0001 | <0.0001 | -0.144 (-0.259--0.0538) | -0.0121 (-0.159-0.13) | <0.0001 | <0.0001 |  |
| Noradrenaline | Decrease | 10.3 (0-20) | 22.4 (7.41-40.7) | <0.0001 | <0.0001 | 4 (0-14.3) | 14.3 (3.03-31) | <0.0001 | <0.0001 | -0.148 (-0.262--0.0556) | -0.0191 (-0.169-0.12) | <0.0001 | <0.0001 |  |
| Noradrenaline | Increase | 10 (0-20) | 22.6 (7.41-40.7) | <0.0001 | <0.0001 | 3.85 (0-13.8) | 14.3 (3.33-31.8) | <0.0001 | <0.0001 | -0.148 (-0.262--0.0554) | -0.0258 (-0.175-0.118) | <0.0001 | <0.0001 |  |
| Propofol | Decrease | 10.7 (0-20.8) | 24 (8-41.4) | <0.0001 | <0.0001 | 4.17 (0-14.3) | 15.1 (3.45-32.1) | <0.0001 | <0.0001 | -0.144 (-0.26--0.0492) | -0.00478 (-0.156-0.138) | <0.0001 | <0.0001 |  |
| Propofol | Increase | 10.7 (0-20.7) | 23.1 (7.69-41.3) | <0.0001 | <0.0001 | 4 (0-14.3) | 14.8 (3.45-32) | <0.0001 | <0.0001 | -0.142 (-0.261--0.0492) | -0.0114 (-0.164-0.137) | <0.0001 | <0.0001 |  |
| Vasopressin | Increase | 0 (0-20.8) | 15.5 (10.8-32.8) | 0.00673 | 0.182 | 0 (0-13.8) | 11.6 (7.17-26) | 0.00517 | 0.14 | -0.211 (-0.348--0.0503) | -0.00238 (-0.075-0.0715) | 0.00321 | 0.0867 |  |
| Vasopressin | Decrease | 14.9 (6.9-26) | 30.1 (19.5-48.7) | 0.0237 | 0.64 | 11 (3.45-21.8) | 21.7 (9.68-36.4) | 0.0897 | 1 | -0.1 (-0.248-0.0237) | 0.102 (-0.107-0.185) | 0.0318 | 0.86 |  |
| **Name** | **Mean Dose Change** | **% time LPRx_15 > 0** | | | | **% time LPRx_15 > 0.25** | | | | **% time LPRx_15 > 0.35** | | | |  |
|  |  | **Pre Dose** | **Post Dose** | **P value** | **Adj P Value** | **Pre Dose** | **Post Dose** | **P value** | **Adj P Value** | **Pre Dose** | **Post Dose** | **P value** | **Adj P Value** |  |
| Dobutamine | Decrease | 26.9 (11.4-40.7) | 46.4 (24.4-67.9) | <0.0001 | <0.0001 | 3.85 (0-16.7) | 18.5 (0-37.5) | <0.0001 | <0.0001 | 0 (0-8.89) | 9.52 (0-27.6) | <0.0001 | <0.0001 |  |
| Dobutamine | Increase | 27.3 (11.1-41.4) | 44.9 (21-66.7) | <0.0001 | <0.0001 | 4.4 (0-16.9) | 17 (2.25-37.8) | <0.0001 | <0.0001 | 0 (0-10) | 8.7 (0-26.9) | <0.0001 | <0.0001 |  |
| Midazolam | Decrease | 28.6 (13.3-41.9) | 47.9 (25.9-66.7) | <0.0001 | <0.0001 | 3.7 (0-15.8) | 15.9 (0-36) | <0.0001 | <0.0001 | 0 (0-8) | 7.14 (0-25.8) | <0.0001 | <0.0001 |  |
| Midazolam | Increase | 28 (12.5-42.3) | 48.1 (25-66.7) | <0.0001 | <0.0001 | 3.85 (0-16.7) | 16.7 (0-36) | <0.0001 | <0.0001 | 0 (0-8.33) | 7.14 (0-25) | <0.0001 | <0.0001 |  |
| Morphine | Decrease | 29.6 (15.4-42.9) | 48.4 (27.6-66.7) | <0.0001 | <0.0001 | 4 (0-17.1) | 17.9 (3.23-37.2) | <0.0001 | <0.0001 | 0 (0-9.09) | 7.69 (0-26.9) | <0.0001 | <0.0001 |  |
| Morphine | Increase | 28 (13.8-42.3) | 48.3 (26.9-67.9) | <0.0001 | <0.0001 | 3.7 (0-16.3) | 17.9 (2.38-37.5) | <0.0001 | <0.0001 | 0 (0-8.33) | 7.69 (0-26.7) | <0.0001 | <0.0001 |  |
| Noradrenaline | Decrease | 28.6 (14.3-41.4) | 46.4 (25.9-66.7) | <0.0001 | <0.0001 | 3.85 (0-16) | 16 (0-36) | <0.0001 | <0.0001 | 0 (0-8) | 7.14 (0-25) | <0.0001 | <0.0001 |  |
| Noradrenaline | Increase | 28 (13.8-41.4) | 46.4 (25-66.7) | <0.0001 | <0.0001 | 3.7 (0-15.6) | 16.1 (0-35.7) | <0.0001 | <0.0001 | 0 (0-7.69) | 7.14 (0-25) | <0.0001 | <0.0001 |  |
| Propofol | Decrease | 29.6 (15-42.9) | 48.3 (28.6-69.1) | <0.0001 | <0.0001 | 4.35 (0-17.4) | 18.5 (3.45-38.5) | <0.0001 | <0.0001 | 0 (0-10.3) | 9.68 (0-27.6) | <0.0001 | <0.0001 |  |
| Propofol | Increase | 29.2 (14.8-42.3) | 48.3 (28.1-69) | <0.0001 | <0.0001 | 4 (0-16.7) | 18.2 (3.45-38) | <0.0001 | <0.0001 | 0 (0-8.33) | 8 (0-27.1) | <0.0001 | <0.0001 |  |
| Vasopressin | Increase | 29 (5.96-45.3) | 45 (37.9-53.9) | 0.0274 | 0.74 | 0 (0-15.8) | 7.85 (3.23-31.4) | 0.0187 | 0.506 | 0 (0-8.85) | 3.85 (0-27.1) | 0.0675 | 1 |  |
| Vasopressin | Decrease | 35 (10.3-50) | 56.4 (41.1-67.7) | 0.0898 | 1 | 5.17 (0-26.6) | 30.9 (9-43.1) | 0.0396 | 1 | 0 (0-17.3) | 19.2 (3-33) | 0.0466 | 1 |  |
| **Name** | **Mean Dose Change** | **Mean LPRx_20** | | | | **% time LPRx_20 > 0** | | | | **% time LPRx_20 > 0.25** | | | |  |
|  |  | **Pre Dose** | **Post Dose** | **P value** | **Adj P Value** | **Pre Dose** | **Post Dose** | **P value** | **Adj P Value** | **Pre Dose** | **Post Dose** | **P value** | **Adj P Value** |  |
| Dobutamine | Decrease | -0.158 (-0.287--0.042) | -0.0339 (-0.188-0.118) | <0.0001 | <0.0001 | 24.1 (7.69-44.4) | 46.2 (20.7-69.2) | <0.0001 | <0.0001 | 0 (0-15.4) | 13.3 (0-37) | <0.0001 | <0.0001 |  |
| Dobutamine | Increase | -0.134 (-0.296--0.0372) | -0.0356 (-0.201-0.122) | <0.0001 | <0.0001 | 25 (7.69-47.6) | 45.1 (20-69.2) | <0.0001 | <0.0001 | 0 (0-15.4) | 13.7 (0-36) | <0.0001 | <0.0001 |  |
| Midazolam | Decrease | -0.128 (-0.254--0.0301) | -0.0186 (-0.154-0.106) | <0.0001 | <0.0001 | 28.6 (10.7-46.4) | 46.2 (23.1-69.1) | <0.0001 | <0.0001 | 0 (0-14.2) | 11.1 (0-32.1) | <0.0001 | <0.0001 |  |
| Midazolam | Increase | -0.129 (-0.254--0.0221) | -0.0207 (-0.16-0.113) | <0.0001 | <0.0001 | 27.8 (10-48) | 46.4 (22.6-69) | <0.0001 | <0.0001 | 0 (0-14.8) | 11.1 (0-33.3) | <0.0001 | <0.0001 |  |
| Morphine | Decrease | -0.121 (-0.239--0.0228) | -0.0122 (-0.143-0.126) | <0.0001 | <0.0001 | 30 (12.5-47.1) | 48.4 (26.7-70) | <0.0001 | <0.0001 | 0 (0-15.4) | 13.3 (0-35.3) | <0.0001 | <0.0001 |  |
| Morphine | Increase | -0.126 (-0.247--0.0241) | -0.00743 (-0.15-0.125) | <0.0001 | <0.0001 | 28.6 (11.1-47.8) | 48.3 (25.3-69.2) | <0.0001 | <0.0001 | 0 (0-14.5) | 13.3 (0-34.7) | <0.0001 | <0.0001 |  |
| Noradrenaline | Decrease | -0.134 (-0.253--0.0297) | -0.0227 (-0.164-0.112) | <0.0001 | <0.0001 | 27.6 (10.7-45.8) | 46.2 (22.9-68.4) | <0.0001 | <0.0001 | 0 (0-14.3) | 10.7 (0-33.3) | <0.0001 | <0.0001 |  |
| Noradrenaline | Increase | -0.131 (-0.25--0.0291) | -0.0262 (-0.172-0.106) | <0.0001 | <0.0001 | 28 (10.7-45.8) | 46.2 (22.2-67.9) | <0.0001 | <0.0001 | 0 (0-14.3) | 10.7 (0-32.1) | <0.0001 | <0.0001 |  |
| Propofol | Decrease | -0.124 (-0.246--0.0192) | -0.00224 (-0.145-0.137) | <0.0001 | <0.0001 | 30 (12-47.8) | 50 (26.7-72) | <0.0001 | <0.0001 | 0 (0-16.7) | 14.3 (0-37.9) | <0.0001 | <0.0001 |  |
| Propofol | Increase | -0.125 (-0.247--0.0229) | -0.00433 (-0.148-0.136) | <0.0001 | <0.0001 | 28.6 (11.5-46.4) | 48.6 (26.7-71.4) | <0.0001 | <0.0001 | 0 (0-15.4) | 14.3 (0-36.8) | <0.0001 | <0.0001 |  |
| Vasopressin | Increase | -0.154 (-0.262--0.026) | -0.0197 (-0.0938-0.047) | 0.0263 | 0.711 | 29.4 (15-51.9) | 38.7 (33.1-59.3) | 0.107 | 1 | 0 (0-16.2) | 3.61 (0-19.2) | 0.321 | 1 |  |
| Vasopressin | Decrease | -0.129 (-0.256-0.0226) | 0.122 (-0.0532-0.159) | 0.0562 | 1 | 26.8 (11.6-54) | 62 (42.5-73.1) | 0.0121 | 0.328 | 0 (0-26) | 31.6 (6-46.4) | 0.0351 | 0.948 |  |
| **Name** | **Mean Dose Change** | **% time LPRx_20 > 0.35** | | | | **Mean LPRx_30** | | | | **% time LPRx_30 > 0** | | | |  |
|  |  | **Pre Dose** | **Post Dose** | **P value** | **Adj P Value** | **Pre Dose** | **Post Dose** | **P value** | **Adj P Value** | **Pre Dose** | **Post Dose** | **P value** | **Adj P Value** |  |
| Dobutamine | Decrease | 0 (0-5.26) | 4 (0-23.7) | <0.0001 | <0.0001 | -0.123 (-0.267-0.00333) | -0.0411 (-0.223-0.12) | <0.0001 | <0.0001 | 25.9 (3.03-55.6) | 42.9 (13.3-73.3) | <0.0001 | <0.0001 |  |
| Dobutamine | Increase | 0 (0-6.88) | 3.51 (0-25) | <0.0001 | <0.0001 | -0.0992 (-0.278-0.0291) | -0.0444 (-0.209-0.127) | <0.0001 | <0.0001 | 29.7 (0-56.5) | 42 (11.1-73.9) | <0.0001 | <0.0001 |  |
| Midazolam | Decrease | 0 (0-4.22) | 0 (0-19.4) | <0.0001 | <0.0001 | -0.0913 (-0.225-0.0237) | -0.0196 (-0.159-0.107) | <0.0001 | <0.0001 | 30.8 (4.35-56.6) | 46.2 (16.7-74.1) | <0.0001 | <0.0001 |  |
| Midazolam | Increase | 0 (0-4.4) | 0 (0-20.7) | <0.0001 | <0.0001 | -0.0871 (-0.227-0.0306) | -0.0176 (-0.16-0.113) | <0.0001 | <0.0001 | 30.8 (5.07-57.7) | 45.8 (14.8-74.1) | <0.0001 | <0.0001 |  |
| Morphine | Decrease | 0 (0-6.98) | 3.57 (0-23.1) | <0.0001 | <0.0001 | -0.0802 (-0.218-0.0359) | -0.0109 (-0.142-0.128) | <0.0001 | <0.0001 | 34.6 (8.7-58.6) | 48.3 (20-75) | <0.0001 | <0.0001 |  |
| Morphine | Increase | 0 (0-4.76) | 3.57 (0-23.1) | <0.0001 | <0.0001 | -0.083 (-0.227-0.0354) | -0.0083 (-0.149-0.126) | <0.0001 | <0.0001 | 32.1 (7.14-58.6) | 48.1 (20-74.6) | <0.0001 | <0.0001 |  |
| Noradrenaline | Decrease | 0 (0-4.35) | 0 (0-20.7) | <0.0001 | <0.0001 | -0.0955 (-0.231-0.0219) | -0.0259 (-0.162-0.107) | <0.0001 | <0.0001 | 30.4 (6.9-56.5) | 44.4 (15.4-73.1) | <0.0001 | <0.0001 |  |
| Noradrenaline | Increase | 0 (0-4.17) | 0 (0-20.7) | <0.0001 | <0.0001 | -0.0907 (-0.226-0.0239) | -0.027 (-0.164-0.102) | <0.0001 | <0.0001 | 30.8 (6.45-57.1) | 44 (14.8-71.4) | <0.0001 | <0.0001 |  |
| Propofol | Decrease | 0 (0-7.41) | 3.85 (0-25) | <0.0001 | <0.0001 | -0.0816 (-0.222-0.038) | 0.0032 (-0.134-0.148) | <0.0001 | <0.0001 | 33.3 (9.09-59.6) | 51.6 (23.1-78.6) | <0.0001 | <0.0001 |  |
| Propofol | Increase | 0 (0-6.9) | 3.85 (0-25) | <0.0001 | <0.0001 | -0.0842 (-0.222-0.033) | 0.0054 (-0.132-0.145) | <0.0001 | <0.0001 | 32.1 (7.69-58.3) | 51.7 (23.1-77.8) | <0.0001 | <0.0001 |  |
| Vasopressin | Increase | 0 (0-11.1) | 1.61 (0-19.1) | 0.452 | 1 | -0.077 (-0.2--0.00428) | -0.0547 (-0.109-0.033) | 0.355 | 1 | 32.8 (16.5-65.1) | 26.7 (22.8-41.1) | 0.914 | 1 |  |
| Vasopressin | Decrease | 0 (0-15.9) | 18.2 (0-30.9) | 0.107 | 1 | -0.166 (-0.209--0.00104) | 0.083 (-0.0674-0.198) | 0.0468 | 1 | 28.6 (6.61-43.8) | 60 (38.5-78.1) | 0.0236 | 0.636 |  |
| **Name** | **Mean Dose Change** | **% time LPRx_30 > 0.25** | | | | **% time LPRx_30 > 0.35** | | | | **Mean LPRx_60** | | | |  |
|  |  | **Pre Dose** | **Post Dose** | **P value** | **Adj P Value** | **Pre Dose** | **Post Dose** | **P value** | **Adj P Value** | **Pre Dose** | **Post Dose** | **P value** | **Adj P Value** |  |
| Dobutamine | Decrease | 0 (0-17.2) | 4.26 (0-31) | <0.0001 | <0.0001 | 0 (0-4) | 0 (0-16) | <0.0001 | <0.0001 | -0.0471 (-0.235-0.117) | -0.0472 (-0.255-0.116) | 0.444 | 1 |  |
| Dobutamine | Increase | 0 (0-18.9) | 3.51 (0-33.3) | <0.0001 | 0.0013 | 0 (0-3.67) | 0 (0-20) | <0.0001 | 0.000107 | -0.0348 (-0.229-0.171) | -0.0569 (-0.216-0.106) | 0.118 | 1 |  |
| Midazolam | Decrease | 0 (0-12.5) | 0 (0-27.7) | <0.0001 | <0.0001 | 0 (0-0) | 0 (0-12.5) | <0.0001 | <0.0001 | -0.0285 (-0.159-0.117) | -0.0121 (-0.152-0.121) | 0.0922 | 1 |  |
| Midazolam | Increase | 0 (0-14.3) | 0 (0-28) | <0.0001 | <0.0001 | 0 (0-2.23) | 0 (0-11.5) | <0.0001 | <0.0001 | -0.0297 (-0.166-0.119) | -0.0189 (-0.156-0.121) | 0.248 | 1 |  |
| Morphine | Decrease | 0 (0-17.2) | 3.85 (0-33.3) | <0.0001 | <0.0001 | 0 (0-3.7) | 0 (0-16.7) | <0.0001 | <0.0001 | -0.015 (-0.171-0.143) | -0.00556 (-0.153-0.142) | 0.158 | 1 |  |
| Morphine | Increase | 0 (0-16.7) | 3.7 (0-33.3) | <0.0001 | <0.0001 | 0 (0-3.45) | 0 (0-17.4) | <0.0001 | <0.0001 | -0.0183 (-0.173-0.136) | -0.00782 (-0.158-0.14) | 0.221 | 1 |  |
| Noradrenaline | Decrease | 0 (0-14.3) | 0 (0-28.3) | <0.0001 | <0.0001 | 0 (0-2.56) | 0 (0-12.9) | <0.0001 | <0.0001 | -0.0256 (-0.176-0.115) | -0.0285 (-0.173-0.114) | 0.635 | 1 |  |
| Noradrenaline | Increase | 0 (0-14.3) | 0 (0-27.6) | <0.0001 | <0.0001 | 0 (0-3.33) | 0 (0-12) | <0.0001 | <0.0001 | -0.0275 (-0.173-0.116) | -0.0232 (-0.168-0.113) | 0.459 | 1 |  |
| Propofol | Decrease | 0 (0-18.2) | 6.67 (0-36.4) | <0.0001 | <0.0001 | 0 (0-4.55) | 0 (0-20) | <0.0001 | <0.0001 | -0.0133 (-0.167-0.139) | 0.00346 (-0.143-0.158) | <0.0001 | <0.0001 |  |
| Propofol | Increase | 0 (0-16) | 6.9 (0-36) | <0.0001 | <0.0001 | 0 (0-3.85) | 0 (0-20.7) | <0.0001 | <0.0001 | -0.016 (-0.164-0.13) | 0.00508 (-0.141-0.159) | <0.0001 | <0.0001 |  |
| Vasopressin | Increase | 0 (0-8.42) | 1.61 (0-9.68) | 0.689 | 1 | 0 (0-0) | 0 (0-3.23) | 0.725 | 1 | -0.0641 (-0.118--0.0157) | -0.0846 (-0.127-0.00584) | 0.883 | 1 |  |
| Vasopressin | Decrease | 0 (0-20.2) | 21.6 (3-52.8) | 0.0727 | 1 | 0 (0-20.2) | 9.94 (0-22) | 0.369 | 1 | -0.0996 (-0.169--0.00433) | 0.0138 (-0.121-0.222) | 0.119 | 1 |  |
| **Name** | **Mean Dose Change** | **% time LPRx_60 > 0** | | | | **% time LPRx_60 > 0.25** | | | | **% time LPRx_60 > 0.35** | | | |  |
|  |  | **Pre Dose** | **Post Dose** | **P value** | **Adj P Value** | **Pre Dose** | **Post Dose** | **P value** | **Adj P Value** | **Pre Dose** | **Post Dose** | **P value** | **Adj P Value** |  |
| Dobutamine | Decrease | 37 (0-83.3) | 34.6 (0-87.5) | 0.52 | 1 | 0 (0-25.9) | 0 (0-15.4) | 0.0401 | 1 | 0 (0-3.57) | 0 (0-0) | 0.164 | 1 |  |
| Dobutamine | Increase | 38.5 (0-91.9) | 28.6 (0-83.3) | 0.0343 | 0.925 | 0 (0-31.4) | 0 (0-14.3) | 0.0134 | 0.362 | 0 (0-6.61) | 0 (0-0) | 0.102 | 1 |  |
| Midazolam | Decrease | 41.4 (0-88) | 46.2 (0-89.3) | 0.125 | 1 | 0 (0-20) | 0 (0-19.2) | 0.704 | 1 | 0 (0-0) | 0 (0-0) | 0.376 | 1 |  |
| Midazolam | Increase | 39.1 (0-88) | 43.9 (0-89.7) | 0.316 | 1 | 0 (0-20) | 0 (0-19.2) | 0.344 | 1 | 0 (0-0) | 0 (0-0) | 0.326 | 1 |  |
| Morphine | Decrease | 46.2 (0-92) | 48 (3.33-92.6) | 0.336 | 1 | 0 (0-25.9) | 0 (0-25) | 0.3 | 1 | 0 (0-7.14) | 0 (0-3.85) | 0.186 | 1 |  |
| Morphine | Increase | 44.9 (0-90) | 46.2 (0-92.3) | 0.53 | 1 | 0 (0-24) | 0 (0-24) | 0.586 | 1 | 0 (0-3.57) | 0 (0-0) | 0.28 | 1 |  |
| Noradrenaline | Decrease | 40.7 (0-86.2) | 39.3 (0-88.9) | 0.0284 | 0.767 | 0 (0-19.2) | 0 (0-15.4) | <0.0001 | <0.0001 | 0 (0-0) | 0 (0-0) | <0.0001 | <0.0001 |  |
| Noradrenaline | Increase | 40 (0-87.5) | 40 (0-89.3) | 0.319 | 1 | 0 (0-18.2) | 0 (0-13.8) | <0.0001 | <0.0001 | 0 (0-0) | 0 (0-0) | <0.0001 | <0.0001 |  |
| Propofol | Decrease | 46.2 (3.45-92) | 51.7 (3.85-96.6) | <0.0001 | 0.00074 | 0 (0-25.9) | 0 (0-30.4) | 0.277 | 1 | 0 (0-3.85) | 0 (0-6.67) | 0.175 | 1 |  |
| Propofol | Increase | 44.4 (3.33-92) | 51.9 (3.7-100) | <0.0001 | <0.0001 | 0 (0-24.1) | 0 (0-29.2) | 0.11 | 1 | 0 (0-3.45) | 0 (0-3.7) | 0.173 | 1 |  |
| Vasopressin | Increase | 3.33 (0-26.7) | 3.28 (0-48.1) | 0.933 | 1 | 0 (0-0) | 0 (0-0) | 0.985 | 1 | 0 (0-0) | 0 (0-0) | 0.698 | 1 |  |
| Vasopressin | Decrease | 10 (0-45.9) | 65.5 (15.2-100) | 0.0662 | 1 | 0 (0-0) | 0 (0-24.8) | 0.0828 | 1 | 0 (0-0) | 0 (0-24.8) | 0.144 | 1 |  |
| **Bolus** | | | | | | | | | | | | | |  |
| **Name** | **Doses** | **Mean MAP** | | | | **Mean CPP** | | | | **% time CPP>60** | | | |  |
|  |  | **Pre Dose** | **Post Dose** | **P value** | **Adj P Value** | **Pre Dose** | **Post Dose** | **P value** | **Adj P Value** | **Pre Dose** | **Post Dose** | **P value** | **Adj P Value** |  |
| Ephedrine | 14 | 70.6 (63-75.4) | 76.6 (64-83.3) | 0.541 | 1 | 57.2 (56.5-64.1) | 60.4 (51.9-75.3) | 0.839 | 1 | 80.7 (34.7-98.9) | 84.4 (4.21-100) | 0.657 | 1 |  |
| Fentanyl | 95 | 82.1 (75.7-89.5) | 80.3 (73.7-88.1) | 0.614 | 1 | 69.4 (63.2-81.2) | 68.8 (62.1-79.2) | 0.488 | 1 | 4.35 (0-24) | 4 (0-35.2) | 0.785 | 1 |  |
| Midazolam | 529 | 79.6 (75.3-85.4) | 79.4 (74.7-85.4) | 0.544 | 1 | 64 (59.2-69.6) | 64.1 (58.8-70.1) | 0.816 | 1 | 16 (0-61.5) | 14.8 (0-66.7) | 0.767 | 1 |  |
| Morphine | 1996 | 80.9 (75-89.4) | 79.9 (73.9-87.8) | <0.0001 | 0.00149 | 68 (62.2-77.3) | 66.8 (60.7-74.9) | <0.0001 | 0.000263 | 3.57 (0-33.3) | 6.9 (0-46.9) | 0.000634 | 0.0171 |  |
| Propofol | 2186 | 79.7 (74.6-85.5) | 79 (73.5-85.2) | 0.00646 | 0.174 | 65.7 (60.1-71.8) | 64.9 (59.2-71.1) | 0.00836 | 0.226 | 11.1 (0-51.8) | 13.8 (0-61.3) | 0.0527 | 1 |  |
| Alfentanil | 192 | 78 (72-85.4) | 77.4 (71.6-84.3) | 0.607 | 1 | 66.2 (59.8-73) | 65.7 (60.6-75.2) | 0.603 | 1 | 13.2 (0-51.8) | 12.1 (0-55.3) | 0.445 | 1 |  |
| **Name** | **% time CPP>70** | | | | **Mean ICP** | | | | **% time ICP>20** | | | |  |  |
|  | **Pre Dose** | **Post Dose** | **P value** | **Adj P Value** | **Pre Dose** | **Post Dose** | **P value** | **Adj P Value** | **Pre Dose** | **Post Dose** | **P value** | **Adj P Value** |  |  |
| Ephedrine | 0 (0-3.33) | 0 (0-74.1) | 0.296 | 1 | 11.6 (5.27-14.2) | 10.6 (6.77-16) | 0.454 | 1 | 0 (0-0) | 0 (0-4.3) | 0.305 | 1 |  |  |
| Fentanyl | 50 (10.7-98.1) | 41.4 (5.49-96.2) | 0.519 | 1 | 10.2 (6.92-14) | 11.2 (6.92-13.7) | 0.62 | 1 | 0 (0-3.77) | 0 (0-3.7) | 0.925 | 1 |  |  |
| Midazolam | 6.98 (0-43.5) | 4.17 (0-45.8) | 0.388 | 1 | 16.5 (12.2-19.1) | 15.9 (12-19.2) | 0.526 | 1 | 4 (0-25) | 3.57 (0-28) | 0.463 | 1 |  |  |
| Morphine | 29.6 (2.12-92.3) | 20 (0-82.1) | <0.0001 | 0.00241 | 12.5 (8.78-16.7) | 12.7 (9.04-16.8) | 0.276 | 1 | 0 (0-8) | 0 (0-8.01) | 0.797 | 1 |  |  |
| Propofol | 12 (0-60.7) | 7.41 (0-58.5) | 0.00021 | 0.00567 | 14.2 (10.1-18.2) | 14.1 (9.93-18.1) | 0.798 | 1 | 0 (0-14.8) | 0 (0-14.3) | 0.0508 | 1 |  |  |
| Alfentanil | 17.5 (3.11-67) | 16.8 (0-84.8) | 0.903 | 1 | 11.8 (7.81-15) | 10.8 (6.88-14) | 0.0736 | 1 | 0 (0-7.14) | 0 (0-0.0644) | 0.00238 | 0.0642 |  |  |
| **Name** | **% time ICP>22** | | | | **Mean LPRx_10** | | | | **% time LPRx_10 > 0** | | | |  |  |
|  | **Pre Dose** | **Post Dose** | **P value** | **Adj P Value** | **Pre Dose** | **Post Dose** | **P value** | **Adj P Value** | **Pre Dose** | **Post Dose** | **P value** | **Adj P Value** |  |  |
| Ephedrine | 0 (0-0) | 0 (0-0) | 1 | 1 | -0.131 (-0.217--0.0947) | 0.0287 (-0.324-0.145) | 0.15 | 1 | 35 (23.6-39.1) | 48.7 (29.5-70.2) | 0.0694 | 1 |  |  |
| Fentanyl | 0 (0-3.19) | 0 (0-0) | 0.764 | 1 | -0.133 (-0.242--0.0309) | -0.057 (-0.19-0.144) | 0.000205 | 0.00554 | 32 (21-41.7) | 49.1 (29.7-71.4) | <0.0001 | <0.0001 |  |  |
| Midazolam | 0 (0-7.69) | 0 (0-8) | 0.851 | 1 | -0.143 (-0.257--0.0634) | 0.00247 (-0.138-0.14) | <0.0001 | <0.0001 | 32 (19.2-41.4) | 49.5 (33.3-66.7) | <0.0001 | <0.0001 |  |  |
| Morphine | 0 (0-2.08) | 0 (0-2.38) | 0.49 | 1 | -0.129 (-0.231--0.0478) | 0.00171 (-0.13-0.141) | <0.0001 | <0.0001 | 33.3 (22.2-41.9) | 50 (34.5-66.7) | <0.0001 | <0.0001 |  |  |
| Propofol | 0 (0-5.1) | 0 (0-3.96) | 0.015 | 0.405 | -0.14 (-0.255--0.0579) | -0.00109 (-0.15-0.139) | <0.0001 | <0.0001 | 32 (20-41.4) | 50 (32-67.6) | <0.0001 | <0.0001 |  |  |
| Alfentanil | 0 (0-3.57) | 0 (0-0) | 0.00721 | 0.195 | -0.136 (-0.25--0.0525) | -0.0151 (-0.165-0.124) | <0.0001 | <0.0001 | 32.1 (21.3-40.7) | 48.3 (28.4-64.3) | <0.0001 | <0.0001 |  |  |
| **Name** | **% time LPRx_10 > 0.25** | | | | **% time LPRx_10 > 0.35** | | | | **Mean LPRx_15** | | | |  |  |
|  | **Pre Dose** | **Post Dose** | **P value** | **Adj P Value** | **Pre Dose** | **Post Dose** | **P value** | **Adj P Value** | **Pre Dose** | **Post Dose** | **P value** | **Adj P Value** |  |  |
| Ephedrine | 5.37 (0-16.6) | 29.8 (19.9-42.6) | 0.0102 | 0.274 | 3.65 (0-8.65) | 21.5 (8.98-33.3) | 0.0189 | 0.51 | -0.16 (-0.303--0.111) | -0.00947 (-0.332-0.0918) | 0.114 | 1 |  |  |
| Fentanyl | 13.3 (4.3-23.1) | 23.1 (8.7-40.7) | 0.00055 | 0.0148 | 7.14 (0-17.9) | 11.5 (0-30) | 0.00193 | 0.0522 | -0.0951 (-0.25--0.0215) | -0.0688 (-0.203-0.161) | 0.00555 | 0.15 |  |  |
| Midazolam | 10 (3.45-20.8) | 25 (10.3-41.7) | <0.0001 | <0.0001 | 4 (0-13.8) | 16.7 (3.7-33.3) | <0.0001 | <0.0001 | -0.138 (-0.253--0.0508) | 0.00613 (-0.111-0.136) | <0.0001 | <0.0001 |  |  |
| Morphine | 11.2 (3.57-20.8) | 25.9 (10.7-40) | <0.0001 | <0.0001 | 4.55 (0-15) | 17.4 (3.85-31.7) | <0.0001 | <0.0001 | -0.119 (-0.23--0.0304) | 0.000323 (-0.132-0.133) | <0.0001 | <0.0001 |  |  |
| Propofol | 10.7 (0-20) | 24.1 (10-40.3) | <0.0001 | <0.0001 | 4.17 (0-14.3) | 15.4 (3.7-30.8) | <0.0001 | <0.0001 | -0.125 (-0.24--0.0417) | 0.00499 (-0.138-0.138) | <0.0001 | <0.0001 |  |  |
| Alfentanil | 11.1 (3.45-22.4) | 25 (8.52-38.5) | <0.0001 | <0.0001 | 5.51 (0-15.4) | 17.9 (4-29.6) | <0.0001 | <0.0001 | -0.127 (-0.212--0.0444) | -0.00792 (-0.135-0.12) | <0.0001 | <0.0001 |  |  |
| **Name** | **% time LPRx_15 > 0** | | | | **% time LPRx_15 > 0.25** | | | | **% time LPRx_15 > 0.35** | | | |  |  |
|  | **Pre Dose** | **Post Dose** | **P value** | **Adj P Value** | **Pre Dose** | **Post Dose** | **P value** | **Adj P Value** | **Pre Dose** | **Post Dose** | **P value** | **Adj P Value** |  |  |
| Ephedrine | 25.1 (8.55-30.7) | 38.4 (25.3-65.4) | 0.0534 | 1 | 1.79 (0-7.61) | 25.6 (19.6-36.6) | 0.0027 | 0.073 | 0 (0-7.61) | 18.5 (11.8-26.7) | 0.0112 | 0.303 |  |  |
| Fentanyl | 32.1 (19.2-44.4) | 42.9 (25.5-71.2) | <0.0001 | 0.00182 | 8 (0-20.7) | 16.7 (1.79-37.5) | 0.00109 | 0.0294 | 0 (0-14.3) | 10 (0-19.9) | 0.0472 | 1 |  |  |
| Midazolam | 29.2 (17.2-40.7) | 50.4 (32.3-70.5) | <0.0001 | <0.0001 | 4 (0-15.4) | 21.9 (3.85-38.1) | <0.0001 | <0.0001 | 0 (0-7.69) | 10.7 (0-28) | <0.0001 | <0.0001 |  |  |
| Morphine | 31 (17.9-44.4) | 50 (31.7-69) | <0.0001 | <0.0001 | 6.82 (0-19) | 20.8 (3.85-39.3) | <0.0001 | <0.0001 | 0 (0-10.7) | 11.5 (0-28) | <0.0001 | <0.0001 |  |  |
| Propofol | 30.8 (16.7-42.9) | 50 (30.4-69.2) | <0.0001 | <0.0001 | 4.17 (0-17.9) | 20 (3.7-39.3) | <0.0001 | <0.0001 | 0 (0-9.52) | 10.3 (0-27.1) | <0.0001 | <0.0001 |  |  |
| Alfentanil | 31.3 (16.7-43.5) | 48.3 (29.4-65.8) | <0.0001 | <0.0001 | 3.85 (0-18.3) | 19.7 (0-36) | <0.0001 | <0.0001 | 0 (0-8.79) | 10.3 (0-25) | <0.0001 | <0.0001 |  |  |
| **Name** | **Mean LPRx_20** | | | | **% time LPRx_20 > 0** | | | | **% time LPRx_20 > 0.25** | | | |  |  |
|  | **Pre Dose** | **Post Dose** | **P value** | **Adj P Value** | **Pre Dose** | **Post Dose** | **P value** | **Adj P Value** | **Pre Dose** | **Post Dose** | **P value** | **Adj P Value** |  |  |
| Ephedrine | -0.153 (-0.326--0.0988) | -0.0476 (-0.259-0.122) | 0.15 | 1 | 23.4 (4.93-33) | 42.4 (26.4-72.5) | 0.0274 | 0.74 | 0 (0-12.6) | 24.2 (10.9-29) | 0.00292 | 0.0789 |  |  |
| Fentanyl | -0.0769 (-0.218-0.000541) | -0.0686 (-0.178-0.145) | 0.0357 | 0.964 | 30 (14.8-49.9) | 45.8 (21.3-74.7) | 0.00463 | 0.125 | 7.69 (0-21.4) | 9.09 (0-31.5) | 0.11 | 1 |  |  |
| Midazolam | -0.125 (-0.245--0.0316) | 0.017 (-0.0942-0.141) | <0.0001 | <0.0001 | 29.2 (12.5-44.4) | 51.9 (32.1-71.1) | <0.0001 | <0.0001 | 0 (0-15.4) | 17.9 (0-39.3) | <0.0001 | <0.0001 |  |  |
| Morphine | -0.11 (-0.222--0.0028) | 0.000869 (-0.129-0.135) | <0.0001 | <0.0001 | 32 (14.8-49.7) | 50 (28.2-71.4) | <0.0001 | <0.0001 | 3.57 (0-18.5) | 17.9 (0-38.5) | <0.0001 | <0.0001 |  |  |
| Propofol | -0.112 (-0.225--0.0148) | 0.0115 (-0.117-0.146) | <0.0001 | <0.0001 | 30.6 (14.3-48.1) | 50.8 (28.6-72.4) | <0.0001 | <0.0001 | 0 (0-16.7) | 16 (0-38.9) | <0.0001 | <0.0001 |  |  |
| Alfentanil | -0.106 (-0.209--0.0168) | 0.00422 (-0.124-0.115) | <0.0001 | <0.0001 | 32.1 (14.3-47.8) | 51.7 (31.9-70) | <0.0001 | <0.0001 | 0 (0-17.9) | 15.8 (0-34.6) | <0.0001 | <0.0001 |  |  |
| **Name** | **% time LPRx_20 > 0.35** | | | | **Mean LPRx_30** | | | | **% time LPRx_30 > 0** | | | |  |  |
|  | **Pre Dose** | **Post Dose** | **P value** | **Adj P Value** | **Pre Dose** | **Post Dose** | **P value** | **Adj P Value** | **Pre Dose** | **Post Dose** | **P value** | **Adj P Value** |  |  |
| Ephedrine | 0 (0-7.45) | 12.8 (0-16.4) | 0.077 | 1 | -0.157 (-0.348--0.049) | -0.0451 (-0.157--0.00407) | 0.114 | 1 | 22 (5.71-36.5) | 56 (25.4-63.6) | 0.00578 | 0.156 |  |  |
| Fentanyl | 0 (0-12.8) | 0 (0-24.5) | 0.115 | 1 | -0.039 (-0.186-0.0493) | -0.032 (-0.121-0.0918) | 0.275 | 1 | 36.2 (11.5-62.4) | 46.4 (25-69.8) | 0.0604 | 1 |  |  |
| Midazolam | 0 (0-4.17) | 6.9 (0-26.2) | <0.0001 | <0.0001 | -0.0993 (-0.224-0.0189) | 0.0146 (-0.098-0.158) | <0.0001 | <0.0001 | 33.3 (10.7-56.7) | 51.7 (25.8-80) | <0.0001 | <0.0001 |  |  |
| Morphine | 0 (0-8) | 7.14 (0-25) | <0.0001 | <0.0001 | -0.0781 (-0.219-0.0357) | 0.00674 (-0.13-0.147) | <0.0001 | <0.0001 | 34.8 (8.7-59.3) | 51.7 (23.6-78.4) | <0.0001 | <0.0001 |  |  |
| Propofol | 0 (0-7.41) | 4.94 (0-25) | <0.0001 | <0.0001 | -0.075 (-0.204-0.0355) | 0.0191 (-0.11-0.156) | <0.0001 | <0.0001 | 33.3 (10.7-59.3) | 53.6 (25-81.2) | <0.0001 | <0.0001 |  |  |
| Alfentanil | 0 (0-6.9) | 6.67 (0-21.7) | <0.0001 | <0.0001 | -0.0587 (-0.172-0.0539) | -0.00291 (-0.11-0.135) | <0.0001 | 0.000841 | 32.1 (12.4-62.3) | 53.5 (25-80) | <0.0001 | 0.000813 |  |  |
| **Name** | **% time LPRx_30 > 0.25** | | | | **% time LPRx_30 > 0.35** | | | | **Mean LPRx_60** | | | |  |  |
|  | **Pre Dose** | **Post Dose** | **P value** | **Adj P Value** | **Pre Dose** | **Post Dose** | **P value** | **Adj P Value** | **Pre Dose** | **Post Dose** | **P value** | **Adj P Value** |  |  |
| Ephedrine | 3.87 (0-15.9) | 1.14 (0-22.9) | 0.981 | 1 | 0 (0-3.13) | 0 (0-13) | 0.931 | 1 | -0.141 (-0.27-0.292) | -0.0262 (-0.263-0.042) | 0.734 | 1 |  |  |
| Fentanyl | 0 (0-29.2) | 8.33 (0-26.9) | 0.335 | 1 | 0 (0-12) | 0 (0-13) | 0.359 | 1 | 0.0248 (-0.125-0.242) | -0.0569 (-0.199-0.229) | 0.365 | 1 |  |  |
| Midazolam | 0 (0-17.2) | 10.7 (0-38.5) | <0.0001 | <0.0001 | 0 (0-3.7) | 0 (0-23.3) | <0.0001 | <0.0001 | -0.0198 (-0.196-0.165) | 0.0158 (-0.128-0.184) | 0.00801 | 0.216 |  |  |
| Morphine | 0 (0-19.6) | 8.38 (0-37) | <0.0001 | <0.0001 | 0 (0-8) | 0 (0-21.4) | <0.0001 | <0.0001 | -0.0173 (-0.172-0.144) | 0.00357 (-0.179-0.165) | 0.0403 | 1 |  |  |
| Propofol | 0 (0-18.5) | 7.41 (0-38.5) | <0.0001 | <0.0001 | 0 (0-4) | 0 (0-21.4) | <0.0001 | <0.0001 | -0.00894 (-0.157-0.139) | 0.0197 (-0.121-0.18) | <0.0001 | <0.0001 |  |  |
| Alfentanil | 0 (0-20.9) | 9.17 (0-33.3) | 0.0182 | 0.49 | 0 (0-8.87) | 0 (0-16.7) | 0.0243 | 0.657 | 0.0167 (-0.153-0.129) | 0.0415 (-0.0936-0.174) | 0.0589 | 1 |  |  |
| **Name** | **% time LPRx_60 > 0** | | | | **% time LPRx_60 > 0.25** | | | | **% time LPRx_60 > 0.35** | | | |  |  |
|  | **Pre Dose** | **Post Dose** | **P value** | **Adj P Value** | **Pre Dose** | **Post Dose** | **P value** | **Adj P Value** | **Pre Dose** | **Post Dose** | **P value** | **Adj P Value** |  |  |
| Ephedrine | 17.4 (0.758-99.1) | 36.9 (0-56) | 0.673 | 1 | 1.52 (0-66.5) | 0 (0-0) | 0.0909 | 1 | 0 (0-45.3) | 0 (0-0) | 0.263 | 1 |  |  |
| Fentanyl | 56.5 (0-100) | 40 (0-100) | 0.449 | 1 | 0 (0-56) | 0 (0-26.9) | 0.204 | 1 | 0 (0-14.3) | 0 (0-12.7) | 0.47 | 1 |  |  |
| Midazolam | 44 (0-88.9) | 50 (2.08-100) | 0.0694 | 1 | 0 (0-32.3) | 0 (0-34.5) | 0.438 | 1 | 0 (0-12) | 0 (0-8.33) | 0.842 | 1 |  |  |
| Morphine | 44.6 (2.22-85.8) | 49.9 (0-96.6) | 0.00798 | 0.215 | 0 (0-28.6) | 0 (0-30.7) | 0.735 | 1 | 0 (0-12.5) | 0 (0-12.5) | 0.825 | 1 |  |  |
| Propofol | 45.3 (3.7-89.3) | 55.2 (4.17-100) | <0.0001 | <0.0001 | 0 (0-27.6) | 0 (0-32.3) | 0.237 | 1 | 0 (0-7.41) | 0 (0-4.44) | 0.913 | 1 |  |  |
| Alfentanil | 50 (11.3-91.8) | 61.2 (16.5-100) | 0.11 | 1 | 0 (0-30.2) | 0 (0-32.9) | 0.986 | 1 | 0 (0-14.7) | 0 (0-4.04) | 0.28 | 1 |  |  |

*The table demonstrates the median and interquartile range of the pre/pose dose windows as well as the Wilcox signed ranked test between these windows with p values adjusted using the Bonferroni analysis. The “change” indicates if the continuous infusion was increase/decrease or was a bolus dose. CPP, cerebral prefusion pressure; ICP, intracranial pressure; LPRx_10, pressure reactivity over 10 minutes; LPRx_15, pressure reactivity over 15 minutes; LPRx_20, pressure reactivity over 20 minutes; LPRx_30, pressure reactivity over 30 minutes; LPRx_60, pressure reactivity over 60 minutes;*

# Appendix K. Pre-time window over 50% time L-PRx_10 > 0.35

The table contains all infusions given with the LPRx_10 pre-time window 50% > 0.35, separated into the continuous infusion doses then the bolus doses.

| **Continuous Intravenous** | | | | | | | | | | | | | | |
| --- | --- | --- | --- | --- | --- | --- | --- | --- | --- | --- | --- | --- | --- | --- |
| **Name** | **Doses** | **Mean Dose Change** | **Mean MAP** | | | | **Mean CPP** | | | | **% time CPP>60** | | | |
|  |  |  | **Pre Dose** | **Post Dose** | **P value** | **Adj P Value** | **Pre Dose** | **Post Dose** | **P value** | **Adj P Value** | **Pre Dose** | **Post Dose** | **P value** | **Adj P Value** |
| Dobutamine | 134 | Decrease | 76.4 (69.9-81.9) | 75.2 (70.1-82) | 0.687 | 1 | 63.1 (58.6-68.8) | 62.5 (57.5-70) | 0.815 | 1 | 14 (0-76.4) | 18 (0-82.1) | 0.409 | 1 |
| Dobutamine | 99 | Increase | 72.7 (67.9-76.8) | 74.2 (67.4-79.8) | 0.467 | 1 | 59 (55.8-64.1) | 61 (56.3-65.6) | 0.181 | 1 | 69.8 (20.8-96.4) | 35.6 (4.71-93.2) | 0.184 | 1 |
| Midazolam | 557 | Increase | 78.6 (73.7-85.5) | 78.2 (73.1-85.4) | 0.341 | 1 | 64.6 (59-71.2) | 64.5 (59.2-71) | 0.959 | 1 | 7.14 (0-66.7) | 8 (0-60) | 0.98 | 1 |
| Midazolam | 529 | Decrease | 78.2 (72.7-85) | 77.9 (72.4-84.5) | 0.636 | 1 | 64.5 (59.3-71.7) | 65 (59.3-71.2) | 0.817 | 1 | 4.35 (0-66.7) | 7.69 (0-60.7) | 0.504 | 1 |
| Morphine | 808 | Increase | 79 (72.5-86.7) | 78.1 (71.8-84.5) | 0.0117 | 0.317 | 65.6 (59.5-73) | 65.2 (59-71.4) | 0.175 | 1 | 6.67 (0-61.6) | 9.23 (0-65.5) | 0.169 | 1 |
| Morphine | 747 | Decrease | 77.5 (71.7-84.8) | 77.1 (71.4-84.5) | 0.385 | 1 | 64.9 (59.4-72.2) | 64.9 (59.2-71.6) | 0.819 | 1 | 4.17 (0-65.4) | 10.3 (0-66.7) | 0.219 | 1 |
| Noradrenaline | 5189 | Decrease | 78.9 (73.5-85.8) | 78.6 (73.2-84.9) | 0.00451 | 0.122 | 66.2 (60.1-72.9) | 65.6 (60.1-72) | 0.0122 | 0.33 | 3.57 (0-51.9) | 6.9 (0-52) | 0.00108 | 0.029 |
| Noradrenaline | 4975 | Increase | 75.8 (70.2-82.1) | 76.2 (70.6-82.2) | 0.153 | 1 | 62.5 (56.8-69) | 63.5 (57.9-69.4) | <0.0001 | <0.0001 | 27.5 (0-88.9) | 21.4 (0-80.8) | <0.0001 | <0.0001 |
| Propofol | 1614 | Decrease | 77.5 (71.4-84.5) | 77.2 (71.1-83.3) | 0.113 | 1 | 64.9 (59-71.9) | 64.4 (58.6-70.8) | 0.14 | 1 | 8.17 (0-68) | 12.9 (0-69.2) | 0.0661 | 1 |
| Propofol | 1536 | Increase | 78.5 (72.6-86) | 77.8 (71.8-84.3) | 0.00426 | 0.115 | 65.6 (59.4-72.3) | 65.1 (59-71.1) | 0.07 | 1 | 7.36 (0-63) | 10.3 (0-64.3) | 0.0859 | 1 |
| Vasopressin | 11 | Decrease | 76.2 (73.9-79.8) | 71.1 (68.3-74.2) | 0.0128 | 0.347 | 62.6 (61.5-64.5) | 55.9 (52.9-62.1) | 0.0233 | 0.629 | 41.7 (8.06-57.7) | 93.8 (25.4-100) | 0.11 | 1 |
| **Name** | **Mean Dose Change** | **% time CPP>70** | | | | **Mean ICP** | | | | **% time ICP>20** | | | |  |
|  |  | **Pre Dose** | **Post Dose** | **P value** | **Adj P Value** | **Pre Dose** | **Post Dose** | **P value** | **Adj P Value** | **Pre Dose** | **Post Dose** | **P value** | **Adj P Value** |  |
| Dobutamine | Decrease | 0 (0-35.4) | 3.34 (0-34.4) | 0.756 | 1 | 13.7 (9.86-16.5) | 13.2 (10.6-16.4) | 0.625 | 1 | 0 (0-6.4) | 0 (0-3.28) | 0.331 | 1 |  |
| Dobutamine | Increase | 0 (0-7.33) | 0 (0-17.4) | 0.318 | 1 | 14 (10.8-17.5) | 13.4 (10.6-17.3) | 0.597 | 1 | 0 (0-18.1) | 0 (0-7.14) | 0.508 | 1 |  |
| Midazolam | Increase | 3.57 (0-60.5) | 4.17 (0-54.2) | 0.518 | 1 | 14.5 (10.9-17.8) | 13.9 (10.6-17.5) | 0.138 | 1 | 0 (0-11.1) | 0 (0-3.85) | 0.0196 | 0.53 |  |
| Midazolam | Decrease | 3.7 (0-64.3) | 4.35 (0-55.6) | 0.483 | 1 | 13.3 (10.1-16.8) | 13.2 (10.1-16.3) | 0.441 | 1 | 0 (0-3.7) | 0 (0-0) | 0.265 | 1 |  |
| Morphine | Increase | 9.52 (0-73.5) | 6.9 (0-60.8) | 0.468 | 1 | 13.2 (9.44-16.6) | 12.7 (9.04-16.5) | 0.148 | 1 | 0 (0-6.56) | 0 (0-3.15) | 0.00809 | 0.218 |  |
| Morphine | Decrease | 4.17 (0-75.3) | 6.67 (0-61) | 0.648 | 1 | 12.4 (8.9-16.1) | 12.4 (8.79-15.8) | 0.602 | 1 | 0 (0-3.33) | 0 (0-0) | 0.489 | 1 |  |
| Noradrenaline | Decrease | 11.5 (0-77.8) | 8 (0-65.6) | 0.000281 | 0.00759 | 12.9 (9.3-16.4) | 13.1 (9.61-16.3) | 0.565 | 1 | 0 (0-3.7) | 0 (0-3.23) | 0.00248 | 0.0668 |  |
| Noradrenaline | Increase | 3.33 (0-37) | 3.57 (0-37.2) | 0.034 | 0.917 | 13.5 (9.7-16.8) | 12.9 (9.17-16.3) | <0.0001 | <0.0001 | 0 (0-6.67) | 0 (0-0) | <0.0001 | <0.0001 |  |
| Propofol | Decrease | 6.67 (0-66.7) | 4.94 (0-52) | 0.521 | 1 | 12.8 (8.59-16.2) | 12.6 (8.29-16.2) | 0.578 | 1 | 0 (0-3.57) | 0 (0-3.57) | 0.913 | 1 |  |
| Propofol | Increase | 8.45 (0-71.4) | 7.14 (0-57.7) | 0.125 | 1 | 13.1 (9.18-16.7) | 12.8 (8.65-16.4) | 0.104 | 1 | 0 (0-6.9) | 0 (0-3.57) | 0.00177 | 0.0478 |  |
| Vasopressin | Decrease | 3.23 (0-18.8) | 0 (0-0) | 0.00608 | 0.164 | 14.8 (12.2-16.5) | 13.9 (13.1-14.5) | 0.478 | 1 | 0 (0-0) | 0 (0-0) | 0.167 | 1 |  |
| **Name** | **Mean Dose Change** | **% time ICP>22** | | | | **Mean LPRx_10** | | | | **% time LPRx_10 > 0** | | | |  |
|  |  | **Pre Dose** | **Post Dose** | **P value** | **Adj P Value** | **Pre Dose** | **Post Dose** | **P value** | **Adj P Value** | **Pre Dose** | **Post Dose** | **P value** | **Adj P Value** |  |
| Dobutamine | Decrease | 0 (0-0) | 0 (0-0) | 0.378 | 1 | 0.381 (0.328-0.444) | 0.0374 (-0.0948-0.149) | <0.0001 | <0.0001 | 86.2 (75.2-94.9) | 53.2 (36.4-71.2) | <0.0001 | <0.0001 |  |
| Dobutamine | Increase | 0 (0-1.16) | 0 (0-0) | 0.795 | 1 | 0.419 (0.338-0.489) | 0.0994 (-0.0554-0.241) | <0.0001 | <0.0001 | 89.7 (78-96.4) | 59.3 (36-78) | <0.0001 | <0.0001 |  |
| Midazolam | Increase | 0 (0-0) | 0 (0-0) | 0.0199 | 0.538 | 0.392 (0.325-0.492) | 0.0202 (-0.164-0.22) | <0.0001 | <0.0001 | 85.7 (76.7-96.2) | 52 (33.3-72) | <0.0001 | <0.0001 |  |
| Midazolam | Decrease | 0 (0-0) | 0 (0-0) | 0.261 | 1 | 0.397 (0.329-0.49) | 0.0371 (-0.13-0.209) | <0.0001 | <0.0001 | 85.4 (75.9-96.2) | 55.6 (36-73.1) | <0.0001 | <0.0001 |  |
| Morphine | Increase | 0 (0-0) | 0 (0-0) | 0.00112 | 0.0302 | 0.398 (0.327-0.5) | 0.0126 (-0.153-0.212) | <0.0001 | <0.0001 | 87.5 (76.9-97.2) | 51.7 (33.3-72) | <0.0001 | <0.0001 |  |
| Morphine | Decrease | 0 (0-0) | 0 (0-0) | 0.413 | 1 | 0.391 (0.332-0.486) | 0.0318 (-0.119-0.207) | <0.0001 | <0.0001 | 86.2 (76-96.2) | 53.8 (36.7-73.6) | <0.0001 | <0.0001 |  |
| Noradrenaline | Decrease | 0 (0-0) | 0 (0-0) | <0.0001 | 0.0016 | 0.395 (0.322-0.481) | 0.0406 (-0.13-0.216) | <0.0001 | <0.0001 | 85.2 (75.9-95.5) | 53.8 (34.5-73.1) | <0.0001 | <0.0001 |  |
| Noradrenaline | Increase | 0 (0-0) | 0 (0-0) | <0.0001 | <0.0001 | 0.397 (0.324-0.482) | 0.0372 (-0.138-0.21) | <0.0001 | <0.0001 | 85.7 (76-95.7) | 53.6 (34.1-73.1) | <0.0001 | <0.0001 |  |
| Propofol | Decrease | 0 (0-0) | 0 (0-0) | 0.805 | 1 | 0.394 (0.317-0.486) | 0.0378 (-0.113-0.213) | <0.0001 | <0.0001 | 85.7 (75.9-96) | 53.8 (37.5-73.1) | <0.0001 | <0.0001 |  |
| Propofol | Increase | 0 (0-0) | 0 (0-0) | 0.00542 | 0.146 | 0.4 (0.323-0.489) | 0.0508 (-0.119-0.216) | <0.0001 | <0.0001 | 85.7 (76.2-96.3) | 55.2 (37-72.4) | <0.0001 | <0.0001 |  |
| Vasopressin | Decrease | 0 (0-0) | 0 (0-0) | 0.167 | 1 | 0.442 (0.327-0.503) | 0.0299 (-0.173-0.0811) | <0.0001 | 0.000536 | 87.5 (77-98.4) | 53.8 (33.3-61.1) | 0.000492 | 0.0133 |  |
| **Name** | **Mean Dose Change** | **% time LPRx_10 > 0.25** | | | | **% time LPRx_10 > 0.35** | | | | **Mean LPRx_15** | | | |  |
|  |  | **Pre Dose** | **Post Dose** | **P value** | **Adj P Value** | **Pre Dose** | **Post Dose** | **P value** | **Adj P Value** | **Pre Dose** | **Post Dose** | **P value** | **Adj P Value** |  |
| Dobutamine | Decrease | 70.6 (64.1-80) | 27.9 (10.1-46.6) | <0.0001 | <0.0001 | 60.7 (56-71.3) | 20.1 (4.57-34.5) | <0.0001 | <0.0001 | 0.369 (0.292-0.453) | 0.0193 (-0.106-0.17) | <0.0001 | <0.0001 |  |
| Dobutamine | Increase | 75.9 (66.7-87.9) | 32 (15.1-51.9) | <0.0001 | <0.0001 | 65.4 (57.4-77.6) | 25.8 (7.69-41.1) | <0.0001 | <0.0001 | 0.389 (0.295-0.498) | 0.0694 (-0.117-0.199) | <0.0001 | <0.0001 |  |
| Midazolam | Increase | 71.4 (63-83.3) | 29.6 (10-48.3) | <0.0001 | <0.0001 | 64 (56-75) | 20.7 (3.85-39.3) | <0.0001 | <0.0001 | 0.374 (0.287-0.475) | 0.0254 (-0.143-0.196) | <0.0001 | <0.0001 |  |
| Midazolam | Decrease | 70 (62.5-79.3) | 30.4 (11.5-50) | <0.0001 | <0.0001 | 62.2 (55.6-72) | 20.8 (4-40.7) | <0.0001 | <0.0001 | 0.363 (0.285-0.462) | 0.0341 (-0.124-0.185) | <0.0001 | <0.0001 |  |
| Morphine | Increase | 72.7 (64.3-84.6) | 27.6 (8.25-50) | <0.0001 | <0.0001 | 65 (57.1-75.9) | 18.5 (3.67-38.5) | <0.0001 | <0.0001 | 0.376 (0.279-0.478) | 0.026 (-0.132-0.196) | <0.0001 | <0.0001 |  |
| Morphine | Decrease | 71.4 (63.3-81.5) | 29 (12-48.3) | <0.0001 | <0.0001 | 63.6 (56-73.1) | 20.7 (4.35-38.5) | <0.0001 | <0.0001 | 0.374 (0.279-0.473) | 0.0436 (-0.117-0.201) | <0.0001 | <0.0001 |  |
| Noradrenaline | Decrease | 70.4 (63-80.6) | 29.6 (11.8-50) | <0.0001 | <0.0001 | 62.5 (55.6-72.4) | 20.7 (6.67-40.5) | <0.0001 | <0.0001 | 0.365 (0.272-0.462) | 0.0359 (-0.126-0.196) | <0.0001 | <0.0001 |  |
| Noradrenaline | Increase | 70.4 (63-80.8) | 28.6 (11.1-48.3) | <0.0001 | <0.0001 | 62.5 (55.8-73.1) | 20 (4.17-39.3) | <0.0001 | <0.0001 | 0.366 (0.278-0.468) | 0.0288 (-0.127-0.189) | <0.0001 | <0.0001 |  |
| Propofol | Decrease | 70.8 (63-81.5) | 29.6 (13.8-48.6) | <0.0001 | <0.0001 | 62.5 (56-73.1) | 20.8 (7.14-40) | <0.0001 | <0.0001 | 0.375 (0.283-0.476) | 0.0411 (-0.104-0.203) | <0.0001 | <0.0001 |  |
| Propofol | Increase | 71.4 (63-81.6) | 29.6 (12-50) | <0.0001 | <0.0001 | 63 (55.6-74.1) | 20.7 (6.9-40) | <0.0001 | <0.0001 | 0.375 (0.273-0.476) | 0.0437 (-0.11-0.198) | <0.0001 | <0.0001 |  |
| Vasopressin | Decrease | 72 (65.6-79) | 15.6 (10.7-38.4) | 0.000499 | 0.0135 | 66.7 (63.9-71) | 11.5 (4.79-35.1) | 0.000138 | 0.00372 | 0.396 (0.336-0.504) | 0.073 (-0.148-0.164) | <0.0001 | 0.0023 |  |
| **Name** | **Mean Dose Change** | **% time LPRx_15 > 0** | | | | **% time LPRx_15 > 0.25** | | | | **% time LPRx_15 > 0.35** | | | |  |
|  |  | **Pre Dose** | **Post Dose** | **P value** | **Adj P Value** | **Pre Dose** | **Post Dose** | **P value** | **Adj P Value** | **Pre Dose** | **Post Dose** | **P value** | **Adj P Value** |  |
| Dobutamine | Decrease | 88.3 (78.2-100) | 53.6 (33.8-75.8) | <0.0001 | <0.0001 | 69.2 (57.1-81.8) | 22.4 (4.79-46) | <0.0001 | <0.0001 | 59.4 (46.2-68.9) | 11.7 (0-36.1) | <0.0001 | <0.0001 |  |
| Dobutamine | Increase | 89.5 (76.9-100) | 58.6 (27.8-76.4) | <0.0001 | <0.0001 | 73.3 (61.5-89.1) | 27.6 (5.83-46.2) | <0.0001 | <0.0001 | 64.7 (49.9-78.6) | 19.5 (0-32.1) | <0.0001 | <0.0001 |  |
| Midazolam | Increase | 88.9 (75-100) | 51.9 (31-75) | <0.0001 | <0.0001 | 69.2 (57.7-84) | 23.1 (3.7-44.4) | <0.0001 | <0.0001 | 60.7 (50-74.1) | 13 (0-35.7) | <0.0001 | <0.0001 |  |
| Midazolam | Decrease | 88.5 (74.8-100) | 55.6 (34.5-73.9) | <0.0001 | <0.0001 | 66.7 (55.6-81) | 23.1 (4.35-47.8) | <0.0001 | <0.0001 | 58.6 (46.7-71.4) | 13 (0-34.5) | <0.0001 | <0.0001 |  |
| Morphine | Increase | 89.3 (75-100) | 53.6 (32-75) | <0.0001 | <0.0001 | 70.8 (57.7-86.2) | 22.6 (4-45.9) | <0.0001 | <0.0001 | 60.7 (47.9-76) | 12.2 (0-33.5) | <0.0001 | <0.0001 |  |
| Morphine | Decrease | 89.3 (75.4-100) | 55.2 (33.3-76.1) | <0.0001 | <0.0001 | 70.4 (57.4-84.6) | 25 (7.14-46.4) | <0.0001 | <0.0001 | 60 (46.4-73.1) | 14.3 (0-34.6) | <0.0001 | <0.0001 |  |
| Noradrenaline | Decrease | 86.7 (74.1-100) | 53.8 (32.1-75) | <0.0001 | <0.0001 | 68 (56-81.5) | 24.1 (6.25-45.8) | <0.0001 | <0.0001 | 58.3 (46.7-72) | 13.8 (0-34.5) | <0.0001 | <0.0001 |  |
| Noradrenaline | Increase | 87.5 (75-100) | 53.6 (32.1-75) | <0.0001 | <0.0001 | 69 (56-82.6) | 23.1 (4.35-45.6) | <0.0001 | <0.0001 | 58.6 (47.6-73.1) | 13.8 (0-34.6) | <0.0001 | <0.0001 |  |
| Propofol | Decrease | 88.5 (75.2-100) | 55.6 (35.7-76.6) | <0.0001 | <0.0001 | 69.2 (57.7-82.9) | 26.7 (7.14-48.1) | <0.0001 | <0.0001 | 59.3 (48-73.1) | 16.1 (0-35.7) | <0.0001 | <0.0001 |  |
| Propofol | Increase | 88.5 (75-100) | 56 (34.5-75) | <0.0001 | <0.0001 | 69.2 (57.1-83.3) | 23.6 (6.9-46.4) | <0.0001 | <0.0001 | 59.3 (48-73.8) | 14.3 (0-35.7) | <0.0001 | <0.0001 |  |
| Vasopressin | Decrease | 90.3 (78.5-98) | 59.4 (28.3-77.8) | 0.0038 | 0.103 | 63.3 (56.1-74.2) | 41.9 (12.7-50.4) | 0.00312 | 0.0842 | 63.3 (54.5-67.7) | 16.7 (4.84-24.3) | 0.000137 | 0.00369 |  |
| **Name** | **Mean Dose Change** | **Mean LPRx_20** | | | | **% time LPRx_20 > 0** | | | | **% time LPRx_20 > 0.25** | | | |  |
|  |  | **Pre Dose** | **Post Dose** | **P value** | **Adj P Value** | **Pre Dose** | **Post Dose** | **P value** | **Adj P Value** | **Pre Dose** | **Post Dose** | **P value** | **Adj P Value** |  |
| Dobutamine | Decrease | 0.355 (0.214-0.48) | 0.0107 (-0.13-0.184) | <0.0001 | <0.0001 | 90.5 (72.5-100) | 52.7 (31.1-74.1) | <0.0001 | <0.0001 | 68.4 (50-82.1) | 16.7 (2.28-48) | <0.0001 | <0.0001 |  |
| Dobutamine | Increase | 0.326 (0.232-0.487) | 0.00429 (-0.15-0.159) | <0.0001 | <0.0001 | 90 (74.1-100) | 53.8 (25.9-74.6) | <0.0001 | <0.0001 | 69 (51.9-86.2) | 20.7 (1.16-42.8) | <0.0001 | <0.0001 |  |
| Midazolam | Increase | 0.333 (0.226-0.448) | 0.0302 (-0.131-0.18) | <0.0001 | <0.0001 | 89.7 (73.1-100) | 53.3 (31-75.9) | <0.0001 | <0.0001 | 66.7 (48.4-84) | 20 (0-44) | <0.0001 | <0.0001 |  |
| Midazolam | Decrease | 0.324 (0.209-0.446) | 0.0366 (-0.116-0.173) | <0.0001 | <0.0001 | 89.3 (70.3-100) | 55.2 (32-75) | <0.0001 | <0.0001 | 63 (46.2-81.5) | 18.5 (0-43.5) | <0.0001 | <0.0001 |  |
| Morphine | Increase | 0.338 (0.204-0.456) | 0.0261 (-0.116-0.189) | <0.0001 | <0.0001 | 91.8 (71.4-100) | 54.6 (30.8-76.7) | <0.0001 | <0.0001 | 66.7 (46.4-86.2) | 20.7 (0-44.9) | <0.0001 | <0.0001 |  |
| Morphine | Decrease | 0.338 (0.205-0.463) | 0.0412 (-0.106-0.193) | <0.0001 | <0.0001 | 89.3 (69.2-100) | 56 (33.3-79.3) | <0.0001 | <0.0001 | 66.7 (45.2-85.1) | 21.4 (3.03-44.4) | <0.0001 | <0.0001 |  |
| Noradrenaline | Decrease | 0.32 (0.204-0.445) | 0.0307 (-0.115-0.183) | <0.0001 | <0.0001 | 87 (69-100) | 53.8 (30.8-76.9) | <0.0001 | <0.0001 | 62.2 (45.8-80.6) | 20 (0-43.6) | <0.0001 | <0.0001 |  |
| Noradrenaline | Increase | 0.325 (0.208-0.454) | 0.0269 (-0.121-0.181) | <0.0001 | <0.0001 | 87.5 (70.4-100) | 53.8 (31-77.8) | <0.0001 | <0.0001 | 64 (46.2-81.8) | 18.2 (0-44.4) | <0.0001 | <0.0001 |  |
| Propofol | Decrease | 0.342 (0.219-0.473) | 0.04 (-0.1-0.208) | <0.0001 | <0.0001 | 89.3 (72.4-100) | 56 (34.5-80) | <0.0001 | <0.0001 | 65.5 (48.1-84) | 24 (3.45-48.2) | <0.0001 | <0.0001 |  |
| Propofol | Increase | 0.335 (0.211-0.459) | 0.0407 (-0.0913-0.208) | <0.0001 | <0.0001 | 89.3 (70.6-100) | 56 (34.5-80.8) | <0.0001 | <0.0001 | 65.1 (48.1-83.3) | 21.3 (2.35-47.7) | <0.0001 | <0.0001 |  |
| Vasopressin | Decrease | 0.333 (0.27-0.421) | 0.0912 (-0.131-0.225) | 0.00245 | 0.0661 | 87.1 (78.3-95.2) | 54.8 (29.6-86.1) | 0.0383 | 1 | 58.1 (54.8-65) | 30 (15.6-60.3) | 0.0758 | 1 |  |
| **Name** | **Mean Dose Change** | **% time LPRx_20 > 0.35** | | | | **Mean LPRx_30** | | | | **% time LPRx_30 > 0** | | | |  |
|  |  | **Pre Dose** | **Post Dose** | **P value** | **Adj P Value** | **Pre Dose** | **Post Dose** | **P value** | **Adj P Value** | **Pre Dose** | **Post Dose** | **P value** | **Adj P Value** |  |
| Dobutamine | Decrease | 56.4 (37.3-71.7) | 6.35 (0-34.3) | <0.0001 | <0.0001 | 0.285 (0.0855-0.45) | 0.0242 (-0.15-0.195) | <0.0001 | <0.0001 | 90.1 (57.9-100) | 53.2 (24.1-81.3) | <0.0001 | <0.0001 |  |
| Dobutamine | Increase | 58.5 (42.1-81.9) | 10 (0-29.8) | <0.0001 | <0.0001 | 0.272 (0.0428-0.464) | -0.0269 (-0.136-0.13) | <0.0001 | <0.0001 | 86.1 (60.6-100) | 44 (27.7-76.8) | <0.0001 | <0.0001 |  |
| Midazolam | Increase | 53.8 (37-73.1) | 7.41 (0-31) | <0.0001 | <0.0001 | 0.242 (0.079-0.393) | 0.0229 (-0.113-0.172) | <0.0001 | <0.0001 | 88 (57.7-100) | 53.3 (28.6-80.6) | <0.0001 | <0.0001 |  |
| Midazolam | Decrease | 51.9 (35.7-71) | 7.41 (0-30) | <0.0001 | <0.0001 | 0.222 (0.0809-0.394) | 0.0227 (-0.113-0.173) | <0.0001 | <0.0001 | 81.5 (58.6-100) | 52 (28.6-78.6) | <0.0001 | <0.0001 |  |
| Morphine | Increase | 53.7 (33.3-75) | 8.17 (0-31.1) | <0.0001 | <0.0001 | 0.239 (0.0784-0.42) | 0.0329 (-0.0999-0.206) | <0.0001 | <0.0001 | 86.2 (58-100) | 55.6 (29.2-86.2) | <0.0001 | <0.0001 |  |
| Morphine | Decrease | 53.6 (35.7-73.2) | 10.7 (0-33.3) | <0.0001 | <0.0001 | 0.243 (0.0891-0.418) | 0.0354 (-0.102-0.212) | <0.0001 | <0.0001 | 84.6 (59-100) | 57.5 (30.8-91.3) | <0.0001 | <0.0001 |  |
| Noradrenaline | Decrease | 51.7 (34.6-70.4) | 7.69 (0-32.1) | <0.0001 | <0.0001 | 0.223 (0.0736-0.387) | 0.027 (-0.106-0.186) | <0.0001 | <0.0001 | 82.1 (56-100) | 54.8 (27.8-84.6) | <0.0001 | <0.0001 |  |
| Noradrenaline | Increase | 51.9 (34.6-72) | 7.41 (0-32) | <0.0001 | <0.0001 | 0.235 (0.0805-0.413) | 0.0289 (-0.104-0.186) | <0.0001 | <0.0001 | 84 (57.1-100) | 55.2 (27.6-84) | <0.0001 | <0.0001 |  |
| Propofol | Decrease | 55.2 (37-74.4) | 11.1 (0-35.7) | <0.0001 | <0.0001 | 0.257 (0.0992-0.438) | 0.0523 (-0.085-0.23) | <0.0001 | <0.0001 | 87.5 (62.1-100) | 60 (31-89.7) | <0.0001 | <0.0001 |  |
| Propofol | Increase | 53.7 (35.7-73.3) | 9.84 (0-35.7) | <0.0001 | <0.0001 | 0.248 (0.0872-0.433) | 0.0576 (-0.0783-0.234) | <0.0001 | <0.0001 | 85.2 (58.6-100) | 60 (33.3-88.9) | <0.0001 | <0.0001 |  |
| Vasopressin | Decrease | 48.4 (46.7-53.2) | 10 (4.79-43.5) | 0.0414 | 1 | 0.154 (0.118-0.284) | 0.118 (-0.00426-0.267) | 0.332 | 1 | 74.2 (64-95.2) | 61.3 (41-88.8) | 0.263 | 1 |  |
| **Name** | **Mean Dose Change** | **% time LPRx_30 > 0.25** | | | | **% time LPRx_30 > 0.35** | | | | **Mean LPRx_60** | | | |  |
|  |  | **Pre Dose** | **Post Dose** | **P value** | **Adj P Value** | **Pre Dose** | **Post Dose** | **P value** | **Adj P Value** | **Pre Dose** | **Post Dose** | **P value** | **Adj P Value** |  |
| Dobutamine | Decrease | 59 (24-87.9) | 13.4 (0-38.4) | <0.0001 | <0.0001 | 43.3 (11.7-72.2) | 0 (0-27.6) | <0.0001 | <0.0001 | 0.0914 (-0.0664-0.322) | 0.1 (-0.0739-0.307) | 0.792 | 1 |  |
| Dobutamine | Increase | 57.7 (23.5-84.5) | 8 (0-35.9) | <0.0001 | <0.0001 | 37.2 (15.1-79.6) | 0 (0-16.3) | <0.0001 | <0.0001 | 0.0995 (-0.0167-0.32) | 0.0573 (-0.0776-0.286) | 0.0313 | 0.846 |  |
| Midazolam | Increase | 51.9 (22.7-80) | 15.4 (0-46.2) | <0.0001 | <0.0001 | 35.5 (10.7-64.3) | 3.23 (0-28.6) | <0.0001 | <0.0001 | 0.0816 (-0.0806-0.304) | 0.0878 (-0.0801-0.294) | 0.875 | 1 |  |
| Midazolam | Decrease | 48.1 (23.1-75.9) | 14.3 (0-40) | <0.0001 | <0.0001 | 36 (10.7-63) | 0 (0-27.6) | <0.0001 | <0.0001 | 0.0554 (-0.0984-0.29) | 0.0626 (-0.0744-0.287) | 0.468 | 1 |  |
| Morphine | Increase | 51.7 (20.7-83.9) | 16.1 (0-45.6) | <0.0001 | <0.0001 | 37.1 (7.34-69.1) | 3.7 (0-30.8) | <0.0001 | <0.0001 | 0.11 (-0.0735-0.342) | 0.0851 (-0.0673-0.317) | 0.359 | 1 |  |
| Morphine | Decrease | 51.9 (25-81.5) | 16.1 (0-46.4) | <0.0001 | <0.0001 | 37.5 (12.1-68) | 3.85 (0-32.1) | <0.0001 | <0.0001 | 0.109 (-0.0953-0.351) | 0.0802 (-0.0651-0.323) | 0.664 | 1 |  |
| Noradrenaline | Decrease | 46.9 (23.1-75) | 12.5 (0-42.3) | <0.0001 | <0.0001 | 33.3 (10.3-62.1) | 0 (0-27.6) | <0.0001 | <0.0001 | 0.0711 (-0.0855-0.29) | 0.0672 (-0.0907-0.294) | 0.653 | 1 |  |
| Noradrenaline | Increase | 50 (22.2-78.3) | 13.6 (0-42.3) | <0.0001 | <0.0001 | 36 (10.7-65.3) | 0 (0-27.6) | <0.0001 | <0.0001 | 0.0897 (-0.077-0.324) | 0.0755 (-0.0755-0.298) | 0.131 | 1 |  |
| Propofol | Decrease | 54.2 (26-83.3) | 17.8 (0-50) | <0.0001 | <0.0001 | 40 (14.3-71) | 4 (0-36.6) | <0.0001 | <0.0001 | 0.127 (-0.049-0.369) | 0.121 (-0.0579-0.376) | 0.763 | 1 |  |
| Propofol | Increase | 52 (24.1-82.8) | 18.5 (0-51.7) | <0.0001 | <0.0001 | 38.5 (11.5-71.4) | 4.94 (0-36) | <0.0001 | <0.0001 | 0.118 (-0.0467-0.365) | 0.127 (-0.0501-0.377) | 0.709 | 1 |  |
| Vasopressin | Decrease | 35.5 (32.3-45.8) | 38.7 (0-59.2) | 0.741 | 1 | 29 (20-40.9) | 22.6 (0-54.8) | 0.426 | 1 | 0.154 (-0.0505-0.232) | 0.121 (0.0596-0.569) | 0.652 | 1 |  |
| **Name** | **Mean Dose Change** | **% time LPRx_60 > 0** | | | | **% time LPRx_60 > 0.25** | | | | **% time LPRx_60 > 0.35** | | | |  |
|  |  | **Pre Dose** | **Post Dose** | **P value** | **Adj P Value** | **Pre Dose** | **Post Dose** | **P value** | **Adj P Value** | **Pre Dose** | **Post Dose** | **P value** | **Adj P Value** |  |
| Dobutamine | Decrease | 74.2 (19.4-100) | 78.2 (23.9-100) | 0.739 | 1 | 11.1 (0-75.4) | 7.55 (0-66.4) | 0.534 | 1 | 0 (0-52.4) | 0 (0-50) | 0.595 | 1 |  |
| Dobutamine | Increase | 82.1 (32.6-100) | 66.7 (18-100) | 0.0723 | 1 | 20 (0-80.2) | 0 (0-59.4) | 0.156 | 1 | 0 (0-48.1) | 0 (0-38.2) | 0.328 | 1 |  |
| Midazolam | Increase | 71.4 (23.1-100) | 71 (17.2-100) | 0.846 | 1 | 10.7 (0-63) | 3.57 (0-66.7) | 0.596 | 1 | 0 (0-42.9) | 0 (0-40.9) | 0.453 | 1 |  |
| Midazolam | Decrease | 65.2 (20.7-100) | 70.4 (17.9-100) | 0.846 | 1 | 4.17 (0-58.6) | 3.33 (0-65.5) | 0.808 | 1 | 0 (0-40.7) | 0 (0-40) | 0.441 | 1 |  |
| Morphine | Increase | 83.3 (19.3-100) | 76.8 (24.8-100) | 0.663 | 1 | 15.7 (0-75) | 8.93 (0-69) | 0.285 | 1 | 0 (0-53.3) | 0 (0-46.2) | 0.216 | 1 |  |
| Morphine | Decrease | 76.9 (19.6-100) | 77.4 (24.1-100) | 0.98 | 1 | 20.8 (0-76.9) | 7.69 (0-69) | 0.0795 | 1 | 0 (0-56.5) | 0 (0-44.4) | 0.0728 | 1 |  |
| Noradrenaline | Decrease | 69.2 (20.7-100) | 70.4 (16-100) | 0.893 | 1 | 6.9 (0-61.5) | 0 (0-63.6) | 0.0351 | 0.947 | 0 (0-44) | 0 (0-40) | 0.046 | 1 |  |
| Noradrenaline | Increase | 75 (23.1-100) | 72.4 (19.2-100) | 0.508 | 1 | 10.3 (0-68) | 2.7 (0-65.4) | 0.00129 | 0.0348 | 0 (0-47.8) | 0 (0-42.9) | <0.0001 | 0.00237 |  |
| Propofol | Decrease | 82 (30-100) | 82.1 (29.6-100) | 0.672 | 1 | 21.1 (0-76) | 14.6 (0-79.3) | 0.662 | 1 | 0 (0-58.8) | 0 (0-58.5) | 0.555 | 1 |  |
| Propofol | Increase | 81.5 (30-100) | 84.6 (29.2-100) | 0.382 | 1 | 17.9 (0-74.1) | 16.7 (0-79.2) | 0.671 | 1 | 0 (0-59) | 0 (0-59.3) | 0.755 | 1 |  |
| Vasopressin | Decrease | 93.5 (33.3-100) | 100 (76.7-100) | 0.605 | 1 | 16.1 (0-54.8) | 23.3 (0-100) | 0.734 | 1 | 0 (0-11.3) | 3.33 (0-83.2) | 0.518 | 1 |  |
| **Bolus** | | | | | | | | | | | | | |  |
| **Name** | **Doses** | **Mean MAP** | | | | **Mean CPP** | | | | **% time CPP>60** | | | |  |
|  |  | **Pre Dose** | **Post Dose** | **P value** | **Adj P Value** | **Pre Dose** | **Post Dose** | **P value** | **Adj P Value** | **Pre Dose** | **Post Dose** | **P value** | **Adj P Value** |  |
| Ephedrine | 7 | 64.4 (61.6-76.3) | 80.9 (76.4-86.9) | 0.0728 | 1 | 47.3 (45.6-58.8) | 67.2 (61.2-68.1) | 0.0728 | 1 | 81.5 (58.9-95.7) | 21.7 (14.5-47.2) | 0.177 | 1 |  |
| Fentanyl | 16 | 83.4 (71-93.7) | 85.9 (72.1-90.3) | 0.836 | 1 | 69.5 (53.9-85.9) | 74.5 (60-80.5) | 0.637 | 1 | 0 (0-84.6) | 9.52 (0-57) | 0.693 | 1 |  |
| Midazolam | 95 | 80.7 (74.9-86.6) | 79.1 (73.5-85.3) | 0.278 | 1 | 63.6 (58.1-70.7) | 63 (58.3-68.6) | 0.484 | 1 | 10.7 (0-72.2) | 20 (0-75) | 0.491 | 1 |  |
| Morphine | 500 | 82.3 (74.6-91.4) | 80.8 (73.5-89.8) | 0.0223 | 0.601 | 68.9 (60.3-79.9) | 67.4 (59.2-76.7) | 0.0553 | 1 | 0 (0-50.4) | 5.2 (0-65.2) | 0.0368 | 0.994 |  |
| Propofol | 462 | 80.9 (74.5-88.4) | 79.1 (73.3-86.5) | 0.0184 | 0.498 | 66.4 (59-74.3) | 64.4 (59.2-73.2) | 0.155 | 1 | 6.94 (0-65.5) | 15.1 (0-60.5) | 0.0453 | 1 |  |
| Alfentanil | 37 | 76.4 (72.4-83.6) | 74.9 (69.2-80.3) | 0.327 | 1 | 65.4 (59.9-72.1) | 61.9 (56.2-70.2) | 0.261 | 1 | 14.8 (0-59.3) | 29.6 (0-83.3) | 0.427 | 1 |  |
| **Name** | **% time CPP>70** | | | | **Mean ICP** | | | | **% time ICP>20** | | | |  |  |
|  | **Pre Dose** | **Post Dose** | **P value** | **Adj P Value** | **Pre Dose** | **Post Dose** | **P value** | **Adj P Value** | **Pre Dose** | **Post Dose** | **P value** | **Adj P Value** |  |  |
| Ephedrine | 0 (0-13.4) | 35.7 (18.5-41.7) | 0.0894 | 1 | 16.2 (12.3-17.3) | 16.5 (11.8-19.5) | 0.62 | 1 | 0 (0-22) | 0 (0-44.3) | 0.571 | 1 |  |  |
| Fentanyl | 34.6 (0-100) | 57.1 (7.49-86.6) | 0.801 | 1 | 13.2 (7.29-14.4) | 6.76 (5.27-14.4) | 0.168 | 1 | 0 (0-5.56) | 0 (0-3.57) | 0.828 | 1 |  |  |
| Midazolam | 4.76 (0-60.3) | 0 (0-38.8) | 0.524 | 1 | 17.1 (13.5-19.9) | 17.1 (13.2-20.4) | 0.872 | 1 | 4 (0-51.6) | 3.85 (0-50) | 0.779 | 1 |  |  |
| Morphine | 35.4 (0-100) | 24.3 (0-93) | 0.125 | 1 | 13.1 (8.84-17.1) | 12.9 (8.81-17.4) | 0.945 | 1 | 0 (0-8.6) | 0 (0-7.69) | 0.545 | 1 |  |  |
| Propofol | 14.4 (0-82.7) | 9.84 (0-71.9) | 0.281 | 1 | 14.3 (10.1-18) | 13.9 (9.14-17.8) | 0.233 | 1 | 0 (0-13.9) | 0 (0-11.1) | 0.139 | 1 |  |  |
| Alfentanil | 12 (0-69) | 7.14 (0-51.9) | 0.543 | 1 | 11.7 (7.9-15.3) | 10.9 (8.54-15) | 0.983 | 1 | 0 (0-0) | 0 (0-4) | 0.468 | 1 |  |  |
| **Name** | **% time ICP>22** | | | | **Mean LPRx_10** | | | | **% time LPRx_10 > 0** | | | |  |  |
|  | **Pre Dose** | **Post Dose** | **P value** | **Adj P Value** | **Pre Dose** | **Post Dose** | **P value** | **Adj P Value** | **Pre Dose** | **Post Dose** | **P value** | **Adj P Value** |  |  |
| Ephedrine | 0 (0-3.7) | 0 (0-5.56) | 1 | 1 | 0.356 (0.307-0.403) | -0.119 (-0.199-0.115) | 0.00233 | 0.0629 | 75 (71.8-88.5) | 33.3 (28.6-57) | 0.0728 | 1 |  |  |
| Fentanyl | 0 (0-0) | 0 (0-2.38) | 0.285 | 1 | 0.302 (0.258-0.366) | -0.0995 (-0.247-0.119) | <0.0001 | <0.0001 | 76.6 (70.5-88.5) | 31.7 (23.9-59.5) | <0.0001 | 0.000173 |  |  |
| Midazolam | 0 (0-13.8) | 0 (0-18) | 0.953 | 1 | 0.383 (0.315-0.443) | 0.0378 (-0.0901-0.186) | <0.0001 | <0.0001 | 85 (76-96.4) | 58.3 (37.6-71) | <0.0001 | <0.0001 |  |  |
| Morphine | 0 (0-2.72) | 0 (0-3.36) | 0.877 | 1 | 0.393 (0.324-0.48) | 0.0862 (-0.0694-0.238) | <0.0001 | <0.0001 | 87.8 (76.9-96.8) | 59.3 (40.9-76.8) | <0.0001 | <0.0001 |  |  |
| Propofol | 0 (0-4) | 0 (0-3.7) | 0.449 | 1 | 0.385 (0.323-0.466) | 0.0718 (-0.0956-0.238) | <0.0001 | <0.0001 | 84.6 (76.2-95.8) | 58.2 (40-74.4) | <0.0001 | <0.0001 |  |  |
| Alfentanil | 0 (0-0) | 0 (0-0) | 0.474 | 1 | 0.407 (0.357-0.508) | 0.0748 (-0.0971-0.188) | <0.0001 | <0.0001 | 86.2 (76.9-92.6) | 56 (39.3-68) | <0.0001 | <0.0001 |  |  |
| **Name** | **% time LPRx_10 > 0.25** | | | | **% time LPRx_10 > 0.35** | | | | **Mean LPRx_15** | | | |  |  |
|  | **Pre Dose** | **Post Dose** | **P value** | **Adj P Value** | **Pre Dose** | **Post Dose** | **P value** | **Adj P Value** | **Pre Dose** | **Post Dose** | **P value** | **Adj P Value** |  |  |
| Ephedrine | 70.8 (66.5-73.6) | 17.4 (16.6-42.6) | 0.00117 | 0.0315 | 60.9 (55.3-63.9) | 13.8 (8.17-23.2) | 0.00408 | 0.11 | 0.312 (0.249-0.354) | -0.0489 (-0.26-0.114) | 0.0111 | 0.299 |  |  |
| Fentanyl | 55.5 (53.5-66.9) | 7.14 (2.5-37) | <0.0001 | <0.0001 | 55.5 (53.5-60.7) | 0 (0-14.8) | <0.0001 | <0.0001 | 0.274 (0.247-0.318) | -0.113 (-0.197-0.0673) | <0.0001 | 0.000822 |  |  |
| Midazolam | 71.4 (61.5-80) | 31 (14.8-50) | <0.0001 | <0.0001 | 61.5 (55.6-71) | 25.9 (4.26-40.3) | <0.0001 | <0.0001 | 0.362 (0.269-0.456) | 0.0432 (-0.111-0.19) | <0.0001 | <0.0001 |  |  |
| Morphine | 71.4 (63.9-84.3) | 32.5 (17.9-52.2) | <0.0001 | <0.0001 | 62.8 (56.1-74.5) | 23.9 (10.3-42.2) | <0.0001 | <0.0001 | 0.388 (0.297-0.491) | 0.0737 (-0.0713-0.233) | <0.0001 | <0.0001 |  |  |
| Propofol | 71 (63-80) | 34.5 (16.7-51.7) | <0.0001 | <0.0001 | 62.1 (55.6-71.4) | 25.9 (8.56-42.1) | <0.0001 | <0.0001 | 0.371 (0.29-0.472) | 0.079 (-0.073-0.23) | <0.0001 | <0.0001 |  |  |
| Alfentanil | 73.9 (68-81.8) | 30.8 (16-50) | <0.0001 | <0.0001 | 66.7 (60-75.9) | 18.5 (10.7-33.3) | <0.0001 | <0.0001 | 0.436 (0.312-0.513) | 0.0117 (-0.123-0.185) | <0.0001 | <0.0001 |  |  |
| **Name** | **% time LPRx_15 > 0** | | | | **% time LPRx_15 > 0.25** | | | | **% time LPRx_15 > 0.35** | | | |  |  |
|  | **Pre Dose** | **Post Dose** | **P value** | **Adj P Value** | **Pre Dose** | **Post Dose** | **P value** | **Adj P Value** | **Pre Dose** | **Post Dose** | **P value** | **Adj P Value** |  |  |
| Ephedrine | 75 (63.6-80.9) | 34.5 (23.5-65.5) | 0.0728 | 1 | 60.9 (56.1-66.9) | 29.6 (14.2-41.7) | 0.0175 | 0.472 | 55.6 (52.6-56.8) | 20 (11.4-34) | 0.0379 | 1 |  |  |
| Fentanyl | 78.7 (67.9-85.6) | 35.7 (15.1-56.7) | 0.000127 | 0.00342 | 59.3 (57-69.2) | 5.63 (2.5-18.5) | 0.000265 | 0.00716 | 53.3 (50-57.1) | 1.85 (0-8.33) | 0.000569 | 0.0154 |  |  |
| Midazolam | 88 (73.1-100) | 50 (36.7-72.9) | <0.0001 | <0.0001 | 69.4 (57.4-83.5) | 26.9 (10.3-45.8) | <0.0001 | <0.0001 | 57.7 (46.3-72) | 17.9 (3.58-38.5) | <0.0001 | <0.0001 |  |  |
| Morphine | 92.6 (77.8-100) | 59.4 (37.9-79.3) | <0.0001 | <0.0001 | 72 (60-87.5) | 29.9 (10.6-52.5) | <0.0001 | <0.0001 | 61.5 (50-75) | 18.5 (3.57-42.3) | <0.0001 | <0.0001 |  |  |
| Propofol | 89 (74.3-100) | 60 (39.2-79.1) | <0.0001 | <0.0001 | 69.7 (57.1-85) | 29.4 (10.8-53.8) | <0.0001 | <0.0001 | 59.3 (47.4-74.3) | 18.8 (0-40.6) | <0.0001 | <0.0001 |  |  |
| Alfentanil | 89.3 (73.3-100) | 48 (32.1-80) | <0.0001 | 0.00013 | 73.1 (63-86.4) | 24 (7.14-48) | <0.0001 | <0.0001 | 63 (56-78.3) | 16.7 (0-40) | <0.0001 | <0.0001 |  |  |
| **Name** | **Mean LPRx_20** | | | | **% time LPRx_20 > 0** | | | | **% time LPRx_20 > 0.25** | | | |  |  |
|  | **Pre Dose** | **Post Dose** | **P value** | **Adj P Value** | **Pre Dose** | **Post Dose** | **P value** | **Adj P Value** | **Pre Dose** | **Post Dose** | **P value** | **Adj P Value** |  |  |
| Ephedrine | 0.21 (0.158-0.309) | 0.0328 (-0.255-0.123) | 0.0262 | 0.708 | 63 (55.6-80.7) | 51.7 (22.2-62.2) | 0.125 | 1 | 50 (46.7-58.2) | 37.9 (13-43.7) | 0.0547 | 1 |  |  |
| Fentanyl | 0.298 (0.177-0.328) | -0.00263 (-0.189-0.074) | 0.00096 | 0.0259 | 78.8 (64.1-91.3) | 41.2 (30.4-63.3) | 0.00432 | 0.117 | 73.1 (35.7-75) | 9.47 (0-25) | <0.0001 | 0.00149 |  |  |
| Midazolam | 0.318 (0.22-0.424) | 0.0057 (-0.129-0.159) | <0.0001 | <0.0001 | 88.5 (71.4-100) | 50 (27.6-78.9) | <0.0001 | <0.0001 | 66.7 (50-84) | 22.9 (3.39-42.9) | <0.0001 | <0.0001 |  |  |
| Morphine | 0.362 (0.256-0.488) | 0.0711 (-0.0693-0.252) | <0.0001 | <0.0001 | 93.1 (78.4-100) | 60.3 (35.5-82.9) | <0.0001 | <0.0001 | 70.8 (52-88.9) | 25.9 (3.7-55.2) | <0.0001 | <0.0001 |  |  |
| Propofol | 0.346 (0.235-0.473) | 0.0688 (-0.0735-0.241) | <0.0001 | <0.0001 | 92.3 (72-100) | 60.7 (38.5-81.4) | <0.0001 | <0.0001 | 67.4 (48.3-86.3) | 27.7 (6.9-53.8) | <0.0001 | <0.0001 |  |  |
| Alfentanil | 0.392 (0.274-0.524) | -0.0342 (-0.146-0.184) | <0.0001 | <0.0001 | 92.6 (74.1-100) | 46.4 (22.2-78.6) | <0.0001 | <0.0001 | 72 (50-88.9) | 22.2 (0-44) | <0.0001 | <0.0001 |  |  |
| **Name** | **% time LPRx_20 > 0.35** | | | | **Mean LPRx_30** | | | | **% time LPRx_30 > 0** | | | |  |  |
|  | **Pre Dose** | **Post Dose** | **P value** | **Adj P Value** | **Pre Dose** | **Post Dose** | **P value** | **Adj P Value** | **Pre Dose** | **Post Dose** | **P value** | **Adj P Value** |  |  |
| Ephedrine | 43.5 (34.2-47.1) | 28 (13-36.6) | 0.0733 | 1 | 0.12 (0.0867-0.232) | 0.138 (-0.0849-0.178) | 0.535 | 1 | 69.6 (46.9-83) | 57.9 (35-78.5) | 0.902 | 1 |  |  |
| Fentanyl | 50 (32.4-75) | 1.85 (0-25) | 0.000437 | 0.0118 | 0.284 (-0.0429-0.461) | -0.0223 (-0.147-0.295) | 0.168 | 1 | 84.9 (46.6-97.1) | 55.8 (25.8-88.9) | 0.332 | 1 |  |  |
| Midazolam | 55.6 (37-69.5) | 7.69 (0-32.9) | <0.0001 | <0.0001 | 0.231 (0.114-0.373) | 0.00751 (-0.164-0.192) | <0.0001 | <0.0001 | 84.6 (63.2-100) | 53.3 (21.8-78.5) | <0.0001 | <0.0001 |  |  |
| Morphine | 56.5 (39.8-77.4) | 13.6 (0-41.8) | <0.0001 | <0.0001 | 0.307 (0.159-0.457) | 0.0821 (-0.0771-0.282) | <0.0001 | <0.0001 | 96 (70.8-100) | 65 (31.7-95.2) | <0.0001 | <0.0001 |  |  |
| Propofol | 55.9 (36.6-76) | 15.4 (0-41.4) | <0.0001 | <0.0001 | 0.283 (0.124-0.459) | 0.0849 (-0.0604-0.267) | <0.0001 | <0.0001 | 90.4 (65.4-100) | 67 (39.3-92.6) | <0.0001 | <0.0001 |  |  |
| Alfentanil | 63 (44.4-83.3) | 12.5 (0-44) | <0.0001 | <0.0001 | 0.388 (0.12-0.518) | -0.0481 (-0.182-0.194) | <0.0001 | 0.00074 | 90.3 (58.6-100) | 38.1 (0-78.3) | <0.0001 | 0.00124 |  |  |
| **Name** | **% time LPRx_30 > 0.25** | | | | **% time LPRx_30 > 0.35** | | | | **Mean LPRx_60** | | | |  |  |
|  | **Pre Dose** | **Post Dose** | **P value** | **Adj P Value** | **Pre Dose** | **Post Dose** | **P value** | **Adj P Value** | **Pre Dose** | **Post Dose** | **P value** | **Adj P Value** |  |  |
| Ephedrine | 48.1 (34.3-54.3) | 51.9 (22.3-57.4) | 1 | 1 | 32.1 (24.3-43.5) | 36 (3.96-42.6) | 1 | 1 | 0.0859 (0.0305-0.177) | 0.188 (0.061-0.347) | 0.318 | 1 |  |  |
| Fentanyl | 78.6 (21.2-84.1) | 14.3 (0-54.8) | 0.0109 | 0.293 | 75 (11.3-80.5) | 0 (0-33.3) | 0.00181 | 0.049 | 0.34 (-0.224-0.623) | 0.12 (0.0229-0.32) | 0.336 | 1 |  |  |
| Midazolam | 50 (29.5-82.1) | 19.2 (0-45.6) | <0.0001 | <0.0001 | 34.3 (15.1-59.3) | 11.1 (0-27.3) | <0.0001 | <0.0001 | 0.126 (-0.0634-0.358) | -0.00534 (-0.125-0.283) | 0.114 | 1 |  |  |
| Morphine | 62.3 (36.5-91.7) | 22.2 (0-55.9) | <0.0001 | <0.0001 | 47.8 (19.2-77.7) | 12 (0-44) | <0.0001 | <0.0001 | 0.181 (-0.00148-0.412) | 0.177 (-0.0438-0.431) | 0.805 | 1 |  |  |
| Propofol | 58.1 (30.4-87.5) | 22.2 (0-53.8) | <0.0001 | <0.0001 | 42.1 (15.4-75) | 10.3 (0-40.6) | <0.0001 | <0.0001 | 0.153 (-0.0349-0.398) | 0.133 (-0.0349-0.404) | 0.804 | 1 |  |  |
| Alfentanil | 69.2 (40-88.9) | 7.69 (0-32.3) | <0.0001 | 0.000144 | 59.3 (27.6-82.8) | 3.85 (0-19.4) | <0.0001 | 0.000261 | 0.142 (-0.0372-0.377) | -0.069 (-0.164-0.2) | 0.0568 | 1 |  |  |
| **Name** | **% time LPRx_60 > 0** | | | | **% time LPRx_60 > 0.25** | | | | **% time LPRx_60 > 0.35** | | | |  |  |
|  | **Pre Dose** | **Post Dose** | **P value** | **Adj P Value** | **Pre Dose** | **Post Dose** | **P value** | **Adj P Value** | **Pre Dose** | **Post Dose** | **P value** | **Adj P Value** |  |  |
| Ephedrine | 63 (37.8-93.5) | 85.2 (62.9-100) | 0.601 | 1 | 35.7 (13.9-59.2) | 57.1 (13.2-89.6) | 0.608 | 1 | 22.2 (9.09-31.3) | 46.4 (2.63-56.4) | 0.518 | 1 |  |  |
| Fentanyl | 100 (0-100) | 70 (52.9-89.3) | 0.376 | 1 | 60.6 (0-100) | 17.3 (0-64.3) | 0.118 | 1 | 43.5 (0-92.3) | 0 (0-39.3) | 0.146 | 1 |  |  |
| Midazolam | 77.1 (40.7-100) | 51.9 (15.7-100) | 0.136 | 1 | 22.2 (0-83.6) | 8.7 (0-59.6) | 0.252 | 1 | 0 (0-60.2) | 0 (0-26.6) | 0.29 | 1 |  |  |
| Morphine | 92.4 (47.7-100) | 100 (36-100) | 0.717 | 1 | 36.2 (0-86) | 28.4 (0-97.4) | 0.756 | 1 | 12.1 (0-69.1) | 7.06 (0-77.3) | 0.742 | 1 |  |  |
| Propofol | 92.6 (38.5-100) | 88.7 (36.7-100) | 0.494 | 1 | 25 (0-83.3) | 25 (0-85.3) | 0.852 | 1 | 7.02 (0-67.3) | 3.06 (0-67.8) | 0.69 | 1 |  |  |
| Alfentanil | 82.6 (38.5-100) | 33.3 (0-100) | 0.0463 | 1 | 25 (0-70.8) | 0 (0-42.9) | 0.125 | 1 | 0 (0-63) | 0 (0-12) | 0.169 | 1 |  |  |

*The table demonstrates the median and interquartile range of the pre/pose dose windows as well as the Wilcox signed ranked test between these windows with p values adjusted using the Bonferroni analysis. The “change” indicates if the continuous infusion was increase/decrease or was a bolus dose. CPP, cerebral prefusion pressure; ICP, intracranial pressure; LPRx_10, pressure reactivity over 10 minutes; LPRx_15, pressure reactivity over 15 minutes; LPRx_20, pressure reactivity over 20 minutes; LPRx_30, pressure reactivity over 30 minutes; LPRx_60, pressure reactivity over 60 minutes;*

# Appendix L. Pre-time window over 50% time L-PRx_10 < 0.35

The table contains all infusions given with the LPRx_10 pre-time window 50% < 0.35, separated into the continuous infusion doses then the bolus doses.

| **Continuous Intravenous** | | | | | | | | | | | | | | |
| --- | --- | --- | --- | --- | --- | --- | --- | --- | --- | --- | --- | --- | --- | --- |
| **Name** | **Doses** | **Mean Dose Change** | **Mean MAP** | | | | **Mean CPP** | | | | **% time CPP>60** | | | |
|  |  |  | **Pre Dose** | **Post Dose** | **P value** | **Adj P Value** | **Pre Dose** | **Post Dose** | **P value** | **Adj P Value** | **Pre Dose** | **Post Dose** | **P value** | **Adj P Value** |
| Dobutamine | 1361 | Decrease | 76.8 (71.2-84.3) | 76.1 (70.8-82.8) | 0.0495 | 1 | 64.6 (58.5-70.5) | 63.2 (58.9-69.1) | 0.0229 | 0.618 | 14.3 (0-72.7) | 17.9 (0-71.7) | 0.17 | 1 |
| Dobutamine | 1110 | Increase | 73.5 (68.1-79.9) | 72.7 (68.2-79.4) | 0.306 | 1 | 60.1 (54-66.4) | 59.6 (54.1-65.4) | 0.396 | 1 | 57.4 (10-100) | 62.7 (8.33-100) | 0.819 | 1 |
| Midazolam | 4794 | Decrease | 78.4 (73.2-84.5) | 78.2 (73.2-84.3) | 0.335 | 1 | 65.3 (59.9-71.1) | 64.9 (59.8-71) | 0.442 | 1 | 9.84 (0-55.2) | 10.3 (0-55.7) | 0.78 | 1 |
| Midazolam | 4553 | Increase | 78.9 (73.5-85) | 78.5 (73.2-84.6) | 0.0407 | 1 | 64.5 (59.2-71) | 64.5 (59.2-70.6) | 0.532 | 1 | 13.7 (0-63) | 11.8 (0-63.3) | 0.348 | 1 |
| Morphine | 7340 | Decrease | 77.8 (72.6-84.2) | 77.5 (72.3-83.7) | 0.147 | 1 | 65.6 (59.9-71.7) | 65.3 (59.6-71.5) | 0.0993 | 1 | 8 (0-55.2) | 9.68 (0-58.3) | 0.368 | 1 |
| Morphine | 7017 | Increase | 78.1 (72.7-84.7) | 77.4 (72.1-83.8) | <0.0001 | <0.0001 | 65.5 (59.7-71.9) | 64.8 (59.3-71.1) | 0.000311 | 0.00841 | 9.09 (0-58.3) | 10.3 (0-63) | 0.236 | 1 |
| Noradrenaline | 47518 | Decrease | 79.1 (73.5-85.4) | 78.4 (73-84.7) | <0.0001 | <0.0001 | 66.2 (60.6-72.7) | 65.6 (60.3-71.9) | <0.0001 | <0.0001 | 7.41 (0-46.2) | 7.41 (0-50) | 0.0835 | 1 |
| Noradrenaline | 44427 | Increase | 76.6 (71.2-83) | 76.6 (71.3-83) | 0.337 | 1 | 63.2 (58-69.5) | 63.6 (58.4-69.9) | <0.0001 | <0.0001 | 25 (0-76) | 18.5 (0-74.1) | <0.0001 | <0.0001 |
| Propofol | 14746 | Decrease | 77.8 (72.2-84.2) | 77.5 (72.1-84) | 0.112 | 1 | 65.4 (59.7-71.8) | 65.1 (59.5-71.7) | 0.0423 | 1 | 10.3 (0-57.4) | 10.7 (0-60) | 0.42 | 1 |
| Propofol | 13198 | Increase | 78.7 (73.1-85.4) | 77.6 (72.2-84.1) | <0.0001 | <0.0001 | 65.9 (60.1-72.6) | 65.1 (59.6-71.8) | <0.0001 | <0.0001 | 8 (0-53.1) | 10.3 (0-57.6) | 0.00368 | 0.0993 |
| Vasopressin | 67 | Decrease | 81.3 (73.3-84.1) | 80.1 (74-82.7) | 0.2 | 1 | 57.5 (55.2-59.1) | 58.7 (55.9-59.6) | 0.205 | 1 | 66 (58.9-81.7) | 60.8 (55.3-94.1) | 0.333 | 1 |
| Vasopressin | 63 | Increase | 75.5 (70.7-83.4) | 77.3 (70.7-82.6) | 0.556 | 1 | 55.3 (51-57.5) | 56.4 (51.4-58.7) | 0.275 | 1 | 91.3 (66-100) | 76.3 (60.8-100) | 0.339 | 1 |
| **Name** | **Mean Dose Change** | **% time CPP>70** | | | | **Mean ICP** | | | | **% time ICP>20** | | | |  |
|  |  | **Pre Dose** | **Post Dose** | **P value** | **Adj P Value** | **Pre Dose** | **Post Dose** | **P value** | **Adj P Value** | **Pre Dose** | **Post Dose** | **P value** | **Adj P Value** |  |
| Dobutamine | Decrease | 5 (0-47.6) | 3.33 (0-32.5) | 0.000152 | 0.00411 | 13.6 (9.64-16.7) | 13.5 (9.89-16.3) | 0.382 | 1 | 0 (0-4.35) | 0 (0-1.96) | 0.000148 | 0.00399 |  |
| Dobutamine | Increase | 0 (0-20) | 0 (0-11.5) | 0.0329 | 0.89 | 14.4 (10.7-17.5) | 14.3 (10.7-17) | 0.484 | 1 | 0 (0-9.52) | 0 (0-4.47) | 0.00416 | 0.112 |  |
| Midazolam | Decrease | 7.41 (0-54.1) | 6.9 (0-55.6) | 0.134 | 1 | 13.5 (10-16.6) | 13.5 (9.84-16.5) | 0.404 | 1 | 0 (0-5.04) | 0 (0-4) | 0.338 | 1 |  |
| Midazolam | Increase | 6.9 (0-52.2) | 4.54 (0-51.6) | 0.0205 | 0.554 | 14.4 (10.8-17.7) | 14.2 (10.6-17.3) | 0.0221 | 0.597 | 0 (0-10.8) | 0 (0-7.41) | <0.0001 | <0.0001 |  |
| Morphine | Decrease | 8.75 (0-59.3) | 7.69 (0-60) | 0.0478 | 1 | 12.4 (8.77-15.8) | 12.4 (8.84-15.8) | 0.962 | 1 | 0 (0-3.45) | 0 (0-3.23) | 0.503 | 1 |  |
| Morphine | Increase | 9.09 (0-60.7) | 6.81 (0-57.1) | <0.0001 | <0.0001 | 12.9 (9.14-16.4) | 12.7 (8.88-16.2) | 0.0162 | 0.437 | 0 (0-4.35) | 0 (0-3.57) | <0.0001 | <0.0001 |  |
| Noradrenaline | Decrease | 13.3 (0-69) | 8.33 (0-64.3) | <0.0001 | <0.0001 | 12.9 (9.35-16.2) | 12.9 (9.36-16.1) | 0.00651 | 0.176 | 0 (0-4.17) | 0 (0-3.45) | <0.0001 | <0.0001 |  |
| Noradrenaline | Increase | 4.17 (0-39.1) | 3.7 (0-43.3) | <0.0001 | 0.00235 | 13.5 (9.9-16.9) | 13.2 (9.48-16.5) | <0.0001 | <0.0001 | 0 (0-7.22) | 0 (0-3.7) | <0.0001 | <0.0001 |  |
| Propofol | Decrease | 8 (0-60.7) | 7.14 (0-60.7) | 0.00369 | 0.0996 | 12.4 (8.66-16) | 12.5 (8.69-16.1) | 0.611 | 1 | 0 (0-3.7) | 0 (0-3.45) | 0.204 | 1 |  |
| Propofol | Increase | 11.5 (0-66.7) | 7.41 (0-60.9) | <0.0001 | <0.0001 | 12.9 (9.01-16.5) | 12.6 (8.77-16.2) | <0.0001 | 0.00117 | 0 (0-6.9) | 0 (0-3.57) | <0.0001 | <0.0001 |  |
| Vasopressin | Decrease | 6.45 (0-6.81) | 5.53 (0-6.74) | 0.0912 | 1 | 15.2 (13.9-17.1) | 14.2 (12.7-16.9) | 0.205 | 1 | 7.22 (0-11.5) | 6.45 (0-8.87) | 0.489 | 1 |  |
| Vasopressin | Increase | 2.09 (0-6.77) | 0 (0-6.74) | 0.529 | 1 | 16.6 (15.1-18.8) | 16.9 (13.9-18.7) | 0.742 | 1 | 8.87 (0-13.5) | 7.22 (3.58-14.9) | 0.994 | 1 |  |
| **Name** | **Mean Dose Change** | **% time ICP>22** | | | | **Mean LPRx_10** | | | | **% time LPRx_10 > 0** | | | |  |
|  |  | **Pre Dose** | **Post Dose** | **P value** | **Adj P Value** | **Pre Dose** | **Post Dose** | **P value** | **Adj P Value** | **Pre Dose** | **Post Dose** | **P value** | **Adj P Value** |  |
| Dobutamine | Decrease | 0 (0-0) | 0 (0-0) | 0.000241 | 0.00651 | -0.032 (-0.179-0.11) | 0.0199 (-0.14-0.158) | <0.0001 | <0.0001 | 45.5 (28.3-63) | 51.1 (33.3-68) | <0.0001 | <0.0001 |  |
| Dobutamine | Increase | 0 (0-0) | 0 (0-0) | 0.0308 | 0.832 | -0.0324 (-0.183-0.103) | 0.00716 (-0.16-0.154) | <0.0001 | 0.00209 | 46.2 (28-63.4) | 50 (30.4-68) | 0.000629 | 0.017 |  |
| Midazolam | Decrease | 0 (0-0) | 0 (0-0) | 0.141 | 1 | -0.0227 (-0.179-0.101) | 0.00124 (-0.162-0.148) | <0.0001 | <0.0001 | 47.2 (28.6-61.7) | 50 (31-66.7) | <0.0001 | <0.0001 |  |
| Midazolam | Increase | 0 (0-3.45) | 0 (0-0) | <0.0001 | 0.000151 | -0.0231 (-0.176-0.104) | -0.000966 (-0.159-0.141) | <0.0001 | <0.0001 | 46.4 (28.6-62.1) | 50 (31-66.7) | <0.0001 | <0.0001 |  |
| Morphine | Decrease | 0 (0-0) | 0 (0-0) | 0.538 | 1 | -0.0201 (-0.165-0.101) | 0.00643 (-0.142-0.148) | <0.0001 | <0.0001 | 47.8 (30-62.1) | 50 (33.3-66.7) | <0.0001 | <0.0001 |  |
| Morphine | Increase | 0 (0-0) | 0 (0-0) | <0.0001 | <0.0001 | -0.0258 (-0.17-0.102) | 0.00229 (-0.153-0.15) | <0.0001 | <0.0001 | 46.4 (28.6-62.5) | 50 (32.1-66.7) | <0.0001 | <0.0001 |  |
| Noradrenaline | Decrease | 0 (0-0) | 0 (0-0) | <0.0001 | <0.0001 | -0.024 (-0.173-0.0996) | 0.00169 (-0.156-0.146) | <0.0001 | <0.0001 | 46.4 (29.2-62.1) | 50 (31-66.7) | <0.0001 | <0.0001 |  |
| Noradrenaline | Increase | 0 (0-0) | 0 (0-0) | <0.0001 | <0.0001 | -0.0242 (-0.175-0.0997) | -0.00178 (-0.16-0.146) | <0.0001 | <0.0001 | 46.4 (28.9-62.1) | 50 (30.8-66.7) | <0.0001 | <0.0001 |  |
| Propofol | Decrease | 0 (0-0) | 0 (0-0) | 0.0228 | 0.616 | -0.0257 (-0.171-0.103) | 0.00498 (-0.15-0.151) | <0.0001 | <0.0001 | 46.4 (29.6-62.1) | 50 (32.1-67.9) | <0.0001 | <0.0001 |  |
| Propofol | Increase | 0 (0-0.204) | 0 (0-0) | <0.0001 | <0.0001 | -0.0247 (-0.17-0.1) | -0.000295 (-0.157-0.148) | <0.0001 | <0.0001 | 46.4 (29.3-62.1) | 50 (31-67.9) | <0.0001 | <0.0001 |  |
| Vasopressin | Decrease | 1.07 (0-1.7) | 1.07 (0-1.3) | 0.654 | 1 | 0.0326 (0.0108-0.0383) | 0.0188 (0.00906-0.0316) | 0.195 | 1 | 53.3 (50-54.4) | 53.3 (51.2-54.2) | 0.791 | 1 |  |
| Vasopressin | Increase | 1.3 (0-2.37) | 1.3 (0-3.29) | 0.854 | 1 | 0.0188 (-0.0584-0.0475) | 0.0188 (-0.0143-0.0383) | 0.665 | 1 | 53.3 (40.8-56.9) | 51.7 (45-54) | 0.372 | 1 |  |
| **Name** | **Mean Dose Change** | **% time LPRx_10 > 0.25** | | | | **% time LPRx_10 > 0.35** | | | | **Mean LPRx_15** | | | |  |
|  |  | **Pre Dose** | **Post Dose** | **P value** | **Adj P Value** | **Pre Dose** | **Post Dose** | **P value** | **Adj P Value** | **Pre Dose** | **Post Dose** | **P value** | **Adj P Value** |  |
| Dobutamine | Decrease | 21.4 (7.41-37.9) | 26.9 (10-42.9) | <0.0001 | <0.0001 | 13.8 (2.78-28.6) | 18.5 (3.7-33.3) | <0.0001 | <0.0001 | -0.0311 (-0.187-0.108) | -0.00241 (-0.156-0.151) | <0.0001 | 0.000559 |  |
| Dobutamine | Increase | 22.2 (7.69-35.7) | 25.9 (10.7-41.3) | <0.0001 | <0.0001 | 14.8 (2.4-26.7) | 16.7 (3.85-33.1) | <0.0001 | 0.000896 | -0.033 (-0.177-0.0984) | -0.00262 (-0.166-0.146) | 0.00106 | 0.0286 |  |
| Midazolam | Decrease | 21.6 (7.41-36) | 24.1 (7.69-41.4) | <0.0001 | <0.0001 | 13.8 (3.33-26.9) | 15.4 (3.45-32.1) | <0.0001 | <0.0001 | -0.0247 (-0.165-0.089) | -0.00618 (-0.151-0.129) | <0.0001 | <0.0001 |  |
| Midazolam | Increase | 21.4 (7.14-36) | 24.1 (7.69-41.4) | <0.0001 | <0.0001 | 13.3 (2.04-26.6) | 15.4 (3.45-32.1) | <0.0001 | <0.0001 | -0.0207 (-0.164-0.092) | -0.00632 (-0.154-0.127) | <0.0001 | <0.0001 |  |
| Morphine | Decrease | 21.8 (7.69-35.7) | 25 (10-41.7) | <0.0001 | <0.0001 | 13.8 (3.45-26.8) | 16.1 (3.57-32.1) | <0.0001 | <0.0001 | -0.0204 (-0.156-0.0984) | 0.00333 (-0.135-0.141) | <0.0001 | <0.0001 |  |
| Morphine | Increase | 20.8 (7.14-35.7) | 25 (9.38-41.4) | <0.0001 | <0.0001 | 12 (0-25.8) | 16 (3.57-32.1) | <0.0001 | <0.0001 | -0.0222 (-0.163-0.0984) | 0.00209 (-0.141-0.139) | <0.0001 | <0.0001 |  |
| Noradrenaline | Decrease | 21.3 (7.41-35.7) | 24.1 (8-41.4) | <0.0001 | <0.0001 | 13 (2.56-25.9) | 15.4 (3.45-32) | <0.0001 | <0.0001 | -0.0282 (-0.167-0.0892) | -0.00431 (-0.152-0.13) | <0.0001 | <0.0001 |  |
| Noradrenaline | Increase | 20.8 (7.14-35.7) | 24.1 (8.33-41.4) | <0.0001 | <0.0001 | 12.5 (0-26.1) | 15.4 (3.45-32.1) | <0.0001 | <0.0001 | -0.0273 (-0.167-0.0916) | -0.0097 (-0.157-0.127) | <0.0001 | <0.0001 |  |
| Propofol | Decrease | 21.4 (7.69-36) | 25 (10-42.3) | <0.0001 | <0.0001 | 13.6 (3.33-26.7) | 16.7 (3.57-33.3) | <0.0001 | <0.0001 | -0.0216 (-0.163-0.0984) | 0.0063 (-0.14-0.149) | <0.0001 | <0.0001 |  |
| Propofol | Increase | 21.4 (7.41-35.7) | 24.1 (9.68-41.4) | <0.0001 | <0.0001 | 13 (2.38-25.9) | 16 (3.57-32.1) | <0.0001 | <0.0001 | -0.0223 (-0.16-0.0993) | 0.000323 (-0.145-0.14) | <0.0001 | <0.0001 |  |
| Vasopressin | Decrease | 30.5 (28.3-30.6) | 29.6 (27.8-29.9) | 0.0986 | 1 | 23.4 (21.1-24.1) | 22.3 (20.5-23) | 0.0706 | 1 | 0.0375 (0.017-0.0647) | 0.0248 (0.017-0.0429) | 0.451 | 1 |  |
| Vasopressin | Increase | 29.6 (11.1-30.9) | 28.3 (15.5-30.5) | 0.638 | 1 | 22.3 (5.17-26.3) | 21.1 (10.7-24.1) | 0.621 | 1 | 0.0248 (-0.0417-0.0647) | 0.0171 (-0.0591-0.0429) | 0.419 | 1 |  |
| **Name** | **Mean Dose Change** | **% time LPRx_15 > 0** | | | | **% time LPRx_15 > 0.25** | | | | **% time LPRx_15 > 0.35** | | | |  |
|  |  | **Pre Dose** | **Post Dose** | **P value** | **Adj P Value** | **Pre Dose** | **Post Dose** | **P value** | **Adj P Value** | **Pre Dose** | **Post Dose** | **P value** | **Adj P Value** |  |
| Dobutamine | Decrease | 45.5 (24.1-64.3) | 50 (28-69.8) | 0.000274 | 0.0074 | 16.7 (0-34.5) | 20.8 (3.57-40.7) | <0.0001 | 0.000197 | 8 (0-22.2) | 11.5 (0-29.6) | <0.0001 | <0.0001 |  |
| Dobutamine | Increase | 45.3 (25-65) | 48.1 (25.7-70.4) | 0.00625 | 0.169 | 16 (2.17-32) | 20 (3.7-40) | <0.0001 | 0.000774 | 7.41 (0-21.7) | 11.1 (0-27.6) | <0.0001 | 0.000302 |  |
| Midazolam | Decrease | 46.4 (26.7-63) | 49.7 (27.4-68) | <0.0001 | <0.0001 | 14.8 (0-32) | 17.9 (2.4-36.7) | <0.0001 | <0.0001 | 6.9 (0-20) | 8 (0-25.9) | <0.0001 | <0.0001 |  |
| Midazolam | Increase | 46.4 (25-63) | 50 (27.6-67.9) | <0.0001 | <0.0001 | 14.8 (0-30.8) | 17.9 (2.33-37) | <0.0001 | <0.0001 | 6.67 (0-20) | 8 (0-25.9) | <0.0001 | <0.0001 |  |
| Morphine | Decrease | 47.8 (27.6-64.3) | 50 (30.8-69) | <0.0001 | <0.0001 | 16.3 (1.1-31.5) | 19.4 (3.57-38.5) | <0.0001 | <0.0001 | 7.14 (0-20.8) | 10.3 (0-27.6) | <0.0001 | <0.0001 |  |
| Morphine | Increase | 46.7 (25.9-65.4) | 50 (29.6-69) | <0.0001 | <0.0001 | 14.8 (0-31) | 19.4 (3.57-38.5) | <0.0001 | <0.0001 | 6.82 (0-20.7) | 10.3 (0-27.3) | <0.0001 | <0.0001 |  |
| Noradrenaline | Decrease | 45.7 (25.9-63) | 50 (28-68) | <0.0001 | <0.0001 | 14.8 (0-30.8) | 17.9 (2.7-37) | <0.0001 | <0.0001 | 6.67 (0-20.7) | 8 (0-25.9) | <0.0001 | <0.0001 |  |
| Noradrenaline | Increase | 46.2 (25.9-63.6) | 48.3 (27.6-67.9) | <0.0001 | <0.0001 | 14.8 (0-31) | 17.9 (2.5-36.8) | <0.0001 | <0.0001 | 6.25 (0-20.7) | 7.69 (0-25.9) | <0.0001 | <0.0001 |  |
| Propofol | Decrease | 46.4 (27.6-64.3) | 50 (30.4-70.1) | <0.0001 | <0.0001 | 16 (2.33-32.1) | 20 (3.57-40) | <0.0001 | <0.0001 | 7.14 (0-21.4) | 10.3 (0-28.6) | <0.0001 | <0.0001 |  |
| Propofol | Increase | 46.4 (26.7-64.3) | 50 (29.6-69.2) | <0.0001 | <0.0001 | 15 (0-32.1) | 19.2 (3.57-38.7) | <0.0001 | <0.0001 | 6.9 (0-21.4) | 10.3 (0-27.6) | <0.0001 | <0.0001 |  |
| Vasopressin | Decrease | 53.4 (50-56.7) | 53.4 (52.1-54.8) | 0.834 | 1 | 27.6 (25.1-30.3) | 26.7 (24.9-28.2) | 0.288 | 1 | 18.9 (16.3-22) | 17.8 (16.3-19.7) | 0.462 | 1 |  |
| Vasopressin | Increase | 53.4 (42.2-60) | 52.3 (44.6-54.8) | 0.166 | 1 | 26.7 (1.61-30.3) | 25.1 (8.17-28.2) | 0.64 | 1 | 17.8 (0-20.7) | 16.5 (3.85-19.7) | 0.534 | 1 |  |
| **Name** | **Mean Dose Change** | **Mean LPRx_20** | | | | **% time LPRx_20 > 0** | | | | **% time LPRx_20 > 0.25** | | | |  |
|  |  | **Pre Dose** | **Post Dose** | **P value** | **Adj P Value** | **Pre Dose** | **Post Dose** | **P value** | **Adj P Value** | **Pre Dose** | **Post Dose** | **P value** | **Adj P Value** |  |
| Dobutamine | Decrease | -0.0279 (-0.184-0.11) | -0.00218 (-0.171-0.142) | 0.00454 | 0.123 | 44.8 (20.8-67.9) | 48.3 (22.7-71.4) | 0.0169 | 0.457 | 12.5 (0-33.3) | 16.7 (0-38.5) | 0.00243 | 0.0656 |  |
| Dobutamine | Increase | -0.0243 (-0.173-0.111) | -0.00544 (-0.175-0.142) | 0.0323 | 0.871 | 46.9 (20-68) | 48.3 (22.8-71.7) | 0.0601 | 1 | 12 (0-32) | 16.7 (0-37.1) | 0.00316 | 0.0853 |  |
| Midazolam | Decrease | -0.0245 (-0.155-0.0808) | -0.00876 (-0.142-0.119) | <0.0001 | <0.0001 | 46.2 (24-65.4) | 48.1 (25-70.4) | <0.0001 | 0.000404 | 10 (0-28) | 12.5 (0-33.3) | <0.0001 | <0.0001 |  |
| Midazolam | Increase | -0.0199 (-0.158-0.0861) | -0.00446 (-0.145-0.123) | <0.0001 | <0.0001 | 46.4 (22.2-66.7) | 48.9 (25.9-70) | <0.0001 | 0.000158 | 10 (0-28.2) | 12.9 (0-34.5) | <0.0001 | <0.0001 |  |
| Morphine | Decrease | -0.0173 (-0.149-0.0968) | 0.00227 (-0.128-0.137) | <0.0001 | <0.0001 | 46.7 (25.8-67.7) | 50 (28.6-71.4) | <0.0001 | <0.0001 | 11.5 (0-29.2) | 15 (0-36.9) | <0.0001 | <0.0001 |  |
| Morphine | Increase | -0.0192 (-0.156-0.0987) | 0.00375 (-0.137-0.136) | <0.0001 | <0.0001 | 46.9 (23.5-68.4) | 50 (28.6-70.8) | <0.0001 | <0.0001 | 10.3 (0-29) | 14.8 (0-36.8) | <0.0001 | <0.0001 |  |
| Noradrenaline | Decrease | -0.0278 (-0.163-0.089) | -0.00817 (-0.148-0.122) | <0.0001 | <0.0001 | 44.8 (22.2-65.5) | 48.3 (25-70) | <0.0001 | <0.0001 | 10 (0-28.6) | 12.8 (0-34.5) | <0.0001 | <0.0001 |  |
| Noradrenaline | Increase | -0.0251 (-0.16-0.0902) | -0.0137 (-0.154-0.117) | <0.0001 | <0.0001 | 45.8 (23.1-66.7) | 48.1 (24.1-69) | <0.0001 | <0.0001 | 10 (0-28.6) | 12 (0-33.3) | <0.0001 | <0.0001 |  |
| Propofol | Decrease | -0.0177 (-0.156-0.1) | 0.00784 (-0.13-0.148) | <0.0001 | <0.0001 | 46.4 (25-67.9) | 51.7 (28.6-73.1) | <0.0001 | <0.0001 | 11.5 (0-30.8) | 15.9 (0-39.1) | <0.0001 | <0.0001 |  |
| Propofol | Increase | -0.0193 (-0.155-0.0999) | 0.00485 (-0.135-0.142) | <0.0001 | <0.0001 | 46.4 (24-68) | 50 (28.6-72) | <0.0001 | <0.0001 | 10.7 (0-30) | 15.4 (0-37.9) | <0.0001 | <0.0001 |  |
| Vasopressin | Decrease | 0.0367 (0.0194-0.0627) | 0.0278 (0.021-0.0517) | 0.761 | 1 | 53.8 (51.6-58.2) | 52.6 (51.6-54.8) | 0.924 | 1 | 25 (22.4-29) | 24.1 (22.4-26.9) | 0.58 | 1 |  |
| Vasopressin | Increase | 0.0278 (-0.0435-0.0601) | 0.0236 (-0.0447-0.0394) | 0.483 | 1 | 53.8 (38.3-58.3) | 52.6 (37.9-55.7) | 0.38 | 1 | 24.1 (3.23-27.3) | 22.4 (3.61-26.9) | 0.448 | 1 |  |
| **Name** | **Mean Dose Change** | **% time LPRx_20 > 0.35** | | | | **Mean LPRx_30** | | | | **% time LPRx_30 > 0** | | | |  |
|  |  | **Pre Dose** | **Post Dose** | **P value** | **Adj P Value** | **Pre Dose** | **Post Dose** | **P value** | **Adj P Value** | **Pre Dose** | **Post Dose** | **P value** | **Adj P Value** |  |
| Dobutamine | Decrease | 3.57 (0-20) | 6.9 (0-26.1) | 0.000165 | 0.00444 | -0.033 (-0.18-0.131) | -0.0254 (-0.188-0.134) | 0.663 | 1 | 44.4 (14.8-73.1) | 45.8 (16-75) | 0.386 | 1 |  |
| Dobutamine | Increase | 3.45 (0-19.2) | 6.45 (0-25.9) | 0.000146 | 0.00395 | -0.0164 (-0.167-0.121) | -0.0232 (-0.185-0.134) | 0.82 | 1 | 46.9 (16.7-75.8) | 45 (15.5-75.9) | 0.579 | 1 |  |
| Midazolam | Decrease | 0 (0-16.1) | 3.45 (0-20.7) | <0.0001 | <0.0001 | -0.0184 (-0.153-0.094) | -0.00382 (-0.143-0.119) | <0.0001 | 0.00114 | 46.2 (17.9-71.7) | 48.1 (20-75) | 0.000768 | 0.0207 |  |
| Midazolam | Increase | 0 (0-16.1) | 3.45 (0-21.4) | <0.0001 | <0.0001 | -0.0189 (-0.149-0.098) | -0.00353 (-0.14-0.122) | 0.00079 | 0.0213 | 45.8 (17.2-72.7) | 48.3 (19.2-75.7) | 0.00543 | 0.147 |  |
| Morphine | Decrease | 3.23 (0-17.9) | 4 (0-23.3) | <0.0001 | <0.0001 | -0.00562 (-0.141-0.115) | 0.00501 (-0.127-0.141) | <0.0001 | <0.0001 | 49.4 (21.4-73.9) | 50 (22.2-77.4) | 0.000142 | 0.00384 |  |
| Morphine | Increase | 0 (0-16.8) | 4 (0-24) | <0.0001 | <0.0001 | -0.0073 (-0.148-0.116) | 0.00446 (-0.128-0.141) | <0.0001 | <0.0001 | 48.3 (18.5-76) | 50 (23.1-76.9) | <0.0001 | 0.00254 |  |
| Noradrenaline | Decrease | 0 (0-16.7) | 3.45 (0-21.7) | <0.0001 | <0.0001 | -0.0207 (-0.156-0.0997) | -0.0101 (-0.145-0.119) | <0.0001 | <0.0001 | 45.8 (17.5-72) | 48 (19.2-75) | <0.0001 | <0.0001 |  |
| Noradrenaline | Increase | 0 (0-16.7) | 3.45 (0-21.4) | <0.0001 | <0.0001 | -0.0183 (-0.152-0.102) | -0.0133 (-0.146-0.113) | <0.0001 | <0.0001 | 46.4 (17.9-73.1) | 46.4 (18.5-73.3) | 0.0209 | 0.564 |  |
| Propofol | Decrease | 3.05 (0-18.5) | 4.65 (0-25.9) | <0.0001 | <0.0001 | -0.0103 (-0.145-0.12) | 0.0136 (-0.121-0.161) | <0.0001 | <0.0001 | 48.1 (20.5-75) | 53.3 (25-80.8) | <0.0001 | <0.0001 |  |
| Propofol | Increase | 1.75 (0-17.9) | 4.17 (0-25.8) | <0.0001 | <0.0001 | -0.0116 (-0.148-0.119) | 0.0113 (-0.121-0.154) | <0.0001 | <0.0001 | 48 (20-75) | 52.5 (24.4-79.3) | <0.0001 | <0.0001 |  |
| Vasopressin | Decrease | 15.2 (10.4-19.1) | 15.2 (13.9-18) | 0.59 | 1 | 0.0267 (-0.0291-0.0357) | 0.0267 (0.0245-0.0534) | 0.481 | 1 | 51.6 (36.3-53.8) | 51.6 (51.5-55.5) | 0.404 | 1 |  |
| Vasopressin | Increase | 15.2 (0-18.8) | 14.1 (0-17.2) | 0.32 | 1 | 0.0267 (-0.0614-0.0504) | 0.0267 (-0.0731-0.0427) | 0.655 | 1 | 51.6 (32.8-59.1) | 51.6 (29.3-54.4) | 0.398 | 1 |  |
| **Name** | **Mean Dose Change** | **% time LPRx_30 > 0.25** | | | | **% time LPRx_30 > 0.35** | | | | **Mean LPRx_60** | | | |  |
|  |  | **Pre Dose** | **Post Dose** | **P value** | **Adj P Value** | **Pre Dose** | **Post Dose** | **P value** | **Adj P Value** | **Pre Dose** | **Post Dose** | **P value** | **Adj P Value** |  |
| Dobutamine | Decrease | 5.26 (0-32.1) | 7.41 (0-34.1) | 0.382 | 1 | 0 (0-19.2) | 0 (0-18.5) | 0.232 | 1 | -0.0113 (-0.185-0.174) | -0.0139 (-0.218-0.156) | 0.251 | 1 |  |
| Dobutamine | Increase | 6.86 (0-33.3) | 7.41 (0-35.9) | 0.872 | 1 | 0 (0-17.9) | 0 (0-23) | 0.0765 | 1 | 0.00181 (-0.168-0.189) | -0.0215 (-0.187-0.149) | 0.0179 | 0.483 |  |
| Midazolam | Decrease | 0 (0-25) | 3.7 (0-30.6) | <0.0001 | 0.000303 | 0 (0-10.7) | 0 (0-14.8) | <0.0001 | <0.0001 | 0.000305 (-0.137-0.146) | 0.0073 (-0.126-0.14) | 0.184 | 1 |  |
| Midazolam | Increase | 0 (0-25) | 3.7 (0-30) | <0.0001 | 0.000544 | 0 (0-11.1) | 0 (0-15.1) | <0.0001 | 0.000208 | -0.00237 (-0.138-0.148) | 0.00576 (-0.127-0.144) | 0.263 | 1 |  |
| Morphine | Decrease | 4 (0-29) | 7.18 (0-34.8) | <0.0001 | <0.0001 | 0 (0-14.8) | 0 (0-19.4) | <0.0001 | <0.0001 | 0.0128 (-0.136-0.171) | 0.0159 (-0.124-0.168) | 0.262 | 1 |  |
| Morphine | Increase | 3.45 (0-28.6) | 7.14 (0-35.7) | <0.0001 | <0.0001 | 0 (0-13) | 0 (0-20) | <0.0001 | <0.0001 | 0.0105 (-0.139-0.169) | 0.0142 (-0.125-0.171) | 0.224 | 1 |  |
| Noradrenaline | Decrease | 3.33 (0-25.9) | 3.45 (0-30.4) | <0.0001 | <0.0001 | 0 (0-11.9) | 0 (0-14.8) | <0.0001 | <0.0001 | -0.000558 (-0.143-0.146) | -0.0027 (-0.141-0.138) | 0.131 | 1 |  |
| Noradrenaline | Increase | 2.94 (0-26.1) | 3.45 (0-29.6) | <0.0001 | <0.0001 | 0 (0-12) | 0 (0-14.8) | <0.0001 | <0.0001 | 0.000767 (-0.139-0.145) | -0.000934 (-0.136-0.14) | 0.26 | 1 |  |
| Propofol | Decrease | 4.17 (0-30.8) | 7.69 (0-38.5) | <0.0001 | <0.0001 | 0 (0-15.4) | 0 (0-22.2) | <0.0001 | <0.0001 | 0.0142 (-0.132-0.172) | 0.0238 (-0.113-0.188) | <0.0001 | <0.0001 |  |
| Propofol | Increase | 3.77 (0-29.2) | 7.69 (0-38.1) | <0.0001 | <0.0001 | 0 (0-14.3) | 0 (0-22.2) | <0.0001 | <0.0001 | 0.0142 (-0.131-0.171) | 0.0226 (-0.112-0.183) | <0.0001 | 0.00215 |  |
| Vasopressin | Decrease | 20.6 (10.6-21.9) | 20.6 (18.8-24) | 0.996 | 1 | 12.4 (5.08-15.5) | 12.1 (1.92-13.7) | 0.302 | 1 | 0.0102 (-0.000735-0.0297) | 0.0215 (-0.0206-0.0398) | 0.755 | 1 |  |
| Vasopressin | Increase | 20.6 (0-21.9) | 19.7 (1.61-20.6) | 0.58 | 1 | 12.4 (0-13.9) | 11.9 (0-12.9) | 0.476 | 1 | 0.01 (-0.0836-0.0215) | 0.01 (-0.0911-0.0398) | 0.402 | 1 |  |
| **Name** | **Mean Dose Change** | **% time LPRx_60 > 0** | | | | **% time LPRx_60 > 0.25** | | | | **% time LPRx_60 > 0.35** | | | |  |
|  |  | **Pre Dose** | **Post Dose** | **P value** | **Adj P Value** | **Pre Dose** | **Post Dose** | **P value** | **Adj P Value** | **Pre Dose** | **Post Dose** | **P value** | **Adj P Value** |  |
| Dobutamine | Decrease | 48 (3.57-92) | 44.8 (0-96.3) | 0.433 | 1 | 0 (0-34.6) | 0 (0-31) | 0.0661 | 1 | 0 (0-14) | 0 (0-7.41) | 0.236 | 1 |  |
| Dobutamine | Increase | 52 (7.43-96.6) | 42 (0-93.3) | 0.00426 | 0.115 | 0 (0-38.4) | 0 (0-25) | 0.00173 | 0.0468 | 0 (0-14.7) | 0 (0-4.13) | 0.0105 | 0.284 |  |
| Midazolam | Decrease | 50 (4.47-92.9) | 51.9 (7.14-95.8) | 0.164 | 1 | 0 (0-26.6) | 0 (0-25.1) | 0.273 | 1 | 0 (0-6.84) | 0 (0-3.85) | 0.109 | 1 |  |
| Midazolam | Increase | 50 (3.85-95.8) | 50 (6.67-96.2) | 0.265 | 1 | 0 (0-26.9) | 0 (0-25) | 0.25 | 1 | 0 (0-6.9) | 0 (0-3.45) | 0.0659 | 1 |  |
| Morphine | Decrease | 53.6 (7.69-96.3) | 54.6 (8.7-100) | 0.197 | 1 | 0 (0-33.3) | 0 (0-32.2) | 0.322 | 1 | 0 (0-14.5) | 0 (0-11.4) | 0.0747 | 1 |  |
| Morphine | Increase | 53.6 (6.9-100) | 53.8 (6.9-100) | 0.546 | 1 | 0 (0-31.9) | 0 (0-32.1) | 0.602 | 1 | 0 (0-10.8) | 0 (0-10.3) | 0.303 | 1 |  |
| Noradrenaline | Decrease | 50 (5.56-92.9) | 48.3 (0-96) | 0.00468 | 0.126 | 0 (0-27.3) | 0 (0-23.1) | <0.0001 | <0.0001 | 0 (0-7.41) | 0 (0-2.5) | <0.0001 | <0.0001 |  |
| Noradrenaline | Increase | 50 (6.67-93.1) | 50 (0-96.3) | 0.00493 | 0.133 | 0 (0-26.2) | 0 (0-22.6) | <0.0001 | <0.0001 | 0 (0-7.14) | 0 (0-0) | <0.0001 | <0.0001 |  |
| Propofol | Decrease | 54.3 (8.94-96.6) | 58.3 (10.7-100) | <0.0001 | 0.000482 | 0 (0-34.6) | 0 (0-37.2) | 0.807 | 1 | 0 (0-12.4) | 0 (0-13.3) | 0.646 | 1 |  |
| Propofol | Increase | 53.8 (9.09-96.7) | 57.9 (10.3-100) | 0.000357 | 0.00964 | 0 (0-33.3) | 0 (0-35.7) | 0.353 | 1 | 0 (0-12) | 0 (0-11.1) | 0.306 | 1 |  |
| Vasopressin | Decrease | 50.2 (45.3-61.9) | 51.5 (48.3-54.3) | 0.853 | 1 | 14.8 (0-15.2) | 14.5 (0-16.4) | 0.794 | 1 | 9.67 (0-9.94) | 9.66 (0-10.8) | 0.784 | 1 |  |
| Vasopressin | Increase | 49.9 (3.33-51.5) | 49.9 (3.23-54.3) | 0.632 | 1 | 14.5 (0-15.2) | 14.5 (0-16.4) | 0.964 | 1 | 9.66 (0-9.94) | 9.66 (0-10.4) | 0.713 | 1 |  |
| **Bolus** | | | | | | | | | | | | | |  |
| **Name** | **Doses** | **Mean MAP** | | | | **Mean CPP** | | | | **% time CPP>60** | | | |  |
|  |  | **Pre Dose** | **Post Dose** | **P value** | **Adj P Value** | **Pre Dose** | **Post Dose** | **P value** | **Adj P Value** | **Pre Dose** | **Post Dose** | **P value** | **Adj P Value** |  |
| Ephedrine | 36 | 71.8 (63.8-83.9) | 75.6 (65.3-84.4) | 0.474 | 1 | 57.9 (54.9-74.2) | 60 (55.8-76) | 0.562 | 1 | 70 (4.43-100) | 59.2 (3.91-100) | 0.846 | 1 |  |
| Fentanyl | 198 | 80.8 (75.8-89.2) | 80.2 (72.5-87.5) | 0.0967 | 1 | 69.2 (62.4-77.5) | 67.9 (61.4-75.6) | 0.323 | 1 | 7.02 (0-33.5) | 11.5 (0-37.1) | 0.326 | 1 |  |
| Midazolam | 990 | 79.3 (75-85.6) | 78.9 (74.2-84.9) | 0.132 | 1 | 63.8 (58.9-69.6) | 63.7 (58.3-69) | 0.475 | 1 | 20.2 (0-65.2) | 20 (0-70.7) | 0.965 | 1 |  |
| Morphine | 4042 | 81.6 (75.3-90.4) | 80.3 (74.1-88.8) | <0.0001 | <0.0001 | 68.8 (62.3-78.7) | 67.3 (61-76.2) | <0.0001 | <0.0001 | 2.74 (0-32.2) | 4.35 (0-44.8) | <0.0001 | <0.0001 |  |
| Propofol | 4262 | 80.3 (74.8-86.5) | 79.2 (73.8-85.6) | <0.0001 | <0.0001 | 66 (60.4-72.9) | 65 (59.7-71.6) | <0.0001 | 0.00223 | 8.16 (0-50) | 12 (0-57.1) | 0.00171 | 0.0462 |  |
| Alfentanil | 352 | 78.8 (72.6-85.8) | 77.4 (71.8-83.6) | 0.113 | 1 | 66.7 (60.6-74.2) | 65.8 (60.8-73.7) | 0.581 | 1 | 11.5 (0-46.2) | 14.8 (0-48.5) | 0.947 | 1 |  |
| **Name** | **% time CPP>70** | | | | **Mean ICP** | | | | **% time ICP>20** | | | |  |  |
|  | **Pre Dose** | **Post Dose** | **P value** | **Adj P Value** | **Pre Dose** | **Post Dose** | **P value** | **Adj P Value** | **Pre Dose** | **Post Dose** | **P value** | **Adj P Value** |  |  |
| Ephedrine | 0 (0-58.9) | 1.16 (0-76.3) | 0.438 | 1 | 11.1 (5.64-14.4) | 9.47 (6.26-15) | 0.714 | 1 | 0 (0-2.44) | 0 (0-2.87) | 0.735 | 1 |  |  |
| Fentanyl | 33.6 (11.1-93.9) | 32 (2.68-80) | 0.0487 | 1 | 12.3 (7.55-15.7) | 11.7 (8-15.1) | 0.258 | 1 | 0 (0-10.4) | 0 (0-7.14) | 0.481 | 1 |  |  |
| Midazolam | 6.59 (0-41.7) | 3.85 (0-37.7) | 0.101 | 1 | 16.4 (12.6-19.3) | 15.9 (12.3-19.1) | 0.132 | 1 | 4.17 (0-27.3) | 3.7 (0-24.1) | 0.113 | 1 |  |  |
| Morphine | 31.6 (2.86-96) | 23.1 (0-88) | <0.0001 | <0.0001 | 12.4 (8.73-16.5) | 12.7 (8.88-16.6) | 0.125 | 1 | 0 (0-6.9) | 0 (0-6.95) | 0.95 | 1 |  |  |
| Propofol | 13.8 (0-69.2) | 8.33 (0-63) | <0.0001 | <0.0001 | 14.2 (10.1-18.1) | 14 (9.78-18) | 0.232 | 1 | 0 (0-14.3) | 0 (0-13) | 0.00192 | 0.0519 |  |  |
| Alfentanil | 18.5 (3.45-72) | 14.8 (0-72.8) | 0.506 | 1 | 11.6 (7.81-15) | 10.8 (7.32-14) | 0.112 | 1 | 0 (0-5.56) | 0 (0-3.33) | 0.00405 | 0.109 |  |  |
| **Name** | **% time ICP>22** | | | | **Mean LPRx_10** | | | | **% time LPRx_10 > 0** | | | |  |  |
|  | **Pre Dose** | **Post Dose** | **P value** | **Adj P Value** | **Pre Dose** | **Post Dose** | **P value** | **Adj P Value** | **Pre Dose** | **Post Dose** | **P value** | **Adj P Value** |  |  |
| Ephedrine | 0 (0-0.122) | 0 (0-0.114) | 0.334 | 1 | 0.00775 (-0.118-0.0276) | -0.013 (-0.241-0.079) | 0.42 | 1 | 51.3 (38.8-57.1) | 45.9 (28.3-54.5) | 0.316 | 1 |  |  |
| Fentanyl | 0 (0-3.7) | 0 (0-3.67) | 0.359 | 1 | -0.00213 (-0.123-0.11) | 0.0164 (-0.156-0.146) | 0.378 | 1 | 50.7 (32-64) | 55.6 (32-70.8) | 0.13 | 1 |  |  |
| Midazolam | 0 (0-8.33) | 0 (0-7.69) | 0.221 | 1 | -0.0198 (-0.158-0.0886) | 0.0067 (-0.133-0.135) | <0.0001 | 0.00133 | 47.5 (30.1-61.5) | 50 (33.3-66.7) | 0.000164 | 0.00442 |  |  |
| Morphine | 0 (0-0.61) | 0 (0-0.838) | 0.445 | 1 | 0.0015 (-0.128-0.113) | 0.0214 (-0.112-0.154) | <0.0001 | <0.0001 | 50 (33.3-64.3) | 51.9 (36-69) | <0.0001 | <0.0001 |  |  |
| Propofol | 0 (0-4.35) | 0 (0-3.85) | 0.000846 | 0.0228 | -0.00981 (-0.147-0.111) | 0.012 (-0.137-0.149) | <0.0001 | <0.0001 | 48.1 (31-63.6) | 51.2 (33.3-68.5) | <0.0001 | <0.0001 |  |  |
| Alfentanil | 0 (0-3.57) | 0 (0-0) | 0.00911 | 0.246 | -0.0133 (-0.159-0.108) | 0.00252 (-0.147-0.139) | 0.0694 | 1 | 46.2 (30.7-63) | 50 (32-66.7) | 0.0632 | 1 |  |  |
| **Name** | **% time LPRx_10 > 0.25** | | | | **% time LPRx_10 > 0.35** | | | | **Mean LPRx_15** | | | |  |  |
|  | **Pre Dose** | **Post Dose** | **P value** | **Adj P Value** | **Pre Dose** | **Post Dose** | **P value** | **Adj P Value** | **Pre Dose** | **Post Dose** | **P value** | **Adj P Value** |  |  |
| Ephedrine | 25.4 (10.4-32.7) | 22.9 (3.81-33.1) | 0.817 | 1 | 17.6 (3.78-26.3) | 16.7 (2.42-25.7) | 0.659 | 1 | 0.0208 (-0.119-0.0806) | -0.0152 (-0.23-0.0467) | 0.267 | 1 |  |  |
| Fentanyl | 25 (9.3-37) | 25.9 (11.1-43.5) | 0.16 | 1 | 16.7 (3.64-26.5) | 17.8 (3.85-30) | 0.221 | 1 | 0.00301 (-0.107-0.094) | -0.00843 (-0.135-0.161) | 0.988 | 1 |  |  |
| Midazolam | 22.2 (7.69-34.6) | 25.9 (10.7-40.7) | <0.0001 | <0.0001 | 12.5 (2.83-25) | 17.2 (4-31.4) | <0.0001 | <0.0001 | -0.0232 (-0.151-0.0908) | 0.00576 (-0.111-0.127) | <0.0001 | 0.000412 |  |  |
| Morphine | 23.3 (9.3-37) | 27.2 (12-42.3) | <0.0001 | <0.0001 | 14.8 (3.7-26.9) | 18.5 (4.55-32.5) | <0.0001 | <0.0001 | 0.00209 (-0.122-0.109) | 0.0205 (-0.112-0.15) | <0.0001 | <0.0001 |  |  |
| Propofol | 21.7 (7.69-36) | 25.6 (10.7-41.4) | <0.0001 | <0.0001 | 13.7 (3.45-26.2) | 16.7 (4-32.1) | <0.0001 | <0.0001 | -0.0092 (-0.137-0.111) | 0.0159 (-0.125-0.15) | <0.0001 | <0.0001 |  |  |
| Alfentanil | 22.2 (7.69-35.7) | 26.9 (11-40.8) | 0.0145 | 0.391 | 12.2 (3.45-24.2) | 19.2 (4.35-31) | 0.000165 | 0.00445 | -0.00856 (-0.145-0.0992) | 0.0128 (-0.113-0.135) | 0.0283 | 0.765 |  |  |
| **Name** | **% time LPRx_15 > 0** | | | | **% time LPRx_15 > 0.25** | | | | **% time LPRx_15 > 0.35** | | | |  |  |
|  | **Pre Dose** | **Post Dose** | **P value** | **Adj P Value** | **Pre Dose** | **Post Dose** | **P value** | **Adj P Value** | **Pre Dose** | **Post Dose** | **P value** | **Adj P Value** |  |  |
| Ephedrine | 50.7 (29.4-61.4) | 46.7 (25-51.9) | 0.284 | 1 | 23.2 (3.85-30.8) | 20.1 (6.47-31.8) | 0.73 | 1 | 8.01 (0-23.3) | 14.8 (0-23.1) | 0.909 | 1 |  |  |
| Fentanyl | 51.7 (32.1-66.7) | 50 (30.8-71.6) | 0.641 | 1 | 19.2 (3.85-33.1) | 20 (3.85-40) | 0.153 | 1 | 10.3 (0-20.8) | 10.5 (0-27) | 0.347 | 1 |  |  |
| Midazolam | 46.2 (28-62.8) | 51.6 (32.5-69.2) | <0.0001 | 0.00128 | 15.4 (0-30.2) | 21.6 (5.34-37.8) | <0.0001 | <0.0001 | 6.52 (0-20.6) | 11.5 (0-25.4) | <0.0001 | <0.0001 |  |  |
| Morphine | 50 (30.8-66.7) | 52.4 (33.3-71.7) | <0.0001 | <0.0001 | 19.2 (3.85-33.3) | 23 (6.28-40.5) | <0.0001 | <0.0001 | 8.33 (0-22.2) | 12.9 (0-28.6) | <0.0001 | <0.0001 |  |  |
| Propofol | 48.3 (29.6-66.7) | 51.9 (32.1-71.4) | <0.0001 | <0.0001 | 17 (2.88-33.3) | 21.4 (4-40.4) | <0.0001 | <0.0001 | 7.41 (0-21) | 11.1 (0-28.6) | <0.0001 | <0.0001 |  |  |
| Alfentanil | 48.1 (29.5-66.7) | 52.2 (32.1-70.1) | 0.0431 | 1 | 14.6 (3.33-31) | 22.4 (3.57-37.6) | 0.00268 | 0.0723 | 6.63 (0-18.9) | 13.1 (0-26) | <0.0001 | 0.00261 |  |  |
| **Name** | **Mean LPRx_20** | | | | **% time LPRx_20 > 0** | | | | **% time LPRx_20 > 0.25** | | | |  |  |
|  | **Pre Dose** | **Post Dose** | **P value** | **Adj P Value** | **Pre Dose** | **Post Dose** | **P value** | **Adj P Value** | **Pre Dose** | **Post Dose** | **P value** | **Adj P Value** |  |  |
| Ephedrine | 0.0288 (-0.108-0.0939) | -0.0442 (-0.217-0.0265) | 0.157 | 1 | 51.7 (31.7-64.7) | 40 (24.3-53.3) | 0.226 | 1 | 22 (0-29.8) | 16.5 (1.7-29.6) | 0.488 | 1 |  |  |
| Fentanyl | 0.0132 (-0.114-0.102) | -0.0141 (-0.133-0.152) | 0.801 | 1 | 52 (29.2-66.7) | 50 (30.8-68) | 0.712 | 1 | 16.6 (0-29.6) | 14.1 (0-35.1) | 0.606 | 1 |  |  |
| Midazolam | -0.0139 (-0.15-0.094) | 0.0167 (-0.102-0.129) | <0.0001 | 0.000394 | 46.4 (25.8-66.7) | 51.9 (32.1-70.4) | 0.000254 | 0.00685 | 11.8 (0-29.6) | 18.5 (0-37.4) | <0.0001 | 0.000129 |  |  |
| Morphine | 0.00419 (-0.125-0.115) | 0.0204 (-0.114-0.156) | <0.0001 | <0.0001 | 50 (28.6-69.8) | 53.3 (30.8-75.6) | <0.0001 | <0.0001 | 15 (0-32) | 20 (0-40) | <0.0001 | <0.0001 |  |  |
| Propofol | -0.00381 (-0.13-0.112) | 0.0206 (-0.11-0.154) | <0.0001 | <0.0001 | 50 (26.9-70.4) | 53.3 (31-75) | <0.0001 | <0.0001 | 13.1 (0-32) | 17.9 (0-39.3) | <0.0001 | <0.0001 |  |  |
| Alfentanil | -0.0102 (-0.121-0.11) | 0.0224 (-0.0993-0.142) | 0.0236 | 0.638 | 48.1 (26.9-69.3) | 53.7 (32.2-73.5) | 0.0253 | 0.683 | 10.3 (0-32.2) | 19.3 (0-36) | 0.000725 | 0.0196 |  |  |
| **Name** | **% time LPRx_20 > 0.35** | | | | **Mean LPRx_30** | | | | **% time LPRx_30 > 0** | | | |  |  |
|  | **Pre Dose** | **Post Dose** | **P value** | **Adj P Value** | **Pre Dose** | **Post Dose** | **P value** | **Adj P Value** | **Pre Dose** | **Post Dose** | **P value** | **Adj P Value** |  |  |
| Ephedrine | 6.01 (0-21.7) | 11 (0-21.6) | 0.893 | 1 | -0.0159 (-0.0943-0.0817) | -0.0196 (-0.137-0.0321) | 0.517 | 1 | 43 (22.9-57) | 52.5 (30.5-61.4) | 0.532 | 1 |  |  |
| Fentanyl | 6.9 (0-17.2) | 6.9 (0-24.8) | 0.26 | 1 | -0.00438 (-0.0919-0.127) | 0.00297 (-0.119-0.148) | 0.429 | 1 | 48.3 (23.4-70.4) | 50 (27.1-72.7) | 0.261 | 1 |  |  |
| Midazolam | 2.47 (0-18) | 7.28 (0-24) | <0.0001 | <0.0001 | -0.0031 (-0.151-0.12) | 0.0156 (-0.0956-0.15) | 0.000471 | 0.0127 | 50 (20.8-75) | 52.1 (26.9-78.6) | 0.0348 | 0.939 |  |  |
| Morphine | 4 (0-19.2) | 8 (0-26.4) | <0.0001 | <0.0001 | 0.00666 (-0.134-0.133) | 0.0272 (-0.112-0.161) | <0.0001 | <0.0001 | 50 (22.5-76.1) | 55.2 (26.9-81.5) | <0.0001 | 0.000189 |  |  |
| Propofol | 3.57 (0-18.5) | 6.98 (0-25.9) | <0.0001 | <0.0001 | 0.00365 (-0.13-0.132) | 0.0289 (-0.0981-0.169) | <0.0001 | <0.0001 | 51 (23.3-76.9) | 55.6 (27.6-83.3) | <0.0001 | <0.0001 |  |  |
| Alfentanil | 1.25 (0-16.7) | 7.69 (0-23.7) | 0.00107 | 0.029 | 0.0144 (-0.116-0.117) | 0.0275 (-0.0878-0.166) | 0.0695 | 1 | 50 (24.1-74.1) | 58.5 (28.9-82.3) | 0.0227 | 0.613 |  |  |
| **Name** | **% time LPRx_30 > 0.25** | | | | **% time LPRx_30 > 0.35** | | | | **Mean LPRx_60** | | | |  |  |
|  | **Pre Dose** | **Post Dose** | **P value** | **Adj P Value** | **Pre Dose** | **Post Dose** | **P value** | **Adj P Value** | **Pre Dose** | **Post Dose** | **P value** | **Adj P Value** |  |  |
| Ephedrine | 14.1 (0-29.9) | 13.2 (0-29.6) | 0.813 | 1 | 0 (0-22.9) | 0 (0-22.7) | 0.888 | 1 | 0.0684 (-0.151-0.237) | 0.0412 (-0.0968-0.148) | 0.569 | 1 |  |  |
| Fentanyl | 7.41 (0-35.3) | 14.6 (0-39.1) | 0.0467 | 1 | 0 (0-16.7) | 1.67 (0-24.4) | 0.136 | 1 | 0.0308 (-0.119-0.238) | 0.015 (-0.139-0.228) | 0.539 | 1 |  |  |
| Midazolam | 6.78 (0-31) | 11.1 (0-37.5) | 0.00948 | 0.256 | 0 (0-16) | 0 (0-21.5) | 0.000521 | 0.0141 | 0.0291 (-0.155-0.196) | 0.0324 (-0.104-0.192) | 0.227 | 1 |  |  |
| Morphine | 8.33 (0-32.1) | 12 (0-39.3) | <0.0001 | 0.000461 | 0 (0-17.9) | 0 (0-22.7) | <0.0001 | <0.0001 | 0.0221 (-0.137-0.189) | 0.0284 (-0.141-0.187) | 0.579 | 1 |  |  |
| Propofol | 6.9 (0-32.1) | 10.5 (0-40) | <0.0001 | <0.0001 | 0 (0-16) | 0 (0-22.2) | <0.0001 | <0.0001 | 0.03 (-0.124-0.19) | 0.0387 (-0.101-0.205) | 0.00286 | 0.0773 |  |  |
| Alfentanil | 7.69 (0-33.3) | 13.5 (0-33.7) | 0.313 | 1 | 0 (0-15) | 0 (0-17.2) | 0.361 | 1 | 0.0339 (-0.103-0.163) | 0.0481 (-0.0776-0.19) | 0.253 | 1 |  |  |
| **Name** | **% time LPRx_60 > 0** | | | | **% time LPRx_60 > 0.25** | | | | **% time LPRx_60 > 0.35** | | | |  |  |
|  | **Pre Dose** | **Post Dose** | **P value** | **Adj P Value** | **Pre Dose** | **Post Dose** | **P value** | **Adj P Value** | **Pre Dose** | **Post Dose** | **P value** | **Adj P Value** |  |  |
| Ephedrine | 55 (14.2-88.1) | 52.6 (21.8-89.5) | 0.642 | 1 | 11.6 (0-50) | 0 (0-30.4) | 0.401 | 1 | 2.26 (0-32.2) | 0 (0-23.2) | 0.317 | 1 |  |  |
| Fentanyl | 62.8 (7.69-100) | 57.9 (0-100) | 0.559 | 1 | 0 (0-45.8) | 0 (0-49.1) | 0.258 | 1 | 0 (0-19.3) | 0 (0-20.2) | 0.574 | 1 |  |  |
| Midazolam | 58.6 (7.21-100) | 57.4 (10.3-100) | 0.329 | 1 | 0 (0-40.6) | 0 (0-40.6) | 0.812 | 1 | 0 (0-19.9) | 0 (0-16) | 0.265 | 1 |  |  |
| Morphine | 54 (10.3-96.6) | 57.1 (7.69-100) | 0.206 | 1 | 0 (0-39.2) | 0 (0-37.9) | 0.0748 | 1 | 0 (0-19.2) | 0 (0-16) | 0.0155 | 0.417 |  |  |
| Propofol | 58.3 (12.8-100) | 63.3 (14.8-100) | 0.00015 | 0.00404 | 0 (0-38.5) | 0 (0-40) | 0.498 | 1 | 0 (0-16.7) | 0 (0-14) | 0.0454 | 1 |  |  |
| Alfentanil | 58.4 (17.9-96.6) | 65 (19.1-100) | 0.144 | 1 | 0 (0-33.3) | 0 (0-33.3) | 0.494 | 1 | 0 (0-19.7) | 0 (0-10.4) | 0.0956 | 1 |  |  |

*The table demonstrates the median and interquartile range of the pre/pose dose windows as well as the Wilcox signed ranked test between these windows with p values adjusted using the Bonferroni analysis. The “change” indicates if the continuous infusion was increase/decrease or was a bolus dose. CPP, cerebral prefusion pressure; ICP, intracranial pressure; LPRx_10, pressure reactivity over 10 minutes; LPRx_15, pressure reactivity over 15 minutes; LPRx_20, pressure reactivity over 20 minutes; LPRx_30, pressure reactivity over 30 minutes; LPRx_60, pressure reactivity over 60 minutes;*

# Appendix M. Continuous infusion going from nothing to agent (and vice versa ie, On to Off)

The table contains continuous infusions given from 0 to on or on to 0 within the cohort.

| **Continuous Infusion** | | | | | | | | | | | | | | |
| --- | --- | --- | --- | --- | --- | --- | --- | --- | --- | --- | --- | --- | --- | --- |
| **Name** | **Doses** | **Change** | **Mean MAP** | | | | **Mean CPP** | | | | **% time CPP>60** | | | |
|  |  |  | **Pre Dose** | **Post Dose** | **P value** | **Adj P Value** | **Pre Dose** | **Post Dose** | **P value** | **Adj P Value** | **Pre Dose** | **Post Dose** | **P value** | **Adj P Value** |
| Dobutamine | 443 | Off | 75.6 (69.4-82.7) | 74.6 (69.8-81.8) | 0.66 | 1 | 62.5 (56.6-69.8) | 62.1 (57.4-68.9) | 0.974 | 1 | 28.6 (0-91.6) | 27.7 (0-88.5) | 0.601 | 1 |
| Dobutamine | 398 | On | 73.8 (68.5-80.3) | 73.4 (68.9-80.5) | 0.843 | 1 | 61.5 (56.2-67.7) | 61.8 (56.7-66.8) | 0.922 | 1 | 37 (3.36-93.7) | 31.4 (3.28-93.5) | 0.985 | 1 |
| Midazolam | 4124 | Off | 78.4 (73.2-84.6) | 78.3 (73.3-84.4) | 0.512 | 1 | 64.9 (59.6-70.8) | 64.7 (59.6-70.8) | 0.848 | 1 | 10.7 (0-60.6) | 10.7 (0-59.3) | 0.983 | 1 |
| Midazolam | 3978 | On | 78.6 (73.4-84.9) | 78.4 (73.1-84.4) | 0.0876 | 1 | 64.8 (59.6-71) | 64.7 (59.4-70.8) | 0.431 | 1 | 11.1 (0-59.3) | 10.7 (0-60.7) | 0.534 | 1 |
| Morphine | 6865 | Off | 77.9 (72.6-84.5) | 77.6 (72.4-83.9) | 0.0613 | 1 | 65.5 (59.8-71.8) | 65.3 (59.6-71.5) | 0.12 | 1 | 7.69 (0-55.6) | 9.09 (0-58.6) | 0.296 | 1 |
| Morphine | 6545 | On | 78.3 (72.8-84.9) | 77.5 (72.2-84) | <0.0001 | 0.00012 | 65.8 (60-72.2) | 65.1 (59.6-71.4) | 0.000249 | 0.00673 | 7.41 (0-53.8) | 8 (0-58.3) | 0.126 | 1 |
| Noradrenaline | 9614 | Off | 77.1 (71.7-83.2) | 77.1 (71.8-82.9) | 0.85 | 1 | 64.7 (59.2-71.1) | 64.7 (59.5-71) | 0.539 | 1 | 11.5 (0-62.5) | 11.5 (0-59.3) | 0.49 | 1 |
| Noradrenaline | 9368 | On | 76.4 (71.1-82.6) | 76.2 (71.1-82.2) | 0.157 | 1 | 63.9 (58.7-70.2) | 64.1 (58.9-70.1) | 0.761 | 1 | 18.5 (0-70.4) | 15 (0-68.2) | 0.000402 | 0.0109 |
| Propofol | 12504 | Off | 78.2 (72.6-84.8) | 77.9 (72.5-84.5) | 0.0754 | 1 | 65.7 (60-72.3) | 65.5 (59.8-72.1) | 0.0308 | 0.832 | 7.69 (0-53.6) | 8.33 (0-55.6) | 0.085 | 1 |
| Propofol | 11947 | On | 78.7 (73.1-85.5) | 77.7 (72.2-84.3) | <0.0001 | <0.0001 | 66 (60.2-72.6) | 65.2 (59.8-71.7) | <0.0001 | <0.0001 | 7.69 (0-51.9) | 8.33 (0-56) | 0.0196 | 0.529 |
| Vasopressin | 28 | Off | 72.4 (68.1-82.7) | 73.4 (68.5-81.3) | 0.724 | 1 | 55.2 (51-58.6) | 55.9 (50-58.7) | 0.825 | 1 | 80.2 (64.7-100) | 92 (60.8-100) | 0.912 | 1 |
| Vasopressin | 25 | On | 73.3 (68.2-82.7) | 75.3 (66.9-81.3) | 0.726 | 1 | 55.5 (51-57.8) | 55.2 (49.8-59.1) | 0.46 | 1 | 92.6 (66-100) | 92 (60.8-100) | 0.611 | 1 |
| **Name** | **Change** | **% time CPP>70** | | | | **Mean ICP** | | | | **% time ICP>20** | | | |  |
|  |  | **Pre Dose** | **Post Dose** | **P value** | **Adj P Value** | **Pre Dose** | **Post Dose** | **P value** | **Adj P Value** | **Pre Dose** | **Post Dose** | **P value** | **Adj P Value** |  |
| Dobutamine | Off | 3.23 (0-34.5) | 2.7 (0-32.1) | 0.591 | 1 | 13.2 (9.86-16.3) | 13 (9.49-16.2) | 0.575 | 1 | 0 (0-2.33) | 0 (0-0) | 0.345 | 1 |  |
| Dobutamine | On | 0 (0-21) | 0 (0-16.5) | 0.354 | 1 | 13 (9.57-16) | 12.8 (9.33-16.1) | 0.816 | 1 | 0 (0-2.35) | 0 (0-0) | 0.711 | 1 |  |
| Midazolam | Off | 6.7 (0-51.7) | 6.59 (0-53.3) | 0.536 | 1 | 13.8 (10.4-16.9) | 13.7 (10.2-16.7) | 0.192 | 1 | 0 (0-6.9) | 0 (0-4.47) | 0.0831 | 1 |  |
| Midazolam | On | 7.06 (0-53.6) | 4.67 (0-51.9) | 0.0577 | 1 | 14 (10.5-17.2) | 13.7 (10.3-16.9) | 0.0981 | 1 | 0 (0-7.41) | 0 (0-4.35) | 0.000403 | 0.0109 |  |
| Morphine | Off | 8.26 (0-61.5) | 7.41 (0-60) | 0.0707 | 1 | 12.6 (8.92-16) | 12.6 (8.89-16) | 0.583 | 1 | 0 (0-3.57) | 0 (0-3.33) | 0.282 | 1 |  |
| Morphine | On | 10.7 (0-64.3) | 7.14 (0-59.6) | <0.0001 | 0.000308 | 12.7 (9.03-16.1) | 12.6 (8.82-15.9) | 0.0796 | 1 | 0 (0-3.7) | 0 (0-2.5) | 0.000143 | 0.00386 |  |
| Noradrenaline | Off | 5.8 (0-53.8) | 6.9 (0-53.3) | 0.72 | 1 | 12.2 (8.7-15.7) | 12.2 (8.76-15.6) | 0.773 | 1 | 0 (0-2) | 0 (0-0.198) | 0.127 | 1 |  |
| Noradrenaline | On | 4.17 (0-44.8) | 3.7 (0-44.4) | 0.00279 | 0.0753 | 12.3 (8.78-15.8) | 12.1 (8.64-15.7) | 0.00935 | 0.252 | 0 (0-3.57) | 0 (0-0) | <0.0001 | <0.0001 |  |
| Propofol | Off | 10.3 (0-65.5) | 7.69 (0-64.3) | 0.0187 | 0.506 | 12.5 (8.72-16.2) | 12.6 (8.72-16.2) | 0.736 | 1 | 0 (0-3.7) | 0 (0-3.7) | 0.608 | 1 |  |
| Propofol | On | 11.5 (0-67.9) | 7.41 (0-62.1) | <0.0001 | <0.0001 | 12.8 (9-16.5) | 12.6 (8.75-16.2) | <0.0001 | 0.00156 | 0 (0-6.67) | 0 (0-3.45) | <0.0001 | <0.0001 |  |
| Vasopressin | Off | 0 (0-6.74) | 0 (0-5.82) | 0.423 | 1 | 15.9 (14-18.3) | 16.9 (13.6-18.2) | 0.902 | 1 | 0 (0-8.87) | 3.92 (0-7.22) | 0.583 | 1 |  |
| Vasopressin | On | 0 (0-6.74) | 0 (0-5.82) | 0.797 | 1 | 15.8 (14.9-18.7) | 16.9 (13.9-18.7) | 0.892 | 1 | 5.1 (0-8.87) | 6.07 (0-8.87) | 0.825 | 1 |  |
| **Name** | **Change** | **% time ICP>22** | | | | **Mean LPRx_10** | | | | **% time LPRx_10 > 0** | | | |  |
|  |  | **Pre Dose** | **Post Dose** | **P value** | **Adj P Value** | **Pre Dose** | **Post Dose** | **P value** | **Adj P Value** | **Pre Dose** | **Post Dose** | **P value** | **Adj P Value** |  |
| Dobutamine | Off | 0 (0-0) | 0 (0-0) | 0.388 | 1 | 0.0226 (-0.151-0.177) | 0.0294 (-0.11-0.154) | 0.882 | 1 | 51.9 (32.1-69.2) | 51.4 (35.7-69) | 0.854 | 1 |  |
| Dobutamine | On | 0 (0-0) | 0 (0-0) | 0.337 | 1 | -0.0048 (-0.172-0.179) | 0.0254 (-0.131-0.164) | 0.34 | 1 | 50 (29.6-68.5) | 51.9 (32.4-70.4) | 0.321 | 1 |  |
| Midazolam | Off | 0 (0-0) | 0 (0-0) | 0.0257 | 0.694 | 0.00783 (-0.154-0.157) | 0.00611 (-0.157-0.154) | 0.556 | 1 | 50 (32.1-67.9) | 50 (32-66.7) | 0.552 | 1 |  |
| Midazolam | On | 0 (0-0) | 0 (0-0) | 0.000151 | 0.00407 | 0.0119 (-0.152-0.166) | 0.00274 (-0.159-0.152) | 0.0535 | 1 | 50.5 (31-69.2) | 50 (31.1-67.7) | 0.221 | 1 |  |
| Morphine | Off | 0 (0-0) | 0 (0-0) | 0.416 | 1 | 0.00921 (-0.144-0.159) | 0.00486 (-0.14-0.15) | 0.886 | 1 | 50 (32.1-68) | 50 (33.3-67.7) | 0.75 | 1 |  |
| Morphine | On | 0 (0-0) | 0 (0-0) | <0.0001 | 0.000487 | 0.00671 (-0.153-0.161) | 0.00311 (-0.152-0.154) | 0.406 | 1 | 50 (30.8-69.2) | 50 (32.1-67.9) | 0.579 | 1 |  |
| Noradrenaline | Off | 0 (0-0) | 0 (0-0) | 0.147 | 1 | 0.0108 (-0.142-0.159) | 0.0128 (-0.138-0.164) | 0.362 | 1 | 51.2 (33.3-68) | 51.1 (33.3-69) | 0.304 | 1 |  |
| Noradrenaline | On | 0 (0-0) | 0 (0-0) | <0.0001 | <0.0001 | 0.0149 (-0.143-0.168) | 0.0124 (-0.142-0.163) | 0.356 | 1 | 51.7 (32.1-69.2) | 51.6 (32.3-69) | 0.585 | 1 |  |
| Propofol | Off | 0 (0-0) | 0 (0-0) | 0.182 | 1 | 0.00374 (-0.153-0.154) | 0.0079 (-0.149-0.157) | 0.114 | 1 | 50 (31.5-68) | 50 (32.1-68) | 0.165 | 1 |  |
| Propofol | On | 0 (0-0) | 0 (0-0) | <0.0001 | <0.0001 | 0.0051 (-0.153-0.157) | 0.000809 (-0.157-0.153) | 0.219 | 1 | 50 (31.1-69) | 50 (32-67.9) | 0.432 | 1 |  |
| Vasopressin | Off | 0 (0-1.3) | 0.451 (0-1.3) | 0.499 | 1 | 0.0326 (0.0108-0.0934) | 0.0199 (0.00906-0.0723) | 0.549 | 1 | 53.3 (48.7-55.2) | 53.3 (50.9-57) | 0.889 | 1 |  |
| Vasopressin | On | 0 (0-1.3) | 0.902 (0-1.3) | 0.519 | 1 | 0.0188 (-0.0196-0.0587) | 0.0108 (-0.015-0.0326) | 0.228 | 1 | 53.3 (46.2-58.3) | 51.7 (45.8-53.7) | 0.346 | 1 |  |
| **Name** | **Change** | **% time LPRx_10 > 0.25** | | | | **% time LPRx_10 > 0.35** | | | | **Mean LPRx_15** | | | |  |
|  |  | **Pre Dose** | **Post Dose** | **P value** | **Adj P Value** | **Pre Dose** | **Post Dose** | **P value** | **Adj P Value** | **Pre Dose** | **Post Dose** | **P value** | **Adj P Value** |  |
| Dobutamine | Off | 27.3 (9.57-45.8) | 26.9 (10.3-43.4) | 0.705 | 1 | 17.9 (3.85-35.4) | 18.8 (3.85-33.3) | 0.544 | 1 | 0.0201 (-0.134-0.174) | 0.0111 (-0.131-0.159) | 0.775 | 1 |  |
| Dobutamine | On | 25 (7.14-44.7) | 25.9 (11.5-42.3) | 0.452 | 1 | 16.7 (2.45-34.6) | 17.2 (4-34.2) | 0.429 | 1 | -0.00952 (-0.171-0.159) | 0.016 (-0.161-0.161) | 0.831 | 1 |  |
| Midazolam | Off | 25 (8.7-42.9) | 25 (8-42.3) | 0.326 | 1 | 16.7 (3.57-33.3) | 16.1 (3.57-33.3) | 0.327 | 1 | 0.000892 (-0.139-0.142) | -0.00158 (-0.149-0.138) | 0.322 | 1 |  |
| Midazolam | On | 25 (8-43.3) | 25 (7.69-41.7) | 0.127 | 1 | 16.7 (3.57-33.3) | 15.4 (3.45-33.3) | 0.133 | 1 | 0.01 (-0.145-0.147) | -0.0031 (-0.154-0.135) | 0.0207 | 0.559 |  |
| Morphine | Off | 25 (10-41.9) | 25 (10-41.9) | 0.735 | 1 | 16.3 (3.7-32.5) | 16.7 (3.57-32.7) | 0.751 | 1 | 0.00579 (-0.134-0.143) | 0.00477 (-0.133-0.145) | 0.636 | 1 |  |
| Morphine | On | 24.1 (8-42.6) | 25 (8.93-41.7) | 0.493 | 1 | 15.4 (3.45-33.3) | 16 (3.57-32.1) | 0.346 | 1 | 0.00643 (-0.141-0.149) | 0.00281 (-0.139-0.144) | 0.311 | 1 |  |
| Noradrenaline | Off | 25 (10.3-42.9) | 25.9 (10.3-42.9) | 0.697 | 1 | 16.7 (3.7-33.3) | 17.2 (3.57-33.3) | 0.894 | 1 | 0.00888 (-0.137-0.152) | 0.00652 (-0.136-0.148) | 0.721 | 1 |  |
| Noradrenaline | On | 25 (8.87-43.8) | 25.9 (10.5-43.3) | 0.21 | 1 | 16.7 (3.57-33.3) | 17.9 (3.85-33.3) | 0.0926 | 1 | 0.014 (-0.134-0.159) | 0.0074 (-0.141-0.148) | 0.00391 | 0.106 |  |
| Propofol | Off | 25 (9.36-42.3) | 25 (10.3-42.9) | 0.117 | 1 | 16 (3.57-33.3) | 16.7 (3.7-33.3) | 0.0527 | 1 | 0.0048 (-0.147-0.147) | 0.00875 (-0.137-0.154) | 0.00535 | 0.144 |  |
| Propofol | On | 25 (8.7-42.7) | 24.1 (10-42.3) | 0.946 | 1 | 16 (3.57-33.3) | 16.3 (3.57-33.3) | 0.48 | 1 | 0.00464 (-0.144-0.151) | 0.00319 (-0.144-0.145) | 0.55 | 1 |  |
| Vasopressin | Off | 30.5 (28.3-38) | 28.3 (27.8-30.4) | 0.117 | 1 | 23.4 (21.1-33.3) | 21.1 (19.8-24.1) | 0.123 | 1 | 0.0375 (0.017-0.0688) | 0.0205 (0.0124-0.043) | 0.321 | 1 |  |
| Vasopressin | On | 29.9 (28.3-35.7) | 28.3 (24-30.6) | 0.189 | 1 | 23 (21.1-32.1) | 21.1 (12.9-24) | 0.161 | 1 | 0.0248 (-0.00337-0.0738) | 0.017 (-0.0506-0.0248) | 0.168 | 1 |  |
| **Name** | **Change** | **% time LPRx_15 > 0** | | | | **% time LPRx_15 > 0.25** | | | | **% time LPRx_15 > 0.35** | | | |  |
|  |  | **Pre Dose** | **Post Dose** | **P value** | **Adj P Value** | **Pre Dose** | **Post Dose** | **P value** | **Adj P Value** | **Pre Dose** | **Post Dose** | **P value** | **Adj P Value** |  |
| Dobutamine | Off | 51.9 (29.1-72.4) | 50 (30.8-70.6) | 0.728 | 1 | 22.2 (4.71-42.9) | 19.4 (3.85-39.2) | 0.199 | 1 | 11.9 (0-30.9) | 9.52 (0-29.6) | 0.317 | 1 |  |
| Dobutamine | On | 50 (25-72.7) | 52 (26.1-72) | 0.639 | 1 | 19 (2.44-44.2) | 21.3 (4-40.7) | 0.683 | 1 | 10 (0-30.7) | 10.3 (0-28.7) | 0.878 | 1 |  |
| Midazolam | Off | 50 (29.6-69.2) | 50 (28.1-69.1) | 0.575 | 1 | 19.2 (3.45-39.3) | 17.9 (3.2-37.5) | 0.0856 | 1 | 9.09 (0-28) | 8.33 (0-26.7) | 0.319 | 1 |  |
| Midazolam | On | 51 (28-70.6) | 50 (27.8-68) | 0.135 | 1 | 19.2 (2.73-39.3) | 17.9 (0-37.5) | 0.0801 | 1 | 8.33 (0-28) | 8 (0-26.7) | 0.217 | 1 |  |
| Morphine | Off | 50 (30-69.2) | 50.5 (30.8-69.2) | 0.6 | 1 | 20 (3.57-38.5) | 20 (3.57-38.5) | 0.724 | 1 | 10.3 (0-27.8) | 10.3 (0-27.6) | 0.808 | 1 |  |
| Morphine | On | 50 (28.6-72) | 50 (29.6-69.2) | 0.412 | 1 | 18.5 (0-39.3) | 20 (3.57-38.7) | 0.183 | 1 | 8.33 (0-27.3) | 10.3 (0-27.5) | 0.18 | 1 |  |
| Noradrenaline | Off | 51 (30.3-70.4) | 50 (30-70.4) | 0.567 | 1 | 20.5 (3.7-40) | 20 (3.57-39.3) | 0.129 | 1 | 11 (0-29.2) | 10.3 (0-28) | 0.236 | 1 |  |
| Noradrenaline | On | 51.9 (30-71.5) | 50 (30.4-69.6) | 0.0169 | 0.458 | 20 (3.45-41.4) | 20.7 (3.7-39.3) | 0.702 | 1 | 10.7 (0-29.6) | 10.7 (0-28.6) | 0.514 | 1 |  |
| Propofol | Off | 50 (29.6-70.1) | 51.6 (30.8-70.8) | 0.0153 | 0.412 | 19.2 (3.57-39.3) | 20.7 (3.7-40.7) | 0.00992 | 0.268 | 10 (0-28.6) | 10.7 (0-29.2) | 0.0158 | 0.426 |  |
| Propofol | On | 50 (28.6-70.8) | 50 (30-70) | 0.932 | 1 | 19.2 (3.45-39.8) | 19.4 (3.57-39.3) | 0.192 | 1 | 8.82 (0-28.1) | 10.3 (0-28) | 0.246 | 1 |  |
| Vasopressin | Off | 52.7 (49-56.7) | 53.4 (51.1-58.3) | 0.743 | 1 | 26.8 (25.1-31.1) | 25.1 (15-28.2) | 0.224 | 1 | 17.8 (15.8-22.3) | 16.5 (8.5-19.7) | 0.475 | 1 |  |
| Vasopressin | On | 53.4 (44.4-60) | 52.1 (48-53.6) | 0.414 | 1 | 26.7 (25.1-30.4) | 25.1 (12-27.6) | 0.147 | 1 | 17.8 (16.5-21.4) | 16.5 (6.45-18.9) | 0.15 | 1 |  |
| **Name** | **Change** | **Mean LPRx_20** | | | | **% time LPRx_20 > 0** | | | | **% time LPRx_20 > 0.25** | | | |  |
|  |  | **Pre Dose** | **Post Dose** | **P value** | **Adj P Value** | **Pre Dose** | **Post Dose** | **P value** | **Adj P Value** | **Pre Dose** | **Post Dose** | **P value** | **Adj P Value** |  |
| Dobutamine | Off | 0.0208 (-0.141-0.17) | 0.00259 (-0.148-0.161) | 0.361 | 1 | 53.7 (24.1-75) | 51.1 (25.9-71.4) | 0.447 | 1 | 17.9 (0-43.3) | 15 (0-37.9) | 0.0346 | 0.934 |  |
| Dobutamine | On | -0.00619 (-0.153-0.168) | 0.00692 (-0.177-0.148) | 0.646 | 1 | 50 (21.6-73.6) | 51.9 (24.4-73.1) | 0.886 | 1 | 14.8 (0-42.1) | 16.1 (0-37) | 0.751 | 1 |  |
| Midazolam | Off | 0.00286 (-0.132-0.133) | -0.00645 (-0.139-0.126) | 0.185 | 1 | 50 (26.6-71.4) | 48.3 (25.9-71.4) | 0.179 | 1 | 13.8 (0-36) | 13 (0-34.6) | 0.136 | 1 |  |
| Midazolam | On | 0.00762 (-0.134-0.141) | -0.00444 (-0.143-0.126) | 0.0172 | 0.464 | 51 (25.9-73.9) | 48.3 (25.9-70.8) | 0.0326 | 0.88 | 13.8 (0-36) | 12.3 (0-34.6) | 0.118 | 1 |  |
| Morphine | Off | 0.00438 (-0.129-0.137) | 0.00425 (-0.127-0.14) | 0.612 | 1 | 50 (28.6-72) | 51.3 (28.6-72.3) | 0.571 | 1 | 15.4 (0-36) | 15.4 (0-37) | 0.797 | 1 |  |
| Morphine | On | 0.00696 (-0.136-0.147) | 0.00416 (-0.135-0.138) | 0.365 | 1 | 51.4 (26.2-75) | 50 (28.6-71.4) | 0.267 | 1 | 14.3 (0-37) | 14.8 (0-37) | 0.297 | 1 |  |
| Noradrenaline | Off | 0.00787 (-0.136-0.149) | 0.00334 (-0.132-0.144) | 0.431 | 1 | 50 (28-73.1) | 50.2 (26.9-72.9) | 0.331 | 1 | 16 (0-38.5) | 14.8 (0-37.5) | 0.00129 | 0.0349 |  |
| Noradrenaline | On | 0.0115 (-0.129-0.154) | 0.00211 (-0.139-0.141) | 0.00143 | 0.0386 | 51.7 (28-74.2) | 50 (26.9-71.4) | 0.00117 | 0.0317 | 15.4 (0-39.1) | 15.4 (0-37.9) | 0.391 | 1 |  |
| Propofol | Off | 0.00283 (-0.14-0.143) | 0.0115 (-0.126-0.153) | 0.000137 | 0.0037 | 50 (26.9-73.1) | 51.7 (29.2-74.1) | 0.000195 | 0.00527 | 15.4 (0-37.5) | 16.7 (0-40) | 0.0114 | 0.307 |  |
| Propofol | On | 0.00583 (-0.14-0.146) | 0.0067 (-0.132-0.147) | 0.388 | 1 | 50 (25.9-73.9) | 51.5 (28.6-72.7) | 0.141 | 1 | 14.8 (0-38.1) | 15.6 (0-37.9) | 0.0945 | 1 |  |
| Vasopressin | Off | 0.0278 (-0.0259-0.0617) | 0.0236 (-0.0373-0.0517) | 0.749 | 1 | 52.6 (39.9-58.4) | 52.6 (47-55.9) | 0.7 | 1 | 23.6 (18.5-26.5) | 22.4 (13.5-26.9) | 0.687 | 1 |  |
| Vasopressin | On | 0.0278 (-0.0311-0.0517) | 0.021 (-0.0381-0.0278) | 0.322 | 1 | 52.6 (39.1-57.7) | 51.6 (40-58.3) | 0.8 | 1 | 24.1 (18.5-26.9) | 22.4 (8-25.3) | 0.206 | 1 |  |
| **Name** | **Change** | **% time LPRx_20 > 0.35** | | | | **Mean LPRx_30** | | | | **% time LPRx_30 > 0** | | | |  |
|  |  | **Pre Dose** | **Post Dose** | **P value** | **Adj P Value** | **Pre Dose** | **Post Dose** | **P value** | **Adj P Value** | **Pre Dose** | **Post Dose** | **P value** | **Adj P Value** |  |
| Dobutamine | Off | 6.25 (0-27.6) | 4 (0-26.6) | 0.11 | 1 | 0.0143 (-0.149-0.19) | -0.0166 (-0.168-0.14) | 0.0405 | 1 | 51.6 (21.9-82.1) | 46.5 (17.6-76.7) | 0.0433 | 1 |  |
| Dobutamine | On | 4 (0-27.2) | 4.36 (0-27.4) | 0.865 | 1 | -0.00437 (-0.16-0.173) | -0.0319 (-0.179-0.136) | 0.0794 | 1 | 48.2 (16.2-80) | 44.8 (16.8-76) | 0.384 | 1 |  |
| Midazolam | Off | 3.7 (0-22.2) | 3.57 (0-21.9) | 0.219 | 1 | 0.00149 (-0.133-0.134) | -0.00204 (-0.136-0.123) | 0.181 | 1 | 50 (21.2-76.7) | 48.4 (20.4-75.9) | 0.311 | 1 |  |
| Midazolam | On | 3.57 (0-22.2) | 3.45 (0-21.4) | 0.292 | 1 | 0.0037 (-0.129-0.14) | -0.00475 (-0.137-0.123) | 0.0427 | 1 | 50 (20-79.3) | 48.3 (20-75) | 0.0714 | 1 |  |
| Morphine | Off | 4 (0-24) | 4.17 (0-24.1) | 0.693 | 1 | 0.01 (-0.127-0.146) | 0.00683 (-0.126-0.145) | 0.994 | 1 | 51.9 (24.1-77.8) | 51.1 (23.1-78.6) | 0.603 | 1 |  |
| Morphine | On | 3.57 (0-23.1) | 3.85 (0-24) | 0.0163 | 0.44 | 0.01 (-0.133-0.149) | 0.0046 (-0.127-0.144) | 0.551 | 1 | 51.9 (21.4-80) | 50 (22.6-77.8) | 0.361 | 1 |  |
| Noradrenaline | Off | 4.76 (0-25.9) | 3.85 (0-25) | 0.00643 | 0.174 | 0.00898 (-0.136-0.154) | 0.00461 (-0.135-0.144) | 0.259 | 1 | 51.7 (22.2-78.7) | 50 (20-79.3) | 0.0971 | 1 |  |
| Noradrenaline | On | 4 (0-25.9) | 4.65 (0-25.8) | 0.904 | 1 | 0.0109 (-0.127-0.157) | 0.0036 (-0.136-0.142) | 0.0128 | 0.346 | 51.7 (22.2-80.8) | 50 (20.8-77.8) | 0.00415 | 0.112 |  |
| Propofol | Off | 4 (0-25.5) | 6.67 (0-26.9) | 0.0199 | 0.539 | 0.00707 (-0.135-0.154) | 0.02 (-0.116-0.171) | <0.0001 | <0.0001 | 51.6 (22.6-79.3) | 53.8 (25-81.8) | <0.0001 | <0.0001 |  |
| Propofol | On | 3.85 (0-25) | 4.35 (0-26.1) | 0.101 | 1 | 0.00621 (-0.135-0.153) | 0.0148 (-0.118-0.159) | <0.0001 | 0.000996 | 50 (22.2-80) | 53.6 (25-80) | 0.000148 | 0.004 |  |
| Vasopressin | Off | 15.2 (9.18-20.9) | 14.1 (5.36-18.3) | 0.521 | 1 | 0.0245 (-0.151-0.0357) | 0.0267 (-0.0238-0.0643) | 0.371 | 1 | 51.5 (30.4-53.4) | 51.6 (47-59.3) | 0.251 | 1 |  |
| Vasopressin | On | 15.2 (11.1-18.8) | 14.1 (3.7-16.3) | 0.128 | 1 | 0.0267 (-0.2-0.0387) | 0.0245 (-0.0988-0.0357) | 0.923 | 1 | 51.6 (31-55.5) | 51.5 (32.3-53.3) | 0.899 | 1 |  |
| **Name** | **Change** | **% time LPRx_30 > 0.25** | | | | **% time LPRx_30 > 0.35** | | | | **Mean LPRx_60** | | | |  |
|  |  | **Pre Dose** | **Post Dose** | **P value** | **Adj P Value** | **Pre Dose** | **Post Dose** | **P value** | **Adj P Value** | **Pre Dose** | **Post Dose** | **P value** | **Adj P Value** |  |
| Dobutamine | Off | 11.5 (0-44.4) | 8 (0-32.9) | 0.00982 | 0.265 | 0 (0-24.1) | 0 (0-19.7) | 0.0752 | 1 | 0.019 (-0.153-0.218) | -0.000687 (-0.169-0.2) | 0.159 | 1 |  |
| Dobutamine | On | 8.6 (0-41.8) | 5.61 (0-35.9) | 0.139 | 1 | 0 (0-22) | 0 (0-23.1) | 0.655 | 1 | 0.0192 (-0.161-0.199) | -0.0114 (-0.172-0.15) | 0.0728 | 1 |  |
| Midazolam | Off | 4 (0-32.1) | 3.85 (0-31) | 0.139 | 1 | 0 (0-16.7) | 0 (0-15.5) | 0.379 | 1 | 0.00663 (-0.135-0.162) | 0.0115 (-0.12-0.154) | 0.103 | 1 |  |
| Midazolam | On | 3.85 (0-33.3) | 3.7 (0-30.8) | 0.31 | 1 | 0 (0-16.7) | 0 (0-15.5) | 0.413 | 1 | 0.00737 (-0.134-0.161) | 0.00856 (-0.122-0.156) | 0.369 | 1 |  |
| Morphine | Off | 7.69 (0-34.6) | 7.84 (0-35.5) | 0.879 | 1 | 0 (0-20) | 0 (0-20.7) | 0.525 | 1 | 0.0205 (-0.133-0.184) | 0.0194 (-0.121-0.176) | 0.841 | 1 |  |
| Morphine | On | 6.67 (0-34.1) | 7.14 (0-35.7) | 0.16 | 1 | 0 (0-17.9) | 0 (0-20.4) | 0.0229 | 0.618 | 0.0168 (-0.136-0.182) | 0.0161 (-0.123-0.175) | 0.865 | 1 |  |
| Noradrenaline | Off | 8.33 (0-37) | 4.55 (0-34.5) | <0.0001 | <0.0001 | 0 (0-21.4) | 0 (0-19.4) | <0.0001 | 0.000265 | 0.0215 (-0.13-0.193) | 0.0143 (-0.129-0.175) | 0.0614 | 1 |  |
| Noradrenaline | On | 7.69 (0-37) | 7.14 (0-35) | 0.0288 | 0.778 | 0 (0-20.8) | 0 (0-20.7) | 0.0561 | 1 | 0.0188 (-0.134-0.189) | 0.0149 (-0.129-0.18) | 0.495 | 1 |  |
| Propofol | Off | 7.69 (0-37) | 8 (0-40) | 0.0343 | 0.926 | 0 (0-21.3) | 0 (0-23.3) | 0.0362 | 0.976 | 0.0192 (-0.127-0.188) | 0.0291 (-0.11-0.205) | <0.0001 | 0.000307 |  |
| Propofol | On | 7.41 (0-36) | 8 (0-39.1) | 0.0174 | 0.47 | 0 (0-20) | 0 (0-23.3) | 0.0127 | 0.344 | 0.0183 (-0.128-0.183) | 0.0276 (-0.108-0.202) | <0.0001 | 0.000702 |  |
| Vasopressin | Off | 19.9 (5.65-21.9) | 20.5 (9.65-24) | 0.915 | 1 | 12.1 (2.78-17.1) | 11.9 (0-12.7) | 0.382 | 1 | 0.0158 (-0.0343-0.0616) | 0.0157 (-0.119-0.0398) | 0.761 | 1 |  |
| Vasopressin | On | 20.6 (3.7-21.9) | 19.7 (4-21.9) | 0.646 | 1 | 12.4 (0-14.8) | 11.9 (0-12.4) | 0.331 | 1 | 0.01 (-0.119-0.033) | 0.0102 (-0.116-0.0326) | 0.831 | 1 |  |
| **Name** | **Change** | **% time LPRx_60 > 0** | | | | **% time LPRx_60 > 0.25** | | | | **% time LPRx_60 > 0.35** | | | |  |
|  |  | **Pre Dose** | **Post Dose** | **P value** | **Adj P Value** | **Pre Dose** | **Post Dose** | **P value** | **Adj P Value** | **Pre Dose** | **Post Dose** | **P value** | **Adj P Value** |  |
| Dobutamine | Off | 56 (7.23-98.3) | 50 (3.57-100) | 0.658 | 1 | 0 (0-46.4) | 0 (0-34.6) | 0.0125 | 0.337 | 0 (0-24.1) | 0 (0-11.3) | 0.0502 | 1 |  |
| Dobutamine | On | 56.3 (3.88-100) | 46 (0-100) | 0.219 | 1 | 0 (0-39.8) | 0 (0-28.2) | 0.0418 | 1 | 0 (0-15.9) | 0 (0-7.1) | 0.191 | 1 |  |
| Midazolam | Off | 50.6 (6.84-96.3) | 54.2 (7.69-96.7) | 0.209 | 1 | 0 (0-29.8) | 0 (0-27.6) | 0.411 | 1 | 0 (0-8) | 0 (0-7.14) | 0.0633 | 1 |  |
| Midazolam | On | 51.9 (4-96.6) | 52.2 (7.14-96.7) | 0.432 | 1 | 0 (0-29.9) | 0 (0-27.4) | 0.378 | 1 | 0 (0-8.33) | 0 (0-4.35) | 0.115 | 1 |  |
| Morphine | Off | 55.6 (10-100) | 55.6 (10.3-100) | 0.68 | 1 | 0 (0-37.9) | 0 (0-34.5) | 0.0633 | 1 | 0 (0-16.7) | 0 (0-13.8) | 0.0238 | 0.643 |  |
| Morphine | On | 55.6 (7.14-100) | 55.2 (7.14-100) | 0.918 | 1 | 0 (0-35.5) | 0 (0-33.3) | 0.309 | 1 | 0 (0-13.8) | 0 (0-10.8) | 0.083 | 1 |  |
| Noradrenaline | Off | 55.6 (11.2-100) | 54.3 (4-100) | 0.0316 | 0.853 | 0 (0-40) | 0 (0-33.7) | <0.0001 | <0.0001 | 0 (0-17.9) | 0 (0-11.1) | <0.0001 | <0.0001 |  |
| Noradrenaline | On | 55.6 (10.3-100) | 55.2 (3.7-100) | 0.0965 | 1 | 0 (0-38.1) | 0 (0-34.5) | <0.0001 | 0.000498 | 0 (0-16.7) | 0 (0-11) | <0.0001 | 0.000249 |  |
| Propofol | Off | 56 (10.5-100) | 60 (11.5-100) | <0.0001 | 0.000492 | 0 (0-38.5) | 0 (0-41.4) | 0.822 | 1 | 0 (0-15.6) | 0 (0-16.3) | 0.776 | 1 |  |
| Propofol | On | 55.6 (10-100) | 60 (11.1-100) | <0.0001 | 0.00269 | 0 (0-36.7) | 0 (0-40) | 0.946 | 1 | 0 (0-14.8) | 0 (0-15.6) | 0.897 | 1 |  |
| Vasopressin | Off | 50.8 (35.2-77) | 50.7 (25.2-54.3) | 0.811 | 1 | 14.7 (0-15.8) | 14.5 (0-16.4) | 0.925 | 1 | 9.66 (0-10.2) | 9.66 (0-10.8) | 0.993 | 1 |  |
| Vasopressin | On | 49.9 (26.7-51.5) | 50 (3.33-53.3) | 0.703 | 1 | 14.5 (0-15.2) | 14.5 (0-15.2) | 0.96 | 1 | 9.66 (0-9.94) | 9.66 (0-9.94) | 0.904 | 1 |  |

*The table demonstrates the median and interquartile range of the pre/pose dose windows as well as the Wilcox signed ranked test between these windows with p values adjusted using the Bonferroni analysis. The “change” indicates if the continuous infusion was increase/decrease or was a bolus dose. CPP, cerebral prefusion pressure; ICP, intracranial pressure; LPRx_10, pressure reactivity over 10 minutes; LPRx_15, pressure reactivity over 15 minutes; LPRx_20, pressure reactivity over 20 minutes; LPRx_30, pressure reactivity over 30 minutes; LPRx_60, pressure reactivity over 60 minutes;*

# Appendix N. Assessing the High/Medium/Low of different infusion agent

The table contains all continuous infusions given with the cohort, separated into different dose amounts.

| **Continuous Intravenous** | | | | | | | | | | | | | | |
| --- | --- | --- | --- | --- | --- | --- | --- | --- | --- | --- | --- | --- | --- | --- |
| **Name** | **Doses** | **Mean Dose Change** | **Mean MAP** | | | | **Mean CPP** | | | | **% time CPP>60** | | | |
|  |  |  | **Pre Dose** | **Post Dose** | **P value** | **Adj P Value** | **Pre Dose** | **Post Dose** | **P value** | **Adj P Value** | **Pre Dose** | **Post Dose** | **P value** | **Adj P Value** |
| Dobutamine | 573 | Low | 75.8 (69.5-83.1) | 75.1 (69.8-82.2) | 0.563 | 1 | 63.1 (57-69.8) | 62.4 (57.6-68.7) | 0.859 | 1 | 25.9 (0-90.9) | 25.9 (0-86.2) | 0.506 | 1 |
| Dobutamine | 245 | Medium | 73.2 (68.1-78.5) | 72.7 (68.6-78.5) | 0.928 | 1 | 60.8 (55.9-66.7) | 61.2 (56.1-65.7) | 0.958 | 1 | 44 (4-94.1) | 43.9 (3.57-96.3) | 0.898 | 1 |
| Dobutamine | 23 | High | 69.9 (66.3-74.7) | 70.8 (66.1-76.6) | 0.728 | 1 | 56.2 (53.4-63.5) | 58.4 (52.2-63.4) | 0.601 | 1 | 96.3 (36.3-100) | 92.3 (11.8-100) | 0.598 | 1 |
| Midazolam | 6406 | Medium | 78.3 (73.1-84.4) | 78.1 (73-84) | 0.11 | 1 | 64.8 (59.5-70.8) | 64.6 (59.4-70.7) | 0.49 | 1 | 11.1 (0-60.7) | 10.7 (0-60.7) | 0.636 | 1 |
| Midazolam | 1696 | High | 79.3 (74.1-85.9) | 79.3 (73.9-85.8) | 0.625 | 1 | 65.2 (59.8-71.4) | 65 (59.6-71.2) | 0.879 | 1 | 9.72 (0-57.1) | 9.09 (0-56.2) | 0.953 | 1 |
| Morphine | 9460 | Low | 77.9 (72.5-84.5) | 77.4 (72.2-83.7) | 0.00069 | 0.0186 | 65.8 (60-72.5) | 65.5 (59.8-71.9) | 0.0111 | 0.299 | 7.41 (0-53.6) | 7.45 (0-56.7) | 0.166 | 1 |
| Morphine | 2555 | Medium | 77.6 (72.7-84.2) | 76.9 (72.1-83.1) | 0.00221 | 0.0596 | 64.6 (59.2-70.3) | 64.1 (58.9-69.7) | 0.00595 | 0.161 | 11.8 (0-65.5) | 15.4 (0-70) | 0.16 | 1 |
| Morphine | 1395 | High | 79.8 (74-87.1) | 79.4 (73.6-86.8) | 0.281 | 1 | 66 (60.1-72.6) | 65.5 (59.9-72.4) | 0.265 | 1 | 5 (0-51.7) | 6.45 (0-54.9) | 0.84 | 1 |
| Noradrenaline | 10764 | Low | 77.1 (71.7-83.3) | 77 (71.7-82.8) | 0.306 | 1 | 64.8 (59.3-71.2) | 64.7 (59.5-70.9) | 0.915 | 1 | 11.7 (0-61.5) | 12 (0-59.3) | 0.46 | 1 |
| Noradrenaline | 8098 | Medium | 76.3 (71.1-82.4) | 76.3 (71.2-82.4) | 0.799 | 1 | 63.9 (58.7-70) | 64.1 (58.9-70) | 0.339 | 1 | 18.5 (0-70.6) | 14.8 (0-69) | 0.000303 | 0.00817 |
| Noradrenaline | 120 | High | 73.4 (69.1-77.5) | 74 (67.7-80.1) | 0.567 | 1 | 57.6 (52.9-62.6) | 58.2 (54.1-63.9) | 0.365 | 1 | 89.3 (33.3-100) | 74.1 (13.2-100) | 0.649 | 1 |
| Propofol | 13132 | Low | 78.3 (72.7-84.8) | 77.9 (72.5-84.6) | 0.0294 | 0.793 | 65.8 (60.1-72.4) | 65.5 (59.8-72.1) | 0.0144 | 0.39 | 7.69 (0-52) | 8.25 (0-55.3) | 0.075 | 1 |
| Propofol | 10651 | Medium | 78.7 (73-85.3) | 77.7 (72.2-84.1) | <0.0001 | <0.0001 | 65.9 (60.1-72.4) | 65.1 (59.7-71.5) | <0.0001 | <0.0001 | 7.69 (0-52) | 8.7 (0-56.7) | 0.0222 | 0.599 |
| Propofol | 668 | High | 79.1 (72.8-87.2) | 78 (71.6-85.9) | 0.0647 | 1 | 66.6 (59.3-74) | 65.4 (58.8-73.5) | 0.196 | 1 | 7.41 (0-61.5) | 7.41 (0-66.7) | 0.746 | 1 |
| Vasopressin | 28 | Low | 72.4 (68.1-82.7) | 73.4 (68.5-81.3) | 0.724 | 1 | 55.2 (51-58.6) | 55.9 (50-58.7) | 0.825 | 1 | 80.2 (64.7-100) | 92 (60.8-100) | 0.912 | 1 |
| Vasopressin | 24 | Medium | 73.2 (68-82.7) | 74.3 (66.9-81.3) | 0.733 | 1 | 54.8 (50.6-58) | 54.9 (49.6-59.1) | 0.476 | 1 | 94.6 (64.7-100) | 94.3 (60.3-100) | 0.625 | 1 |
| **Name** | **Mean Dose Change** | **% time CPP>70** | | | | **Mean ICP** | | | | **% time ICP>20** | | | |  |
|  |  | **Pre Dose** | **Post Dose** | **P value** | **Adj P Value** | **Pre Dose** | **Post Dose** | **P value** | **Adj P Value** | **Pre Dose** | **Post Dose** | **P value** | **Adj P Value** |  |
| Dobutamine | Low | 3.7 (0-35.7) | 2.7 (0-33.3) | 0.447 | 1 | 13 (9.98-16.2) | 13 (9.51-16.2) | 0.594 | 1 | 0 (0-2.38) | 0 (0-0) | 0.36 | 1 |  |
| Dobutamine | Medium | 0 (0-15.4) | 0 (0-8.33) | 0.489 | 1 | 12.9 (8.86-16.2) | 12.5 (9.08-16.1) | 0.88 | 1 | 0 (0-2.44) | 0 (0-0) | 0.492 | 1 |  |
| Dobutamine | High | 0 (0-15.1) | 0 (0-10.2) | 0.97 | 1 | 13.7 (12.2-15.3) | 13.4 (12.1-15.5) | 0.828 | 1 | 0 (0-0) | 0 (0-4.05) | 0.18 | 1 |  |
| Midazolam | Medium | 6.9 (0-50) | 4.76 (0-51.6) | 0.0858 | 1 | 13.7 (10.2-16.8) | 13.5 (10-16.6) | 0.0543 | 1 | 0 (0-6.67) | 0 (0-3.85) | 0.000411 | 0.0111 |  |
| Midazolam | High | 7.02 (0-58.6) | 6.9 (0-58.6) | 0.578 | 1 | 14.7 (11.3-17.8) | 14.6 (11.1-17.6) | 0.385 | 1 | 0 (0-10.7) | 0 (0-8.7) | 0.181 | 1 |  |
| Morphine | Low | 10.7 (0-65.5) | 8 (0-63.8) | 0.00195 | 0.0528 | 12.2 (8.53-15.6) | 12.2 (8.45-15.6) | 0.208 | 1 | 0 (0-3.12) | 0 (0-0.733) | 0.0388 | 1 |  |
| Morphine | Medium | 5.88 (0-48.1) | 3.57 (0-40) | 0.00062 | 0.0167 | 13.4 (9.98-16.6) | 13.4 (9.88-16.6) | 0.454 | 1 | 0 (0-4.17) | 0 (0-3.45) | 0.00355 | 0.0958 |  |
| Morphine | High | 11.1 (0-69.6) | 10 (0-69.8) | 0.3 | 1 | 14.3 (10.5-17.2) | 14.1 (10.3-17.3) | 0.568 | 1 | 0 (0-8.76) | 0 (0-7.85) | 0.254 | 1 |  |
| Noradrenaline | Low | 6.82 (0-54.2) | 6.9 (0-52.4) | 0.561 | 1 | 12.2 (8.64-15.7) | 12.1 (8.62-15.5) | 0.266 | 1 | 0 (0-2.13) | 0 (0-0.0444) | 0.0134 | 0.363 |  |
| Noradrenaline | Medium | 4 (0-43.2) | 3.7 (0-44.1) | 0.0279 | 0.752 | 12.4 (8.84-15.9) | 12.3 (8.8-15.7) | 0.0811 | 1 | 0 (0-3.57) | 0 (0-0) | <0.0001 | <0.0001 |  |
| Noradrenaline | High | 0 (0-3.6) | 0 (0-3.64) | 0.785 | 1 | 14 (10.8-18.4) | 14.2 (10-17.7) | 0.453 | 1 | 0 (0-14.3) | 0 (0-9.11) | 0.936 | 1 |  |
| Propofol | Low | 10.3 (0-66.7) | 8 (0-65) | 0.00662 | 0.179 | 12.5 (8.69-16.2) | 12.6 (8.69-16.2) | 0.89 | 1 | 0 (0-3.7) | 0 (0-3.57) | 0.288 | 1 |  |
| Propofol | Medium | 11.1 (0-66.7) | 7.14 (0-60.7) | <0.0001 | <0.0001 | 12.8 (9.07-16.5) | 12.6 (8.81-16.2) | 0.000129 | 0.00347 | 0 (0-6.67) | 0 (0-3.45) | <0.0001 | <0.0001 |  |
| Propofol | High | 16.7 (0-83.5) | 11.1 (0-73.3) | 0.0243 | 0.655 | 13.3 (8.73-17) | 12.9 (8.47-16.7) | 0.359 | 1 | 0 (0-10.3) | 0 (0-7.14) | 0.0691 | 1 |  |
| Vasopressin | Low | 0 (0-6.74) | 0 (0-5.82) | 0.423 | 1 | 15.9 (14-18.3) | 16.9 (13.6-18.2) | 0.902 | 1 | 0 (0-8.87) | 3.92 (0-7.22) | 0.583 | 1 |  |
| Vasopressin | Medium | 0 (0-6.74) | 0 (0-5.82) | 0.791 | 1 | 15.8 (14.7-18.7) | 17 (13.6-18.7) | 0.942 | 1 | 4.22 (0-8.87) | 5.12 (0-7.63) | 0.805 | 1 |  |
| **Name** | **Mean Dose Change** | **% time ICP>22** | | | | **Mean LPRx_10** | | | | **% time LPRx_10 > 0** | | | |  |
|  |  | **Pre Dose** | **Post Dose** | **P value** | **Adj P Value** | **Pre Dose** | **Post Dose** | **P value** | **Adj P Value** | **Pre Dose** | **Post Dose** | **P value** | **Adj P Value** |  |
| Dobutamine | Low | 0 (0-0) | 0 (0-0) | 0.371 | 1 | 0.00972 (-0.16-0.169) | 0.0261 (-0.123-0.149) | 0.711 | 1 | 51.1 (32-69) | 50 (34.1-69) | 0.855 | 1 |  |
| Dobutamine | Medium | 0 (0-0) | 0 (0-0) | 0.219 | 1 | 0.00237 (-0.16-0.185) | 0.0359 (-0.113-0.177) | 0.543 | 1 | 51 (31-69.2) | 53.7 (34.6-71.4) | 0.377 | 1 |  |
| Dobutamine | High | 0 (0-0) | 0 (0-0) | 0.42 | 1 | 0.0103 (-0.212-0.212) | 0.057 (-0.0871-0.196) | 0.446 | 1 | 48 (28.3-64.7) | 58.6 (36.8-68.8) | 0.317 | 1 |  |
| Midazolam | Medium | 0 (0-0) | 0 (0-0) | <0.0001 | 0.00263 | 0.0116 (-0.151-0.162) | 0.00697 (-0.155-0.157) | 0.198 | 1 | 51 (32-69) | 50 (32-67.9) | 0.245 | 1 |  |
| Midazolam | High | 0 (0-3.45) | 0 (0-0.796) | 0.0811 | 1 | 0.00083 (-0.158-0.163) | -0.00688 (-0.165-0.136) | 0.178 | 1 | 50 (29.9-68) | 48.3 (30.8-66.7) | 0.624 | 1 |  |
| Morphine | Low | 0 (0-0) | 0 (0-0) | 0.0634 | 1 | 0.00896 (-0.144-0.158) | 0.00862 (-0.138-0.154) | 0.81 | 1 | 50 (32.1-68.9) | 50 (33.3-67.9) | 0.649 | 1 |  |
| Morphine | Medium | 0 (0-0) | 0 (0-0) | 0.000126 | 0.0034 | 0.0099 (-0.156-0.164) | -0.00118 (-0.162-0.147) | 0.136 | 1 | 51.7 (31-69) | 50 (31-66.7) | 0.145 | 1 |  |
| Morphine | High | 0 (0-0) | 0 (0-0) | 0.384 | 1 | 0.00113 (-0.175-0.163) | -0.00876 (-0.172-0.15) | 0.55 | 1 | 50 (28.6-69) | 48.1 (30-66.7) | 0.351 | 1 |  |
| Noradrenaline | Low | 0 (0-0) | 0 (0-0) | 0.0449 | 1 | 0.0108 (-0.144-0.16) | 0.0113 (-0.14-0.164) | 0.459 | 1 | 51.2 (33.3-68.2) | 51 (33.3-69) | 0.311 | 1 |  |
| Noradrenaline | Medium | 0 (0-0) | 0 (0-0) | <0.0001 | <0.0001 | 0.015 (-0.141-0.169) | 0.0141 (-0.141-0.163) | 0.442 | 1 | 51.6 (32.6-69.2) | 51.7 (32.3-69) | 0.5 | 1 |  |
| Noradrenaline | High | 0 (0-2.61) | 0 (0-0) | 0.221 | 1 | 0.0456 (-0.0717-0.156) | 0.0316 (-0.12-0.149) | 0.399 | 1 | 53.7 (40.7-65.8) | 53.6 (37.7-67.9) | 0.821 | 1 |  |
| Propofol | Low | 0 (0-0) | 0 (0-0) | 0.0636 | 1 | 0.00439 (-0.152-0.154) | 0.00721 (-0.149-0.156) | 0.29 | 1 | 50 (32-68) | 50 (32.1-67.9) | 0.334 | 1 |  |
| Propofol | Medium | 0 (0-0) | 0 (0-0) | <0.0001 | <0.0001 | 0.00525 (-0.155-0.158) | 0.00246 (-0.157-0.155) | 0.52 | 1 | 50 (31-69) | 50 (32-68) | 0.769 | 1 |  |
| Propofol | High | 0 (0-3.36) | 0 (0-0) | 0.101 | 1 | -0.00453 (-0.155-0.151) | -0.0134 (-0.149-0.15) | 0.643 | 1 | 50 (29.1-68) | 48.1 (31-66.7) | 0.699 | 1 |  |
| Vasopressin | Low | 0 (0-1.3) | 0.451 (0-1.3) | 0.499 | 1 | 0.0326 (0.0108-0.0934) | 0.0199 (0.00906-0.0723) | 0.549 | 1 | 53.3 (48.7-55.2) | 53.3 (50.9-57) | 0.889 | 1 |  |
| Vasopressin | Medium | 0 (0-1.3) | 0.902 (0-1.3) | 0.496 | 1 | 0.0188 (-0.0238-0.0589) | 0.0108 (-0.0291-0.0237) | 0.227 | 1 | 53.3 (45.3-58.5) | 51.7 (45.6-53.4) | 0.352 | 1 |  |
| **Name** | **Mean Dose Change** | **% time LPRx_10 > 0.25** | | | | **% time LPRx_10 > 0.35** | | | | **Mean LPRx_15** | | | |  |
|  |  | **Pre Dose** | **Post Dose** | **P value** | **Adj P Value** | **Pre Dose** | **Post Dose** | **P value** | **Adj P Value** | **Pre Dose** | **Post Dose** | **P value** | **Adj P Value** |  |
| Dobutamine | Low | 26.7 (8.82-44.4) | 26.1 (10.5-42.9) | 0.922 | 1 | 17.2 (3.7-34.8) | 18.5 (3.7-33.3) | 0.85 | 1 | 0.00158 (-0.14-0.17) | 0.00516 (-0.143-0.156) | 0.718 | 1 |  |
| Dobutamine | Medium | 25.9 (7.5-44.7) | 25.8 (12-43.8) | 0.692 | 1 | 18.5 (2.94-36.4) | 16.7 (4.17-34.6) | 0.766 | 1 | 0.0178 (-0.155-0.169) | 0.0291 (-0.139-0.165) | 0.841 | 1 |  |
| Dobutamine | High | 26.9 (2-47) | 36 (19.3-45.1) | 0.559 | 1 | 21.4 (1.85-34) | 26.7 (11.6-32.2) | 0.659 | 1 | -0.0847 (-0.182-0.13) | -0.00375 (-0.181-0.166) | 0.542 | 1 |  |
| Midazolam | Medium | 25.6 (8.89-43.3) | 25 (7.69-42.9) | 0.12 | 1 | 16.7 (3.57-34.2) | 16 (3.57-33.3) | 0.136 | 1 | 0.00722 (-0.138-0.147) | 0.000996 (-0.148-0.139) | 0.062 | 1 |  |
| Midazolam | High | 24.1 (7.41-42.3) | 23.5 (8-40) | 0.428 | 1 | 16 (3.45-33.3) | 14.8 (3.57-31) | 0.36 | 1 | -0.00348 (-0.157-0.137) | -0.0145 (-0.161-0.12) | 0.151 | 1 |  |
| Morphine | Low | 25 (9.09-41.9) | 25 (10-41.9) | 0.389 | 1 | 15.6 (3.57-32.4) | 16.3 (3.7-32.5) | 0.352 | 1 | 0.00626 (-0.133-0.146) | 0.00814 (-0.13-0.149) | 0.408 | 1 |  |
| Morphine | Medium | 25 (9.31-44) | 25 (8.7-42.3) | 0.351 | 1 | 16.7 (3.57-33.3) | 16 (3.57-32.1) | 0.325 | 1 | 0.0106 (-0.141-0.157) | -0.00237 (-0.143-0.139) | 0.11 | 1 |  |
| Morphine | High | 24 (7.69-41.3) | 24.1 (7.14-41.4) | 0.832 | 1 | 15.4 (3.45-31) | 15.4 (3.57-32) | 0.802 | 1 | 0.000257 (-0.168-0.132) | -0.0104 (-0.164-0.129) | 0.305 | 1 |  |
| Noradrenaline | Low | 25 (10.3-42.9) | 25.9 (10.3-42.9) | 0.61 | 1 | 16.7 (3.7-33.3) | 17.2 (3.57-33.3) | 0.754 | 1 | 0.00852 (-0.138-0.152) | 0.00577 (-0.137-0.147) | 0.618 | 1 |  |
| Noradrenaline | Medium | 25 (9.09-44) | 25.9 (10.7-43.3) | 0.199 | 1 | 16.7 (3.57-34.4) | 17.4 (3.85-33.3) | 0.0849 | 1 | 0.0155 (-0.134-0.16) | 0.00762 (-0.141-0.149) | 0.00427 | 0.115 |  |
| Noradrenaline | High | 31.3 (16.5-48) | 28.5 (12.4-42.8) | 0.335 | 1 | 24.6 (10.8-33.6) | 20.2 (7.14-37) | 0.264 | 1 | 0.0573 (-0.0685-0.187) | 0.0345 (-0.0967-0.174) | 0.535 | 1 |  |
| Propofol | Low | 25 (9.68-42.3) | 25 (10.3-42.6) | 0.28 | 1 | 16 (3.7-33.3) | 16.7 (3.7-33.3) | 0.124 | 1 | 0.00462 (-0.144-0.148) | 0.00827 (-0.137-0.152) | 0.0189 | 0.509 |  |
| Propofol | Medium | 25 (8.7-42.9) | 24.4 (10-42.3) | 0.75 | 1 | 16 (3.57-33.3) | 16.7 (3.57-33.3) | 0.242 | 1 | 0.00468 (-0.145-0.15) | 0.00464 (-0.144-0.147) | 0.804 | 1 |  |
| Propofol | High | 25.9 (8.25-41.7) | 25 (10.3-42) | 0.691 | 1 | 17.2 (3.7-33.3) | 17.2 (3.7-32.2) | 0.88 | 1 | 0.00429 (-0.162-0.152) | -0.00378 (-0.144-0.135) | 0.884 | 1 |  |
| Vasopressin | Low | 30.5 (28.3-38) | 28.3 (27.8-30.4) | 0.117 | 1 | 23.4 (21.1-33.3) | 21.1 (19.8-24.1) | 0.123 | 1 | 0.0375 (0.017-0.0688) | 0.0205 (0.0124-0.043) | 0.321 | 1 |  |
| Vasopressin | Medium | 30 (27.7-36) | 28.3 (22.5-30.9) | 0.186 | 1 | 22.9 (19.7-32.1) | 21.1 (12.7-24) | 0.157 | 1 | 0.0248 (-0.0201-0.077) | 0.017 (-0.0513-0.0248) | 0.169 | 1 |  |
| **Name** | **Mean Dose Change** | **% time LPRx_15 > 0** | | | | **% time LPRx_15 > 0.25** | | | | **% time LPRx_15 > 0.35** | | | |  |
|  |  | **Pre Dose** | **Post Dose** | **P value** | **Adj P Value** | **Pre Dose** | **Post Dose** | **P value** | **Adj P Value** | **Pre Dose** | **Post Dose** | **P value** | **Adj P Value** |  |
| Dobutamine | Low | 50 (27.9-72.4) | 50 (28.6-70.4) | 0.715 | 1 | 20.5 (4-42.9) | 20 (3.57-39.3) | 0.324 | 1 | 11.1 (0-30.8) | 10 (0-29.3) | 0.55 | 1 |  |
| Dobutamine | Medium | 53.6 (28.1-73.1) | 53.6 (29.3-75.9) | 0.588 | 1 | 20 (3.7-45.9) | 21.4 (4.44-41.4) | 0.947 | 1 | 11.1 (0-31) | 9.38 (0-29.6) | 0.61 | 1 |  |
| Dobutamine | High | 35.7 (27-68.1) | 50 (26.4-65.9) | 0.775 | 1 | 21.4 (0-41.7) | 27.6 (7.68-41.5) | 0.452 | 1 | 10.7 (0-22.9) | 20.7 (1.92-34.2) | 0.285 | 1 |  |
| Midazolam | Medium | 51 (29.6-70.4) | 50 (28.6-69) | 0.159 | 1 | 19.4 (3.45-39.3) | 18.5 (3.12-37.9) | 0.0256 | 0.69 | 9.34 (0-28.6) | 8.7 (0-26.9) | 0.246 | 1 |  |
| Midazolam | High | 50 (26.7-67.9) | 48.3 (26.9-66.7) | 0.684 | 1 | 18.5 (0-38.5) | 17.2 (0-35.7) | 0.316 | 1 | 7.41 (0-26.7) | 7.41 (0-24.1) | 0.241 | 1 |  |
| Morphine | Low | 50 (29.6-70.4) | 51.7 (31-70.3) | 0.262 | 1 | 19.2 (3.45-38.5) | 20 (3.7-39.3) | 0.096 | 1 | 9.68 (0-27.6) | 10.3 (0-27.6) | 0.116 | 1 |  |
| Morphine | Medium | 51 (29.6-72) | 50 (28.6-68) | 0.0827 | 1 | 20 (3.33-40.4) | 20 (3.45-39.3) | 0.633 | 1 | 10.3 (0-28) | 10.7 (0-26.9) | 0.573 | 1 |  |
| Morphine | High | 50 (26.1-69) | 46.9 (26.7-66.7) | 0.265 | 1 | 17.2 (0-37) | 17.2 (2.62-36) | 0.997 | 1 | 8 (0-25) | 7.41 (0-25) | 0.915 | 1 |  |
| Noradrenaline | Low | 51 (30-70.4) | 50 (30-70.4) | 0.702 | 1 | 20 (3.57-40) | 20 (3.57-39.3) | 0.194 | 1 | 10.7 (0-29) | 10.3 (0-27.6) | 0.321 | 1 |  |
| Noradrenaline | Medium | 51.9 (30.1-72) | 50 (30.3-69.6) | 0.00492 | 0.133 | 20 (3.45-41.4) | 20.7 (3.7-40) | 0.795 | 1 | 10.7 (0-29.8) | 10.7 (0-29.2) | 0.499 | 1 |  |
| Noradrenaline | High | 54.2 (40.6-72.3) | 55.4 (40.4-72.4) | 0.668 | 1 | 28 (9.15-48.5) | 26.4 (10.4-41.9) | 0.824 | 1 | 19.6 (4-36.1) | 14.8 (0-32.3) | 0.285 | 1 |  |
| Propofol | Low | 50 (29.6-70) | 51.4 (30.8-70.4) | 0.0372 | 1 | 19.4 (3.57-39.3) | 20.6 (3.57-40) | 0.0308 | 0.832 | 10 (0-28.2) | 10.7 (0-28.6) | 0.0428 | 1 |  |
| Propofol | Medium | 50 (28.6-70.8) | 50 (30-70) | 0.775 | 1 | 18.5 (3.45-39.5) | 19.4 (3.57-39.3) | 0.114 | 1 | 8.7 (0-28) | 10.3 (0-28.6) | 0.145 | 1 |  |
| Propofol | High | 49.1 (26.5-70.8) | 50 (29.9-69) | 0.601 | 1 | 20 (3.45-40.7) | 22.2 (3.85-39.3) | 0.466 | 1 | 8.99 (0-29.8) | 11.1 (0-28.6) | 0.603 | 1 |  |
| Vasopressin | Low | 52.7 (49-56.7) | 53.4 (51.1-58.3) | 0.743 | 1 | 26.8 (25.1-31.1) | 25.1 (15-28.2) | 0.224 | 1 | 17.8 (15.8-22.3) | 16.5 (8.5-19.7) | 0.475 | 1 |  |
| Vasopressin | Medium | 53.4 (43.1-60.5) | 52.1 (48-53.4) | 0.426 | 1 | 26.7 (24.6-31.2) | 25 (12-27.6) | 0.137 | 1 | 17.8 (16.3-21.6) | 16.4 (5.84-18.9) | 0.145 | 1 |  |
| **Name** | **Mean Dose Change** | **Mean LPRx_20** | | | | **% time LPRx_20 > 0** | | | | **% time LPRx_20 > 0.25** | | | |  |
|  |  | **Pre Dose** | **Post Dose** | **P value** | **Adj P Value** | **Pre Dose** | **Post Dose** | **P value** | **Adj P Value** | **Pre Dose** | **Post Dose** | **P value** | **Adj P Value** |  |
| Dobutamine | Low | 0.0101 (-0.146-0.17) | 0.00192 (-0.169-0.158) | 0.247 | 1 | 52 (23.3-74.1) | 50 (23.1-70.2) | 0.407 | 1 | 16.1 (0-42.9) | 14.6 (0-37) | 0.0611 | 1 |  |
| Dobutamine | Medium | 0.0103 (-0.15-0.18) | 0.0174 (-0.134-0.161) | 0.873 | 1 | 52.2 (24.1-75) | 55.1 (30.8-76) | 0.675 | 1 | 16 (0-44.7) | 16.1 (0-39.5) | 0.563 | 1 |  |
| Dobutamine | High | -0.0552 (-0.202-0.107) | -0.00341 (-0.223-0.107) | 0.862 | 1 | 47.9 (23.5-65.3) | 48 (19.7-65.3) | 0.982 | 1 | 14.3 (0-33.1) | 22.2 (0-44.7) | 0.462 | 1 |  |
| Midazolam | Medium | 0.00763 (-0.131-0.139) | -0.00284 (-0.139-0.129) | 0.0213 | 0.574 | 50.9 (26.7-73.1) | 49.8 (25.9-71.4) | 0.0211 | 0.569 | 14.3 (0-36.4) | 13.3 (0-35.7) | 0.0532 | 1 |  |
| Midazolam | High | -0.00605 (-0.139-0.124) | -0.0154 (-0.148-0.113) | 0.211 | 1 | 49.1 (24.1-70.4) | 46.8 (24.9-70) | 0.396 | 1 | 11.5 (0-34.6) | 11.1 (0-32.1) | 0.343 | 1 |  |
| Morphine | Low | 0.0066 (-0.127-0.143) | 0.00828 (-0.124-0.146) | 0.399 | 1 | 51.3 (28-73.1) | 51.9 (29.3-73.1) | 0.41 | 1 | 14.9 (0-36.7) | 15.8 (0-37.9) | 0.355 | 1 |  |
| Morphine | Medium | 0.00959 (-0.14-0.145) | 0.000373 (-0.137-0.134) | 0.245 | 1 | 51.6 (26.9-75) | 50 (28.6-70) | 0.138 | 1 | 15.4 (0-37.9) | 15.4 (0-37) | 0.798 | 1 |  |
| Morphine | High | -0.0103 (-0.153-0.124) | -0.0215 (-0.164-0.109) | 0.154 | 1 | 48.3 (24.1-70.4) | 46.2 (24-68) | 0.205 | 1 | 10.3 (0-33.3) | 10.2 (0-30) | 0.722 | 1 |  |
| Noradrenaline | Low | 0.00678 (-0.136-0.147) | 0.0019 (-0.133-0.142) | 0.37 | 1 | 50 (27.8-73.1) | 50 (26.9-72.4) | 0.392 | 1 | 15.6 (0-38.5) | 14.8 (0-37) | 0.00202 | 0.0546 |  |
| Noradrenaline | Medium | 0.0129 (-0.129-0.157) | 0.00307 (-0.141-0.142) | 0.00128 | 0.0344 | 51.7 (28-75) | 50 (26.9-71.4) | 0.000293 | 0.00791 | 16 (0-39.3) | 15.4 (0-38.5) | 0.387 | 1 |  |
| Noradrenaline | High | 0.0591 (-0.0533-0.191) | 0.044 (-0.0638-0.184) | 0.713 | 1 | 56.9 (37.8-76) | 55 (39.8-76.1) | 0.798 | 1 | 25.9 (6.45-47.2) | 22.1 (10.3-41.3) | 0.707 | 1 |  |
| Propofol | Low | 0.0033 (-0.139-0.143) | 0.0109 (-0.126-0.152) | 0.000487 | 0.0132 | 50 (26.9-73.1) | 51.7 (29.2-74.1) | 0.000337 | 0.0091 | 15.4 (0-37.5) | 16.1 (0-39.3) | 0.0277 | 0.749 |  |
| Propofol | Medium | 0.00592 (-0.141-0.146) | 0.00682 (-0.133-0.148) | 0.325 | 1 | 50 (25.9-74.1) | 51.1 (28.6-73.1) | 0.158 | 1 | 14.3 (0-38.1) | 16 (0-37.9) | 0.0548 | 1 |  |
| Propofol | High | -0.00656 (-0.156-0.151) | 0.007 (-0.133-0.139) | 0.426 | 1 | 48.3 (25.9-73.1) | 51.9 (31-72) | 0.409 | 1 | 17.3 (0-38.7) | 17.9 (0-40) | 0.565 | 1 |  |
| Vasopressin | Low | 0.0278 (-0.0259-0.0617) | 0.0236 (-0.0373-0.0517) | 0.749 | 1 | 52.6 (39.9-58.4) | 52.6 (47-55.9) | 0.7 | 1 | 23.6 (18.5-26.5) | 22.4 (13.5-26.9) | 0.687 | 1 |  |
| Vasopressin | Medium | 0.0278 (-0.0412-0.0464) | 0.021 (-0.0414-0.0278) | 0.326 | 1 | 52.6 (38.7-57.7) | 51.6 (39.3-58.8) | 0.828 | 1 | 24.1 (17.5-27.1) | 22.4 (7.61-25.4) | 0.203 | 1 |  |
| **Name** | **Mean Dose Change** | **% time LPRx_20 > 0.35** | | | | **Mean LPRx_30** | | | | **% time LPRx_30 > 0** | | | |  |
|  |  | **Pre Dose** | **Post Dose** | **P value** | **Adj P Value** | **Pre Dose** | **Post Dose** | **P value** | **Adj P Value** | **Pre Dose** | **Post Dose** | **P value** | **Adj P Value** |  |
| Dobutamine | Low | 4.65 (0-27.6) | 4 (0-25.9) | 0.131 | 1 | 0.0117 (-0.149-0.18) | -0.0261 (-0.175-0.133) | 0.0105 | 0.284 | 50 (20.7-81.5) | 46 (15-75) | 0.0152 | 0.411 |  |
| Dobutamine | Medium | 5 (0-27.3) | 4.17 (0-25.9) | 0.67 | 1 | 0.0106 (-0.157-0.18) | -0.0185 (-0.161-0.146) | 0.334 | 1 | 50 (19.1-80) | 47.8 (22.7-80.8) | 0.987 | 1 |  |
| Dobutamine | High | 3.7 (0-7.41) | 14.8 (0-32.9) | 0.234 | 1 | -0.0717 (-0.248-0.07) | -0.0323 (-0.305-0.0662) | 0.794 | 1 | 40 (13.1-70.4) | 34.8 (0-67.8) | 0.595 | 1 |  |
| Midazolam | Medium | 3.7 (0-23.1) | 3.57 (0-22.2) | 0.146 | 1 | 0.00444 (-0.13-0.14) | -0.0015 (-0.135-0.128) | 0.0415 | 1 | 50.5 (21.4-78.6) | 49.3 (20.7-76) | 0.0894 | 1 |  |
| Midazolam | High | 2.13 (0-20.4) | 0 (0-18.9) | 0.482 | 1 | -0.0107 (-0.135-0.123) | -0.0115 (-0.142-0.104) | 0.213 | 1 | 46.4 (19.2-75.6) | 46.7 (18.5-72.4) | 0.316 | 1 |  |
| Morphine | Low | 3.85 (0-24) | 4.65 (0-25) | 0.0824 | 1 | 0.0137 (-0.125-0.153) | 0.011 (-0.12-0.152) | 0.895 | 1 | 51.9 (24-80) | 51.9 (24.1-79.3) | 0.803 | 1 |  |
| Morphine | Medium | 4 (0-23.8) | 4.55 (0-24.2) | 0.295 | 1 | 0.00783 (-0.138-0.147) | 0.00186 (-0.135-0.145) | 0.835 | 1 | 52 (22.2-80) | 50 (20.8-76.9) | 0.322 | 1 |  |
| Morphine | | | | | | | | | | | | | |  |
| Noradrenaline | Low | 4.3 (0-25.9) | 3.85 (0-25) | 0.0172 | 0.465 | 0.00818 (-0.136-0.148) | 0.00381 (-0.135-0.141) | 0.305 | 1 | 51.1 (22.2-78.6) | 50 (20-79.2) | 0.146 | 1 |  |
| Noradrenaline | Medium | 4.17 (0-26.7) | 5 (0-25.9) | 0.787 | 1 | 0.0122 (-0.127-0.164) | 0.00349 (-0.136-0.145) | 0.00524 | 0.141 | 51.9 (22.2-82.1) | 50 (20.8-77.8) | 0.000661 | 0.0178 |  |
| Noradrenaline | High | 15.7 (0-35.2) | 12.1 (0-27.7) | 0.351 | 1 | 0.0528 (-0.0874-0.208) | 0.07 (-0.0398-0.186) | 0.689 | 1 | 58 (32.1-78.9) | 64.3 (42.9-85.2) | 0.116 | 1 |  |
| Propofol | Low | 4 (0-25.6) | 6.45 (0-26.9) | 0.0455 | 1 | 0.00754 (-0.134-0.154) | 0.0192 (-0.115-0.169) | <0.0001 | <0.0001 | 51.7 (22.7-79.3) | 53.8 (25-81.5) | <0.0001 | <0.0001 |  |
| Propofol | Medium | 3.85 (0-25) | 4.35 (0-26.1) | 0.0607 | 1 | 0.00669 (-0.134-0.154) | 0.0144 (-0.12-0.161) | 0.000235 | 0.00635 | 50 (22.2-80) | 53.6 (25-80.5) | 0.000666 | 0.018 |  |
| Propofol | High | 4.77 (0-25.9) | 7.45 (0-27.6) | 0.516 | 1 | -0.00332 (-0.157-0.143) | 0.0212 (-0.121-0.154) | 0.0208 | 0.563 | 48.1 (20.7-79.3) | 51.7 (25-80.8) | 0.105 | 1 |  |
| Vasopressin | Low | 15.2 (9.18-20.9) | 14.1 (5.36-18.3) | 0.521 | 1 | 0.0245 (-0.151-0.0357) | 0.0267 (-0.0238-0.0643) | 0.371 | 1 | 51.5 (30.4-53.4) | 51.6 (47-59.3) | 0.251 | 1 |  |
| Vasopressin | Medium | 15.2 (10.8-20.6) | 14 (2.78-16.6) | 0.118 | 1 | 0.0256 (-0.2-0.0364) | 0.0245 (-0.11-0.0461) | 0.942 | 1 | 51.6 (29.8-54.1) | 51.5 (32.2-55.3) | 0.869 | 1 |  |
| **Name** | **Mean Dose Change** | **% time LPRx_30 > 0.25** | | | | **% time LPRx_30 > 0.35** | | | | **Mean LPRx_60** | | | |  |
|  |  | **Pre Dose** | **Post Dose** | **P value** | **Adj P Value** | **Pre Dose** | **Post Dose** | **P value** | **Adj P Value** | **Pre Dose** | **Post Dose** | **P value** | **Adj P Value** |  |
| Dobutamine | Low | 11.4 (0-42.3) | 7.41 (0-32.3) | 0.00375 | 0.101 | 0 (0-23.1) | 0 (0-20) | 0.0776 | 1 | 0.0155 (-0.154-0.212) | -0.00818 (-0.171-0.179) | 0.0848 | 1 |  |
| Dobutamine | Medium | 10.3 (0-44.4) | 7.14 (0-38.5) | 0.295 | 1 | 0 (0-25) | 0 (0-24) | 0.687 | 1 | 0.0329 (-0.131-0.207) | 0.00671 (-0.135-0.167) | 0.223 | 1 |  |
| Dobutamine | High | 0 (0-14.8) | 0 (0-22) | 0.684 | 1 | 0 (0-5.63) | 0 (0-13.6) | 0.679 | 1 | -0.0139 (-0.238-0.119) | -0.2 (-0.366-0.118) | 0.486 | 1 |  |
| Midazolam | Medium | 4.55 (0-33.3) | 4 (0-32) | 0.0385 | 1 | 0 (0-17.2) | 0 (0-16.7) | 0.203 | 1 | 0.00986 (-0.135-0.17) | 0.0125 (-0.118-0.163) | 0.042 | 1 |  |
| Midazolam | High | 0 (0-29.2) | 3.45 (0-29.2) | 0.853 | 1 | 0 (0-14.8) | 0 (0-13) | 0.9 | 1 | -0.00795 (-0.135-0.137) | -9.25e-05 (-0.13-0.127) | 0.952 | 1 |  |
| Morphine | Low | 7.69 (0-35.7) | 8.89 (0-36.6) | 0.362 | 1 | 0 (0-20.4) | 0 (0-21.4) | 0.128 | 1 | 0.0231 (-0.13-0.194) | 0.0224 (-0.116-0.184) | 0.836 | 1 |  |
| Morphine | Medium | 6.9 (0-33.3) | 7.69 (0-36.5) | 0.156 | 1 | 0 (0-18.5) | 0 (0-20.7) | 0.0512 | 1 | 0.0125 (-0.146-0.172) | 0.0114 (-0.132-0.182) | 0.499 | 1 |  |
| Morphine | High | 0 (0-27.6) | 0 (0-26.7) | 0.332 | 1 | 0 (0-11.6) | 0 (0-11.1) | 0.715 | 1 | -0.00901 (-0.139-0.131) | -0.0125 (-0.143-0.121) | 0.431 | 1 |  |
| Noradrenaline | Low | 8 (0-36) | 5.32 (0-33.3) | <0.0001 | <0.0001 | 0 (0-20.6) | 0 (0-18.6) | <0.0001 | 0.000596 | 0.0204 (-0.13-0.187) | 0.0138 (-0.128-0.173) | 0.0911 | 1 |  |
| Noradrenaline | Medium | 8 (0-37.9) | 7.14 (0-36) | 0.0176 | 0.476 | 0 (0-21.4) | 0 (0-21.4) | 0.0678 | 1 | 0.0197 (-0.136-0.195) | 0.015 (-0.132-0.184) | 0.418 | 1 |  |
| Noradrenaline | High | 21.1 (0-45.8) | 19.1 (0-44.5) | 0.881 | 1 | 9.53 (0-31.1) | 1.32 (0-26.1) | 0.169 | 1 | 0.0749 (-0.0597-0.262) | 0.0657 (-0.0607-0.258) | 0.934 | 1 |  |
| Propofol | Low | 7.69 (0-36.9) | 8 (0-40) | 0.0639 | 1 | 0 (0-21.2) | 0 (0-23.3) | 0.0528 | 1 | 0.0197 (-0.125-0.189) | 0.0299 (-0.108-0.204) | <0.0001 | 0.000212 |  |
| Propofol | Medium | 7.41 (0-36) | 8 (0-39.3) | 0.019 | 0.513 | 0 (0-20.2) | 0 (0-23.3) | 0.0224 | 0.605 | 0.0189 (-0.127-0.183) | 0.0273 (-0.109-0.202) | 0.000267 | 0.00722 |  |
| Propofol | High | 6.9 (0-35.7) | 10.9 (0-40.7) | 0.106 | 1 | 0 (0-19.8) | 0 (0-23.5) | 0.058 | 1 | 0.000213 (-0.162-0.182) | 0.024 (-0.116-0.235) | 0.0187 | 0.504 |  |
| Vasopressin | Low | 19.9 (5.65-21.9) | 20.5 (9.65-24) | 0.915 | 1 | 12.1 (2.78-17.1) | 11.9 (0-12.7) | 0.382 | 1 | 0.0158 (-0.0343-0.0616) | 0.0157 (-0.119-0.0398) | 0.761 | 1 |  |
| Vasopressin | Medium | 20.6 (2.78-21.9) | 19.7 (3.93-22.3) | 0.633 | 1 | 12.4 (0-15.7) | 11.9 (0-12.4) | 0.336 | 1 | 0.01 (-0.12-0.0244) | 0.0106 (-0.119-0.0326) | 0.749 | 1 |  |
| **Name** | **Mean Dose Change** | **% time LPRx_60 > 0** | | | | **% time LPRx_60 > 0.25** | | | | **% time LPRx_60 > 0.35** | | | |  |
|  |  | **Pre Dose** | **Post Dose** | **P value** | **Adj P Value** | **Pre Dose** | **Post Dose** | **P value** | **Adj P Value** | **Pre Dose** | **Post Dose** | **P value** | **Adj P Value** |  |
| Dobutamine | Low | 55.6 (4.65-100) | 46.2 (0-100) | 0.327 | 1 | 0 (0-44.4) | 0 (0-31.5) | 0.00247 | 0.0667 | 0 (0-21.7) | 0 (0-8.51) | 0.0203 | 0.547 |  |
| Dobutamine | Medium | 61 (9.68-100) | 51.9 (7.69-100) | 0.638 | 1 | 0 (0-44) | 0 (0-30.8) | 0.215 | 1 | 0 (0-19.6) | 0 (0-11.5) | 0.528 | 1 |  |
| Dobutamine | High | 32 (2-71.7) | 6.9 (0-69.6) | 0.337 | 1 | 0 (0-32.6) | 0 (0-22.5) | 0.8 | 1 | 0 (0-26.9) | 0 (0-16.8) | 0.679 | 1 |  |
| Midazolam | Medium | 52 (6.3-97.2) | 54.8 (8.73-100) | 0.109 | 1 | 0 (0-31) | 0 (0-29.2) | 0.489 | 1 | 0 (0-10.7) | 0 (0-7.41) | 0.052 | 1 |  |
| Midazolam | High | 46.4 (3.57-92.9) | 50 (3.57-90) | 0.977 | 1 | 0 (0-26.2) | 0 (0-25) | 0.189 | 1 | 0 (0-3.85) | 0 (0-0) | 0.119 | 1 |  |
| Morphine | Low | 57.1 (10.3-100) | 57.1 (11.1-100) | 0.559 | 1 | 0 (0-39.3) | 0 (0-36) | 0.0186 | 0.502 | 0 (0-18.2) | 0 (0-14.3) | 0.00222 | 0.0599 |  |
| Morphine | Medium | 55 (4.26-100) | 52.6 (3.85-100) | 0.889 | 1 | 0 (0-32.1) | 0 (0-34.6) | 0.239 | 1 | 0 (0-14.3) | 0 (0-15.6) | 0.727 | 1 |  |
| Morphine | High | 50 (3.57-88.9) | 45.5 (3.23-92.6) | 0.79 | 1 | 0 (0-22.1) | 0 (0-16.1) | 0.0514 | 1 | 0 (0-0) | 0 (0-0) | 0.178 | 1 |  |
| Noradrenaline | Low | 55.6 (11.1-100) | 54 (4-100) | 0.0343 | 0.927 | 0 (0-38.5) | 0 (0-32.9) | <0.0001 | <0.0001 | 0 (0-16.7) | 0 (0-10.8) | <0.0001 | <0.0001 |  |
| Noradrenaline | Medium | 56 (10.1-100) | 55.2 (3.57-100) | 0.0837 | 1 | 0 (0-39.2) | 0 (0-36.1) | 0.000172 | 0.00466 | 0 (0-17.9) | 0 (0-11.5) | <0.0001 | 0.000873 |  |
| Noradrenaline | High | 69.5 (29.2-100) | 67.9 (25.7-100) | 0.812 | 1 | 10 (0-59.1) | 0 (0-58.3) | 0.33 | 1 | 0 (0-28) | 0 (0-33.6) | 0.779 | 1 |  |
| Propofol | Low | 56 (10.7-100) | 60.6 (11.5-100) | <0.0001 | 0.000247 | 0 (0-38.5) | 0 (0-40.9) | 0.965 | 1 | 0 (0-16) | 0 (0-16.1) | 0.931 | 1 |  |
| Propofol | Medium | 55.9 (10.3-100) | 60 (11.1-100) | 0.000607 | 0.0164 | 0 (0-36.7) | 0 (0-40) | 0.842 | 1 | 0 (0-14.3) | 0 (0-15.4) | 0.931 | 1 |  |
| Propofol | High | 51.9 (3.57-96.6) | 59.3 (11.4-100) | 0.1 | 1 | 0 (0-33.6) | 0 (0-53.6) | 0.107 | 1 | 0 (0-14.3) | 0 (0-27.8) | 0.167 | 1 |  |
| Vasopressin | Low | 50.8 (35.2-77) | 50.7 (25.2-54.3) | 0.811 | 1 | 14.7 (0-15.8) | 14.5 (0-16.4) | 0.925 | 1 | 9.66 (0-10.2) | 9.66 (0-10.8) | 0.993 | 1 |  |
| Vasopressin | Medium | 49.9 (20-50.5) | 50 (2.5-53.3) | 0.647 | 1 | 14.5 (0-15.2) | 13.5 (0-15.5) | 0.966 | 1 | 8.4 (0-9.74) | 9 (0-10.2) | 0.821 | 1 |  |

*The table demonstrates the median and interquartile range of the pre/pose dose windows as well as the Wilcox signed ranked test between these windows with p values adjusted using the Bonferroni analysis. The “change” indicates if the continuous infusion was increase/decrease, note for some agent infusion we did not have the weight and thus the infusion was removed. CPP, cerebral prefusion pressure; hr, hour; ICP, intracranial pressure; IU, infusion units; kg, kilogram; LPRx_10, pressure reactivity over 10 minutes; LPRx_15, pressure reactivity over 15 minutes; LPRx_20, pressure reactivity over 20 minutes; LPRx_30, pressure reactivity over 30 minutes; LPRx_60, pressure reactivity over 60 minutes; mg, milligram; min, minutes; ml, millilitres; ug, micrograms;*

# Appendix O. Histogram Distributions of Continuous Infusion Agents

*The histograms contain all continuous infusions given with the cohort, separated dose and weight adjusted doses. hr, hour; IU, infusion units; kg, kilogram; mg, milligram; min, minutes; ml, millilitres; ug, micrograms*


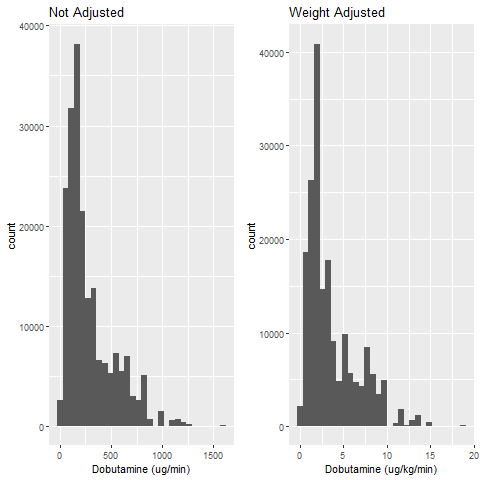


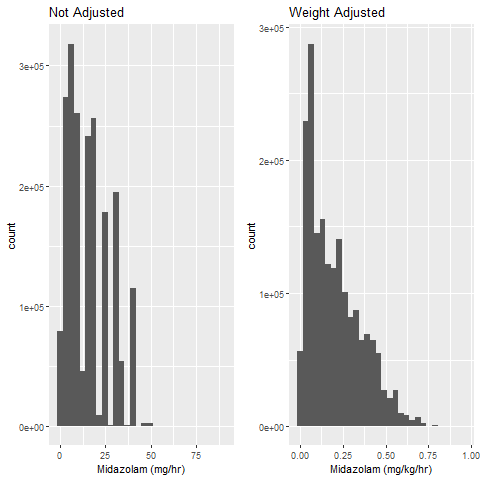

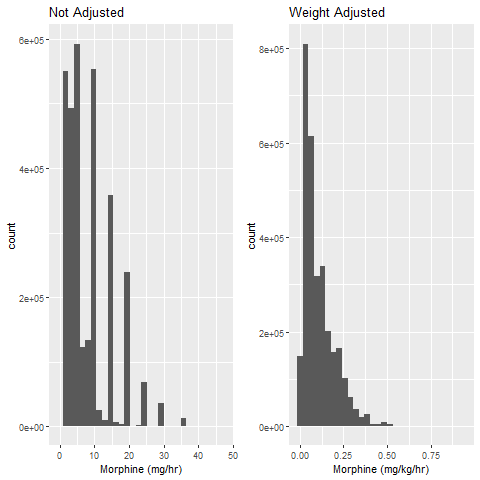

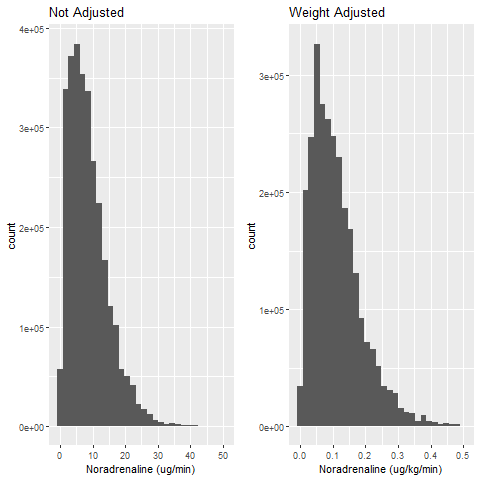

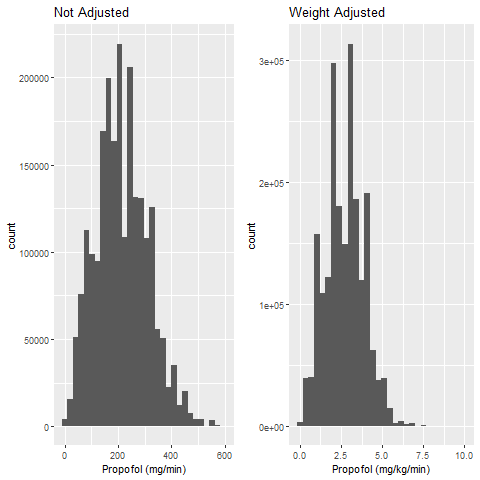

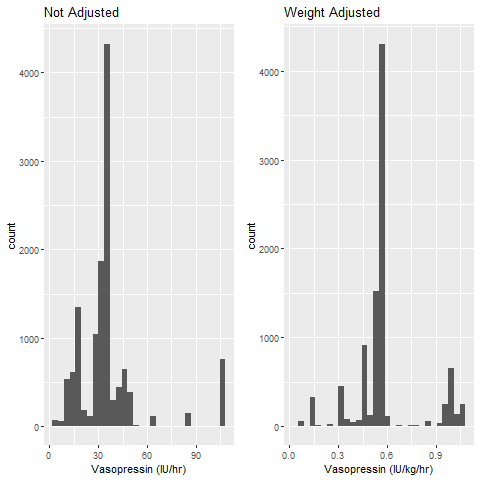

Supplement: Supplementary file 1 — Additional file 1: Appendix A. LOESS Curves – CPP/ICP/LPRx_10/LPRx_60. Appendix B. LOESS Curves – MAP/LPRx_15/LPRx_20/LPRx_30. Appendix C1. Multiple lnear model for all data. Appendix C2. Multiple linear model for sedatives data. Appendix C3. Multiple linear model for vasopressor data. Appendix D. Multiple linear model for segment Marshall CT score data. Appendix D1. Multiple linear model for Marshall CT data = 1. Appendix D2. Multiple linear model for Marshall CT data = 2. Appendix D3. Multiple linear model for Marshall CT data = 3. Appendix D5. Linear model for Marshall CT data = 5. Appendix E. One-Way ANOVA of physiology and Marshall CT score. Appendix F. Infusions of all data. Appendix G. Pre-time window over 50% time ICP > 20 mmHg. Appendix H. Pre-time window over 50% time ICP < 20 mmHg. Appendix I. Pre-time window over 50% time L-PRx_10 > 0. Appendix J. Pre-time window over 50% time L-PRx_10 < 0. Appendix K. Pre-time window over 50% time L-PRx_10 > 0.35. Appendix L. Pre-time window over 50% time L-PRx_10 < 0.35. Appendix M. Continuous infusion going from nothing to agent (and vice versa ie, On to Off). Appendix N. Assessing the High/Medium/Low of different infusion agent. Appendix O. Histogram distributions of continuous infusion agents. [file 40635_2023_524_MOESM1_ESM.docx]
